# Three-component vicinal-diarylation of alkenes *via* direct transmetalation of arylboronic acids†
†Electronic supplementary information (ESI) available. CCDC 1852875 and 1852877. For ESI and crystallographic data in CIF or other electronic format see DOI: 10.1039/c9sc02182e


**DOI:** 10.1039/c9sc02182e

**Published:** 2019-07-03

**Authors:** Yun Zhang, Gong Chen, Dongbing Zhao

**Affiliations:** a State Key Laboratory and Institute of Elemento-Organic Chemistry , College of Chemistry , Nankai University , 94 Weijin Road , Tianjin 300071 , China . Email: gongchen@nankai.edu.cn ; Email: dongbing.chem@nankai.edu.cn

## Abstract

Herein, we report three-component vicinal-diarylation of non-conjugated alkenes initiated by transmetalation of arylboronic acids, which provides complementary access to β,γ-diaryl carbonyl compounds. We have also screened a large number of chiral ligands for developing an enantioselective version of this reaction and obtained the preliminary results (up to 79 : 21 e.r.). Notably, the methodology developed herein represents the first three component *syn*-vicinal-dicarbofunctionalization of non-conjugated alkenes involving palladium catalysis.

## Abstract 2

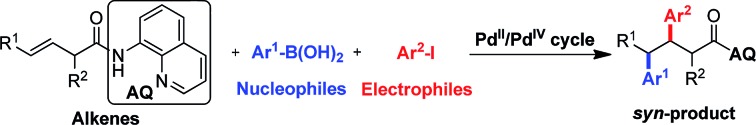
Transmetalation-initiated three-component vicinal-diarylation of alkenes.

## Introduction

**Scheme 1:**
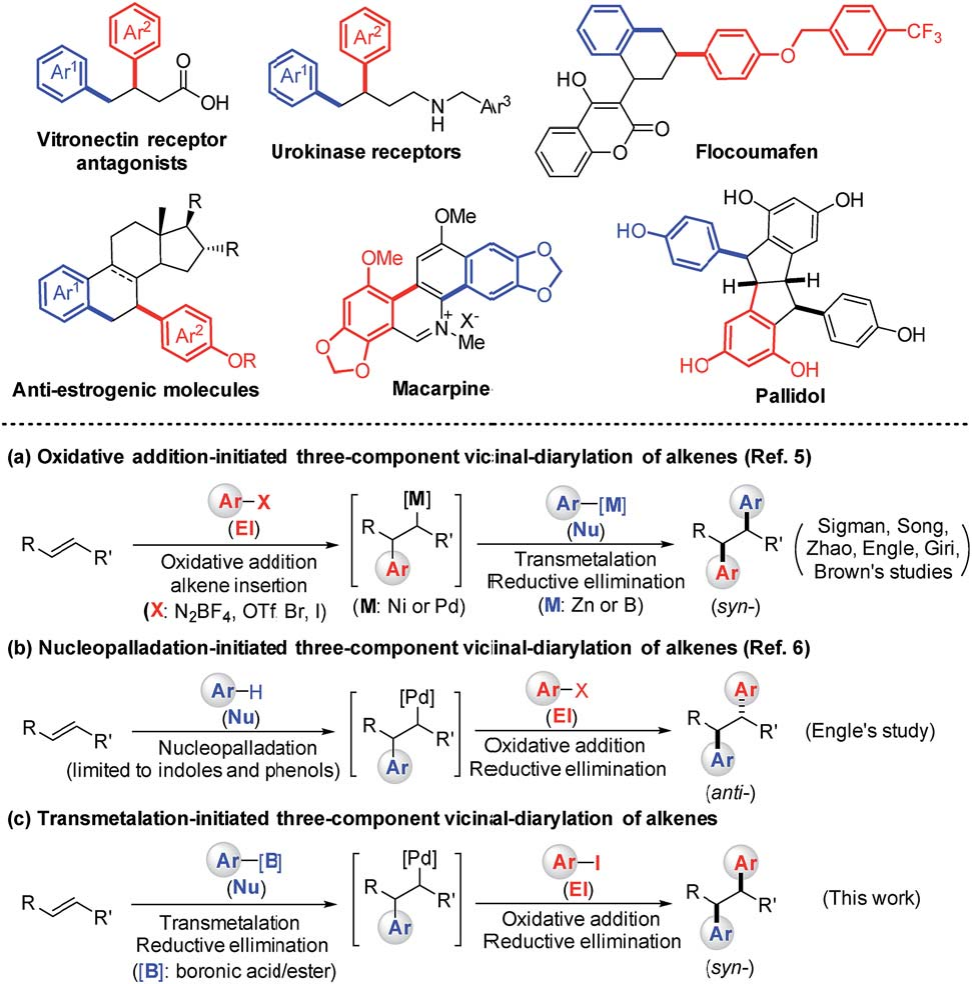
The usefulness of β,γ-diaryl carbonyl structures as well as the previous work and our design of three-component diarylation of olefins.

Vicinal-diaryl structures are a common scaffold in various medicinally relevant molecules and can also be widely utilized as chemical feedstocks to access a number of natural products (Scheme 1, upper panel).^1^ Traditional reactions to access vicinal-diaryl structures generally suffer from disadvantages such as multi-step processes, limited substrate scope, and difficulties in accessing starting materials.^1^,^2^

Alkenes are among the most abundant classes of organic molecules, available in bulk quantities from petrochemical feedstocks and renewable resources. Thus, the intermolecular diarylation of olefins represents one of the most powerful and straightforward methods to rapidly produce vicinal diaryl structures.^3^ For example, homodiarylation of alkenes with aryl metals or halides is a quick method to introduce two identical aryl groups across an olefin.^4^ On the other hand, two general approaches for the introduction of two different aryl moieties across an olefin have been developed: (1) a sequence involving the formation of an Ar–MX species *via* oxidative addition of an M^0^ catalyst (Ni^0^ or Pd^0^) to an aryl electrophile (El), alkene insertion, transmetalation with the aryl nucleophiles (Nu), and C–C reductive elimination (Scheme 1a, lower panel);^5^ (2) π-Lewis acid (Pd^II^ as the Lewis acid) activation of the alkene to enable attack of an aryl C–H nucleophile (limited to indoles and phenols) to form a carbopalladated Wacker-type intermediate, followed by the oxidative addition of aryl electrophiles (Ar–X) and C–C reductive elimination (Scheme 1b, lower panel).^6^

Transmetalation of aryl metal reagents with a palladium(ii) catalyst to generate the Ar–PdX intermediate has been well documented.^7^ In recent years, several groups have developed an array of methods for alkene functionalization triggered by Pd^II^-catalyzed transmetalation of arylboronic acids such as the oxidative boron Heck reaction.^8^,^9^ We thus questioned whether three-component heterodiarylation of olefins with aryl halides and arylboronic acids could also be initiated by a transmetalation step (Scheme 1c, lower panel). Such a reaction would provide complementary access to the previous two approaches to vicinal-diaryl structures. Herein, we report the first transmetalation-initiated three-component vicinal-diarylation of γ-olefinic acids by using a directing-group strategy, which enables rapid construction of *syn*-β,γ-diaryl carbonyl compounds.

## Results and discussion

In a preliminary experiment, a 3-butenoic acid derivative **1a** bearing an 8-aminoquinoline (AQ)-directing group (0.3 mmol) was treated with Pd(OAc)_2_ (10 mol%), K_2_CO_3_ (0.3 mmol), 2-phenylboronic acid **2a** (0.6 mmol) and 4-methoxyphenyl iodide **3a** (0.9 mmol) in hexafluoroisopropanol (HFIP, 2 mL) at 100 °C for 24 h, which was completely unreactive (Table 1, entry 1). Inspired by Toste's work,^10^ we changed the solvent to a mixture of DCM/H_2_O/CH_3_CN (2 mL : 0.4 mL : 0.2 mL) and the base to Na_2_CO_3_ (0.3 mmol). Then a variety of palladium(ii) catalysts were evaluated (Table 1, entries 2–8). (–)-SparteinePdCl_2_ was proven to be the best choice (entry 5). Subsequently, different bases were also screened (entries 9–11). All others gave inferior results. We further found that the addition of extra (–)-sparteine (20 mol%) would increase the yield to 83% (entry 12). The effect of each component of the mixed solvents was also investigated (entries 13–16). It has been proven that all the three solvents are necessary to ensure a satisfactory yield. Switching the solvent back to HFIP dramatically shut off the reaction (entry 17). Notably, we also checked the enantioselectivity of this reaction under the optimized conditions developed in this study. However, the product **4aa** was always racemic at this stage. Different types of aryl boron compounds were also screened (entries 18–19).

**Table 1:** Optimization of the directed three-component *syn*-vicinal-diarylation of alkene **1a** with phenylboronic acids **2a** and 4-methoxyphenyl iodide **3a**^*a*^

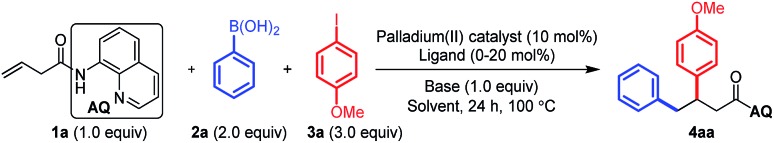				
Entry	Pd^II^ cat.	Base	Solvent	Yield^*b*^ [%]
1	Pd(OAc)_2_	K_2_CO_3_	HFIP (2 mL)	NR
2	Pd(OAc)_2_	Na_2_CO_3_	DCM/H_2_O/MeCN (1 : 0.2 : 0.1)	54
3	PdCl_2_	Na_2_CO_3_	DCM/H_2_O/MeCN (1 : 0.2 : 0.1)	44
4	Pd(PhCN)_2_Cl_2_	Na_2_CO_3_	DCM/H_2_O/MeCN (1 : 0.2 : 0.1)	69
5	(–)-SparteinePdCl_2_	Na_2_CO_3_	DCM/H_2_O/MeCN (1 : 0.2 : 0.1)	71
6	(–)-SparteinePd(OAc)_2_	Na_2_CO_3_	DCM/H_2_O/MeCN (1 : 0.2 : 0.1)	6
7	(Bipy)PdCl_2_	Na_2_CO_3_	DCM/H_2_O/MeCN (1 : 0.2 : 0.1)	60
8	PddppfCl_2_	Na_2_CO_3_	DCM/H_2_O/MeCN (1 : 0.2 : 0.1)	11
9	(–)-SparteinePdCl_2_	K_2_CO_3_	DCM/H_2_O/MeCN (1 : 0.2 : 0.1)	66
10	(–)-SparteinePdCl_2_	Cs_2_CO_3_	DCM/H_2_O/MeCN (1 : 0.2 : 0.1)	44
11	(–)-SparteinePdCl_2_	K_3_PO_4_	DCM/H_2_O/MeCN (1 : 0.2 : 0.1)	53
**12** ^*c*^	**(–)-SparteinePdCl**_**2**_	**Na**_**2**_ **CO**_**3**_	**DCM/H**_**2**_ **O/MeCN (1 : 0.2 : 0.1)**	**83**
13^*c*^	(–)-SparteinePdCl_2_	Na_2_CO_3_	DCM/H_2_O/MeCN (1 : 0.1 : 0.1)	72
14^*c*^	(–)-SparteinePdCl_2_	Na_2_CO_3_	DCM/H_2_O (1 : 0.2)	77
15^*c*^	(–)-SparteinePdCl_2_	Na_2_CO_3_	DCM/CH_3_CN (1 : 0.1)	14
16^*c*^	(–)-SparteinePdCl_2_	Na_2_CO_3_	DCM (2 mL)	18
17^*c*^	(–)-SparteinePdCl_2_	Na_2_CO_3_	HFIP (2 mL)	NR
18^*c*^ ^*d*^	(–)-SparteinePdCl_2_	Na_2_CO_3_	DCM/H_2_O/MeCN (1 : 0.2 : 0.1)	81
19^*c*^ ^*e*^	(–)-SparteinePdCl_2_	Na_2_CO_3_	DCM/H_2_O/MeCN (1 : 0.2 : 0.1)	35

^*a*^Reactions were carried out by using Pd^II^ catalyst (10 mol%), ligand (0–20 mol%), base (0.3 mmol, 1.0 equiv.), **1a** (0.3 mmol, 1.0 equiv.), phenylboronic acid **2a** (2.0 equiv.), and 4-methoxyphenyl iodide **3a** (3.0 equiv.) in the mixture of DCM/H_2_O/CH_3_CN (2 mL : 0.4 mL : 0.2 mL) for 24 h at 100 °C under a N_2_ atmosphere.

^*b*^Isolated yields.

^*c*^(–)-Sparteine (20 mol%) was added.

^*d*^(PhBO)_3_ instead of **2a**.

^*e*^PhBNep instead of **2a**.

**Scheme 2:**
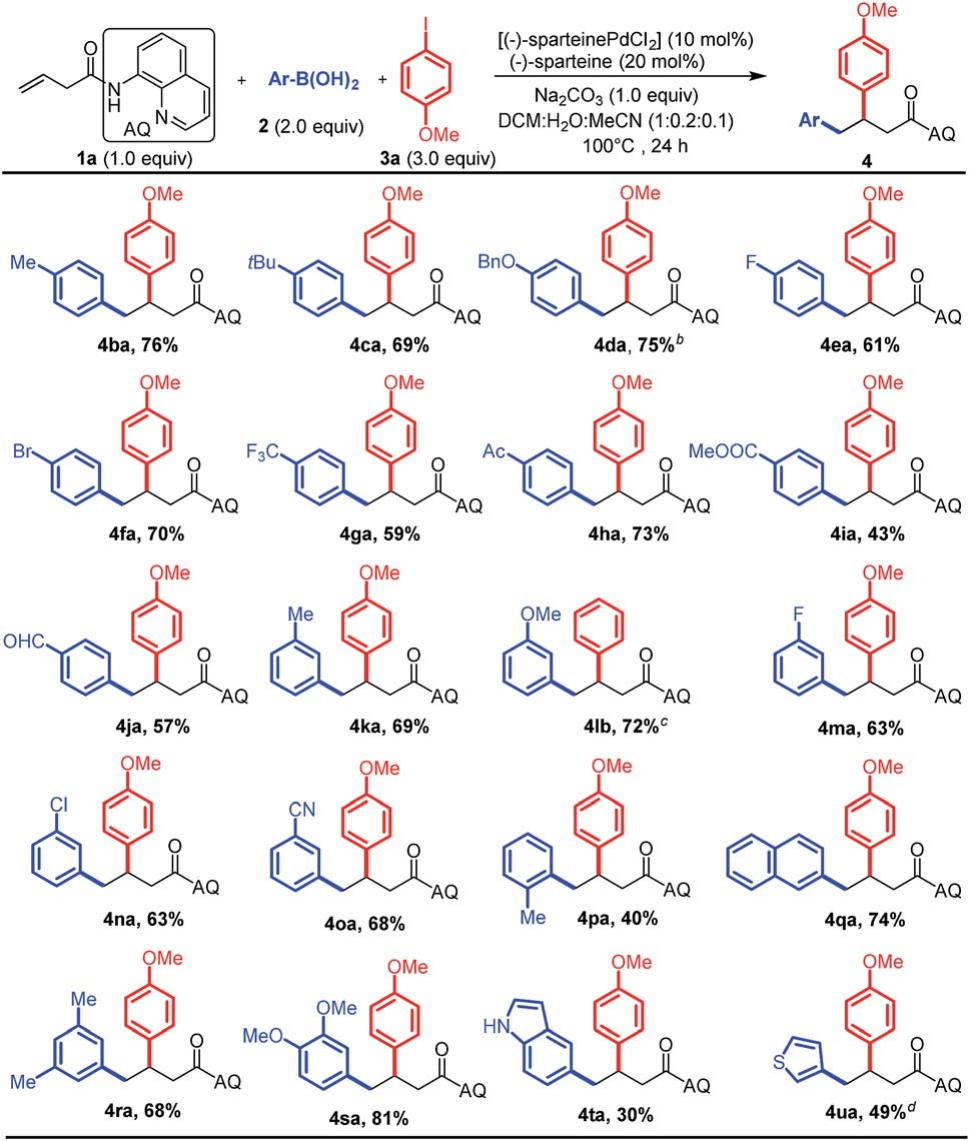
The substrate scope of aryl boronic acid nucleophiles.^a *a*^Reactions were carried out by using (–)-sparteinePdCl_2_ (0.03 mmol, 10 mol%), (–)-sparteine (0.06 mmol, 20 mol%), Na_2_CO_3_ (0.3 mmol, 1 equiv.), **1a** (0.3 mmol, 1.0 equiv.), arylboronic acid **2** (0.6 mmol, 2.0 equiv.), and 4-methoxyphenyl iodide **3a** (0.9 mmol, 3.0 equiv.) in the mixture of DCM/H_2_O/CH_3_CN (2 mL : 0.4 mL : 0.2 mL) for 24 h at 100 °C under a N_2_ atmosphere. ^*b*^Alkene **1a** (0.2 mmol) was used in the reaction. ^*c*^Iodobenzene was used as the electrophile; ^*d*^the reaction was conducted for 48 h and 4.0 equiv. of **3a** were used.

Having optimized the reaction conditions, we first investigated the substrate scope of aryl boronic acid nucleophiles by using the 3-butenoic acid derivative **1a** as the alkene and 4-iodoanisole **3a** as the electrophile (Scheme 2). To our delight, the reaction was found to tolerate an array of functional groups on the aromatic ring of boronic acid, including electron-neutral, electron-donating and electron-withdrawing substituents, at both the *para*- and *meta*-positions (**4ba–oa**). Substituents on the *ortho*-position led to a decreased yield, presumably due to steric effects (**4pa**). Multi-substituted aryl boronic acids (**4ra–sa**) and heteroaryl boronic acids were also reactive (**4ta–ua**). It is important to stress that these reaction conditions were compatible with a variety of functional groups such as halogens (F, Cl, and Br) and acetyl, ester, aldehyde, cyano, and methoxy groups on the aryl boronic acids, which could be subjected to further synthetic transformations.

**Scheme 3:**
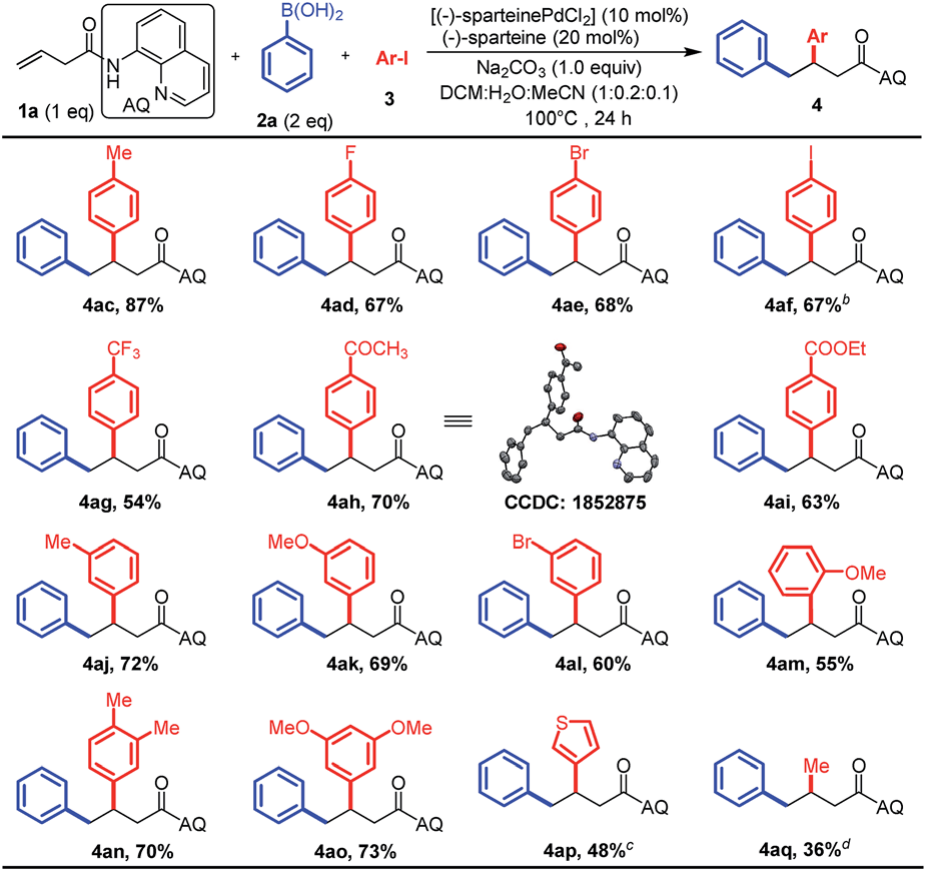
The scope of aryl iodide electrophiles.^a *a*^Reactions were carried out by using (–)-sparteinePdCl_2_ (0.03 mmol, 10 mol%), (–)-sparteine (0.06 mmol, 20 mol%), Na_2_CO_3_ (0.3 mmol, 1.0 equiv.), **1a** (0.3 mmol, 1.0 equiv.), phenylboronic acid **2a** (0.6 mmol, 2 equiv.), and aryl iodide **3** (3–10 equiv.) in the mixture of DCM/H_2_O/CH_3_CN (2 mL : 0.4 mL : 0.2 mL) for 24 h at 100 °C under a N_2_ atmosphere. ^*b*^A by-product **4af′** with 8% yield was observed in this reaction from the activation of the second C–I bond in the aromatic ring. ^*c*^The reaction was conducted for 36 h and 4.0 equiv. of aryl iodides were used. ^*d*^The reaction was conducted for 48 h and 10.0 equivalent of methyl iodide were used.

Subsequently, a variety of substituted aryl and heteroaryl iodide electrophiles were tested under the optimized conditions, and the results are summarized in Scheme 3. It was gratifying to find whether aryl iodides are electron-rich, electron-poor, or sterically hindered, and all of them afforded moderate to high yields (**4ac–an**). Heteroaryl iodides were also competent coupling partners (**5n**). Notably, a lot of important functional groups such as halogens (F, Br, and I), esters, and acetyl groups on the aryl iodides were tolerated, presenting the opportunity for subsequent diversification. Besides diverse (hetero)aryl iodides, the employment of methyl iodide as the electrophile also smoothly delivered the desired products in moderated yields in this reaction (Scheme 3, **4aq**). X-ray analysis of a single crystal **4ah** clearly confirmed that the diaryl groups of the structural connection order in the product is opposite to those vicinal-diaryl products produced by M^0^ (Ni^0^ or Pd^0^)-catalyzed diarylation of alkenes with aryl electrophiles and nucleophiles.^5^

**Scheme 4:**
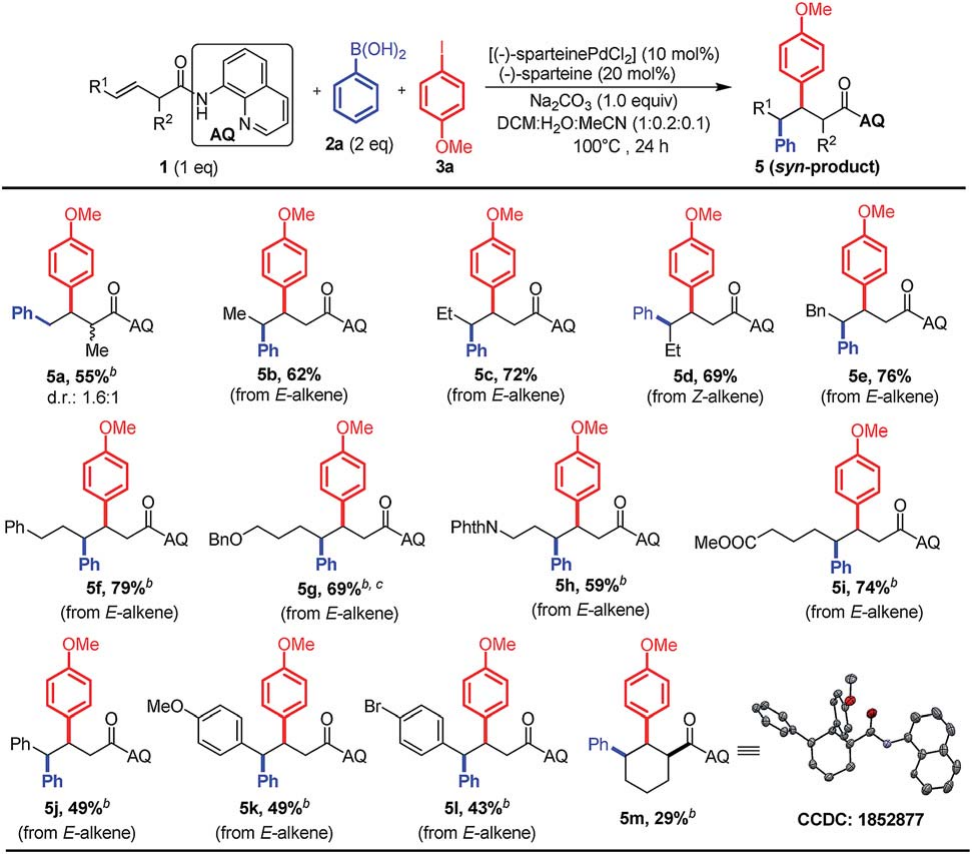
The scope of various non-conjugated alkenes.^a *a*^Reactions were carried out by using (–)-sparteinePdCl_2_ (0.03 mmol, 10 mol%), (–)-sparteine (0.06 mmol, 20 mol%), Na_2_CO_3_ (0.3 mmol, 1.0 equiv.), **1** (0.3 mmol, 1.0 equiv.), phenylboronic acid **2a** (0.6 mmol, 2 equiv.), and 4-methoxyphenyl iodide **3a** (0.9 mmol, 3 equiv.) in the mixture of DCM/H_2_O/CH_3_CN (2 mL : 0.4 mL : 0.2 mL) for 24 h at 100 °C under a N_2_ atmosphere. ^*b*^The reaction was conducted for 36 h and 4.0 equiv. of **3a** were used. ^*c*^An alkene substrate (0.2 mmol) was used in the reaction.

We next evaluated the utility of this method for various non-conjugated alkenes by using phenylboronic acid **2a** as the nucleophile and 4-methoxyphenyl iodide **3a** as the electrophile (Scheme 4). The terminal alkene bearing mono-substitution at the α-position proceeded smoothly to afford the desired product in moderate yield; nevertheless harsher conditions are needed (Scheme 4, **5a**). Furthermore, we proved that a variety of internal alkenes could also be efficiently converted into the *syn*-diastereomers with nearly negligible electronic effects of substituents (Scheme 4, **5b–m**). The *syn*-selectivity of the reaction was confirmed by X-ray analysis of the crystal structure of **5m**,^11^ which was in accordance with our proposed mechanism. Notably, both diastereomers could be accessed based on the stereochemistry of the alkene substrate (*i.e.*, *E*-alkene to **5c** and *Z*-alkene to **5d**), suggesting that the alkene does not undergo isomerization in the Pd-catalyzed process used in this study. In addition, a pendant phthalimide-protected amine, benzyl-protected alcohol and ester were also well tolerated in the reaction (**5g–i**).

In light of our success in the transmetalation-initiated three-component vicinal-diarylation of alkenes to synthesize racemic *syn*-β,γ-diaryl carbonyl compounds, we have tried to develop the enantioselective version of this reaction. After much work on ligand evaluation and optimization of the reaction conditions (see Fig. S2 and S3 in the ESI† for details of the optimization studies), we identified a pyridyl-oxazolidine ligand **L41** as an effective ligand, producing a chiral product with moderate enantioselectivity (79 : 21 e.r.) and 60% yield (eqn (1)).1
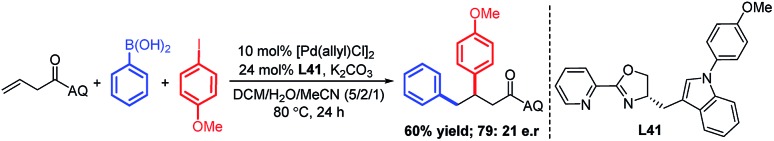



**Scheme 5:**
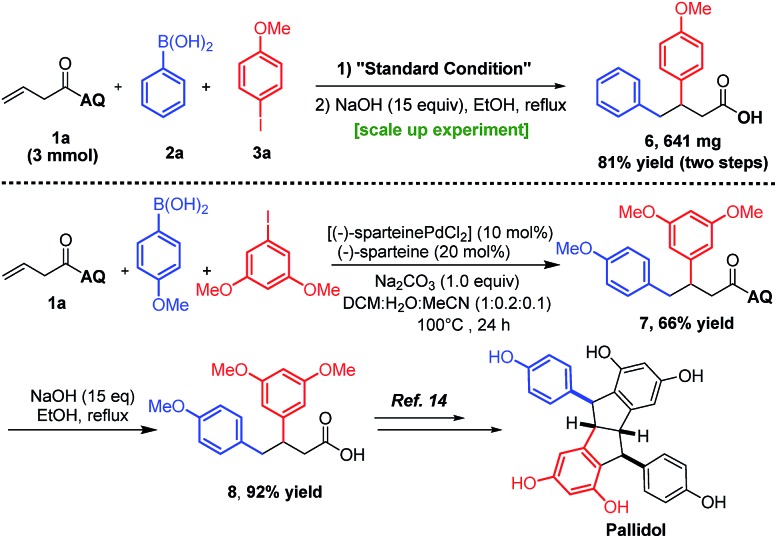
The synthetic usefulness of our method.

To demonstrate the practicality of this method, we first scaled up the reaction to obtain ∼1 g of **4aa** (Scheme 5, upper panel). Then, the AQ-directing group was directly removed by treatment with NaOH to yield the free carboxylic acid **6***via* two steps with an overall yield of 81%. Furthermore, the carboxylic acid **8**, which has been employed as the intermediate to obtain the natural product pallidol, has been efficiently synthesized with 92% yield by using the Pd(ii)-catalyzed method (Scheme 5, lower panel). In contrast, the methods in the literature involved a non-catalytic procedure with three steps (86% yield), which shows the advantage and usefulness of our method in the modular synthesis of β,γ-diaryl carbonyl compounds.^12^

**Scheme 6:**
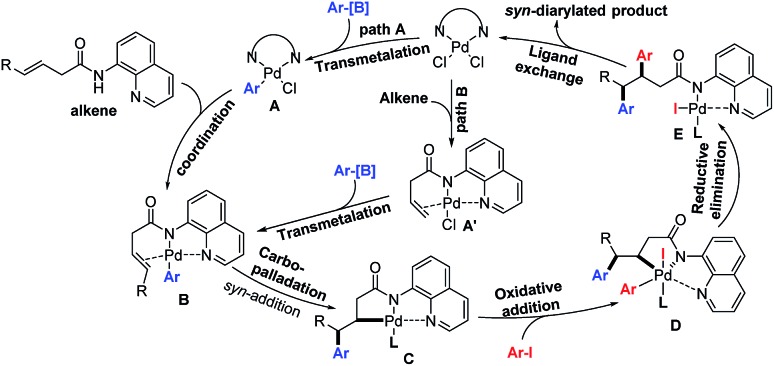
Proposed catalytic cycle of this three-component vicinal-diarylation of alkenes.

On the basis of literature reports,^7^–^9^ we propose a Pd^II^/Pd^IV^ catalytic cycle that starts with the transmetalation (TM) of arylboronic acids to form Ar–PdX species **A** (Scheme 6). Then the Ar–PdX species would coordinate with the directing group and alkene to form the intermediate **B**, which would involve the carbopalladation step resulting in the intermediate **C**. Because of the exclusive *syn*-addition during the carbopalladation step, the stereoselectivity of the as-synthesized diarylated products is opposite to the alkenes produced by the diarylation method involving a Wacker-type *anti*-nucleopalladation step.^6^ Moreover, the intermediate **C** would be intercepted by the oxidative addition of aryl iodides to form Pd^IV^ species **D**. Finally, reductive elimination from the high-valent palladium center and subsequent ligand exchange produce the product and regenerate the Pd^II^ catalyst.^13^ Alternatively, alkene coordination with the Pd^II^ catalyst could also happen first to form the intermediate **A′**, and then the transmetalation of aryl boronic acids to form the intermediate **B**.

To support our aforementioned continuous mechanism, several control experiments were performed. We found that no reaction occurred when *N*-(naphthalenyl)butenamide **9** was used as the alkene substrate (eqn (2)). This result indicates that the presence of the AQ directing group on the unconjugated alkene was indispensable. The Heck reaction between aryl iodides and alkene **1a** did not happen under the optimized conditions and 80% starting material **1a** was recovered (eqn (3)). It indicates that the Pd^0^ species might not be formed in this reaction system. Notably, the homodiarylated product with 8% yield was observed (eqn (3)). To obtain the intermediate **C**, the reaction of alkene **1a** and phenylboronic acids **2a** was run in the presence of a stoichiometric amount of the Pd^II^ catalyst (eqn (4)). The Heck product **1k** (40% yield) and the starting material **1a** (51% recovery) were isolated. It means that the transmetalation of aryl boronic acids and carbopalladation would preferentially occur in the presence of the Pd^II^ catalyst, which follows a β-H elimination step in the absence of an electrophile to form the Heck product. To rule out the possibility of the Heck product **11** as the intermediate for this three-component vicinal-diarylation of alkenes, we carried out the reaction of the Heck product **11** with aryl iodides with a stoichiometric amount of the Pd^0^ catalyst (eqn (5)). No desired product **4aa** was observed. Finally, we exclude the possibility of the hydrocarbofunctionalized product **13** as the competent intermediate because only 7% diarylated product **4aa** was yielded when the prepared hydrocarbofunctionalized intermediate **13** was exposed to the standard conditions in the absence of additional nucleophiles (eqn (6)).2
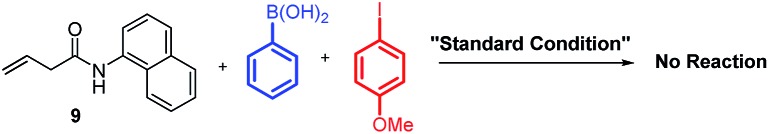

3
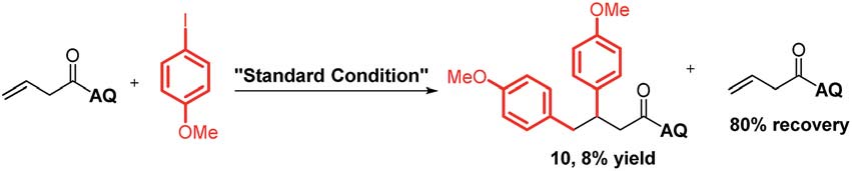

4
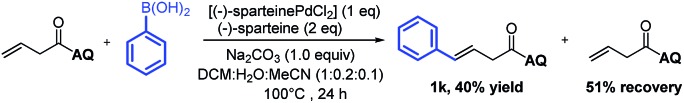

5
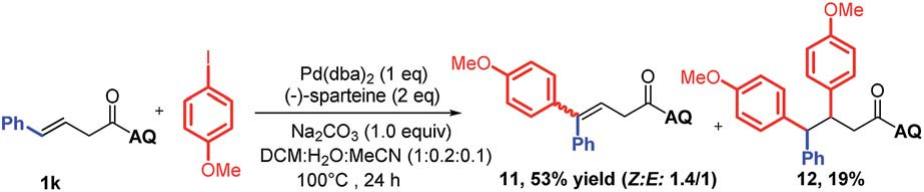

6
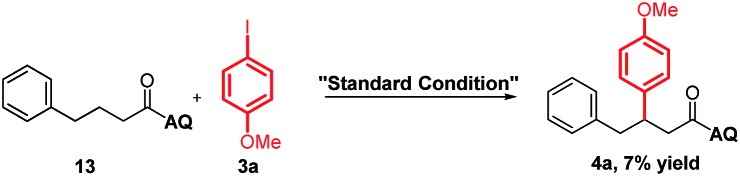



## Conclusions

In conclusion, we have described the transmetalation-initiated three-component *syn*-β,γ-diarylation of olefins with aryl halides and arylboronic acids. The process is not only simple and convenient in terms of the reaction conditions, but also tolerates a variety of functional groups, thus constituting a practical route to β,γ-diaryl carbonyl compounds prevalent in bioactive molecules.

## Conflicts of interest

There are no conflicts to declare.

## Supplementary Material

Supplementary information

Crystal structure data
