# Supplementary material for: Three-component vicinal-diarylation of alkenes via direct transmetalation of arylboronic acids
Source: Chem Sci. 2019 Jul 3;10(34):7952–7. doi: 10.1039/c9sc02182e (PMC6836572; doi:10.1039/c9sc02182e)
Supplement: Supplementary file 1 [file SC-010-C9SC02182E-s001.pdf]

Electronic Supplementary Material (ESI) for Chemical Science.
This journal is © The Royal Society of Chemistry 2019

Three-component vicinal-diarylation of alkenes via direct transmetalation of arylboronic Acids

Yun Zhang, Gong Chen*, and Dongbing Zhao*

State Key Laboratory and Institute of Elemento-Organic Chemistry, College of Chemistry, Nankai University, Tianjin 300071, China

E-mail: gongchen@nankai.edu.cn; dongbing.chem@nankai.edu.cn

Table of Contents

1. General Information.. S2

2. Experimental Procedures......................... ....S2

2.1 General Procedure for Synthesis of Alkene Substrates................S2

2.2 General Procedure for *β*,*γ*-Diarylation of Alkenes...................... S4

3. Characterization of New Products **4-6**...S5

4 Screening of Asymmetric Reaction Conditions.................................S42

5. Removal of Directing Group & Derivative reaction.........................S46

6. Control Experiments & Mechanistic Experiments............................S50

7. X-Ray Crystallography Data.. S53

8. References.. S55

9. HPLC Trace and NMR Spectra.. S57

1. General Information

Commercially available reagents were received from commercial sources without further purification and Dry solvents (<50 ppm H_2O) were purchased and stored over molecular sieves under N_2 atmosphere and were transferred under N_2.

NMR spectra were recorded on Bruker AV 400 spectrometer at 400 MHz (^{1}H NMR), 100 MHz (^{13}C NMR), 376 MHz (^{19}F NMR). Chemical shifts (δ) for ^{1}H and ^{13}C NMR spectra are given in ppm relative to TMS or residual solvent signals. ^{1}H, ^{13}C and ^{19}F multiplicities are reported as follows: singlet (s), doublet (d), triplet (t), quartet (q), doublet of doublets (dd), triplet of quartets (tq), multiplet (m), and broad resonance (br). High resolution mass spectra (HRMS) were recorded on an Agilent 6520 Q-TOF LC/MS with Electron Spray Ionization (ESI) resource. Thin-layer chromatographies were done on pre-coated silica gel 60 F254 plates (Merck). Silica gel 60H (300-400 mesh) manufactured by Qingdao Haiyang Chemical Group Co. (China) was used for general chromatography. Enantiomeric excess (ee) was determined on Thermo Scientific Dionex UltiMate 3000 Standard Systems using Darcel Chiral IA-3.

2. Experimental Procedures

2.1 General Procedure for Synthesis of Alkene Substrates

Step 1:

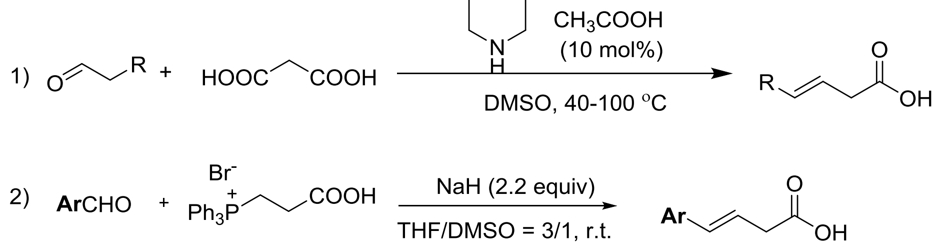

Step 2:

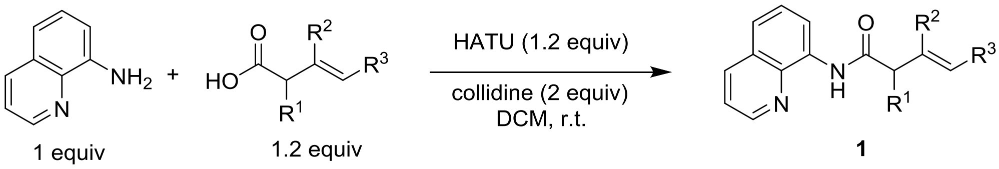

Step 1:

1) The alkyl-substituted olefinic carboxylic acids were synthesized according to the reported procedures.[1, 2] To a stirred solution of aldehyde in DMSO (1M), malonic acid (2.0 equiv), acetic acid (6 μL) and piperidine (10 μL) were added in one portion. The mixture was heated at 40 ℃ for 4 h and then heat to 100 ℃ for 12 h. After been cooled to room temperature, the reaction mixture was poured into brine and extracted with ethyl acetate for several times. The combined organic layer was dried over Na_2SO_4, filtered and concentrated in vacuo. The crude product was used without purification in the next step.

2) The aryl-substituted olefinic carboxylic acids were synthesized according to the reported procedures.[3] To a suspension of Wittig salt (1.1 equiv) in THF (15 mL) and DMSO (5 mL), NaH (2.2 equiv) was added at room temperature. After the evolution of hydrogen gas, aldehyde (1 equiv) was added slowly at room temperature. The mixture was stirred at this temperature overnight and then acidified with 1N HCl. After extraction with ethyl acetate for several times, organic layer was dried over Na_2SO_4, and concentrated. The crude material was purified by column chromatography. If needed, the products can be further purified by recrystallization.

Step 2：

Following the literatures' procedure,[1-5] corresponding vinyl acetic acid (1.2 equiv) was charged into a 250 mL RB flask containing dichloromethane (0.2 M). 8-Amino-quinoline (1 equiv), collidine (2 equiv), and HATU (1.2 equiv) were added sequentially and the reaction was stirred at ambient temperature for 16-24 h. The solvent was removed by evaporation. Then the residue was dissolved in EtOAc (100 mL), washed with sat. $NaHCO_3$ (2 × 70 mL), brine (1 × 70 mL) and purified by column chromatography (10-15% EtOAc in Hexanes) to afford the target product.

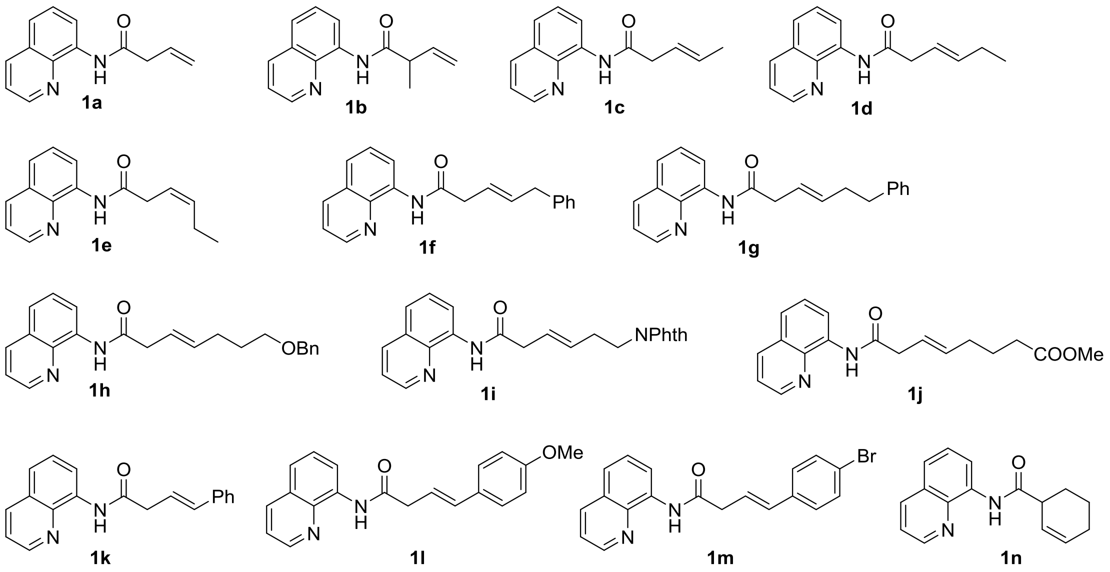

Figure S1. Summary of substrates **1**

2.2 General Procedure for *β,γ*-Diarylation of Alkenes

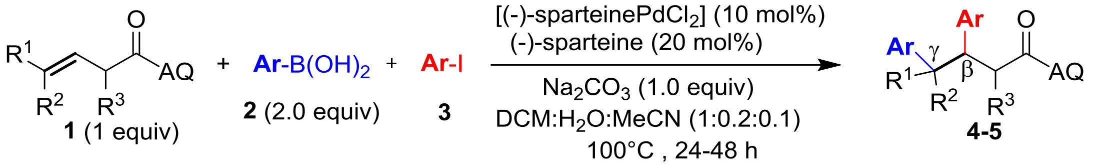

Alkene substrate (0.3 mmol, 1.0 equiv), arylboronic acid (0.6 mmol, 2.0 equiv), the appropriate carbon electrophile (3.0−10.0 equiv), Pd[(-)-sparteine]Cl_2 (12.3 mg, 0.03 mmol, 10.0 mol%), Na_2CO_3 (31.8 mg, 0.3 mmol, 1.0 equiv) were added to a 25 mL glass vial containing a stirring bar. After purged with N_2, DCM (2 mL), H_2O (0.4 mL) and MeCN (0.2 mL) and (-)-sparteine (14.0 mg, 0.06 mmol, 20 mol%) were added, The tube was sealed with a teflon cap and placed in a hotplate pre-heated to 100 ℃ with vigorous stirring for 24−48 h. After been cooled to room temperature, the reaction mixture was dried over Na_2SO_4 and filtered through a pad of celite and concentrated in vacuo. The resulting residue was purified by silica gel flash chromatography to give the desired product.

3. Characterization of Products 4−6

3-(4-Methoxyphenyl)-4-phenyl-*N*-(quinolin-8-yl)butanamide (4aa)

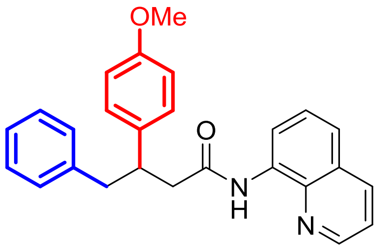

Following the general procedure, the reaction was carried out with **1a** (0.3 mmol, 1.0 equiv), phenylboronic acid **2a** (0.6 mmol, 2.0 equiv), 4-methoxyphenyl iodide **3a** (0.9 mmol, 3.0 equiv), Pd[(-)-sparteine]Cl_2 (0.03 mmol, 10 mol%), (-)-sparteine (0.06 mmol, 20 mol%), Na_2CO_3 (31.8 mg, 0.3 mmol) in the mixture of DCM/H_2O/CH_3CN (2 mL:0.4 mL:0.2 mL) for 24 h at 100 ℃ under a N_2 atmosphere. 3-(4-Methoxyphenyl)-4-phenyl-*N*-(quinolin-8-yl)butanamide (**4aa**) was isolated by column chromatography (PE/acetone = 11/1) as a white solid in 83 % yield. **^{1}H NMR (400 MHz, CDCl₃)**: δ 9.66 (s, 1H), 8.75 (dd, *J* = 4.2, 1.6 Hz, 1H), 8.70 (dd, *J* = 7.1, 1.7 Hz, 1H), 8.12 (dd, *J* = 8.3, 1.5 Hz, 1H), 7.52 – 7.44 (m, 2H), 7.42 (dd, *J* = 8.3, 4.2 Hz, 1H), 7.25 – 7.06 (m, 7H), 6.78 (d, *J* = 8.6 Hz, 2H), 3.72 (s, 3H), 3.66 – 3.52 (m, 1H), 3.02 (ddd, *J* = 21.5, 13.5, 7.4 Hz, 2H), 2.88 (qd, *J* = 14.8, 7.4 Hz, 2H). **^{13}C NMR (100 MHz, $CDCl_3$)**: δ 170.27, 158.17, 148.04, 139.78, 138.27, 136.32, 135.57, 134.44, 129.41, 128.63, 128.22, 127.89, 127.39, 126.10, 121.59, 121.42, 116.45, 113.88, 55.17, 44.32, 43.45, 43.15. **HRMS (ESI)** m/z Calcd. for $C_{26}H_{24}N_2O_2$ $[M+H]^+$ 397.1911, Found 397.1915. **HPLC** (IA-3, hexane/*i*-PrOH = 90:10, detector: 254 nm, flow rate: 1 mL/min): < 1% ee, t_1 = 17.5 min, t_2 = 18.9 min.

3-(4-Methoxyphenyl)-*N*-(quinolin-8-yl)-4-(*p*-tolyl)butanamide (4ba)

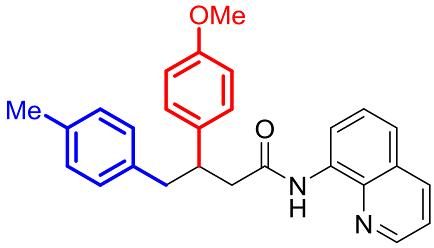

Following the general procedure, the reaction was carried out with **1a** (0.3 mmol, 1.0 equiv), *p*-tolylboronic acid **2b** (0.6 mmol, 2.0 equiv), 4-methoxyphenyl iodide **3a** (0.9 mmol, 3.0 equiv), Pd[(-)-sparteine]Cl_2 (0.03 mmol, 10 mol%), (-)-sparteine (0.06 mmol, 20 mol%), Na_2CO_3 (31.8 mg, 0.3 mmol) in the mixture of DCM/H_2O/CH_3CN (2 mL:0.4 mL:0.2 mL) for 24 h at 100 ℃ under a N_2 atmosphere. 3-(4-Methoxyphenyl)-*N*-(quinolin-8-yl)-4-(*p*-tolyl)butanamide (**4ba**) was isolated by column chromatography (PE/acetone = 11/1) as a white wax in 76 % yield. **^{1}H NMR (400 MHz, $CDCl_3$)**: δ 9.89 (s, 1H), 8.99 – 8.92 (m, 2H), 8.31 (dd, *J* = 8.3, 1.6 Hz, 1H), 7.74 – 7.58 (m, 3H), 7.40 (t, *J* = 5.8 Hz, 2H), 7.28 – 7.20 (m, 4H), 7.07 – 7.00 (m, 2H), 3.94 (s, 3H), 3.88 – 3.78 (m, 1H), 3.28 – 3.18 (m, 2H), 3.09 (ddd, *J* = 23.2, 14.8, 7.3 Hz, 2H), 2.49 (s, 3H). **^{13}C NMR (100 MHz, $CDCl_3$):** δ 170.30, 158.15, 147.99, 138.26, 136.63, 136.26, 135.77, 135.48, 134.47, 129.26, 128.90, 128.61, 127.87, 127.36, 121.53, 121.34, 116.41, 113.88, 55.14, 44.32, 43.45, 42.71, 21.05. **HRMS (ESI)** m/z Calcd. for $C_{27}H_{26}N_2O_2$ $[M+H]^+$ 411.2067, Found 411.2070.

4-(4-(*tert*-Butyl)phenyl)-3-(4-methoxyphenyl)-*N*-(quinolin-8-yl)butanamide (4ca)

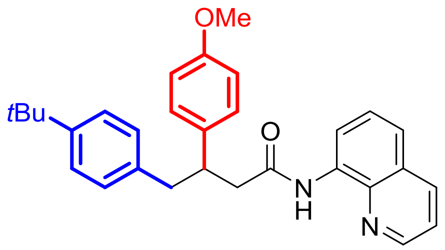

Following the general procedure, the reaction was carried out with **1a** (0.3 mmol, 1.0 equiv), (4-(*tert*-butyl)phenyl)boronic acid **2c** (0.6 mmol, 2.0 equiv), 4-methoxyphenyl iodide **3a** (0.9 mmol, 3.0 equiv), Pd[(-)-sparteine]Cl_2 (0.03 mmol, 10 mol%), (-)-sparteine (0.06 mmol, 20 mol%), Na_2CO_3 (31.8 mg, 0.3 mmol) in the mixture of DCM/H_2O/CH_3CN (2 mL:0.4 mL:0.2 mL) for 24 h at 100 ℃ under a N_2 atmosphere. 4-(4-(*tert*-Butyl)phenyl)-3-(4-methoxyphenyl)-*N*-(quinolin-8-yl)butane-mide (**4ca**) was isolated by column chromatography (PE/acetone = 14/1) as a white solid in 69% yield. **^{1}H NMR (400 MHz, $CDCl_3$)**: δ 9.63 (s, 1H), 8.74 (dd, *J* = 4.1, 1.4 Hz, 1H), 8.70 (d, *J* = 7.3 Hz, 1H), 8.08 (d, *J* = 8.1 Hz, 1H), 7.49 – 7.41 (m, 2H), 7.39 (dd, *J* = 8.3, 4.2 Hz, 1H), 7.24 (t, *J* = 9.1 Hz, 4H), 7.09 (d, *J* = 8.1 Hz, 2H), 6.81 (d, *J* = 8.5 Hz,

2H), 3.72 (s, 3H), 3.67 – 3.60 (m, 1H), 3.04 – 2.78 (m, 4H), 1.28 (s, 9H). **^{13}C NMR (101 MHz, $CDCl_3$)**: δ 170.35, 158.20, 148.89, 148.00, 138.29, 136.68, 136.29, 136.10, 134.50, 129.05, 128.60, 127.90, 127.40, 125.18, 121.55, 121.33, 116.43, 113.95, 55.18, 44.27, 43.29, 42.74, 34.39, 31.44. **HRMS (ESI)** m/z Calcd. for $C_{30}H_{32}N_2O_2$ $[M+H]^+$ 453.2537, Found 453.2541.

4-(4-(Benzyloxy)phenyl)-3-(4-methoxyphenyl)-*N*-(quinolin-8-yl)butanamide (4da)

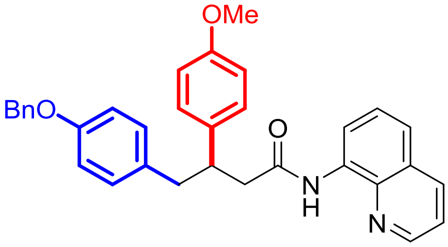

Following the general procedure, the reaction was carried out with **1a** (0.2 mmol, 1.0 equiv), (4-fluorophenyl)boronic acid **2d** (0.4 mmol, 2.0 equiv), 4-methoxyphenyl iodide **3a** (0.6 mmol, 3.0 equiv), Pd[(-)-sparteine]Cl_2 (0.02 mmol, 10 mol%), (-)-sparteine (0.04 mmol, 20 mol%), Na_2CO_3 (21.2 mg, 0.2 mmol) in the mixture of DCM/H_2O/CH_3CN (2 mL:0.4 mL:0.2 mL) for 24 h at 100 ℃ under a N_2 atmosphere. 4-(4-(Benzyloxy)phenyl)-3-(4-methoxyphenyl)-*N*-(quinolin-8-yl)butanamide (**4da**) was isolated by column chromatography (PE/acetone = 8/1) as a white solid in 75% yield. **^{1}H NMR (400 MHz, $CDCl_3$)**: δ 9.67 (s, 1H), 8.76 (dd, *J* = 4.2, 1.6 Hz, 1H), 8.72 (dd, *J* = 7.3, 1.6 Hz, 1H), 8.11 (dd, *J* = 8.3, 1.6 Hz, 1H), 7.51 – 7.36 (m, 7H), 7.35 – 7.29 (m, 1H), 7.17 (d, *J* = 8.7 Hz, 2H), 7.01 (d, *J* = 8.6 Hz, 2H), 6.81 (dd, *J* = 12.8, 8.7 Hz, 4H), 4.98 (s, 2H), 3.73 (s, 3H), 3.63 – 3.52 (m, 1H), 3.04 – 2.80 (m, 4H). **^{13}C NMR (101 MHz, $CDCl_3$)**: δ 170.39, 158.19, 157.26, 148.07, 138.33, 137.27, 136.36, 135.76, 134.51, 132.18, 130.40, 128.68, 128.62, 127.97, 127.94, 127.59, 127.45, 121.61, 121.43, 116.49, 114.62, 113.93, 70.04, 55.22, 44.31, 43.64, 42.34. **HRMS (ESI)** m/z Calcd. for $C_{26}H_{23}FN_2O_2$ $[M+H]^+$ 503.2329, Found 503.2333.

4-(4-Fluorophenyl)-3-(4-methoxyphenyl)-*N*-(quinolin-8-yl)butanamide (4ea)

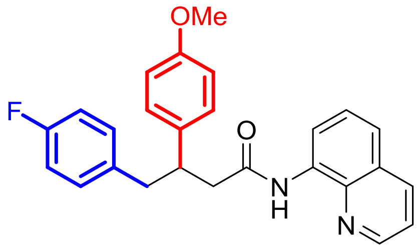

Following the general procedure, the reaction was carried out with **1a** (0.3 mmol, 1.0 equiv), (4-fluorophenyl)boronic acid **2e** (0.6 mmol, 2.0 equiv), 4-methoxyphenyl iodide **3a** (0.9 mmol, 3.0 equiv), Pd[(-)-sparteine]Cl_2 (0.03 mmol, 10 mol%), (-)-sparteine (0.06 mmol, 20 mol%), Na_2CO_3 (31.8 mg, 0.3 mmol) in the mixture of DCM/H_2O/CH_3CN (2 mL:0.4 mL:0.2 mL) for 24 h at 100 ℃ under a N_2 atmosphere. 4-(4-Fluorophenyl)-3-(4-methoxyphenyl)-*N*-(quinolin-8-yl)butanamide (**4ea**) was isolated by column chromatography (PE/acetone = 14/1) as a white solid in 61% yield. **^{1}H NMR (400 MHz, $CDCl_3$)**: δ 9.69 (s, 1H), 8.75 (dd, *J* = 4.2, 1.5 Hz, 1H), 8.72 (dd, *J* = 7.2, 1.5 Hz, 1H), 8.11 (d, *J* = 8.2 Hz, 1H), 7.52 – 7.44 (m, 2H), 7.42 (dd, *J* = 8.3, 4.2 Hz, 1H), 7.12 (d, *J* = 8.6 Hz, 2H), 7.01 (dd, *J* = 8.5, 5.5 Hz, 2H), 6.87 (t, *J* = 8.7 Hz, 2H), 6.79 (t, *J* = 5.7 Hz, 2H), 3.72 (s, 3H), 3.60 – 3.51 (m, 1H), 3.05 (dd, *J* = 13.5, 6.3 Hz, 1H), 2.92 (dd, *J* = 9.3, 4.2 Hz, 1H), 2.90 – 2.84 (m, 2H). **^{13}C NMR (101 MHz, $CDCl_3$)**: δ 170.17, 161.47 (d, *J* = 243.6 Hz), 158.30, 148.10, 138.34, 136.38, 135.47 (d, *J* = 3.2 Hz), 135.28, 134.46, 130.78 (d, *J* = 7.9 Hz), 128.67, 127.96, 127.44, 121.57 (d, *J* = 12.3 Hz), 116.52, 114.05, 114.85, 113.97, 55.21, 44.38, 43.64, 42.23. **^{19}F NMR (376 MHz, $CDCl_3$)**: δ -117.04. **HRMS (ESI)** m/z Calcd. for $C_{26}H_{23}FN_2O_2$ $[M+H]^+$ 415.1816, Found 415.1820.

4-(4-Bromophenyl)-3-(4-methoxyphenyl)-*N*-(quinolin-8-yl)butanamide (4fa)

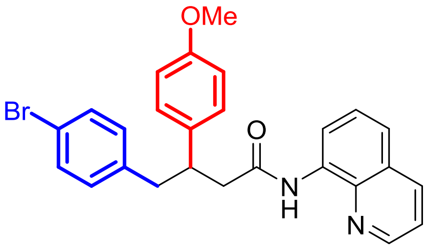

Following the general procedure, the reaction was carried out with **1a** (0.3 mmol, 1.0

equiv), (4-bromophenyl)boronic acid **2f** (0.6 mmol, 2.0 equiv), 4-methoxyphenyl iodide **3a** (0.9 mmol, 3.0 equiv), Pd[(-)-sparteine]Cl_2 (0.03 mmol, 10 mol%), (-)-sparteine (0.06 mmol, 20 mol%), Na_2CO_3 (31.8 mg, 0.3 mmol) in the mixture of DCM/H_2O/CH_3CN (2 mL:0.4 mL:0.2 mL) for 24 h at 100 ℃ under a N_2 atmosphere. 4-(4-Bromophenyl)-3-(4-methoxyphenyl)-*N*-(quinolin-8-yl)butanamide **(4fa)** was isolated by column chromatography (PE/acetone = 14/1) as a white solid in 70% yield. **^{1}H NMR (400 MHz, CDCl$_3$)**: δ 9.69 (s, 1H), 8.75 (dd, *J* = 4.2, 1.6 Hz, 1H), 8.72 (dd, *J* = 7.3, 1.4 Hz, 1H), 8.11 (dd, *J* = 8.3, 1.4 Hz, 1H), 7.52 – 7.44 (m, 2H), 7.41 (dd, *J* = 8.3, 4.2 Hz, 1H), 7.30 (d, *J* = 8.3 Hz, 2H), 7.11 (d, *J* = 8.6 Hz, 2H), 6.92 (d, *J* = 8.3 Hz, 2H), 6.78 (d, *J* = 8.6 Hz, 2H), 3.72 (s, 3H), 3.62 – 3.52 (m, 1H), 3.04 (dd, *J* = 13.5, 6.1 Hz, 1H), 2.94 – 2.83 (m, 3H). **^{13}C NMR (101 MHz, CDCl$_3$)**: δ 170.02, 158.33, 148.08, 138.80, 138.32, 136.34, 135.10, 134.43, 131.23, 131.15, 128.64, 128.56, 127.95, 127.41, 121.61, 121.51, 119.97, 116.52, 114.00, 55.19, 44.41, 43.36, 42.37. **HRMS (ESI)** m/z Calcd. for $C_{26}H_{23}BrN_2O_2$ $[M+H]^+$ 475.1016, Found 475.1020.

3-(4-Methoxyphenyl)-*N*-(quinolin-8-yl)-4-(4-(trifluoromethyl)phenyl)butanamide (4ga)

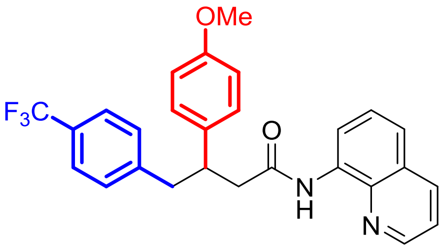

Following the general procedure, the reaction was carried out with **1a** (0.3 mmol, 1.0 equiv), (4-(trifluoromethyl)phenyl)boronic acid **2g** (0.6 mmol, 2.0 equiv), 4-methoxyphenyl iodide **3a** (0.9 mmol, 3.0 equiv), Pd[(-)-sparteine]Cl_2 (0.03 mmol, 10 mol%), (-)-sparteine (0.06 mmol, 20 mol%), Na_2CO_3 (31.8 mg, 0.3 mmol) in the mixture of DCM/H_2O/CH_3CN (2 mL:0.4 mL:0.2 mL) for 24 h at 100 ℃ under an N_2 atmosphere. 3-(4-Methoxyphenyl)-*N*-(quinolin-8-yl)-4-(4-(trifluoromethyl)phenyl)-butanamide (**4ga**) was isolated by column chromatography (PE/acetone = 14/1) as a white solid in 59% yield. **^{1}H NMR (400 MHz, CDCl$_3$)**: δ 9.69 (s, 1H), 8.76 (dd, *J* = 4.2, 1.6 Hz, 1H), 8.71 (dd, *J* = 7.0, 1.9 Hz, 1H), 8.13 (dd, *J* = 8.3, 1.6 Hz, 1H), 7.51 –

7.41 (m, 5H), 7.14 (dd, *J* = 18.6, 8.3 Hz, 4H), 6.79 (d, *J* = 8.7 Hz, 2H), 3.73 (s, 3H), 3.60 (dd, *J* = 14.5, 7.5 Hz, 1H), 3.16 (dd, *J* = 13.4, 6.1 Hz, 1H), 2.99 (dd, *J* = 13.4, 8.7 Hz, 1H), 2.89 (dd, *J* = 7.3, 1.8 Hz, 2H). **^{13}C NMR (100 MHz, $CDCl_3$)**: δ 169.97, 158.45, 148.16, 144.03, 138.39, 136.43, 134.99, 134.44, 129.74, 128.66, 128.45 (*q*, *J* = 32.32 Hz), 128.02, 127.48, 125.13 (*q*, *J* = 3.5 Hz), 124.45 (*q*, *J* = 272.70 Hz), 121.69, 121.61, 116.60, 114.10, 55.26, 44.56, 43.34, 42.78. **^{19}F NMR (376 MHz, $CDCl_3$)**: δ -62.00. **HRMS (ESI)** m/z Calcd. for $C_{27}H_{23}F_3N_2O_2$ $[M+H]^+$ 465.1784, Found 465.1788.

4-(4-Acetylphenyl)-3-(4-methoxyphenyl)-*N*-(quinolin-8-yl)butanamide (4ha) (CAS: 2088104-10-1)[6]

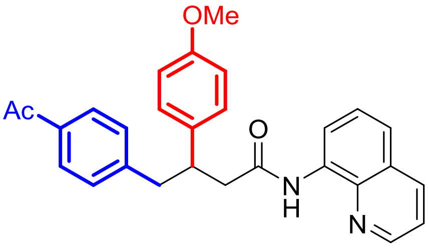

Following the general procedure, the reaction was carried out with **1a** (0.3 mmol, 1.0 equiv), (4-acetylphenyl)boronic acid **2h** (0.6 mmol, 2.0 equiv), 4-methoxyphenyl iodide **3a** (0.9 mmol, 3.0 equiv), Pd[(-)-sparteine]Cl_2 (0.03 mmol, 10 mol%), (-)-sparteine (0.06 mmol, 20 mol%), Na_2CO_3 (31.8 mg, 0.3 mmol) in the mixture of DCM/H_2O/CH_3CN (2 mL:0.4 mL:0.2 mL) for 24 h at 100 ℃ under a N_2 atmosphere. 4-(4-Acetylphenyl)-3-(4-methoxyphenyl)-*N*-(quinolin-8-yl)butanamide (**4ha**) was isolated by column chromatography (PE/acetone = 9/1) as a white solid in 73% yield. **^{1}H NMR (400 MHz, $CDCl_3$)**: δ 9.68 (s, 1H), 8.75 (dd, *J* = 4.2, 1.5 Hz, 1H), 8.70 (dd, *J* = 6.9, 1.9 Hz, 1H), 8.12 (dd, *J* = 8.3, 1.5 Hz, 1H), 7.76 (d, *J* = 8.2 Hz, 2H), 7.53 – 7.44 (m, 2H), 7.42 (dd, *J* = 8.3, 4.2 Hz, 1H), 7.13 (dd, *J* = 13.6, 8.4 Hz, 4H), 6.77 (d, *J* = 8.6 Hz, 2H), 3.71 (s, 3H), 3.67 – 3.57 (m, 1H), 3.15 (dd, *J* = 13.4, 6.1 Hz, 1H), 2.99 (dd, *J* = 13.3, 8.7 Hz, 1H), 2.89 (dd, *J* = 7.3, 2.1 Hz, 2H), 2.51 (s, 3H). **^{13}C NMR (100 MHz, $CDCl_3$)**: δ 197.92, 169.99, 158.38, 148.14, 145.69, 138.34, 136.40, 135.27, 135.05, 134.42, 129.63, 128.62, 128.36, 127.98, 127.43, 121.66, 121.57, 116.55, 114.04, 55.23, 44.61, 43.30, 42.99, 26.58. **HRMS (ESI)** m/z Calcd. for $C_{28}H_{26}N_2O_3$

$[M+H]^+$ 439.2016, Found 439.2020.

Methyl 4-(2-(4-methoxyphenyl)-4-oxo-4-(quinolin-8-ylamino)butyl)benzoate (4ia) (CAS: 2088104-09-8)[6]

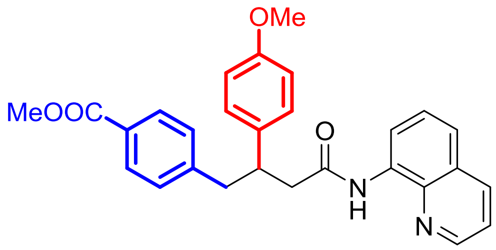

Following the general procedure, the reaction was carried out with **1a** (0.3 mmol, 1.0 equiv), (4-(methoxycarbonyl)phenyl)boronic acid **2i** (0.6 mmol, 2.0 equiv), 4-methoxyphenyl iodide **3a** (1.2 mmol, 4.0 equiv), Pd[(-)-sparteine]Cl_2 (0.03 mmol, 10 mol%), (-)-sparteine (0.06 mmol, 20 mol%), Na_2CO_3 (31.8 mg, 0.3 mmol) in the mixture of DCM/H_2O/CH_3CN (2 mL:0.4 mL:0.2 mL) for 48 h at 100 ℃ under a N_2 atmosphere. Methyl 4-(2-(4-methoxyphenyl)-4-oxo-4-(quinolin-8-ylamino)butyl)benzoate (**4ia**) was isolated by column chromatography (PE/acetone = 9/1) as a white solid in 43% yield. **^{1}H NMR (400 MHz, $CDCl_3$)**: δ 9.69 (s, 1H), 8.75 (dd, *J* = 4.2, 1.6 Hz, 1H), 8.71 (dd, *J* = 7.1, 1.9 Hz, 1H), 8.11 (dd, *J* = 8.3, 1.6 Hz, 1H), 7.85 (d, *J* = 8.3 Hz, 2H), 7.51 – 7.44 (m, 2H), 7.41 (dd, *J* = 8.3, 4.2 Hz, 1H), 7.10 (dd, *J* = 12.0, 5.1 Hz, 4H), 6.80 – 6.72 (m, 2H), 3.86 (s, 3H), 3.71 (s, 3H), 3.65 – 3.57 (m, 1H), 3.15 (dd, *J* = 13.3, 6.1 Hz, 1H), 3.01 – 2.93 (m, 1H), 2.93 – 2.83 (m, 2H). **^{13}C NMR (100 MHz, $CDCl_3$)**: δ 170.04, 167.20, 158.28, 148.12, 145.39, 138.27, 136.39, 134.93, 134.35, 129.52, 129.45, 128.61, 128.00, 127.93, 127.40, 121.65, 121.56, 116.52, 113.95, 55.19, 52.03, 44.49, 43.32, 43.01. **HRMS (ESI)** m/z Calcd. for $C_{28}H_{26}N_2O_4$ $[M+H]^+$ 455.1965, Found 455.1968.

4-(4-Formylphenyl)-3-(4-methoxyphenyl)-*N*-(quinolin-8-yl)butanamide (4ja)

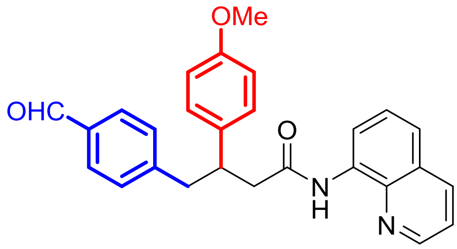

Following the general procedure, the reaction was carried out with **1a** (0.3 mmol, 1.0 equiv), (4-formylphenyl)boronic acid **2j** (0.6 mmol, 2.0 equiv), 4-methoxyphenyl iodide **3a** (1.2 mmol, 4.0 equiv), Pd[(-)-sparteine]Cl_2 (0.03 mmol, 10 mol%), (-)-sparteine (0.06 mmol, 20 mol%), Na_2CO_3 (31.8 mg, 0.3 mmol) in the mixture of DCM/H_2O/CH_3CN (2 mL:0.4 mL:0.2 mL) for 48 h at 100 ℃ under a N_2 atmosphere. 4-(4-Formylphenyl)-3-(4-methoxyphenyl)-*N*-(quinolin-8-yl)butanamide (**4ja**) was isolated by column chromatography (PE/acetone = 9/1) as a light yellow solid in 57% yield. **^{1}H NMR (400 MHz, $CDCl_3$)**: δ 9.88 (s, 1H), 9.70 (s, 1H), 8.74 (dd, *J* = 4.2, 1.6 Hz, 1H), 8.71 (dd, *J* = 7.0, 2.0 Hz, 1H), 8.10 (dd, *J* = 8.3, 1.5 Hz, 1H), 7.67 (d, *J* = 8.1 Hz, 2H), 7.51 – 7.42 (m, 2H), 7.40 (dd, *J* = 8.3, 4.2 Hz, 1H), 7.20 (d, *J* = 8.0 Hz, 2H), 7.12 – 7.06 (m, 2H), 6.79 – 6.72 (m, 2H), 3.69 (s, 3H), 3.62 (dd, *J* = 8.5, 6.4 Hz, 1H), 3.18 (dd, *J* = 13.3, 6.0 Hz, 1H), 2.99 (dd, *J* = 13.3, 8.9 Hz, 1H), 2.89 (dd, *J* = 7.3, 1.4 Hz, 2H). **^{13}C NMR (100 MHz, $CDCl_3$)**: δ 192.05, 169.90, 158.33, 148.10, 147.33, 138.25, 136.37, 134.78, 134.59, 134.31, 130.03, 129.69, 128.56, 127.91, 127.35, 121.64, 121.59, 116.50, 113.98, 55.16, 44.52, 43.26, 43.09. **HRMS (ESI)** m/z Calcd. for $C_{27}H_{24}N_2O_3$ $[M+H]^+$ 425.1860, Found 425.1864.

3-(4-Methoxyphenyl)-*N*-(quinolin-8-yl)-4-(*m*-tolyl)butanamide (4ka)

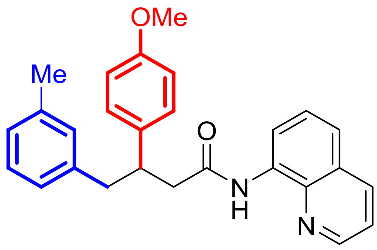

Following the general procedure, the reaction was carried out with **1a** (0.3 mmol, 1.0 equiv), *m*-tolylboronic acid **2k** (0.6 mmol, 2.0 equiv), 4-methoxyphenyl iodide **3a** (0.9

mmol, 3.0 equiv), Pd[(-)-sparteine]Cl_2 (0.03 mmol, 10 mol%), (-)-sparteine (0.06 mmol, 20 mol%), Na_2CO_3 (31.8 mg, 0.3 mmol) in the mixture of DCM/H_2O/CH_3CN (2 mL:0.4 mL:0.2 mL) for 24 h at 100 ℃ under a N_2 atmosphere. 3-(4-Methoxyphenyl)-*N*-(quinolin-8-yl)-4-(*m*-tolyl)butanamide **(4ka)** was isolated by column chromatography (PE/acetone = 11/1) as a white solid in 69% yield. **^{1}H NMR (400 MHz, $CDCl_3$)**: δ 9.67 (s, 1H), 8.75 (dd, *J* = 4.2, 1.5 Hz, 1H), 8.72 (dd, *J* = 7.3, 1.3 Hz, 1H), 8.10 (dd, *J* = 8.3, 1.4 Hz, 1H), 7.52 – 7.43 (m, 2H), 7.41 (dd, *J* = 8.3, 4.2 Hz, 1H), 7.19 (d, *J* = 8.6 Hz, 2H), 7.11 (dd, *J* = 9.6, 5.9 Hz, 1H), 6.97 (s, 2H), 6.91 (d, *J* = 7.5 Hz, 1H), 6.81 (d, *J* = 8.6 Hz, 2H), 3.72 (s, 3H), 3.63 (dd, *J* = 14.6, 7.3 Hz, 1H), 3.03 – 2.80 (m, 4H), 2.28 (s, 3H). **^{13}C NMR (100 MHz, $CDCl_3$)**: δ 170.34, 158.23, 148.04, 139.72, 138.34, 137.75, 136.32, 135.86, 134.52, 130.23, 128.64, 128.11, 127.93, 127.42, 126.89, 126.48, 121.57, 121.39, 116.48, 113.94, 55.21, 44.28, 43.42, 43.16, 21.44. **HRMS (ESI)** m/z Calcd. for $C_{27}H_{26}N_2O_2$ $[M+H]^+$ 411.2067, Found 411.2072.

4-(3-Methoxyphenyl)-3-phenyl-*N*-(quinolin-8-yl)butanamide (4lb)

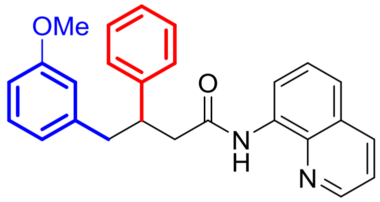

Following the general procedure, the reaction was carried out with **1a** (0.3 mmol, 1.0 equiv), (3-methoxyphenyl)boronic acid **2l** (0.6 mmol, 2.0 equiv), iodobenzene **3b** (1.2 mmol, 4.0 equiv), Pd[(-)-sparteine]Cl_2 (0.03 mmol, 10 mol%), (-)-sparteine (0.06 mmol, 20 mol%), Na_2CO_3 (31.8 mg, 0.3 mmol) in the mixture of DCM/H_2O/CH_3CN (2 mL:0.4 mL:0.2 mL) for 24 h at 100 ℃ under a N_2 atmosphere. 4-(3-Methoxyphenyl)-3-phenyl-*N*-(quinolin-8-yl)butanamide **(4lb)** was isolated by column chromatography (PE/acetone = 11/1) as a white wax in 72% yield. **^{1}H NMR (400 MHz, $CDCl_3$)**: δ 9.72 (s, 1H), 8.77 (dd, *J* = 4.2, 1.6 Hz, 1H), 8.74 (dd, *J* = 7.3, 1.1 Hz, 1H), 8.11 (d, *J* = 8.2 Hz, 1H), 7.53 – 7.44 (m, 2H), 7.41 (dd, *J* = 8.2, 4.2 Hz, 1H), 7.28 (dd, *J* = 4.7, 3.0 Hz, 4H), 7.20 – 7.13 (m, 2H), 6.78 – 6.69 (m, 2H), 6.65 (s, 1H), 3.75 – 3.66 (m, 4H), 3.12 – 2.88 (m, 4H). **^{13}C NMR (100 MHz, $CDCl_3$)**: δ

170.12, 159.44, 148.05, 143.60, 141.20, 138.25, 136.31, 134.40, 129.17, 128.52, 127.87, 127.73, 127.36, 126.60, 121.82, 121.57, 121.43, 116.44, 114.83, 111.82, 55.09, 44.05, 43.95, 43.03. **HRMS (ESI)** m/z Calcd. for $C_{26}H_{24}N_2O_2$ $[M+H]^+$ 397.1911, Found 397.1914.

4-(3-Fluorophenyl)-3-(4-methoxyphenyl)-*N*-(quinolin-8-yl)butanamide (4ma)

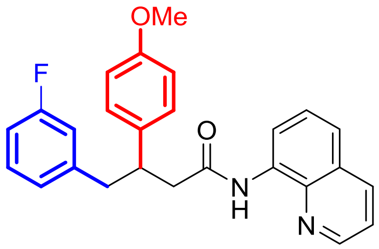

Following the general procedure, the reaction was carried out with **1a** (0.3 mmol, 1.0 equiv), (3-fluorophenyl)boronic acid **2m** (0.6 mmol, 2.0 equiv), 4-methoxyphenyl iodide **3a** (0.9 mmol, 3.0 equiv), Pd[(-)sparteine]Cl_2 (0.03 mmol, 10 mol%), (-)sparteine (0.06 mmol, 20 mol%), Na_2CO_3 (31.8 mg, 0.3 mmol) in the mixture of DCM/H_2O/CH_3CN (2 mL:0.4 mL:0.2 mL) for 24 h at 100 ℃ under a N_2 atmosphere. 4-(3-Fluorophenyl)-3-(4-methoxyphenyl)-*N*-(quinolin-8-yl)butanamide **(4ma)** was isolated by column chromatography (PE/acetone = 10/1) as a white solid in 66% yield. **^{1}H NMR (400 MHz, CDCl$_3$)**: δ 9.70 (s, 1H), 8.75 (d, *J* = 4.1 Hz, 1H), 8.72 (d, *J* = 7.2 Hz, 1H), 8.11 (d, *J* = 8.2 Hz, 1H), 7.52 – 7.44 (m, 2H), 7.41 (dd, *J* = 8.2, 4.2 Hz, 1H), 7.19 – 7.10 (m, 3H), 6.90 – 6.73 (m, 5H), 3.71 (s, 3H), 3.65 – 3.55 (m, 1H), 3.12 – 2.93 (m, 2H), 2.89 (dd, *J* = 14.6, 7.8 Hz, 2H). **^{13}C NMR (101 MHz, CDCl$_3$)**: δ 170.07, 162.75 (d, *J* = 245.2 Hz), 158.30, 148.08, 142.42 (d, *J* = 7.2 Hz), 138.26, 136.40, 135.14, 134.39, 129.58 (d, *J* = 8.3 Hz), 128.59, 127.93, 127.42, 125.08 (d, *J* = 2.5 Hz), 121.58 (d, *J* = 10.7 Hz), 116.53, 116.19 (d, *J* = 20.9 Hz), 113.98, 113.11, 112.90. 55.19, 44.40, 43.33, 42.72. **^{19}F NMR (376 MHz, CDCl$_3$)**: δ -113.40. **HRMS (ESI)** m/z Calcd. for $C_{26}H_{23}FN_2O_2$ $[M+H]^+$ 415.1816, Found 415.1820.

4-(3-Chlorophenyl)-3-(4-methoxyphenyl)-*N*-(quinolin-8-yl)butanamide (4na)

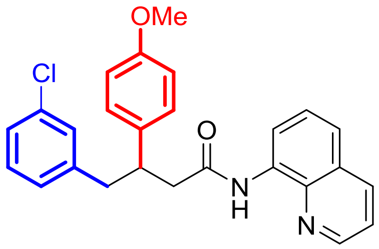

Following the general procedure, the reaction was carried out with **1a** (0.3 mmol, 1.0 equiv), (3-chlorophenyl)boronic acid **2n** (0.6 mmol, 2.0 equiv), 4-methoxyphenyl iodide **3a** (0.9 mmol, 3.0 equiv), Pd[(-)-sparteine]Cl_2 (0.03 mmol, 10 mol%), (-)-sparteine (0.06 mmol, 20 mol%), Na_2CO_3 (31.8 mg, 0.3 mmol) in the mixture of DCM/H_2O/CH_3CN (2 mL:0.4 mL:0.2 mL) for 24 h at 100 ℃ under a N_2 atmosphere. 4-(3-Chlorophenyl)-3-(4-methoxyphenyl)-*N*-(quinolin-8-yl)butanamide **(4na)** was isolated by column chromatography (PE/acetone = 10/1) as a white solid in 63% yield. **^{1}H NMR (400 MHz, $CDCl_3$)**: δ 9.70 (s, 1H), 8.76 (dd, *J* = 4.2, 1.6 Hz, 1H), 8.72 (dd, *J* = 7.1, 1.7 Hz, 1H), 8.11 (dd, *J* = 8.3, 1.6 Hz, 1H), 7.53 – 7.39 (m, 3H), 7.12 (dd, *J* = 10.1, 6.5 Hz, 5H), 6.97 – 6.91 (m, 1H), 6.79 (d, *J* = 8.7 Hz, 2H), 3.72 (s, 3H), 3.63 – 3.53 (m, 1H), 3.05 (dd, *J* = 13.5, 6.5 Hz, 1H), 2.95 – 2.90 (m, 1H), 2.90 – 2.81 (m, 2H). **^{13}C NMR (100 MHz, $CDCl_3$)**: δ 170.03, 158.30, 148.12, 141.88, 138.28, 136.37, 135.06, 134.37, 133.92, 129.45, 129.44, 128.59, 127.92, 127.60, 127.41, 126.33, 121.64, 121.54, 116.50, 113.98, 55.22, 44.33, 43.31, 42.66. **HRMS (ESI)** m/z Calcd. for $C_{26}H_{23}ClN_2O_2$ $[M+H]^+$ 431.1521, Found 431.1525.

4-(3-Cyanophenyl)-3-(4-methoxyphenyl)-*N*-(quinolin-8-yl)butanamide (4oa)

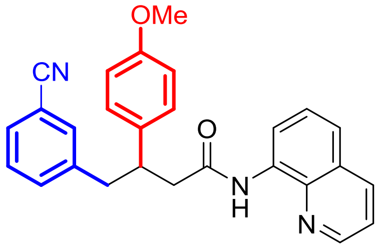

Following the general procedure, the reaction was carried out with **1a** (0.3 mmol, 1.0 equiv), (3-cyanophenyl)boronic acid **2o** (0.6 mmol, 2.0 equiv), 4-methoxyphenyl iodide **3a** (1.2 mmol, 4.0 equiv), Pd[(-)-sparteine]Cl_2 (0.03 mmol, 10 mol%),

(-)-sparteine (0.06 mmol, 20 mol%), Na_2CO_3 (31.8 mg, 0.3 mmol) in the mixture of DCM/H_2O/CH_3CN (2 mL:0.4 mL:0.2 mL) for 24 h at 100 ℃ under a N_2 atmosphere. 4-(3-cyanophenyl)-3-(4-methoxyphenyl)-*N*-(quinolin-8-yl)butanamide **(4oa)** was isolated by column chromatography (PE/acetone = 9/1) as a white solid in 68% yield. **^{1}H NMR (400 MHz, CDCl$_3$)**: δ 9.71 (s, 1H), 8.75 (dd, *J* = 4.2, 1.5 Hz, 1H), 8.70 (dd, *J* = 6.8, 1.9 Hz, 1H), 8.11 (dd, *J* = 8.3, 1.4 Hz, 1H), 7.51 – 7.44 (m, 2H), 7.41 (dd, *J* = 8.3, 4.2 Hz, 1H), 7.39 – 7.34 (m, 1H), 7.31 (s, 1H), 7.28 – 7.21 (m, 2H), 7.06 (d, *J* = 8.6 Hz, 2H), 6.76 (d, *J* = 8.6 Hz, 2H), 3.70 (s, 3H), 3.60 – 3.51 (m, 1H), 3.13 (dd, *J* = 13.5, 5.7 Hz, 1H), 2.96 – 2.83 (m, 3H). **^{13}C NMR (100 MHz, CDCl$_3$):** δ 169.75, 158.43, 148.18, 141.31, 138.25, 136.37, 134.42, 134.28, 133.90, 132.80, 129.89, 128.89, 128.55, 127.92, 127.35, 121.67, 121.63, 118.99, 116.50, 114.07, 112.08, 55.19, 44.40, 43.30, 42.35. **HRMS (ESI)** m/z Calcd. for $C_{27}H_{23}N_3O_2$ $[M+H]^+$ 422.1863, Found 422.1865.

3-(4-Methoxyphenyl)-*N*-(quinolin-8-yl)-4-(*o*-tolyl)butanamide (4pa)

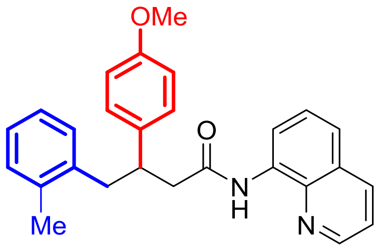

Following the general procedure, the reaction was carried out with **1a** (0.3 mmol, 1.0 equiv), 1-iodo-2-methylbenzene **2p** (0.6 mmol, 2.0 equiv), 4-methoxyphenyl iodide **3a** (0.9 mmol, 3.0 equiv), [(-)-sparteine]$PdCl_2$ (0.03 mmol, 10 mol%), (-)-sparteine (0.06 mmol, 20 mol%), Na_2CO_3 (31.8 mg, 0.3 mmol) in the mixture of DCM/H_2O/CH_3CN (2 mL:0.4 mL:0.2 mL) for 24 h at 100 ℃ under a N_2 atmosphere. 3-(4-methoxyphenyl)-*N*-(quinolin-8-yl)-4-(*o*-tolyl)butanamide **(4pa)** was isolated by column chromatography (PE/acetone = 9/1) as a white wax in 40% yield. **^{1}H NMR (400 MHz, CDCl$_3$)**: δ 9.69 (s, 1H), 8.76 (dd, *J* = 4.2, 1.6 Hz, 1H), 8.71 (dd, *J* = 7.1, 1.7 Hz, 1H), 8.11 (dd, *J* = 8.3, 1.6 Hz, 1H), 7.51 – 7.44 (m, 2H), 7.42 (dd, *J* = 8.3, 4.2 Hz, 1H), 7.17 (d, *J* = 8.6 Hz, 2H), 7.12 – 7.00 (m, 4H), 6.80 (t, *J* = 5.8 Hz, 2H), 3.72 (s, 3H), 3.64 – 3.54 (m, 1H), 3.07 (dd, *J* = 13.7, 7.1 Hz, 1H), 3.00 – 2.85 (m, 3H),

2.27 (s, 3H). **^{13}C NMR (100 MHz, CDCl$_3$)**: δ 170.31, 158.27, 148.07, 138.34, 138.09, 136.56, 136.36, 135.95, 134.51, 130.33, 130.28, 128.56, 127.94, 127.45, 126.28, 125.72, 121.60, 121.42, 116.51, 113.96, 55.23, 44.33, 42.37, 40.69, 19.58. **HRMS (ESI)** m/z Calcd. for $C_{27}H_{26}N_2O_2$ $[M+H]^+$ 411.2067, Found 411.2071.

3-(4-Methoxyphenyl)-4-(naphthalen-2-yl)-*N*-(quinolin-8-yl)butanamide (4qa)

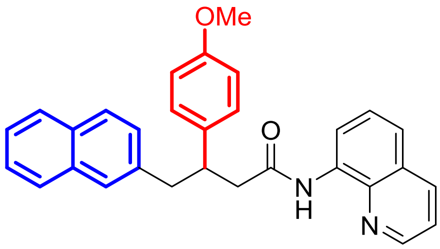

Following the general procedure, the reaction was carried out with **1a** (0.3 mmol, 1.0 equiv), naphthalen-2-ylboronic acid **2q** (0.6 mmol, 2.0 equiv), 4-methoxyphenyl iodide **3a** (0.9 mmol, 3.0 equiv), [(-)-sparteine]$PdCl_2$ (0.03 mmol, 10 mol%), (-)-sparteine (0.06 mmol, 20 mol%), Na_2CO_3 (31.8 mg, 0.3 mmol) in the mixture of DCM/H_2O/CH_3CN (2 mL:0.4 mL:0.2 mL) for 24 h at 100 ℃ under a N_2 atmosphere. 3-(4-Methoxyphenyl)-4-(naphthalen-2-yl)-*N*-(quinolin-8-yl)butanamide **(4qa)** was isolated by column chromatography (PE/acetone = 11/1) as a white solid in 74% yield. **^{1}H NMR (400 MHz, CDCl$_3$)**: δ 9.68 (s, 1H), 8.78 – 8.68 (m, 2H), 8.21 (d, *J* = 8.4 Hz, 1H), 8.11 (dd, *J* = 8.3, 1.6 Hz, 1H), 7.82 (d, *J* = 7.5 Hz, 1H), 7.67 (d, *J* = 8.2 Hz, 1H), 7.54 – 7.44 (m, 4H), 7.41 (dd, *J* = 8.3, 4.2 Hz, 1H), 7.29 – 7.23 (m, 1H), 7.15 (d, *J* = 8.6 Hz, 2H), 7.10 (d, *J* = 6.7 Hz, 1H), 6.77 (d, *J* = 8.7 Hz, 2H), 3.79 (dd, *J* = 14.7, 7.4 Hz, 1H), 3.71 (s, 3H), 3.59 (dd, *J* = 13.7, 7.0 Hz, 1H), 3.33 (dd, *J* = 13.7, 7.8 Hz, 1H), 3.03 – 2.91 (m, 2H). **^{13}C NMR (100 MHz, CDCl$_3$)**: δ 170.32, 158.25, 148.08, 138.31, 136.37, 136.01, 135.89, 134.48, 133.97, 132.22, 128.81, 128.54, 127.93, 127.70, 127.46, 127.03, 126.08, 125.51, 125.28, 124.17, 121.61, 121.45, 116.51, 113.96, 55.24, 44.65, 42.47, 40.54. **HRMS (ESI)** m/z Calcd. for $C_{30}H_{26}N_2O_2$ $[M+H]^+$ 447.2067, Found 447.2072.

4-(3,5-Dimethylphenyl)-3-(4-methoxyphenyl)-*N*-(quinolin-8-yl)butanamide (4ra)

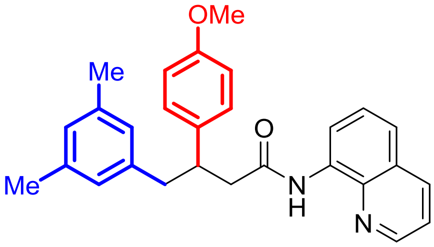

Following the general procedure, the reaction was carried out with **1a** (0.3 mmol, 1.0 equiv), (3,5-dimethylphenyl)boronic acid **2r** (0.6 mmol, 2.0 equiv), 4-methoxyphenyl iodide **3a** (0.9 mmol, 3.0 equiv), [(-)-sparteine]$PdCl_2$ (0.03 mmol, 10 mol%), (-)-sparteine (0.06 mmol, 20 mol%), Na_2CO_3 (31.8 mg, 0.3 mmol) in the mixture of DCM/H_2O/CH_3CN (2 mL:0.4 mL:0.2 mL) for 24 h at 100 ℃ under a N_2 atmosphere. 4-(3,5-Dimethylphenyl)-3-(4-methoxyphenyl)-*N*-(quinolin-8-yl)butanamide **(4ra)** was isolated by column chromatography (PE/acetone = 11/1) as a white solid in 68% yield. **^{1}H NMR (400 MHz, $CDCl_3$)**: δ 9.66 (s, 1H), 8.75 (dd, *J* = 4.2, 1.7 Hz, 1H), 8.72 (dd, *J* = 7.3, 1.6 Hz, 1H), 8.10 (dd, *J* = 8.3, 1.6 Hz, 1H), 7.46 (dt, *J* = 8.3, 7.4 Hz, 2H), 7.41 (dd, *J* = 8.3, 4.2 Hz, 1H), 7.25 – 7.19 (m, 2H), 6.84 – 6.76 (m, 5H), 3.73 (s, 3H), 3.68 – 3.57 (m, 1H), 2.97 – 2.78 (m, 4H), 2.25 (s, 6H). **^{13}C NMR (100 MHz, $CDCl_3$)**: δ 170.41, 158.15, 148.01, 139.61, 138.25, 137.62, 136.31, 136.03, 134.46, 128.58, 127.87, 127.79, 127.38, 127.27, 121.55, 121.36, 116.40, 113.88, 55.19, 44.08, 43.29, 43.14, 21.33. **HRMS (ESI)** m/z Calcd. for $C_{28}H_{28}N_2O_2$ $[M+H]^+$ 425.2225, Found 425.2228.

4-(3,4-Dimethoxyphenyl)-3-(4-methoxyphenyl)-*N*-(quinolin-8-yl)butanamide(4sa)

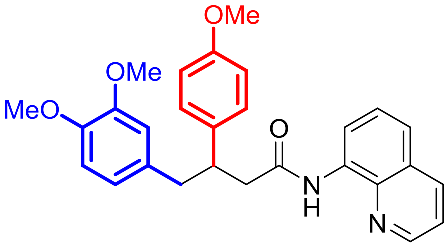

Following the general procedure, the reaction was carried out with **1a** (0.3 mmol, 1.0 equiv), (3,4-dimethoxyphenyl)boronic acid **2s** (0.6 mmol, 2.0 equiv), 4-methoxyphenyl iodide **3a** (0.9 mmol, 3.0 equiv), Pd[(-)-sparteine]Cl_2 (0.03 mmol,

10 mol%), (-)-sparteine (0.06 mmol, 20 mol%), Na_2CO_3 (31.8 mg, 0.3 mmol) in the mixture of DCM/H_2O/CH_3CN (2 mL:0.4 mL:0.2 mL) for 24 h at 100 ℃ under a N_2 atmosphere. 4-(3,4-Dimethoxyphenyl)-3-(4-methoxyphenyl)-*N*-(quinolin-8-yl)butanamide (**4sa**) was isolated by column chromatography (PE/acetone = 11/1) as a white solid in 81% yield. **^{1}H NMR (400 MHz, $CDCl_3$)**: δ 9.67 (s, 1H), 8.70 (dd, *J* = 4.7, 2.7 Hz, 2H), 8.10 – 8.01 (m, 1H), 7.48 – 7.39 (m, 2H), 7.37 (ddd, *J* = 7.1, 4.1, 3.0 Hz, 1H), 7.13 (d, *J* = 8.6 Hz, 2H), 6.77 (d, *J* = 8.6 Hz, 2H), 6.64 (dt, *J* = 8.2, 5.0 Hz, 2H), 6.50 (d, *J* = 1.8 Hz, 1H), 3.77 (s, 3H), 3.73 (s, 3H), 3.68 (s, 3H), 3.56 (dd, *J* = 14.6, 7.1 Hz, 1H), 2.99 (dd, *J* = 13.5, 6.6 Hz, 1H), 2.95 – 2.80 (m, 3H). **^{13}C NMR (100 MHz, $CDCl_3$)**: δ 170.24, 158.07, 148.33, 147.93, 147.15, 138.11, 136.21, 135.51, 134.30, 132.13, 128.61, 127.76, 127.24, 121.48, 121.35, 121.30, 116.29, 113.75, 112.41, 110.74, 55.69, 55.60, 55.07, 44.11, 43.44, 42.62. **HRMS (ESI)** m/z Calcd. for $C_{28}H_{28}N_2O_4$ $[M+H]^+$ 457.2122, Found 457.2125.

4-(1*H*-Indol-5-yl)-3-(4-methoxyphenyl)-*N*-(quinolin-8-yl)butanamide (4ta)

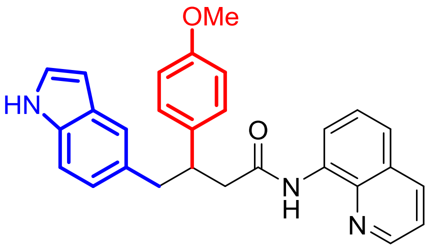

Following the general procedure, the reaction was carried out with **1a** (0.3 mmol, 1.0 equiv), (1*H*-indol-5-yl)boronic acid **2t** (0.6 mmol, 2.0 equiv), 4-methoxyphenyl iodide **3a** (1.2 mmol, 4.0 equiv), Pd[(-)-sparteine]Cl_2 (0.03 mmol, 10 mol%), (-)-sparteine (0.06 mmol, 20 mol%), Na_2CO_3 (31.8 mg, 0.3 mmol) in the mixture of DCM/H_2O/CH_3CN (2 mL:0.4 mL:0.2 mL) for 48 h at 100 ℃ under a N_2 atmosphere. 4-(1*H*-Indol-5-yl)-3-(4-methoxyphenyl)-*N*-(quinolin-8-yl)butanamide **(4ta)** was isolated by column chromatography (PE/acetone = 6/1) as a light yellow solid in 30% yield. **^{1}H NMR (400 MHz, $CDCl_3$)**: δ 9.66 (s, 1H), 8.74 (dd, *J* = 4.2, 1.7 Hz, 1H), 8.70 (dd, *J* = 7.0, 1.9 Hz, 1H), 8.27 (s, 1H), 8.10 (dd, *J* = 8.3, 1.6 Hz, 1H), 7.49 – 7.38 (m, 4H), 7.25 – 7.18 (m, 3H), 7.15 – 7.10 (m, 1H), 6.95 (dd, *J* = 8.3, 1.5 Hz, 1H), 6.82 – 6.75 (m, 2H), 6.45 (dd, *J* = 2.6, 1.7 Hz, 1H), 3.76 – 3.62 (m, 4H), 3.10 (dd, *J* = 7.4,

3.1 Hz, 2H), 2.95 (dd, *J* = 14.8, 6.0 Hz, 1H), 2.83 (dd, *J* = 14.9, 8.9 Hz, 1H). **^{13}C NMR (100 MHz, $CDCl_3$)**: δ 170.74, 158.08, 148.06, 138.30, 136.33, 136.22, 134.64, 134.49, 130.98, 128.68, 127.99, 127.91, 127.41, 124.36, 123.77, 121.58, 121.38, 121.12, 116.46, 113.90, 110.87, 102.28, 55.21, 44.17, 44.01, 43.47. **HRMS (ESI)** m/z Calcd. for $C_{28}H_{25}N_3O_2$ $[M+H]^+$ 436.2020, Found. 436.2023.

3-(4-Methoxyphenyl)-*N*-(quinolin-8-yl)-4-(thiophen-3-yl)butanamide (4ua)

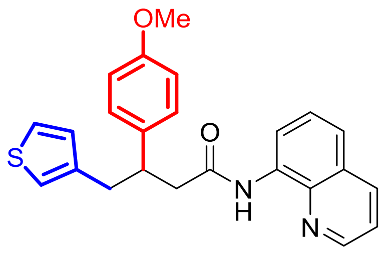

Following the general procedure, the reaction was carried out with **1a** (0.3 mmol, 1.0 equiv), thiophen-3-ylboronic acid **2u** (0.6 mmol, 2.0 equiv), 4-methoxyphenyl iodide **3a** (1.2 mmol, 4.0 equiv), Pd[(-)-sparteine]Cl_2 (0.03 mmol, 10 mol%), (-)-sparteine (0.06 mmol, 20 mol%), Na_2CO_3 (31.8 mg, 0.3 mmol) in the mixture of DCM/H_2O/CH_3CN (2 mL:0.4 mL:0.2 mL) for 48 h at 100 ℃ under a N_2 atmosphere. 3-(4-Methoxyphenyl)-*N*-(quinolin-8-yl)-4-(thiophen-3-yl)butanamide **(4ua)** was isolated by column chromatography (PE/acetone = 9/1) as a white wax in 49% yield. **^{1}H NMR (400 MHz, $CDCl_3$)**: δ 9.68 (s, 1H), 8.75 (dd, *J* = 4.2, 1.6 Hz, 1H), 8.72 (dd, *J* = 7.2, 1.6 Hz, 1H), 8.11 (dd, *J* = 8.3, 1.4 Hz, 1H), 7.52 – 7.44 (m, 2H), 7.41 (dd, *J* = 8.3, 4.2 Hz, 1H), 7.21 – 7.14 (m, 3H), 6.87 – 6.78 (m, 4H), 3.73 (s, 3H), 3.61 (t, *J* = 7.3 Hz, 1H), 3.06 (ddd, *J* = 22.0, 14.1, 7.3 Hz, 2H), 2.87 (ddd, *J* = 22.9, 14.8, 7.4 Hz, 2H). **^{13}C NMR (100 MHz, $CDCl_3$)**: δ 170.26, 158.25, 148.08, 140.01, 138.31, 136.36, 135.70, 134.46, 128.84, 128.58, 127.93, 127.42, 125.09, 121.84, 121.62, 121.47, 116.49, 113.95, 55.21, 44.46, 42.77, 37.31. **HRMS (ESI)** m/z Calcd. for $C_{24}H_{22}N_2O_2S$ $[M+H]^+$ 403.1475, Found 403.1478.

4-Phenyl-*N*-(quinolin-8-yl)-3-(*p*-tolyl)butanamide (4ac)

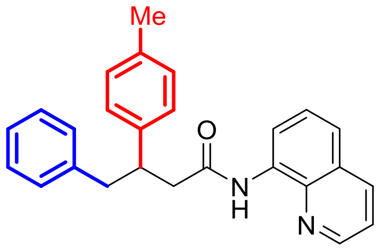

Following the general procedure, the reaction was carried out with **1a** (0.3 mmol, 1.0 equiv), phenylboronic acid **2a** (0.6 mmol, 2.0 equiv), 1-iodo-4-methylbenzene **3c** (0.9 mmol, 3.0 equiv), Pd[(-)-sparteine]Cl_2 (0.03 mmol, 10 mol%), (-)-sparteine (0.06 mmol, 20 mol%), Na_2CO_3 (31.8 mg, 0.3 mmol) in the mixture of DCM/H_2O/CH_3CN (2 mL:0.4 mL:0.2 mL) for 24 h at 100 ℃ under a N_2 atmosphere. 4-Phenyl-*N*-(quinolin-8-yl)-3-(*p*-tolyl)butanamide (**4ac**) was isolated by column chromatography (PE/acetone = 11/1) as a white wax in 87 % yield. **^{1}H NMR (400 MHz, CDCl$_3$):** δ 9.53 (s, 1H), 8.62 – 8.53 (m, 2H), 7.93 (d, *J* = 8.1 Hz, 1H), 7.30 (dt, *J* = 8.2, 7.5 Hz, 2H), 7.23 (dd, *J* = 8.2, 4.2 Hz, 1H), 7.12 – 6.93 (m, 7H), 6.90 (d, *J* = 7.8 Hz, 2H), 3.48 (dd, *J* = 14.4, 7.2 Hz, 1H), 2.95 – 2.81 (m, 2H), 2.73 (qd, *J* = 14.9, 7.3 Hz, 2H), 2.10 (s, 3H). **^{13}C NMR (100 MHz, CDCl$_3$):** δ 170.25, 148.02, 140.56, 139.80, 138.26, 136.31, 135.97, 134.46, 129.41, 129.23, 128.94, 128.23, 127.88, 127.54, 127.39, 126.11, 121.56, 121.39, 116.45, 44.09, 43.76, 43.04, 21.11. **HRMS (ESI)** m/z Calcd. for $C_{26}H_{24}N_2O$ $[M+H]^+$ 381.1961, Found 381.1965.

3-(4-Fluorophenyl)-4-phenyl-*N*-(quinolin-8-yl)butanamide (4ad)

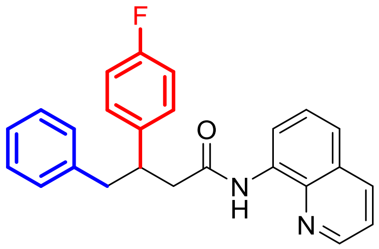

Following the general procedure, the reaction was carried out with **1a** (0.3 mmol, 1.0 equiv), phenylboronic acid **2a** (0.6 mmol, 2.0 equiv), 1-fluoro-4-iodobenzene **3d** (0.9 mmol, 3.0 equiv), Pd[(-)-sparteine]Cl_2 (0.03 mmol, 10 mol%), (-)-sparteine (0.06 mmol, 20 mol%), Na_2CO_3 (31.8 mg, 0.3 mmol) in the mixture of DCM/H_2O/CH_3CN

(2 mL:0.4 mL:0.2 mL) for 24 h at 100 ℃ under a N_2 atmosphere. 3-(4-Fluorophenyl)-4-phenyl-*N*-(quinolin-8-yl)butanamide (**4ad**) was isolated by column chromatography (PE/acetone = 14/1) as a white wax in 67 % yield. **^{1}H NMR (400 MHz, $CDCl_3$)**: δ 9.68 (s, 1H), 8.76 (dd, *J* = 4.2, 1.6 Hz, 1H), 8.71 (dd, *J* = 7.1, 1.8 Hz, 1H), 8.11 (dd, *J* = 8.3, 1.6 Hz, 1H), 7.52 – 7.45 (m, 2H), 7.42 (dd, *J* = 8.3, 4.2 Hz, 1H), 7.26 – 7.12 (m, 5H), 7.09 (d, *J* = 7.0 Hz, 2H), 6.97 – 6.89 (m, 2H), 3.71 – 3.62 (m, 1H), 3.09 (dd, *J* = 13.5, 6.7 Hz, 1H), 3.00 – 2.91 (m, 2H), 2.85 (dd, *J* = 14.9, 8.4 Hz, 1H). **^{13}C NMR (100 MHz, $CDCl_3$)**: δ 169.93, 161.61 (d, *J* = 244.1 Hz), 148.11, 139.43, 139.20 (d, *J* = 3.2 Hz), 138.30, 136.39, 134.37, 129.38, 129.21, 129.13, 128.31, 127.95, 127.41, 126.26, 121.60 (d, *J* = 9.1 Hz), 116.53, 115.32 (d, *J* = 21.1 Hz), 44.20, 43.58, 43.08. **^{19}F NMR (376 MHz, $CDCl_3$)**: δ -116.57. **HRMS (ESI)** m/z Calcd. for $C_{25}H_{21}FN_2O$ $[M+H]^+$ 385.1711, Found 385.1713.

3-(4-Bromophenyl)-4-phenyl-*N*-(quinolin-8-yl)butanamide (4ae)

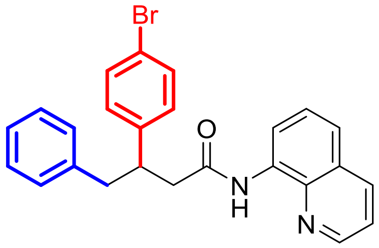

Following the general procedure, the reaction was carried out with **1a** (0.3 mmol, 1.0 equiv), phenylboronic acid **2a** (0.6 mmol, 2.0 equiv), 1-bromo-4-iodobenzene **3e** (0.9 mmol, 3.0 equiv), Pd[(-)-sparteine]Cl_2 (0.03 mmol, 10 mol%), (-)-sparteine (0.06 mmol, 20 mol%), Na_2CO_3 (31.8 mg, 0.3 mmol) in the mixture of DCM/H_2O/CH_3CN (2 mL:0.4 mL:0.2 mL) for 24 h at 100 ℃ under a N_2 atmosphere. 3-(4-Bromophenyl)-4-phenyl-*N*-(quinolin-8-yl)butanamide (**4ae**) was isolated by column chromatography (PE/acetone = 14/1) as a white solid in 68 % yield. **^{1}H NMR (400 MHz, $CDCl_3$)**: δ 9.66 (s, 1H), 8.76 (dd, *J* = 4.2, 1.6 Hz, 1H), 8.68 (dd, *J* = 6.5, 2.4 Hz, 1H), 8.13 (dd, *J* = 8.3, 1.5 Hz, 1H), 7.52 – 7.46 (m, 2H), 7.44 (dd, *J* = 8.3, 4.2 Hz, 1H), 7.35 (d, *J* = 8.4 Hz, 2H), 7.22 (t, *J* = 7.2 Hz, 2H), 7.11 (ddd, *J* = 12.0, 11.4, 7.0 Hz, 5H), 3.67 – 3.58 (m, 1H), 3.06 (dd, *J* = 13.5, 6.8 Hz, 1H), 2.99 – 2.89 (m, 2H), 2.84 (dd, *J* = 15.0, 8.4 Hz, 1H). **^{13}C NMR (100 MHz, $CDCl_3$)**: δ 169.78, 148.20,

142.64, 139.28, 138.34, 136.44, 134.37, 131.67, 129.57, 129.40, 128.40, 128.00, 127.46, 126.37, 121.71, 121.63, 120.42, 116.58, 43.96, 43.79, 42.86. **HRMS (ESI)** m/z Calcd. for $C_{25}H_{21}BrN_2O$ $[M+H]^+$ 445.0910, Found 445.0913.

3-(4-Iodophenyl)-4-phenyl-*N*-(quinolin-8-yl)butanamide (4af)

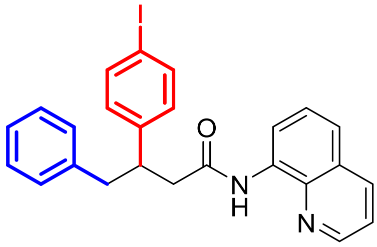

Following the general procedure, the reaction was carried out with **1a** (0.3 mmol, 1.0 equiv), phenylboronic acid **2a** (0.6 mmol, 2.0 equiv), 1,4-diiodobenzene **3f** (0.9 mmol, 3.0 equiv), Pd[(-)-sparteine]Cl_2 (0.03 mmol, 10 mol%), (-)-sparteine (0.06 mmol, 20 mol%), Na_2CO_3 (31.8 mg, 0.3 mmol) in the mixture of DCM/H_2O/CH_3CN (2 mL:0.4 mL:0.2 mL) for 24 h at 100 ℃ under a N_2 atmosphere. 3-(4-Iodophenyl)-4-phenyl-*N*-(quinolin-8-yl)butanamide (**4af**) was isolated by column chromatography (PE/acetone = 14/1) as a white solid in 67 % yield. **^{1}H NMR (400 MHz, $CDCl_3$)**: δ 9.66 (s, 1H), 8.77 (dd, *J* = 4.2, 1.7 Hz, 1H), 8.68 (dd, *J* = 6.5, 2.5 Hz, 1H), 8.13 (dd, *J* = 8.3, 1.6 Hz, 1H), 7.59 – 7.41 (m, 5H), 7.25 – 7.13 (m, 3H), 7.12 – 7.05 (m, 2H), 6.99 (d, *J* = 8.3 Hz, 2H), 3.61 (t, *J* = 6.9 Hz, 1H), 3.06 (dd, *J* = 13.5, 6.8 Hz, 1H), 2.98 – 2.79 (m, 3H). **^{13}C NMR (100 MHz, $CDCl_3$)**: δ 169.78, 148.21, 143.32, 139.25, 138.28, 137.60, 136.44, 134.32, 129.86, 129.38, 128.39, 127.96, 127.44, 126.36, 121.71, 121.62, 116.54, 91.96, 43.85, 43.83, 42.79. **HRMS (ESI)** m/z Calcd. for $C_{25}H_{21}IN_2O$ $[M+H]^+$ 493.0771, Found 493.0775.

3,3'-(1,4-Phenylene)bis(4-phenyl-N-(quinolin-8-yl)butanamide) (4af′)

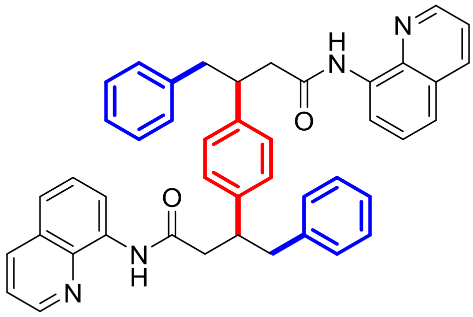

^{1}H NMR (400 MHz, $CDCl_3$): δ 9.63 (s, 2H), 8.75 (dt, *J* = 4.2, 1.5 Hz, 2H), 8.67 (d, *J*

= 7.1 Hz, 2H), 8.09 (dt, *J* = 8.3, 1.5 Hz, 2H), 7.51 – 7.36 (m, 6H), 7.09 – 6.95 (m, 10H), 6.94 – 6.86 (m, 4H), 3.57 – 3.47 (m, 2H), 3.01 – 2.94 (m, 2H), 2.88 – 2.73 (m, 6H). **HRMS (ESI)** m/z Calcd. for $C_{44}H_{38}N_4O_2$ $[M+H]^+$ 655.3068, Found. 655.3070.

4-Phenyl-*N*-(quinolin-8-yl)-3-(4-(trifluoromethyl)phenyl)butanamide (4ag)

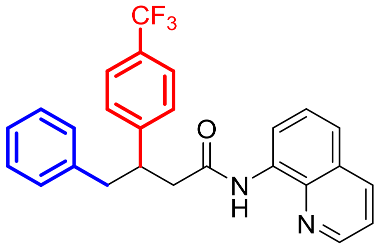

Following the general procedure, the reaction was carried out with **1a** (0.3 mmol, 1.0 equiv), phenylboronic acid **2a** (0.6 mmol, 2.0 equiv), 1-iodo-4-(trifluoromethyl)benzene **3g** (0.9 mmol, 3.0 equiv), Pd[(-)-sparteine]Cl_2 (0.03 mmol, 10 mol%), (-)-sparteine (0.06 mmol, 20 mol%), Na_2CO_3 (31.8 mg, 0.3 mmol) in the mixture of DCM/H_2O/CH_3CN (2 mL:0.4 mL:0.2 mL) for 24 h at 100 ℃ under a N_2 atmosphere. 4-Phenyl-*N*-(quinolin-8-yl)-3-(4-(trifluoromethyl)phenyl)-butanamide (**4ag**) was isolated by column chromatography (PE/acetone = 14/1) as a white wax in 54 % yield. **^{1}H NMR (400 MHz, $CDCl_3$)**: δ 9.68 (s, 1H), 8.75 (dd, *J* = 4.2, 1.6 Hz, 1H), 8.70 (dd, *J* = 6.7, 2.0 Hz, 1H), 8.12 (dd, *J* = 8.3, 1.5 Hz, 1H), 7.48 (dt, *J* = 8.6, 5.3 Hz, 4H), 7.43 (dd, *J* = 8.3, 4.2 Hz, 1H), 7.36 (d, *J* = 8.1 Hz, 2H), 7.26 – 7.14 (m, 3H), 7.10 (d, *J* = 7.0 Hz, 2H), 3.75 (dd, *J* = 14.6, 7.3 Hz, 1H), 3.14 – 2.86 (m, 4H). **^{13}C NMR (100 MHz, $CDCl_3$):** δ 169.59, 148.17, 147.81, 139.05, 138.27, 136.43, 134.27, 129.34, 128.85 (*q*, *J* = 272.70 Hz), 128.43, 128.13, 127.96, 127.41, 126.45, 125.52 (q, *J* = 3.7 Hz), 124.32 (q, *J* = 272.70 Hz), 121.69 (d, *J* = 3.0 Hz). 116.55, 44.05, 43.63, 42.72. **^{19}F NMR (376 MHz, $CDCl_3$)**: δ -62.03. **HRMS (ESI)** m/z Calcd. for $C_{26}H_{21}F_3N_2O$ $[M+H]^+$ 435.1679, Found 435.1682.

3-(4-Acetylphenyl)-4-phenyl-*N*-(quinolin-8-yl)butanamide (4ah)

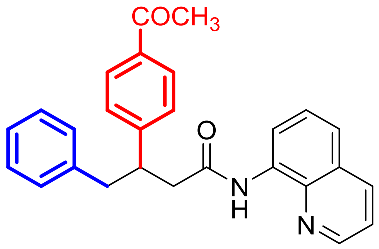

Following the general procedure, the reaction was carried out with **1a** (0.3 mmol, 1.0 equiv), phenylboronic acid **2a** (0.6 mmol, 2.0 equiv), 1-(4-iodophenyl)ethan-1-one **3h** (0.9 mmol, 3.0 equiv), Pd[(-)-sparteine]Cl_2 (0.03 mmol, 10 mol%), (-)-sparteine (0.06 mmol, 20 mol%), Na_2CO_3 (31.8 mg, 0.3 mmol) in the mixture of DCM/H_2O/CH_3CN (2 mL:0.4 mL:0.2 mL) for 24 h at 100 ℃ under a N_2 atmosphere. 3-(4-Acetylphenyl)-4-phenyl-*N*-(quinolin-8-yl)butanamide (**4ah**) was isolated by column chromatography (PE/acetone = 9/1) as a white solid in 70 % yield. **^{1}H NMR (400 MHz, $CDCl_3$):** δ 9.59 (s, 1H), 8.64 (d, *J* = 3.0 Hz, 1H), 8.61 – 8.54 (m, 1H), 8.01 (d, *J* = 8.1 Hz, 1H), 7.73 (d, *J* = 8.1 Hz, 2H), 7.41 – 7.34 (m, 2H), 7.32 (dd, *J* = 8.1, 4.1 Hz, 1H), 7.23 (d, *J* = 8.1 Hz, 2H), 7.14 – 7.03 (m, 3H), 6.98 (d, *J* = 7.1 Hz, 2H), 3.71 – 3.58 (m, 1H), 3.01 (dd, *J* = 13.4, 6.7 Hz, 1H), 2.96 – 2.76 (m, 3H), 2.41 (s, 3H). **^{13}C NMR (100 MHz, $CDCl_3$):** δ 197.89, 169.61, 149.31, 148.13, 139.09, 138.24, 136.40, 135.64, 134.26, 129.31, 128.70, 128.36, 128.00, 127.91, 127.38, 126.37, 121.67, 121.61, 116.51, 44.23, 43.64, 42.63, 26.60. **HRMS (ESI)** m/z Calcd. for $C_{27}H_{24}N_2O_2$ $[M+H]^+$ 409.1911, Found 409.1913.

Ethyl 4-(4-oxo-1-phenyl-4-(quinolin-8-ylamino)butan-2-yl)benzoate (4ai)

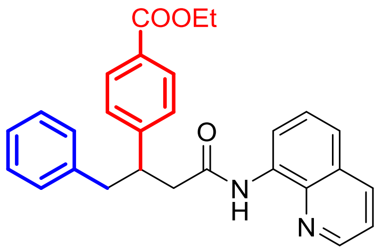

Following the general procedure, the reaction was carried out with **1a** (0.3 mmol, 1.0 equiv), phenylboronic acid **2a** (0.6 mmol, 2.0 equiv), ethyl 4-iodobenzoate **3i** (0.9 mmol, 3.0 equiv), Pd[(-)-sparteine]Cl_2 (0.03 mmol, 10 mol%), (-)-sparteine (0.06

mmol, 20 mol%), Na_2CO_3 (31.8 mg, 0.3 mmol) in the mixture of DCM/H_2O/CH_3CN (2 mL:0.4 mL:0.2 mL) for 24 h at 100 ℃ under a N_2 atmosphere. Ethyl 4-(4-oxo-1-phenyl-4-(quinolin-8-ylamino)butan-2-yl)benzoate (**4ai**) was isolated by column chromatography (PE/acetone = 9/1) as a white solid in 63 % yield. **^{1}H NMR (400 MHz, $CDCl_3$)**: δ 9.69 (s, 1H), 8.74 (dd, *J* = 4.2, 1.7 Hz, 1H), 8.68 (dd, *J* = 6.7, 2.2 Hz, 1H), 8.11 (dd, *J* = 8.3, 1.6 Hz, 1H), 7.92 (d, *J* = 8.3 Hz, 2H), 7.51 – 7.44 (m, 2H), 7.41 (dd, *J* = 8.3, 4.2 Hz, 1H), 7.30 (d, *J* = 8.3 Hz, 2H), 7.23 – 7.11 (m, 3H), 7.10 – 7.04 (m, 2H), 4.32 (q, *J* = 7.1 Hz, 2H), 3.80 – 3.67 (m, 1H), 3.11 (dd, *J* = 13.5, 6.7 Hz, 1H), 3.04 – 2.85 (m, 3H), 1.35 (t, *J* = 7.1 Hz, 3H). **^{13}C NMR (100 MHz, $CDCl_3$)**: δ 169.69, 166.62, 148.91, 148.13, 139.14, 138.27, 136.40, 134.29, 129.87, 129.34, 128.88, 128.35, 127.93, 127.81, 127.40, 126.34, 121.65, 121.60, 116.54, 60.87, 44.27, 43.75, 42.71, 14.42. **HRMS (ESI)** m/z Calcd. for $C_{28}H_{26}N_2O_3$ $[M+H]^+$ 439.2016, Found 439.2020.

4-Phenyl-*N*-(quinolin-8-yl)-3-(*m*-tolyl)butanamide (4aj)

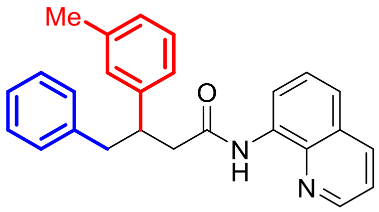

Following the general procedure, the reaction was carried out with **1a** (0.3 mmol, 1.0 equiv), phenylboronic acid **2a** (0.6 mmol, 2.0 equiv), 1-iodo-3-methylbenzene **3j** (0.9 mmol, 3.0 equiv), Pd[(-)-sparteine]Cl_2 (0.03 mmol, 10 mol%), (-)-sparteine (0.06 mmol, 20 mol%), Na_2CO_3 (31.8 mg, 0.3 mmol) in the mixture of DCM/H_2O/CH_3CN (2 mL:0.4 mL:0.2 mL) for 24 h at 100 ℃ under a N_2 atmosphere. 4-Phenyl-*N*-(quinolin-8-yl)-3-(*m*-tolyl)butanamide (**4aj**) was isolated by column chromatography (PE/acetone = 11/1) as a white wax in 72 % yield. **^{1}H NMR (400 MHz, $CDCl_3$)**: δ 9.67 (s, 1H), 8.76 (dd, *J* = 4.2, 1.6 Hz, 1H), 8.71 (dd, *J* = 7.2, 1.6 Hz, 1H), 8.11 (dd, *J* = 8.3, 1.6 Hz, 1H), 7.47 (dt, *J* = 8.3, 7.4 Hz, 2H), 7.42 (dd, *J* = 8.2, 4.3 Hz, 1H), 7.22 (t, *J* = 7.2 Hz, 2H), 7.18 – 7.11 (m, 4H), 7.10 – 7.04 (m, 2H), 6.96 (d, *J* = 7.4 Hz, 1H), 3.63 (p, *J* = 7.4 Hz, 1H), 3.11 – 2.98 (m, 2H), 2.97 – 2.84 (m, 2H), 2.28 (s, 3H). **^{13}C NMR (100 MHz, $CDCl_3$)**: δ 170.30, 148.07, 143.63, 139.81, 138.37,

138.03, 136.37, 134.51, 129.46, 128.52, 128.42, 128.26, 127.95, 127.45, 127.40, 126.17, 124.76, 121.61, 121.43, 116.52, 44.18, 44.01, 43.07, 21.56. **HRMS (ESI)** m/z Calcd. for $C_{26}H_{24}N_2O$ $[M+H]^+$ 381.1961, Found 381.1965.

3-(3-Methoxyphenyl)-4-phenyl-*N*-(quinolin-8-yl)butanamide (4ak)

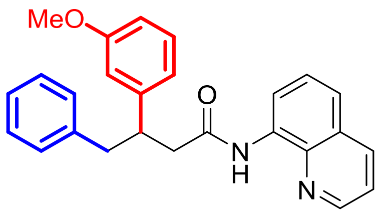

Following the general procedure, the reaction was carried out with **1a** (0.3 mmol, 1.0 equiv), phenylboronic acid **2a** (0.6 mmol, 2.0 equiv), 1-iodo-3-methoxybenzene **3k** (0.9 mmol, 3.0 equiv), Pd[(-)-sparteine]Cl_2 (0.03 mmol, 10 mol%), (-)-sparteine (0.06 mmol, 20 mol%), Na_2CO_3 (31.8 mg, 0.3 mmol) in the mixture of DCM/H_2O/CH_3CN (2 mL:0.4 mL:0.2 mL) for 24 h at 100 ℃ under a N_2 atmosphere. 3-(3-Methoxyphenyl)-4-phenyl-*N*-(quinolin-8-yl)butanamide (**4ak**) was isolated by column chromatography (PE/acetone = 11/1) as a white wax in 69 % yield. **^{1}H NMR (400 MHz, CDCl$_3$)**: δ 9.71 (s, 1H), 8.78 – 8.68 (m, 2H), 8.09 (d, *J* = 8.3 Hz, 1H), 7.51 – 7.42 (m, 2H), 7.40 (ddd, *J* = 8.2, 4.2, 1.2 Hz, 1H), 7.22 (dd, *J* = 13.8, 6.6 Hz, 2H), 7.15 (dd, *J* = 13.4, 7.7 Hz, 4H), 6.87 (d, *J* = 7.5 Hz, 1H), 6.81 (d, *J* = 1.5 Hz, 1H), 6.70 (dd, *J* = 8.2, 2.4 Hz, 1H), 3.74 – 3.62 (m, 4H), 3.05 (qd, *J* = 13.5, 7.5 Hz, 2H), 2.97 – 2.86 (m, 2H). **^{13}C NMR (100 MHz, CDCl$_3$)**: δ 170.11, 159.64, 148.04, 145.24, 139.63, 138.25, 136.30, 134.41, 129.48, 129.38, 128.23, 127.87, 127.36, 126.16, 121.57, 121.43, 120.04, 116.44, 113.46, 112.01, 55.14, 44.26, 43.95, 42.91. **HRMS (ESI)** m/z Calcd. for $C_{26}H_{24}N_2O_2$ $[M+H]^+$ 397.1911, Found 397.1913.

3-(3-Bromophenyl)-4-phenyl-*N*-(quinolin-8-yl)butanamide (4al)

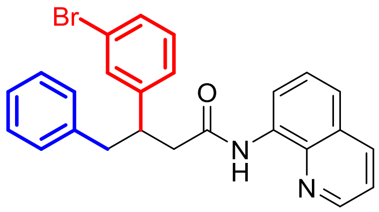

Following the general procedure, the reaction was carried out with **1a** (0.3 mmol, 1.0 equiv), phenylboronic acid **2a** (0.6 mmol, 2.0 equiv), 1-bromo-3-iodobenzene **3l** (0.9

mmol, 3.0 equiv), Pd[(-)-sparteine]Cl_2 (0.03 mmol, 10 mol%), (-)-sparteine (0.06 mmol, 20 mol%), Na_2CO_3 (31.8 mg, 0.3 mmol) in the mixture of DCM/H_2O/CH_3CN (2 mL:0.4 mL:0.2 mL) for 24 h at 100 ℃ under a N_2 atmosphere. 3-(3-Bromophenyl)-4-phenyl-*N*-(quinolin-8-yl)butanamide (**4al**) was isolated by column chromatography (PE/acetone = 11/1) as a light yellow wax in 60 % yield. **^{1}H NMR (400 MHz, CDCl$_3$)**: δ 9.69 (s, 1H), 8.80 (dd, *J* = 4.2, 1.6 Hz, 1H), 8.71 (dd, *J* = 6.8, 2.1 Hz, 1H), 8.14 (dd, *J* = 8.3, 1.6 Hz, 1H), 7.52 – 7.43 (m, 4H), 7.27 (ddd, *J* = 21.9, 8.7, 4.4 Hz, 3H), 7.20 – 7.06 (m, 5H), 3.70 – 3.60 (m, 1H), 3.08 (dd, *J* = 13.5, 7.0 Hz, 1H), 3.03 – 2.84 (m, 3H). **^{13}C NMR (100 MHz, CDCl$_3$)**: δ 169.71, 148.20, 146.08, 139.16, 138.30, 136.41, 134.31, 130.61, 130.11, 129.80, 129.39, 128.39, 127.94, 127.42, 126.76, 126.40, 122.68, 121.69, 121.61, 116.54, 43.96, 43.71, 42.83. **HRMS (ESI)** m/z Calcd. for $C_{25}H_{21}BrN_2O$ $[M+H]^+$ 445.0910, Found 445.0913.

3-(2-Methoxyphenyl)-*N*-(naphthalen-1-yl)-4-phenylbutanamide (4am)

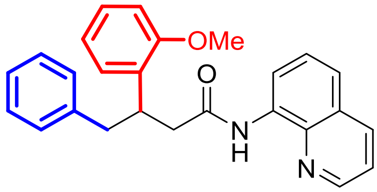

Following the general procedure, the reaction was carried out with **1a** (0.3 mmol, 1.0 equiv), phenylboronic acid **2a** (0.6 mmol, 2.0 equiv), 1-iodo-2-methoxybenzene **3m** (0.9 mmol, 3.0 equiv), Pd[(-)-sparteine]Cl_2 (0.03 mmol, 10 mol%), (-)-sparteine (0.06 mmol, 20 mol%), Na_2CO_3 (31.8 mg, 0.3 mmol) in the mixture of DCM/H_2O/CH_3CN (2 mL:0.4 mL:0.2 mL) for 24 h at 100 ℃ under a N_2 atmosphere. 3-(2-Methoxyphenyl)-*N*-(naphthalen-1-yl)-4-phenylbutanamide **(4am)** was isolated by column chromatography (PE/acetone = 11/1) as a white solid in 55 % yield. **^{1}H NMR (400 MHz, CDCl$_3$)**: δ 9.75 (s, 1H), 8.74 (ddd, *J* = 8.8, 5.7, 1.6 Hz, 2H), 8.11 (dd, *J* = 8.3, 1.6 Hz, 1H), 7.49 – 7.40 (m, 3H), 7.22 – 7.10 (m, 7H), 6.87 – 6.79 (m, 2H), 4.03 (p, *J* = 7.4 Hz, 1H), 3.83 (s, 3H), 3.13 – 2.98 (m, 3H), 2.91 (dd, *J* = 14.7, 6.9 Hz, 1H). **^{13}C NMR (100 MHz, CDCl$_3$)**: δ 170.84, 157.43, 148.00, 140.26, 138.34, 136.34, 134.63, 131.34, 129.44, 128.49, 128.09, 127.91, 127.55, 127.46, 125.95, 121.56, 121.26, 120.60, 116.43, 110.79, 55.43, 42.39, 41.00, 38.92. **HRMS (ESI)** m/z

Calcd. for $C_{26}H_{24}N_2O_2$ $[M+H]^+$ 397.1911, Found 397.1914.

3-(3,4-Dimethylphenyl)-4-phenyl-*N*-(quinolin-8-yl)butanamide (4an)

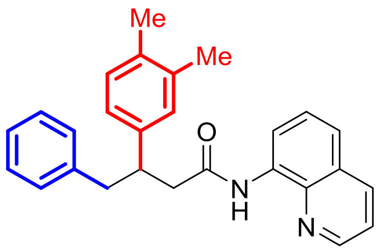

Following the general procedure, the reaction was carried out with **1a** (0.3 mmol, 1.0 equiv), phenylboronic acid **2a** (0.6 mmol, 2.0 equiv), 4-iodo-1,2-dimethylbenzene **3n** (0.9 mmol, 3.0 equiv), Pd[(-)-sparteine]Cl_2 (0.03 mmol, 10 mol%), (-)-sparteine (0.06 mmol, 20 mol%), Na_2CO_3 (31.8 mg, 0.3 mmol) in the mixture of DCM/H_2O/CH_3CN (2 mL:0.4 mL:0.2 mL) for 24 h at 100 ℃ under a N_2 atmosphere. 3-(3,4-Dimethylphenyl)-4-phenyl-*N*-(quinolin-8-yl)butanamide **(4an)** was isolated by column chromatography (PE/acetone = 11/1) as a white solid in 70 % yield. **^{1}H NMR (400 MHz, CDCl$_3$)**: δ 9.68 (s, 1H), 8.76 – 8.71 (m, 2H), 8.07 (dd, *J* = 8.3, 1.6 Hz, 1H), 7.50 – 7.42 (m, 2H), 7.38 (dd, *J* = 8.3, 4.2 Hz, 1H), 7.26 – 7.21 (m, 2H), 7.19 – 7.14 (m, 3H), 7.07 (s, 1H), 7.02 (d, *J* = 0.7 Hz, 2H), 3.69 – 3.58 (m, 1H), 3.05 (d, *J* = 7.4 Hz, 2H), 2.93 – 2.85 (m, 2H), 2.19 (s, 3H), 2.16 (s, 3H). **^{13}C NMR (100 MHz, CDCl$_3$)**: δ 170.34, 147.95, 141.11, 139.91, 138.26, 136.49, 136.26, 134.59, 134.48, 129.75, 129.40, 128.96, 128.22, 127.85, 127.35, 126.09, 124.92, 121.50, 121.32, 116.43, 44.05, 43.72, 43.03, 19.85, 19.38. **HRMS (ESI)** m/z Calcd. for $C_{27}H_{26}N_2O$ $[M+H]^+$ 395.2118, Found 395.2121.

3-(3,5-Dimethoxyphenyl)-4-phenyl-*N*-(quinolin-8-yl)butanamide (4ao)

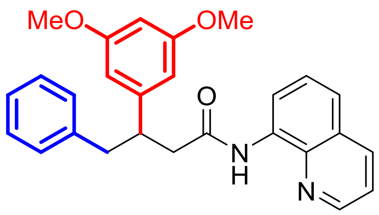

Following the general procedure, the reaction was carried out with **1a** (0.3 mmol, 1.0 equiv), phenylboronic acid **2a** (0.6 mmol, 2.0 equiv), 1-iodo-3,5-dimethoxybenzene **3o** (0.9 mmol, 3.0 equiv), Pd[(-)-sparteine]Cl_2 (0.03 mmol, 10 mol%), (-)-sparteine

(0.06 mmol, 20 mol%), Na_2CO_3 (31.8 mg, 0.3 mmol) in the mixture of DCM/H_2O/CH_3CN (2 mL:0.4 mL:0.2 mL) for 24 h at 100 ℃ under a N_2 atmosphere. 3-(3,5-Dimethoxyphenyl)-4-phenyl-*N*-(quinolin-8-yl)butanamide **(4ao)** was isolated by column chromatography (PE/acetone = 11/1) as a white wax in 73 % yield. **^{1}H NMR (400 MHz, CDCl$_3$):** δ 9.71 (s, 1H), 8.74 (d, *J* = 7.2 Hz, 2H), 8.10 (d, *J* = 8.2 Hz, 1H), 7.46 (dd, *J* = 13.6, 7.9 Hz, 2H), 7.42 – 7.37 (m, 1H), 7.27 – 7.21 (m, 2H), 7.16 (t, *J* = 6.8 Hz, 3H), 6.44 (s, 2H), 6.27 (s, 1H), 3.70 (s, 6H), 3.63 (dd, *J* = 14.5, 7.2 Hz, 1H), 3.12 – 2.98 (m, 2H), 2.90 (d, *J* = 7.2 Hz, 2H). **^{13}C NMR (100 MHz, CDCl$_3$):** δ 170.11, 160.82, 148.02, 146.05, 139.63, 138.30, 136.28, 134.46, 129.39, 128.24, 127.88, 127.34, 126.17, 121.56, 121.42, 116.44, 105.83, 98.70, 55.25, 44.56, 43.95, 42.85. **HRMS (ESI)** m/z Calcd. for $C_{27}H_{26}N_2O_3$ $[M+H]^+$ 427.2016, Found 427.2020.

4-Phenyl-*N*-(quinolin-8-yl)-3-(thiophen-3-yl)butanamide (4ap)

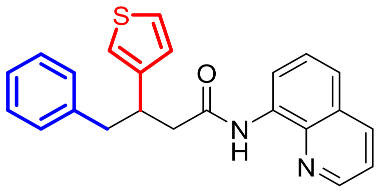

Following the general procedure, the reaction was carried out with **1a** (0.3 mmol, 1.0 equiv), phenylboronic acid **2a** (0.6 mmol, 2.0 equiv), 3-iodothiophene **3p** (1.2mmol, 3.0 equiv), Pd[(-)-sparteine]Cl_2 (0.03 mmol, 10 mol%), (-)-sparteine (0.06 mmol, 20 mol%), Na_2CO_3 (31.8 mg, 0.3 mmol) in the mixture of DCM/H_2O/CH_3CN (2 mL:0.4 mL:0.2 mL) for 36 h at 100 ℃ under a N_2 atmosphere. 4-Phenyl-*N*-(quinolin-8-yl)-3-(thiophen-3-yl)butanamide **(4ap)** was isolated by column chromatography (PE/acetone = 10/1) as a white solid in 48 % yield. **^{1}H NMR (400 MHz, CDCl$_3$)**: δ 9.70 (s, 1H), 8.77 (dd, *J* = 4.2, 1.6 Hz, 1H), 8.73 (dd, *J* = 7.0, 1.9 Hz, 1H), 8.14 (dd, *J* = 8.3, 1.6 Hz, 1H), 7.53 – 7.41 (m, 3H), 7.25 – 7.13 (m, 4H), 7.13 – 7.06 (m, 2H), 7.03 (dd, *J* = 5.0, 1.2 Hz, 1H), 7.00 – 6.93 (m, 1H), 3.87 – 3.72 (m, 1H), 3.11 – 2.78 (m, 4H). **^{13}C NMR (100 MHz, CDCl$_3$)**: δ 170.18, 148.13, 144.36, 139.59, 138.33, 136.40, 134.45, 129.40, 128.28, 127.95, 127.46, 126.92, 126.25, 125.70, 121.66, 121.53, 120.87, 116.53, 43.96, 42.64, 39.49. **HRMS (ESI)** m/z Calcd. for $C_{23}H_{20}N_2OS$ $[M+H]^+$ 373.1369, Found 373.1372.

3-Methyl-4-phenyl-*N*-(quinolin-8-yl)butanamide (4aq) (Cas: 1449299-47-1)[7]

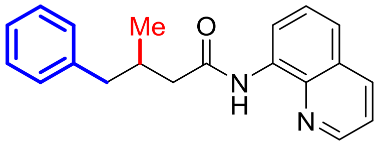

Following the general procedure, the reaction was carried out with **1a** (0.3 mmol, 1.0 equiv), phenylboronic acid **2a** (0.6 mmol, 2.0 equiv), iodomethane **3q** (3 mmol, 10.0 equiv), Pd[(-)-sparteine]Cl_2 (0.03 mmol, 10 mol%), (-)-sparteine (0.06 mmol, 20 mol%), Na_2CO_3 (31.8 mg, 0.3 mmol) in the mixture of DCM/H_2O/CH_3CN (2 mL:0.4 mL:0.2 mL) for 48 h at 100 ℃ under a N_2 atmosphere. 3-Methyl-4-phenyl-*N*-(quinolin-8-yl)butanamide **(4aq)** was isolated by column chromatography (PE/acetone = 14/1) as a white wax in 36 % yield. **^{1}H NMR (400 MHz, $CDCl_3$):** δ 9.56 (s, 1H), 8.63 – 8.49 (m, 2H), 7.93 (dd, *J* = 8.3, 1.6 Hz, 1H), 7.33 – 7.21 (m, 3H), 7.02 (tt, *J* = 8.7, 7.3 Hz, 5H), 2.56 (dd, *J* = 13.2, 6.0 Hz, 1H), 2.42 – 2.26 (m, 3H), 2.15 (dd, *J* = 13.9, 7.7 Hz, 1H), 0.82 (d, *J* = 6.4 Hz, 3H). **^{13}C NMR (100 MHz, $CDCl_3$):** δ 171.16, 148.23, 140.45, 138.48, 136.48, 134.64, 129.44, 128.39, 128.07, 127.56, 126.13, 121.69, 121.50, 116.57, 77.48, 77.16, 76.84, 45.16, 43.22, 32.81, 19.76.

3-(4-Methoxyphenyl)-2-methyl-4-phenyl-*N*-(quinolin-8-yl)butanamide (5a)

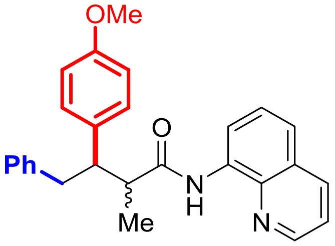

d.r.: 1.6:1

Following the general procedure, the reaction was carried out with **1b** (0.3 mmol, 1.0 equiv), phenylboronic acid **2a** (0.6 mmol, 2.0 equiv), 4-methoxyphenyl iodide **3a** (1.2 mmol, 4.0 equiv), Pd[(-)-sparteine]Cl_2 (0.03 mmol, 10 mol%), (-)-sparteine (0.06 mmol, 20 mol%), Na_2CO_3 (31.8 mg, 0.3 mmol) in the mixture of DCM/H_2O/CH_3CN (2 mL:0.4 mL:0.2 mL) for 36 h at 100 ℃ under a N_2 atmosphere. 3-(4-Methoxyphenyl)-2-methyl-4-phenyl-*N*-(quinolin-8-yl)butanamide **(5a)** was

isolated by column chromatography (PE/acetone = 16/1) as a white solid in 55 % yield. One diastereoisomer: **^{1}H NMR (400 MHz, $CDCl_3$)**: δ 9.58 (s, 1H), 8.75 (dd, *J* = 4.2, 1.6 Hz, 1H), 8.65 (dd, *J* = 6.9, 2.0 Hz, 1H), 8.10 (dd, *J* = 8.3, 1.6 Hz, 1H), 7.49 – 7.38 (m, 3H), 7.18 – 7.01 (m, 7H), 6.65 – 6.58 (m, 2H), 3.58 (s, 3H), 3.34 – 3.21 (m, 2H), 2.98 – 2.85 (m, 2H), 1.50 (d, *J* = 6.9 Hz, 3H). **^{13}C NMR (100 MHz, $CDCl_3$)**: δ 173.86, 158.03, 148.06, 140.31, 138.49, 136.30, 134.52, 134.15, 129.39, 129.37, 128.12, 127.93, 127.45, 125.85, 121.56, 121.32, 116.44, 113.62, 55.07, 50.50, 48.77, 38.87, 16.24. **HRMS (ESI)** m/z Calcd. for $C_{27}H_{26}N_2O_2$ $[M+H]^+$ 411.2067, Found 411.2071. The other diastereoisomer**: ^{1}H NMR (400 MHz, $CDCl_3$)**: δ 9.99 (s, 1H), 8.93 – 8.81 (m, 2H), 8.18 (dd, *J* = 8.2, 1.3 Hz, 1H), 7.56 (dt, *J* = 15.1, 7.6 Hz, 2H), 7.48 (dd, *J* = 8.2, 4.2 Hz, 1H), 7.13 – 6.97 (m, 5H), 6.92 (d, *J* = 6.8 Hz, 2H), 6.77 (d, *J* = 8.6 Hz, 2H), 3.76 (s, 3H), 3.30 – 3.11 (m, 2H), 2.87 (tt, *J* = 13.1, 8.7 Hz, 2H), 1.13 (d, *J* = 6.8 Hz, 3H). **^{13}C NMR (100 MHz, $CDCl_3$)**: δ 174.92, 158.25, 148.32, 140.19, 138.65, 136.54, 134.64, 133.71, 129.63, 129.32, 128.13, 127.95, 127.62, 125.74, 121.76, 121.68, 116.83, 113.75, 55.26, 50.95, 49.12, 41.50, 17.25. **HRMS (ESI)** m/z Calcd. for $C_{27}H_{26}N_2O_2$ $[M+H]^+$ 411.2067, Found 411.2074.

3-(4-Methoxyphenyl)-4-phenyl-*N*-(quinolin-8-yl)pentanamide (5b)

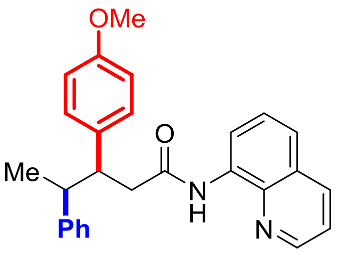

Following the general procedure, the reaction was carried out with **1c** (0.3 mmol, 1.0 equiv), phenylboronic acid **2a** (0.6 mmol, 2.0 equiv), 4-methoxyphenyl iodide **3a** (0.9 mmol, 3.0 equiv), Pd[(-)-sparteine]Cl_2 (0.03 mmol, 10 mol%), (-)-sparteine (0.06 mmol, 20 mol%), Na_2CO_3 (31.8 mg, 0.3 mmol) in the mixture of DCM/H_2O/CH_3CN (2 mL:0.4 mL:0.2 mL) for 48 h at 100 ℃ under a N_2 atmosphere. 3-(4-Methoxyphenyl)-4-phenyl-*N*-(quinolin-8-yl)pentanamide **(5b)** was isolated by column chromatography (PE/acetone = 14/1) as a light yellow wax in 62 % yield. **^{1}H**

NMR (400 MHz, $CDCl_3$): δ 9.67 (s, 1H), 8.76 (dd, *J* = 4.2, 1.6 Hz, 1H), 8.69 (dd, *J* = 6.9, 1.9 Hz, 1H), 8.11 (dd, *J* = 8.3, 1.4 Hz, 1H), 7.50 – 7.45 (m, 2H), 7.42 (dd, *J* = 8.3, 4.3 Hz, 1H), 7.24 – 7.14 (m, 3H), 7.02 (d, *J* = 7.0 Hz, 2H), 6.95 (t, *J* = 6.2 Hz, 2H), 6.70 (d, *J* = 8.6 Hz, 2H), 3.69 (s, 3H), 3.58 (dt, *J* = 8.9, 6.3 Hz, 1H), 3.21 – 3.12 (m, 1H), 3.00 (dd, *J* = 14.9, 6.0 Hz, 1H), 2.82 (dd, *J* = 14.9, 9.1 Hz, 1H), 1.36 (d, *J* = 7.1 Hz, 3H). **^{13}C NMR (100 MHz, $CDCl_3$)**: δ 170.64, 158.08, 148.08, 144.05, 138.36, 136.37, 134.53, 133.61, 129.81, 128.62, 127.94, 127.46, 127.29, 126.22, 121.62, 121.43, 116.52, 113.34, 55.16, 47.74, 44.81, 41.95, 19.20. **HRMS (ESI)** m/z Calcd. for $C_{27}H_{26}N_2O_2$ $[M+H]^+$ 411.2067, Found 411.2071.

3-(4-Methoxyphenyl)-4-phenyl-*N*-(quinolin-8-yl)hexanamide (5c, from *E*-alkene)

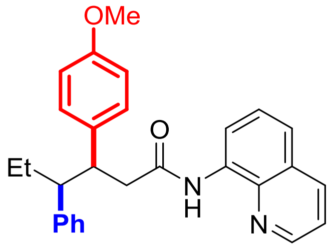

Following the general procedure, the reaction was carried out with **1d** (0.3 mmol, 1.0 equiv), phenylboronic acid **2a** (0.6 mmol, 2.0 equiv), 4-methoxyphenyl iodide **3a** (0.9 mmol, 3.0 equiv), Pd[(-)-sparteine]Cl_2 (0.03 mmol, 10 mol%), (-)-sparteine (0.06 mmol, 20 mol%), Na_2CO_3 (31.8 mg, 0.3 mmol) in the mixture of DCM/H_2O/CH_3CN (2 mL:0.4 mL:0.2 mL) for 48 h at 100 ℃ under a N_2 atmosphere. 3-(4-Methoxyphenyl)-4-phenyl-*N*-(quinolin-8-yl)hexanamide **(5c)** was isolated by column chromatography (PE/acetone = 14/1) as a white wax in 72 % yield. **^{1}H NMR (400 MHz, $CDCl_3$):** δ 9.70 (s, 1H), 8.76 (dd, *J* = 4.2, 1.6 Hz, 1H), 8.73 (dd, *J* = 7.3, 1.5 Hz, 1H), 8.10 (dd, *J* = 8.3, 1.6 Hz, 1H), 7.51 – 7.38 (m, 3H), 7.24 – 7.15 (m, 3H), 6.96 – 6.87 (m, 4H), 6.71 (d, *J* = 8.7 Hz, 2H), 3.72 – 3.63 (m, 4H), 3.00 (dd, *J* = 14.9, 6.7 Hz, 1H), 2.90 (dt, *J* = 10.5, 5.2 Hz, 1H), 2.78 (dd, *J* = 14.9, 8.3 Hz, 1H), 1.95 – 1.83 (m, 1H), 1.68 (tdd, *J* = 13.6, 9.1, 6.2 Hz, 1H), 0.80 (t, *J* = 7.3 Hz, 3H). **^{13}C NMR (100 MHz, $CDCl_3$):** δ 170.62, 158.08, 148.05, 141.55, 138.35, 136.31, 134.54, 133.35, 130.04, 129.63, 127.92, 127.74, 127.41, 126.21, 121.59, 121.40, 116.50, 113.16, 55.12, 52.56, 46.31, 42.51, 26.28, 12.47. **HRMS (ESI)** m/z Calcd. for

$C_{28}H_{28}N_2O_2$ [M+H]$^+$ 425.2224, Found 425.2228.

3-(4-Methoxyphenyl)-4-phenyl-*N*-(quinolin-8-yl)hexanamide (5d, from *Z*-alkene)

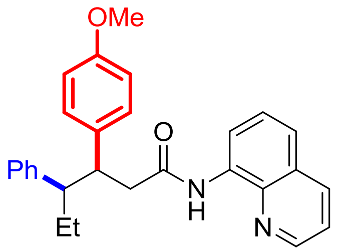

Following the general procedure, the reaction was carried out with **1e** (0.3 mmol, 1.0 equiv), phenylboronic acid **2a** (0.6 mmol, 2.0 equiv), 4-methoxyphenyl iodide **3a** (0.9 mmol, 3.0 equiv), Pd[(-)-sparteine]Cl_2 (0.03 mmol, 10 mol%), (-)-sparteine (0.06 mmol, 20 mol%), Na_2CO_3 (31.8 mg, 0.3 mmol) in the mixture of DCM/H_2O/CH_3CN (2 mL:0.4 mL:0.2 mL) for 48 h at 100 ℃ under a N_2 atmosphere. 3-(4-Methoxyphenyl)-4-phenyl-*N*-(quinolin-8-yl)hexanamide **(5d)** was isolated by column chromatography (PE/acetone = 14/1) as a white solid in 69 % yield. **^{1}H NMR (400 MHz, $CDCl_3$)**: δ 9.40 (s, 1H), 8.69 (dd, *J* = 4.2, 1.6 Hz, 1H), 8.58 (dd, *J* = 7.2, 1.7 Hz, 1H), 8.03 (d, *J* = 8.2 Hz, 1H), 7.43 – 7.28 (m, 9H), 7.23 – 7.18 (m, 1H), 6.84 (d, *J* = 8.6 Hz, 2H), 3.70 (s, 3H), 3.51 (td, *J* = 10.4, 4.3 Hz, 1H), 2.76 – 2.55 (m, 3H), 1.59 – 1.39 (m, 2H), 0.61 (t, *J* = 7.3 Hz, 3H). **^{13}C NMR (100 MHz, $CDCl_3$)**: δ 170.47, 158.16, 147.86, 143.43, 138.12, 136.20, 135.31, 134.42, 129.13, 128.61, 128.55, 127.77, 127.29, 126.51, 121.44, 121.14, 116.21, 113.95, 55.13, 53.93, 47.74, 44.05, 27.22, 12.29. **HRMS (ESI)** m/z Calcd. for $C_{28}H_{28}N_2O_2$ [M+H]$^+$ 425.2224, Found 425.2227.

3-(4-Methoxyphenyl)-4,5-diphenyl-*N*-(quinolin-8-yl)pentanamide (5e)

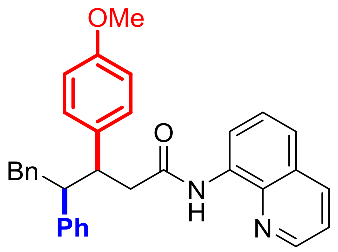

Following the general procedure, the reaction was carried out with **1f** (0.3 mmol, 1.0 equiv), phenylboronic acid **2a** (0.6 mmol, 2.0 equiv), 4-methoxyphenyl iodide **3a** (0.9

mmol, 3.0 equiv), Pd[(-)-sparteine]Cl_2 (0.03 mmol, 10 mol%), (-)-sparteine (0.06 mmol, 20 mol%), Na_2CO_3 (31.8 mg, 0.3 mmol) in the mixture of DCM/H_2O/CH_3CN (2 mL:0.4 mL:0.2 mL) for 48 h at 100 ℃ under a N_2 atmosphere. 3-(4-Methoxyphenyl)-4,5-diphenyl-*N*-(quinolin-8-yl)pentanamide **(5e)** was isolated by column chromatography (PE/acetone = 11/1) as a white wax in 76 % yield. **^{1}H NMR (400 MHz, CDCl$_3$)**: δ 9.67 (s, 1H), 8.77 – 8.70 (m, 2H), 8.12 (dd, *J* = 8.3, 1.6 Hz, 1H), 7.53 – 7.40 (m, 3H), 7.19 – 7.10 (m, 5H), 7.05 (d, *J* = 7.2 Hz, 3H), 6.93 (ddd, *J* = 9.0, 6.4, 3.8 Hz, 4H), 6.79 – 6.70 (m, 2H), 3.80 – 3.72 (m, 4H), 3.44 (ddd, *J* = 11.1, 10.4, 6.3 Hz, 1H), 3.18 (dd, *J* = 14.0, 5.7 Hz, 1H), 3.04 (dd, *J* = 14.9, 6.7 Hz, 1H), 2.93 (dd, *J* = 13.9, 9.5 Hz, 1H), 2.82 (dd, *J* = 14.9, 8.3 Hz, 1H). **^{13}C NMR (100 MHz, CDCl$_3$)**: δ 170.41, 158.24, 148.07, 140.75, 140.42, 138.35, 136.37, 134.51, 132.70, 130.31, 129.89, 129.14, 128.15, 127.95, 127.68, 127.45, 126.41, 125.81, 121.63, 121.47, 116.53, 113.25, 55.19, 51.86, 45.83, 42.79, 39.77. **HRMS (ESI)** m/z Calcd. for $C_{33}H_{30}N_2O_2$ $[M+H]^+$ 487.2380, Found 487.2385.

3-(4-Methoxyphenyl)-4,6-diphenyl-*N*-(quinolin-8-yl)hexanamide (5f)

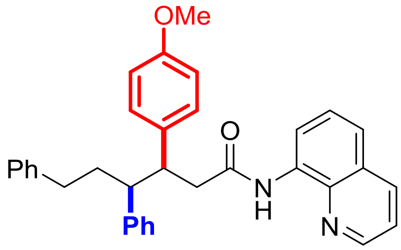

Following the general procedure, the reaction was carried out with **1g** (0.3 mmol, 1.0 equiv), phenylboronic acid **2a** (0.6 mmol, 2.0 equiv), 4-methoxyphenyl iodide **3a** (1.2 mmol, 4.0 equiv), Pd[(-)-sparteine]Cl_2 (0.03 mmol, 10 mol%), (-)-sparteine (0.06 mmol, 20 mol%), Na_2CO_3 (31.8 mg, 0.3 mmol) in the mixture of DCM/H_2O/CH_3CN (2 mL:0.4 mL:0.2 mL) for 36 h at 100 ℃ under a N_2 atmosphere. 3-(4-Methoxyphenyl)-4,6-diphenyl-*N*-(quinolin-8-yl)hexanamide **(5f)** was isolated by column chromatography (PE/acetone = 11/1) as a white wax in 79 % yield. **^{1}H NMR (400 MHz, CDCl$_3$):** δ 9.70 (s, 1H), 8.76 (dd, *J* = 4.2, 1.6 Hz, 1H), 8.72 (dd, *J* = 7.2, 1.5 Hz, 1H), 8.19 – 7.95 (m, 1H), 7.51 – 7.44 (m, 2H), 7.41 (dd, *J* = 8.3, 4.2 Hz, 1H),

7.23 (td, *J* = 7.4, 3.3 Hz, 5H), 7.16 (t, *J* = 7.3 Hz, 1H), 7.09 (d, *J* = 7.1 Hz, 2H), 7.02 – 6.94 (m, 2H), 6.90 (d, *J* = 8.6 Hz, 2H), 6.70 (d, *J* = 8.6 Hz, 2H), 3.80 – 3.56 (m, 4H), 3.09 – 2.82 (m, 2H), 2.76 (dd, *J* = 14.9, 8.4 Hz, 1H), 2.46 (t, *J* = 7.9 Hz, 2H), 2.30 – 1.88 (m, 2H). **^{13}C NMR (100 MHz, $CDCl_3$):** δ 170.55, 158.15, 148.10, 142.49, 141.09, 138.36, 136.38, 134.48, 132.86, 130.15, 129.76, 128.50, 128.36, 127.93, 127.45, 126.49, 125.78, 121.64, 121.49, 116.56, 113.20, 55.16, 50.22, 46.55, 42.45, 35.52, 34.04. **HRMS (ESI)** m/z Calcd. for $C_{34}H_{32}N_2O_2$ $[M+H]^+$ 501.2537, Found 501.2540.

7-(Benzyloxy)-3-(4-methoxyphenyl)-4-phenyl-N-(quinolin-8-yl)heptanamide (5g)

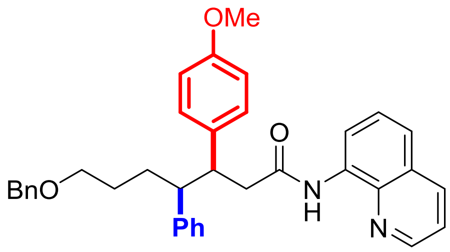

Following the general procedure, the reaction was carried out with **1h** (0.2 mmol, 1.0 equiv), phenylboronic acid **2a** (0.4 mmol, 2.0 equiv), 4-methoxyphenyl iodide **3a** (0.8 mmol, 4.0 equiv), Pd[(-)-sparteine]Cl_2 (0.02 mmol, 10 mol%), (-)-sparteine (0.04 mmol, 20 mol%), Na_2CO_3 (0.2 mmol, 1.0 equiv) in the mixture of DCM/H_2O/CH_3CN (2 mL:0.4 mL:0.2 mL) for 36 h at 100 ℃ under a N_2 atmosphere. 7-(Benzyloxy)-3-(4-methoxyphenyl)-4-phenyl-N-(quinolin-8-yl)heptanamide **(5g)** was isolated by column chromatography (PE/acetone = 9/1) as a white solid in 69 % yield. **^{1}H NMR (400 MHz, $CDCl_3$)**: δ 9.60 (s, 1H), 8.66 (d, *J* = 4.1 Hz, 1H), 8.63 (d, *J* = 7.2 Hz, 1H), 8.00 (d, *J* = 8.2 Hz, 1H), 7.41 – 7.33 (m, 2H), 7.31 (dd, *J* = 8.2, 4.2 Hz, 1H), 7.21 (dt, *J* = 16.0, 7.9 Hz, 5H), 7.13 – 7.06 (m, 3H), 6.87 – 6.78 (m, 4H), 6.60 (d, *J* = 8.2 Hz, 2H), 4.34 (s, 2H), 3.64 – 3.51 (m, 4H), 3.36 – 3.25 (m, 2H), 2.90 (dd, *J* = 14.5, 6.5 Hz, 2H), 2.67 (dd, *J* = 14.9, 8.5 Hz, 1H), 1.88 (ddd, *J* = 14.3, 10.2, 5.1 Hz, 1H), 1.63 (ddd, *J* = 19.3, 12.3, 5.7 Hz, 1H), 1.46 – 1.29 (m, 2H). **^{13}C NMR (100 MHz, $CDCl_3$)**: δ 170.52, 158.08, 148.04, 141.40, 138.70, 138.32, 136.31, 134.50, 133.15, 130.04, 129.55, 128.38, 127.90, 127.80, 127.69, 127.51, 127.39, 126.29,

121.58, 121.40, 116.48, 113.17, 72.78, 70.21, 55.10, 50.45, 46.56, 42.41, 29.88, 27.97. **HRMS (ESI)** m/z Calcd. for $C_{36}H_{36}N_2O_3$ $[M+H]^+$ 545.2799, Found 545.2802.

6-(1,3-Dioxoisoindolin-2-yl)-3-(4-methoxyphenyl)-4-phenyl-*N*-(quinolin-8-yl)hexanamide (5h)

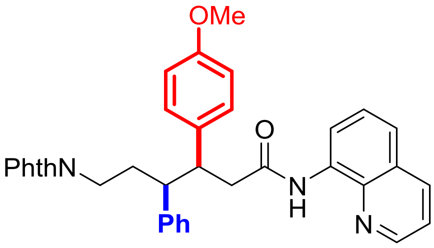

Following the general procedure, the reaction was carried out with **1i** (0.3 mmol, 1.0 equiv), phenylboronic acid **2a** (0.6 mmol, 2.0 equiv), 4-methoxyphenyl iodide **3a** (1.2 mmol, 4.0 equiv), Pd[(-)-sparteine]Cl_2 (0.03 mmol, 10 mol%), (-)-sparteine (0.06 mmol, 20 mol%), Na_2CO_3 (31.8 mg, 0.3 mmol) in the mixture of DCM/H_2O/CH_3CN (2 mL:0.4 mL:0.2 mL) for 36 h at 100 ℃ under a N_2 atmosphere. 6-(1,3-Dioxoisoindolin-2-yl)-3-(4-methoxyphenyl)-4-phenyl-*N*-(quinolin-8-yl)hexanamide **(5h)** was isolated by column chromatography (PE/acetone = 8/1) as a white solid in 59 % yield. **^{1}H NMR (400 MHz, $CDCl_3$)**: δ 9.66 (s, 1H), 8.75 (dd, *J* = 4.2, 1.6 Hz, 1H), 8.66 (dd, *J* = 6.3, 2.7 Hz, 1H), 8.12 (dd, *J* = 8.3, 1.6 Hz, 1H), 7.73 (dt, *J* = 7.1, 3.6 Hz, 2H), 7.67 – 7.63 (m, 2H), 7.47 (dd, *J* = 9.4, 5.4 Hz, 2H), 7.42 (dd, *J* = 8.3, 4.3 Hz, 1H), 7.16 (t, *J* = 7.3 Hz, 2H), 7.09 (t, *J* = 7.2 Hz, 1H), 6.95 (d, *J* = 7.1 Hz, 2H), 6.87 (d, *J* = 8.6 Hz, 2H), 6.68 (d, *J* = 8.6 Hz, 2H), 3.70 (s, 3H), 3.68 – 3.50 (m, 3H), 3.15 – 3.08 (m, 1H), 2.94 (dd, *J* = 15.0, 6.7 Hz, 1H), 2.74 (dd, *J* = 15.0, 8.4 Hz, 1H), 2.20 – 2.07 (m, 2H). **^{13}C NMR (100 MHz, $CDCl_3$)**: δ 170.21, 168.30, 158.29, 148.10, 139.86, 138.34, 136.36, 134.46, 133.78, 132.22, 132.16, 130.18, 129.62, 127.92, 127.43, 126.62, 123.09, 121.63, 121.46, 116.54, 113.28, 55.17, 48.42, 46.49, 41.99, 37.01, 31.93. **HRMS (ESI)** m/z Calcd. for $C_{36}H_{31}N_3O_4$ $[M+Na]^+$ 592.2207, Found 592.2210.

Methyl-6-(4-methoxyphenyl)-8-oxo-5-phenyl-8-(quinolin-8-ylamino)octanoate (5i)

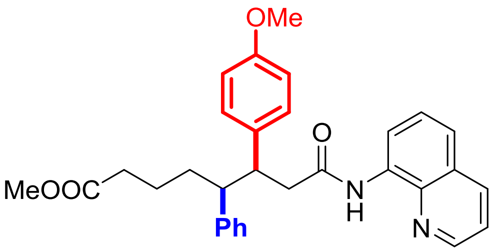

Following the general procedure, the reaction was carried out with **1j** (0.3 mmol, 1.0 equiv), phenylboronic acid **2a** (0.6 mmol, 2.0 equiv), 4-methoxyphenyl iodide **3a** (1.2 mmol, 4.0 equiv), Pd[(-)-sparteine]Cl_2 (0.03 mmol, 10 mol%), (-)-sparteine (0.06 mmol, 20 mol%), Na_2CO_3 (31.8 mg, 0.3 mmol) in the mixture of DCM/H_2O/CH_3CN (2 mL:0.4 mL:0.2 mL) for 36 h at 100 ℃ under a N_2 atmosphere. Methyl -6-(4-methoxyphenyl)-8-oxo-5-phenyl-8-(quinolin-8-ylamino)octanoate **(5i)** was isolated by column chromatography (PE/acetone = 9/1) as a white solid in 74 % yield. **^{1}H NMR (400 MHz, CDCl$_3$)**: δ 9.67 (s, 1H), 8.76 (dd, *J* = 4.2, 1.6 Hz, 1H), 8.70 (dd, *J* = 6.9, 2.0 Hz, 1H), 8.12 (dd, *J* = 8.3, 1.6 Hz, 1H), 7.51 – 7.44 (m, 2H), 7.42 (dd, *J* = 8.3, 4.3 Hz, 1H), 7.23 – 7.15 (m, 3H), 6.90 (dd, *J* = 12.6, 5.2 Hz, 4H), 6.69 (d, *J* = 8.6 Hz, 2H), 3.70 (s, 3H), 3.66 – 3.59 (m, 4H), 2.98 (dt, *J* = 14.8, 6.8 Hz, 2H), 2.76 (dd, *J* = 14.9, 8.4 Hz, 1H), 2.24 (t, *J* = 7.5 Hz, 2H), 1.90 – 1.79 (m, 1H), 1.71 – 1.63 (m, 1H), 1.57 – 1.40 (m, 2H). **^{13}C NMR (100 MHz, CDCl$_3$)**: δ 174.07, 170.52, 158.16, 148.12, 141.12, 138.39, 136.40, 134.51, 133.02, 130.09, 129.56, 127.98, 127.91, 127.47, 126.46, 121.66, 121.49, 116.57, 113.24, 55.18, 51.56, 50.43, 46.49, 42.35, 34.12, 32.82, 23.27. **HRMS (ESI)** m/z Calcd. for $C_{31}H_{32}N_2O_4$ $[M+H]^+$ 497.2435, Found 497.2438.

3-(4-Methoxyphenyl)-4,4-diphenyl-*N*-(quinolin-8-yl)butanamide (5j)

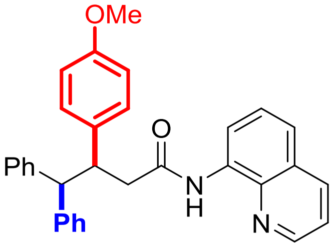

Following the general procedure, the reaction was carried out with **1k** (0.3 mmol, 1.0 equiv), phenylboronic acid **2a** (0.6 mmol, 2.0 equiv), 4-methoxyphenyl iodide **3a** (1.2 mmol, 4.0 equiv), Pd[(-)-sparteine]Cl_2 (0.03 mmol, 10 mol%), (-)-sparteine (0.06 mmol, 20 mol%), Na_2CO_3 (31.8 mg, 0.3 mmol) in the mixture of DCM/H_2O/CH_3CN (2 mL:0.4 mL:0.2 mL) for 36 h at 100 ℃ under a N_2 atmosphere. 3-(4-Methoxyphenyl)-4,4-diphenyl-*N*-(quinolin-8-yl)butanamide **(5j)** was isolated by column chromatography (PE/acetone = 11/1) as a white solid in 49 % yield. **^{1}H NMR (400 MHz, $CDCl_3$)**: δ 9.43 (s, 1H), 8.70 (dd, *J* = 4.2, 1.7 Hz, 1H), 8.64 (dd, *J* = 7.3, 1.5 Hz, 1H), 8.07 (dd, *J* = 8.3, 1.6 Hz, 1H), 7.52 (d, *J* = 7.3 Hz, 2H), 7.47 – 7.30 (m, 5H), 7.23 – 7.14 (m, 5H), 7.10 (t, *J* = 7.6 Hz, 2H), 6.99 (t, *J* = 7.3 Hz, 1H), 6.68 – 6.61 (m, 2H), 4.25 (d, *J* = 3.8 Hz, 2H), 3.59 (s, 3H), 2.94 (dd, *J* = 14.6, 2.0 Hz, 1H), 2.79 – 2.67 (m, 1H). **^{13}C NMR (100 MHz, $CDCl_3$)**: δ 170.29, 157.86, 147.95, 143.51, 143.23, 138.21, 136.28, 134.46, 134.41, 129.28, 128.99, 128.39, 128.35, 128.24, 127.85, 127.37, 126.70, 125.96, 121.52, 121.30, 116.34, 113.71, 58.16, 55.02, 45.77, 44.34. **HRMS (ESI)** m/z Calcd. for $C_{32}H_{28}N_2O_2$ $[M+H]^+$ 473.2224, Found 473.2227.

3,4-Bis(4-methoxyphenyl)-4-phenyl-*N*-(quinolin-8-yl)butanamide (5k)

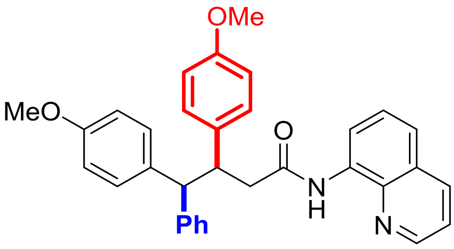

Following the general procedure, the reaction was carried out with **1l** (0.3 mmol, 1.0 equiv), phenylboronic acid **2a** (0.6 mmol, 2.0 equiv), 4-methoxyphenyl iodide **3a** (1.2 mmol, 4.0 equiv), Pd[(-)-sparteine]Cl_2 (0.03 mmol, 10 mol%), (-)-sparteine (0.06 mmol, 20 mol%), Na_2CO_3 (31.8 mg, 0.3 mmol) in the mixture of DCM/H_2O/CH_3CN (2 mL:0.4 mL:0.2 mL) for 36 h at 100 ℃ under a N_2 atmosphere. 3,4-Bis(4-methoxyphenyl)-4-phenyl-*N*-(quinolin-8-yl)butanamide **(5k)** was isolated by column chromatography (PE/acetone = 9/1) as a white solid in 49 % yield. **^{1}H NMR (400 MHz, $CDCl_3$)**: δ 9.40 (s, 1H), 8.70 (dd, *J* = 4.2, 1.6 Hz, 1H), 8.63 (dd, *J* = 7.1, 1.7 Hz, 1H), 8.08 (dd, *J* = 8.2, 1.5 Hz, 1H), 7.46 – 7.36 (m, 5H), 7.18 (d, *J* = 8.6 Hz, 4H), 7.09 (t,

J = 7.6 Hz, 2H), 6.98 (t, *J* = 7.3 Hz, 1H), 6.83 (d, *J* = 8.6 Hz, 2H), 6.64 (d, *J* = 8.6 Hz, 2H), 4.19 (d, *J* = 3.4 Hz, 2H), 3.67 (s, 3H), 3.60 (s, 3H), 2.94 (dd, *J* = 14.6, 2.2 Hz, 1H), 2.77 – 2.67 (m, 1H). **^{13}C NMR (101 MHz, $CDCl_3$)**: δ 170.47, 158.26, 157.82, 147.92, 143.60, 138.21， 136.37, 135.63, 134.69, 134.46, 129.35, 129.25, 128.23, 127.88, 127.42, 125.86, 121.54, 121.32, 116.41, 114.32, 113.70, 57.38, 55.23, 55.04, 45.96, 44.46. **HRMS (ESI)** m/z Calcd. for $C_{33}H_{30}N_2O_3$ $[M+H]^+$ 503.2329, Found 503.2333.

4-(4-Bromophenyl)-3-(4-methoxyphenyl)-4-phenyl-*N*-(quinolin-8-yl)butanamide (5l)

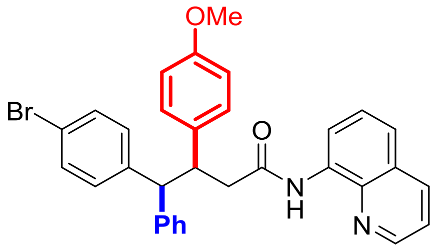

Following the general procedure, the reaction was carried out with **1m** (0.3 mmol, 1.0 equiv), phenylboronic acid **2a** (0.6 mmol, 2.0 equiv), 4-methoxyphenyl iodide **3a** (1.2 mmol, 4.0 equiv), Pd[(-)-sparteine]Cl_2 (0.03 mmol, 10 mol%), (-)-sparteine (0.06 mmol, 20 mol%), Na_2CO_3 (31.8 mg, 0.3 mmol) in the mixture of DCM/H_2O/CH_3CN (2 mL:0.4 mL:0.2 mL) for 36 h at 100 ℃ under a N_2 atmosphere. 4-(4-Bromophenyl)-3-(4-methoxyphenyl)-4-phenyl-*N*-(quinolin-8-yl)butanamide **(5l)** was isolated by column chromatography (PE/acetone = 11/1) as a white solid in 43 % yield. **^{1}H NMR (400 MHz, $CDCl_3$)**: δ 9.42 (s, 1H), 8.70 (dd, *J* = 4.0, 1.6 Hz, 1H), 8.63 (d, *J* = 7.2 Hz, 1H), 8.07 (dd, *J* = 8.2, 1.6 Hz, 1H), 7.49 – 7.35 (m, 7H), 7.17 (dd, *J* = 5.4, 3.3 Hz, 4H), 7.10 (dd, *J* = 9.5, 4.2 Hz, 2H), 7.05 – 6.97 (m, 1H), 6.64 (dd, *J* = 4.7, 3.9 Hz, 2H), 4.23 (d, *J* = 7.2 Hz, 2H), 3.59 (s, 3H), 2.94 – 2.83 (m, 1H), 2.73 (dd, *J* = 14.0, 7.5 Hz, 1H). **^{13}C NMR (101 MHz, $CDCl_3$)**: δ 170.05, 157.95, 147.96, 142.62, 142.55, 138.16, 136.30, 134.35, 134.18, 132.00, 130.15, 129.21, 128.36, 128.23, 127.88, 127.35, 126.20, 121.54, 121.43, 120.57, 116.36, 113.77, 57.48, 55.01, 45.58, 44.29. **HRMS (ESI)** m/z Calcd. for $C_{32}H_{27}BrN_2O_2$ $[M+H]^+$ 551.1329, Found 551.1333.

2-(4-Methoxyphenyl)-3-phenyl-*N*-(quinolin-8-yl)cyclohexane-1-carboxamide (5m)

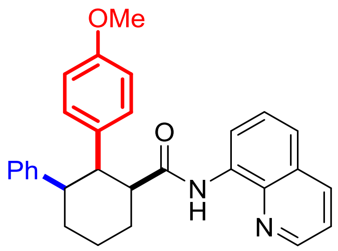

Following the general procedure, the reaction was carried out with **1n** (0.3 mmol, 1.0 equiv), phenylboronic acid **2a** (0.6 mmol, 2.0 equiv), 4-methoxyphenyl iodide **3a** (1.2 mmol, 4.0 equiv), Pd[(-)-sparteine]Cl_2 (0.03 mmol, 10 mol%), (-)-sparteine (0.06 mmol, 20 mol%), Na_2CO_3 (31.8 mg, 0.3 mmol) in the mixture of DCM/H_2O/CH_3CN (2 mL:0.4 mL:0.2 mL) for 36 h at 100 ℃ under a N_2 atmosphere. 2-(4-Methoxyphenyl)-3-phenyl-*N*-(quinolin-8-yl)cyclohexane-1-carboxamide **(5m)** was isolated by column chromatography (PE/acetone = 14/1) as a white solid in 29 % yield. **^{1}H NMR (400 MHz, CDCl$_3$)**: δ 9.83 (s, 1H), 8.83 – 8.72 (m, 1H), 8.54 (dd, *J* = 5.6, 3.3 Hz, 1H), 8.13 – 8.04 (m, 1H), 7.40 (dd, *J* = 6.7, 3.8 Hz, 3H), 7.15 – 6.98 (m, 5H), 6.92 (d, *J* = 7.1 Hz, 2H), 6.50 (d, *J* = 8.6 Hz, 2H), 3.76 (t, *J* = 4.8 Hz, 1H), 3.56 (s, 3H), 3.24 (dt, *J* = 9.3, 4.3 Hz, 2H), 2.34 (dddd, *J* = 31.4, 21.5, 15.6, 7.9 Hz, 4H), 1.88 – 1.69 (m, 2H). **^{13}C NMR (100 MHz, CDCl$_3$):** δ 172.48, 157.90, 148.06, 144.30, 138.58, 136.29, 134.45, 131.78, 130.79, 127.95, 127.89, 127.85, 127.43, 125.95, 121.51, 121.15, 116.41, 113.00, 54.93, 51.07, 49.52, 48.73, 25.63, 25.39, 23.38. **HRMS (ESI)** m/z Calcd. for $C_{29}H_{28}N_2O_2$ $[M+H]^+$ 437.2224, Found 437.2228.

4 Screening of Asymmetric Reaction Conditions

4.1 Evaluation of chiral ligands

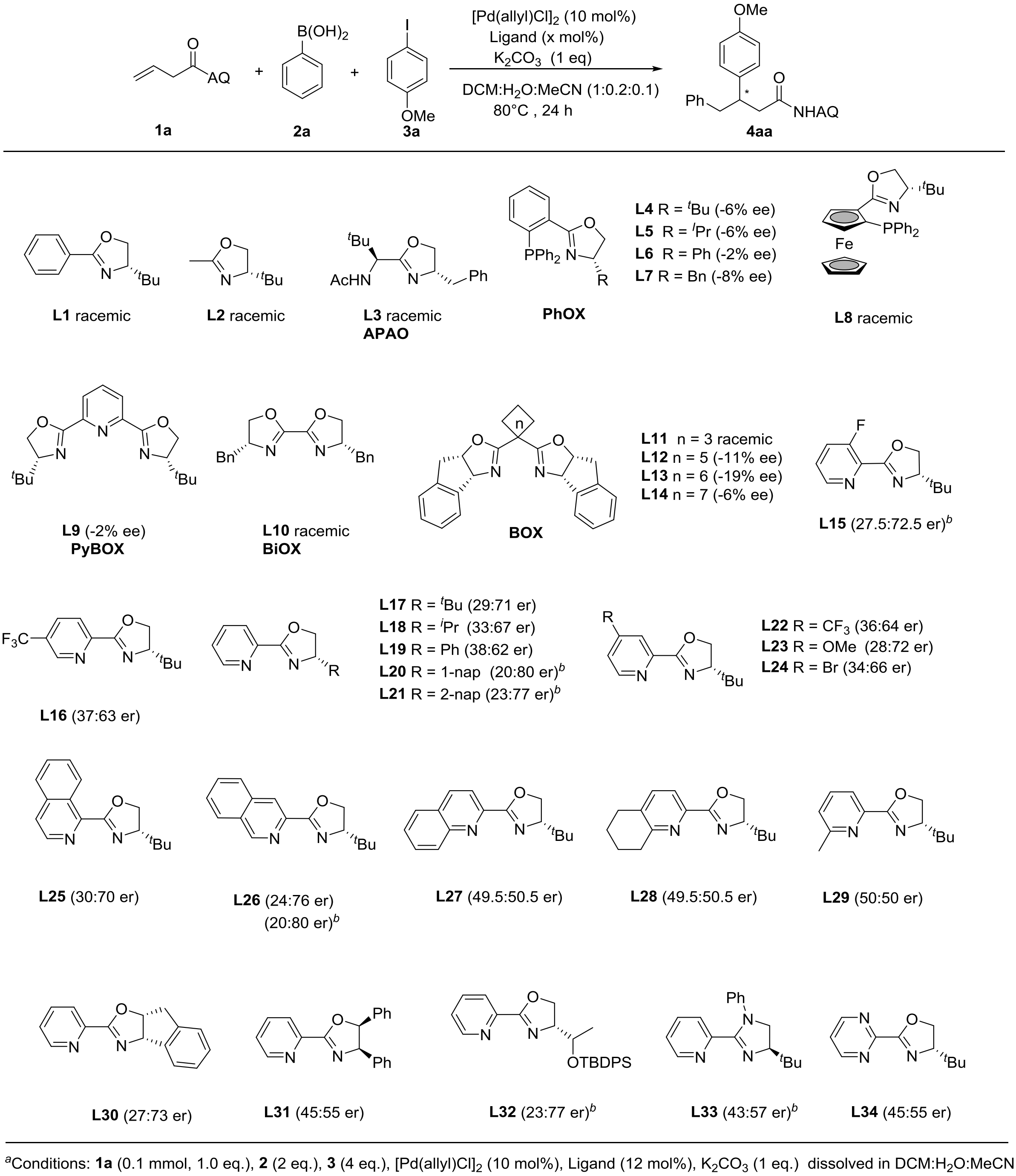

[a]Conditions: **1a** (0.1 mmol, 1.0 eq.), **2** (2 eq.), **3** (4 eq.), $[Pd(allyl)Cl]_2$ (10 mol%), Ligand (12 mol%), K_2CO_3 (1 eq.) dissolved in $DCM:H_2O:MeCN$ (1 mL:0.2mL:0.1mL) and heated to 80 °C in 24 h. [b]$[Pd(allyl)Cl]_2$ (10 mol%), Ligand (24 mol%), $DCM:H_2O:MeCN$ (0.5 mL+0.1 mL+0.05 mL).

Figure S2. Evaluation of chiral ligands

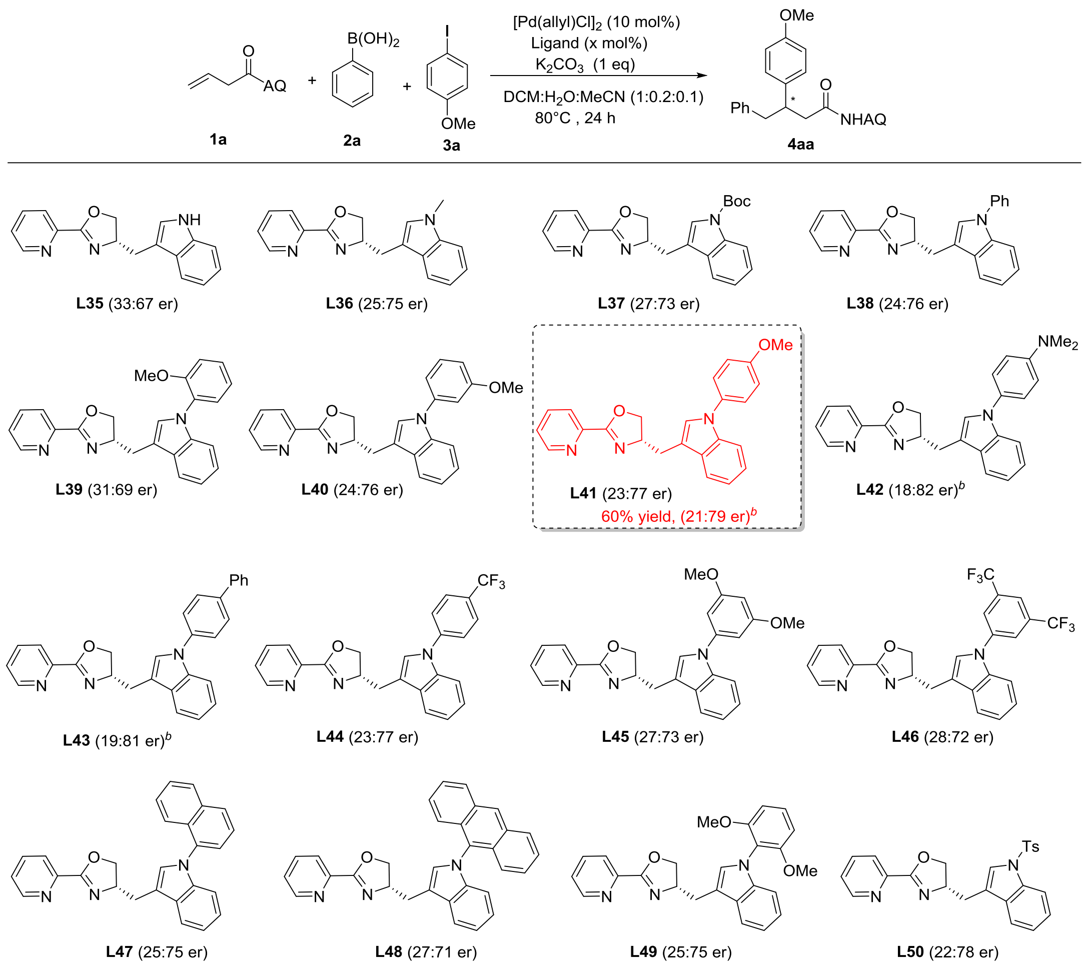

[a]Conditions: **1a** (0.1 mmol, 1eq.), **2** (2 eq.), **3** (4 eq.), $[Pd(allyl)Cl]_2$ (10 mol%), ligand (12 mol%), K_2CO_3 (1 eq.) were dissolved in $DCM:H_2O:MeCN$ (1 mL:0.2mL:0.1mL) and heated to 80 °C in 24 h. [b]$[Pd(allyl)Cl]_2$ (10 mol%), Ligand (24 mol%), $DCM:H_2O:MeCN$ (0.5 mL:0.1 mL:0.05 mL).

Figure S3. Evaluation of PyOX(NH) (PyOX(N-Ar))

4.2 General procedure for preparation of PyOX(NH) (PyOX(N-Ar))

PyOX(NH) (**PyOX(N-Ar)**) ligands were synthesized following the reported procedures. [8-9] Herein we take **L41** as an example for the operation in detail.

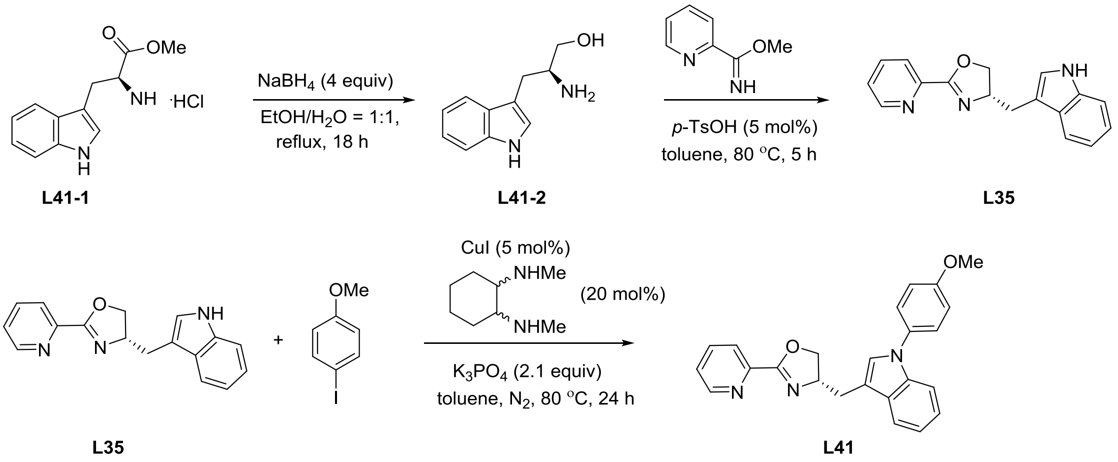

Step 1: The reduction of L-Tryptophane[10-11]

To a solution of **L41-1** (22.5 g, 0.089 mol) in a 1:1 (v/v) mixture (200 mL) of water and ethanol, was added slowly with stirring a solution of sodium borohydride (11.6 g, 0.306 mol) in 50 mL of the same solvent. When the addition of borohydride was complete the mixture was heated to reflux for 18 h. After been cooled to room temperature, the solution was evaporated under reduced pressure to remove ethanol. The residual aqueous solution was treated with sodium hydroxide to hydrolyze esters of boric acid and then with sodium chloride to saturate the solution before exhaustive extraction with ethyl acetate. The combined organic phase was washed with water, dried over anhydrous Na_2SO_4, and evaporated to dryness, affording 12 g (70%) of **L41-2** as a glassy solid, which was used without purification in the next step.

Step 2: Preparation of PyOX(NH)

To a flame-dried round bottom flask charged with a stir bar was added crude methoxyimidate (2.04 g, 15 mmol, 1.0 equiv), **L41-2** (3.14 g, 16.5 mmol, 1.1 equiv), toluene (100 mL), and *p*-TsOH ·H_2O (143 mg, 0.75 mmol, 5 mol %). The mixture was stirred at 80 ℃ in an oil bath for 5 h, at which time the starting material was consumed as indicated by TLC analysis (45:1 DCM/ Methanol). The reaction was cooled to ambient temperature and quenched with sat. $NaHCO_3$ (60 mL). The reaction was partitioned with EtOAc and water, and the aqueous phase was extracted with EtOAc (3 × 50 mL). The combined organic extracts were washed with water (2 × 50 mL), dried ($MgSO_4$) and concentrated in vacuo. And the mixture was purified by silica gel flash chromatography using 50:1 DCM/methanol to give the product (3.3 g, 12 mmol, 80%) **L35** as a pale-yellow solid. **^{1}H NMR (400 MHz, $CDCl_3$):** δ 8.71 (d, *J* = 4.6 Hz, 1H), 8.30 (s, 1H), 8.07 (d, *J* = 7.9 Hz, 1H), 7.78 (td, *J* = 7.8, 1.6 Hz, 1H), 7.67 (d, *J* = 7.8 Hz, 1H), 7.42 – 7.30 (m, 2H), 7.14 (ddd, *J* = 26.7, 17.3, 4.7 Hz, 3H), 4.78 (ddd, *J* = 16.8, 9.1, 5.0 Hz, 1H), 4.44 (t, *J* = 9.0 Hz, 1H), 4.27 (t, *J* = 8.1 Hz, 1H), 3.41 (dd, *J* = 14.5, 4.8 Hz, 1H), 2.92 (dd, *J* = 14.5, 9.0 Hz, 1H).

Step 3: Preparation of PyOX(N-Ar)[12]

To a 150 mL seal tube with a stir bar, was added **L35** (2.2 g, 8 mmol), CuI (76 mg, 0.4 mmol), K_3PO_4 (3.6 g, 16.8 mmol) and toluene (30 mL). 4-methoxyphenyl iodide (2.8 g, 1.5 equiv), *N,N'-d*imethyl-1,2-cyclohexanediamine (224 mg, 20 mol%) were then added successively under a stream of nitrogen. The reaction tube was sealed and the reaction mixture was heated to 80 ℃ for 24 h. The reaction mixture was then cooled to ambient temperature, diluted with ethyl acetate (100×3 mL), filtered through a plug of silica gel and eluted with additional ethyl acetate (50 mL). The filtrate was concentrated and the resulting residue was purified by column chromatography (PE/EA = 1:1 + 0.1% Et_3N) to provide the desired product **L41** (2.5 g, 81% yield, as white solid). (R_f = 0.2, hexane:EtOAc = 1:1 (v/v)). **^{1}H NMR (400 MHz, $CDCl_3$)**: δ 8.72 (d, *J* = 4.4 Hz, 1H), 8.09 (d, *J* = 7.9 Hz, 1H), 7.79 (t, *J* = 7.7 Hz, 1H), 7.71 (d, *J* = 7.5 Hz, 1H), 7.48 – 7.32 (m, 4H), 7.24 – 7.14 (m, 3H), 7.01 (d, *J* = 8.8 Hz, 2H), 4.83 (qd, *J* = 9.1, 4.8 Hz, 1H), 4.51 (t, *J* = 9.0 Hz, 1H), 4.33 (t, *J* = 8.1 Hz, 1H), 3.87 (s, 3H), 3.47 (dd, *J* = 14.6, 4.7 Hz, 1H), 2.97 (dd, *J* = 14.6, 9.1 Hz, 1H).

4.3 Synthesis of chiral 4aa

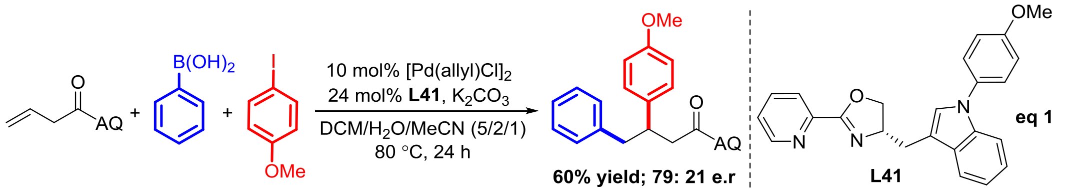

Preparation of the catalyst (10 mol %)

$[Pd(allyl)Cl]_2$ (7.8 mg, 0. 02 mmol, 10 mol%) was added to a solution of **L41** (18.4 mg, 0.048 mmol, 24 mol%) in CH_2Cl_2 (0.2 mL) and the reaction mixture was stirred for 30 min.

The solution containing the catalyst was then added to a solution of alkene substrate **1a** (0.2 mmol, 1.0 equiv), phenylboronic acid **2a** (0.4 mmol, 2.0 equiv), 4-methoxyphenyl iodide **3a** (0.8 mmol, 4.0 equiv), K_2CO_3 (27.6 mg, 0.2 mmol) in the mixture of $DCM/H_2O/CH_3CN$ (0.8 mL:0.2 mL:0.1 mL) for 24 h at 80 ℃ under a N_2 atmosphere. Chiral 3-(4-Methoxyphenyl)-4-phenyl-*N*-(quinolin-8-yl)butanamide (**4aa**)

was isolated by column chromatography (PE/acetone = 11/1) as a white solid in 60% yield. **HPLC** (OD-3, hexane/*i*PrOH = 85:15, detector: 254 nm, flow rate: 0.82 mL/min): 79:21 er, t_{major} = 22.67 min, t_{minor} = 30.96 min.

5. Removal of Directing Group & Derivative Reaction

5.1 Gram-scale Experiment & Removal of Directing Group

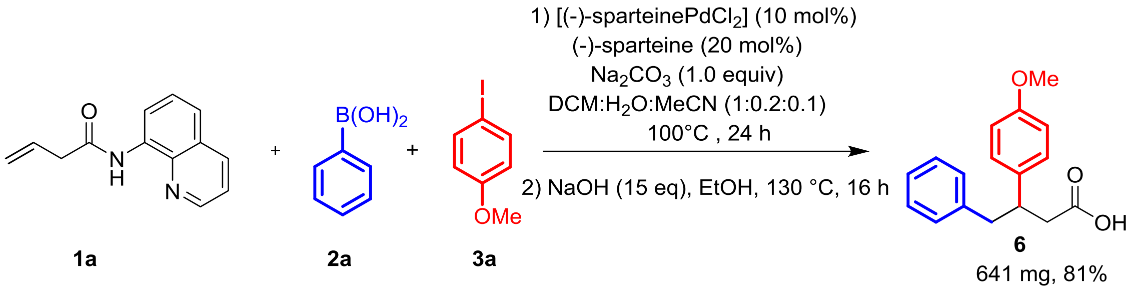

Step 1: Alkene **1a** (636 mg, 3 mmol), phenylboronic acid **2a** (732 mg, 6 mmol), 4-methoxyphenyl iodide **3a** (2.1 g, 9 mmol), Pd[(-)-sparteine]Cl_2 (123 mg, 0.3 mmol), Na_2CO_3 (318 mg, 3 mmol) were added to a 150 mL glass vial containing a stirring bar. After purged with N_2, DCM (20 mL), H_2O (4 mL) and MeCN (2 mL) and (-) sparteine (140 mg, 0.6 mmol) were added, The tube was sealed with a teflon cap and placed in a hotplate pre-heated to 100 ℃ with vigorous stirring for 36 h. After been cooled to room temperature, the mixture was diluted with DCM and washed with water for several times. The organic phase was dried over Na_2SO_4, filtered and concentrated *in vacuo*. The resulting residue was purified by silica gel flash chromatography (PE/acetone = 11/1) to give **4aa** (0.99 g) as a white solid in 84% yield.

Step 2: To an oven-dried 50 mL Schlenk tube equipped with a Teflon stirrer bar were subsequently added 3-(4-methoxyphenyl)-4-phenyl-N-(quinolin-8-yl)butanamide **4aa** (2.5 mmol, 0.99 g), NaOH (37.5 mmol, 1.5 g) and EtOH (25 mL). After stirred at 130 ℃ for 16 h, the reaction was allowed to cool to room temperature, diluted with EtOAc (100 mL) and washed with HCl (1 M, 3 × 50 mL). The organic layers were combined,

dried over Na_2SO_4, concentrated under reduced pressure and purified by flash column chromatography (PE/EA = 5/1) to give the carboxylic acid **6** (641 mg, 95%) as a white solid. **^{1}H NMR (400 MHz, $CDCl_3$)**: δ 7.27 – 7.14 (m, 3H), 7.07 (t, *J* = 7.7 Hz, 4H), 6.83 (t, *J* = 5.8 Hz, 2H), 3.78 (s, 3H), 3.36 (dd, *J* = 14.8, 7.5 Hz, 1H), 2.90 (d, *J* = 7.5 Hz, 2H), 2.65 (qd, *J* = 15.8, 7.5 Hz, 2H).

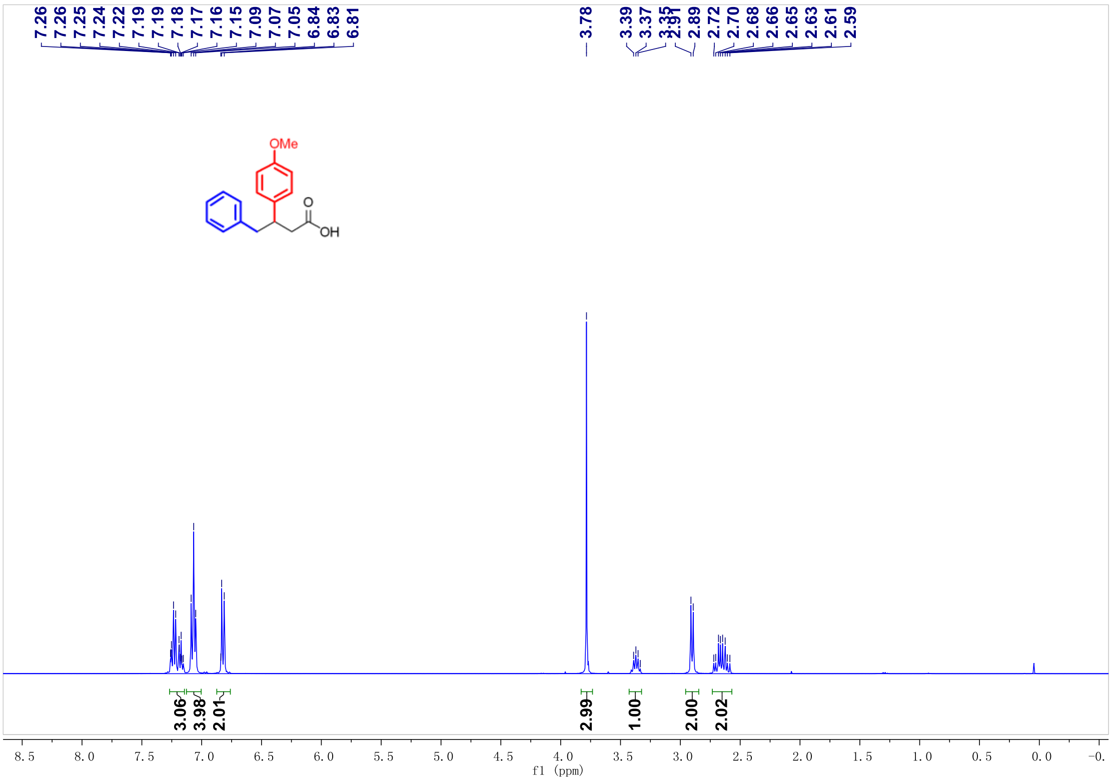

5.2 Synthetic Applications

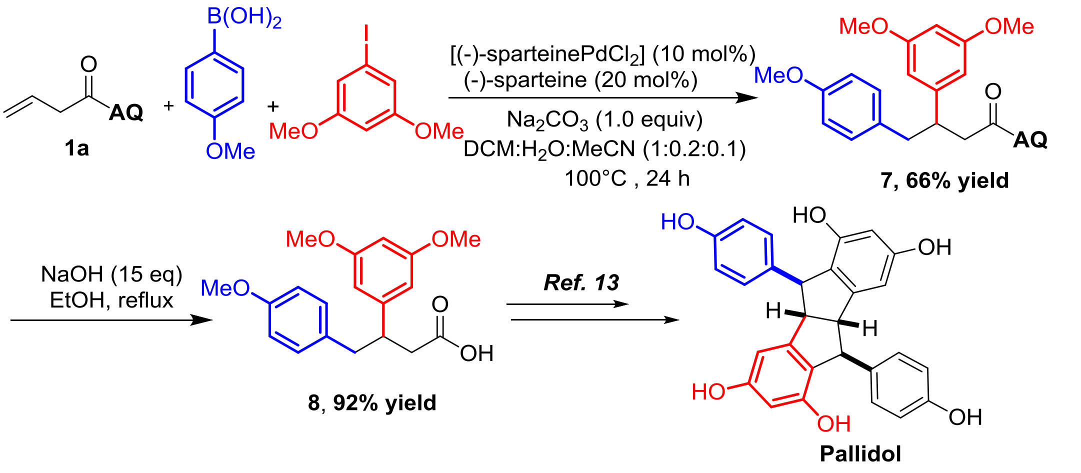

Alkene **1a** (63.6 mg, 0.3 mmol), 4-methoxy-phenyl boronic acid (91.2 mg, 0.6 mmol), 1-iodo-3,5-dimethoxybenzene (317 g, 1.2 mmol), Pd[(-)-sparteine]Cl_2 (12.3 mg, 0.03 mmol), Na_2CO_3 (31.8 mg, 0.3 mmol) were added to a 150 mL glass vial containing a stirring bar. After purged with N_2, DCM (2 mL), H_2O (0.4 mL) and MeCN (0.2 mL) and (-) sparteine (14 mg, 0.06 mmol) were added, The tube was sealed with a teflon cap and placed in a hotplate pre-heated to 100 ℃ with vigorous stirring for 36 h. After been cooled to room temperature, the reaction mixture was dried over Na_2SO_4 and filtered through a pad of celite and concentrated in vacuo. The resulting residue was purified by silica gel flash chromatography (PE/acetone = 9/1) to give 3-(3,5-dimethoxyphenyl)-4-(4-methoxyphenyl)-*N*-(quinolin-8-yl)butanamide **7** as a white solid in 66% yield. **^{1}H NMR (400 MHz, $CDCl_3$)**: δ 9.67 (s, 1H), 8.74 (dd, *J* = 4.2, 1.6 Hz, 1H), 8.71 (dd, *J* = 7.2, 1.6 Hz, 1H), 8.10 (dd, *J* = 8.3, 1.5 Hz, 1H), 7.51 – 7.43 (m, 2H), 7.41 (dd, *J* = 8.3, 4.2 Hz, 1H), 7.04 (d, *J* = 8.6 Hz, 2H), 6.75 (d, *J* = 8.6 Hz, 2H), 6.41 (d, *J* = 2.2 Hz, 2H), 6.25 (t, *J* = 2.2 Hz, 1H), 3.72 (s, 3H), 3.70 (s, 6H), 3.60 – 3.50 (m, 1H), 2.97 (d, *J* = 7.2 Hz, 2H), 2.86 (dd, *J* = 7.3, 2.3 Hz, 2H). **^{13}C NMR (100 MHz, $CDCl_3$)**: δ 170.24, 160.82, 158.05, 148.05, 146.24, 138.32, 136.32, 134.50, 131.70, 130.34, 127.92, 127.39, 121.60, 121.43, 116.45, 113.69, 105.85, 98.64, 55.30, 55.25, 44.77, 43.97, 41.99. **HRMS (ESI)** m/z Calcd. for $C_{28}H_{28}N_2O_4$ $[M+H]^+$ 457.2122, Found 457.2125.

To an oven-dried 25 mL Schlenk tube equipped with a Teflon stirrer bar were subsequently added 3-(3,5-dimethoxyphenyl)-4-(4-methoxyphenyl)-N-(quinolin-8-yl) -butanamide **7** (0.2 mmol, 91.2 mg), NaOH (3 mmol, 120 mg) and EtOH (1 mL). After stirred at 130 ℃ for 16 h, the reaction was allowed to cool to room temperature, diluted with EtOAc (100 mL) and washed with HCl (1 M, 3 × 50 mL). The organic layers were combined, dried over Na_2SO_4, concentrated under reduced pressure and purified by flash column chromatography (PE/EA = 5/1) to give 3-(3,5-dimethoxyphenyl)-4-(4-methoxyphenyl)butanoic acid **8** (61 mg, 92%) as a white solid. **^{1}H NMR (400 MHz, $CDCl_3$)**: δ 6.98 (d, *J* = 8.5 Hz, 2H), 6.77 (d, *J* = 8.6 Hz, 2H), 6.29 (s, 3H), 3.77 – 3.67 (m, 9H), 3.27 (dd, *J* = 14.7, 7.4 Hz, 1H), 2.82 (qd, *J* = 13.7, 7.5 Hz, 2H), 2.69 – 2.54 (m, 2H).

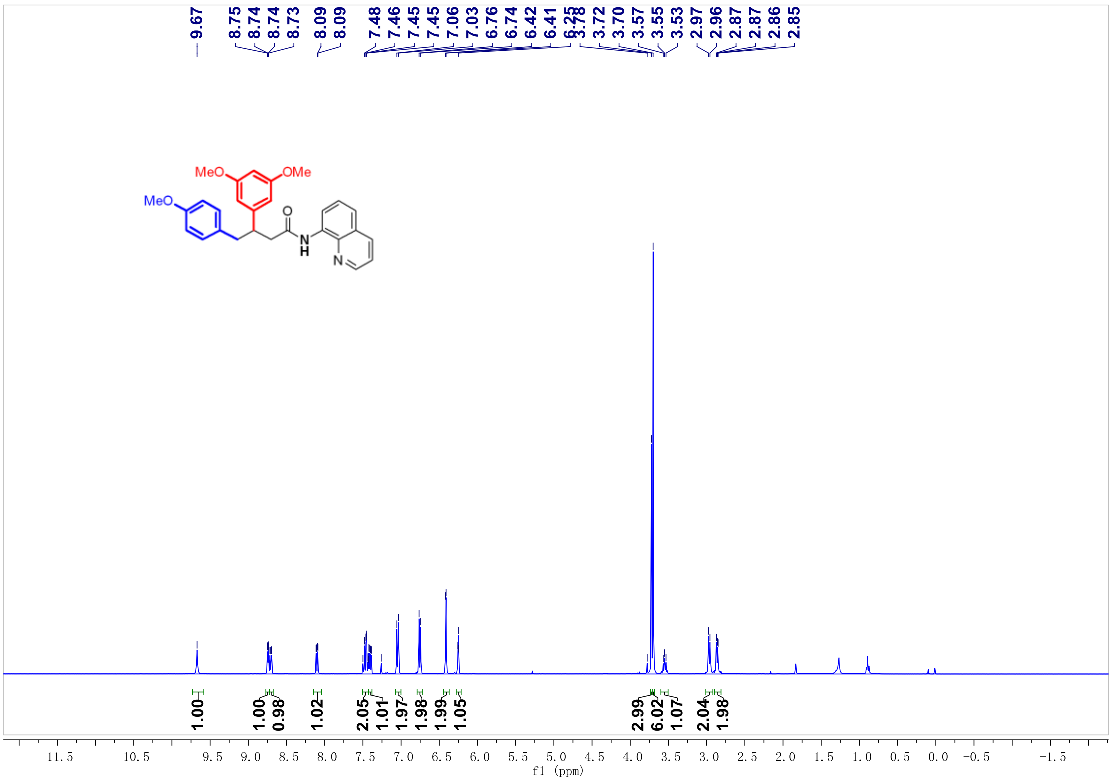
9.67
8.75
8.74
8.74
8.73
8.09
8.09
7.48
7.46
7.45
7.45
7.06
7.03
6.76
6.74
6.42
6.41
6.25
3.78
3.72
3.70
3.57
3.55
3.53
2.97
2.96
2.87
2.87
2.86
2.85
MeO
OMe
MeO
O
N
H
N
1.00
1.00
0.98
1.02
2.05
1.01
1.97
1.98
1.99
1.05
2.99
6.02
1.07
2.04
1.98
f1 (ppm)

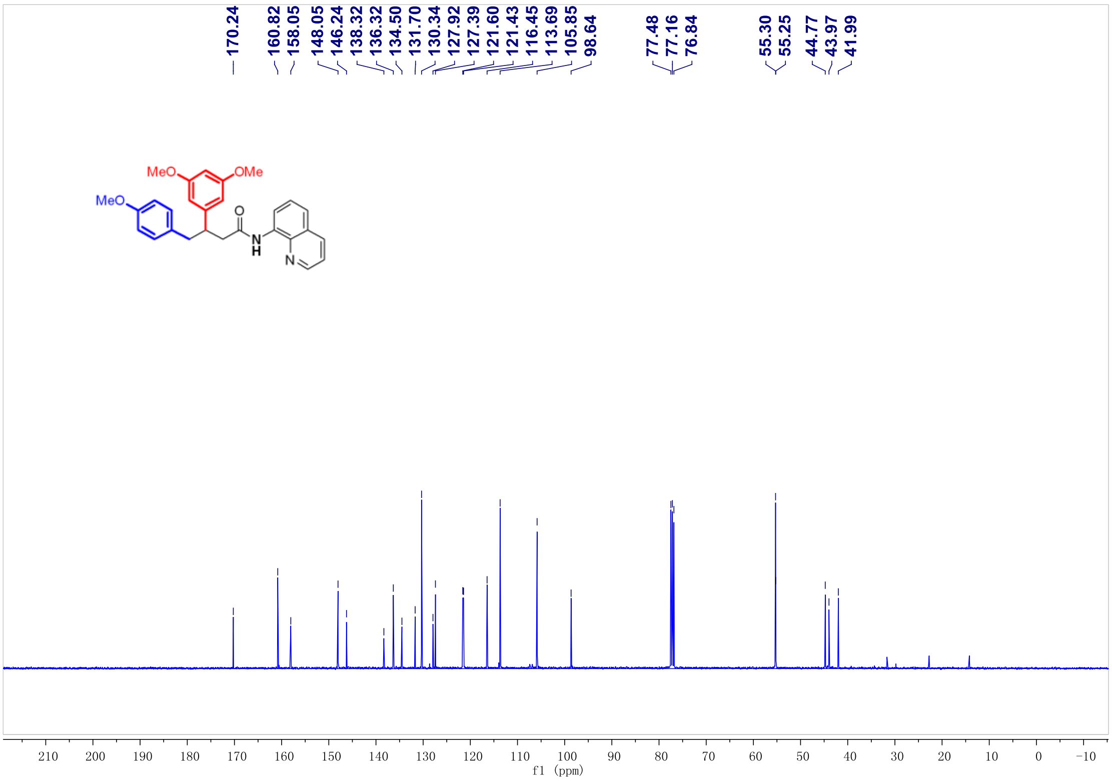
170.24
160.82
158.05
148.05
146.24
138.32
136.32
134.50
131.70
130.34
127.92
127.39
121.60
121.43
116.45
113.69
105.85
98.64
77.48
77.16
76.84
55.30
55.25
44.77
43.97
41.99
MeO
OMe
MeO
O
N
H
N
f1 (ppm)

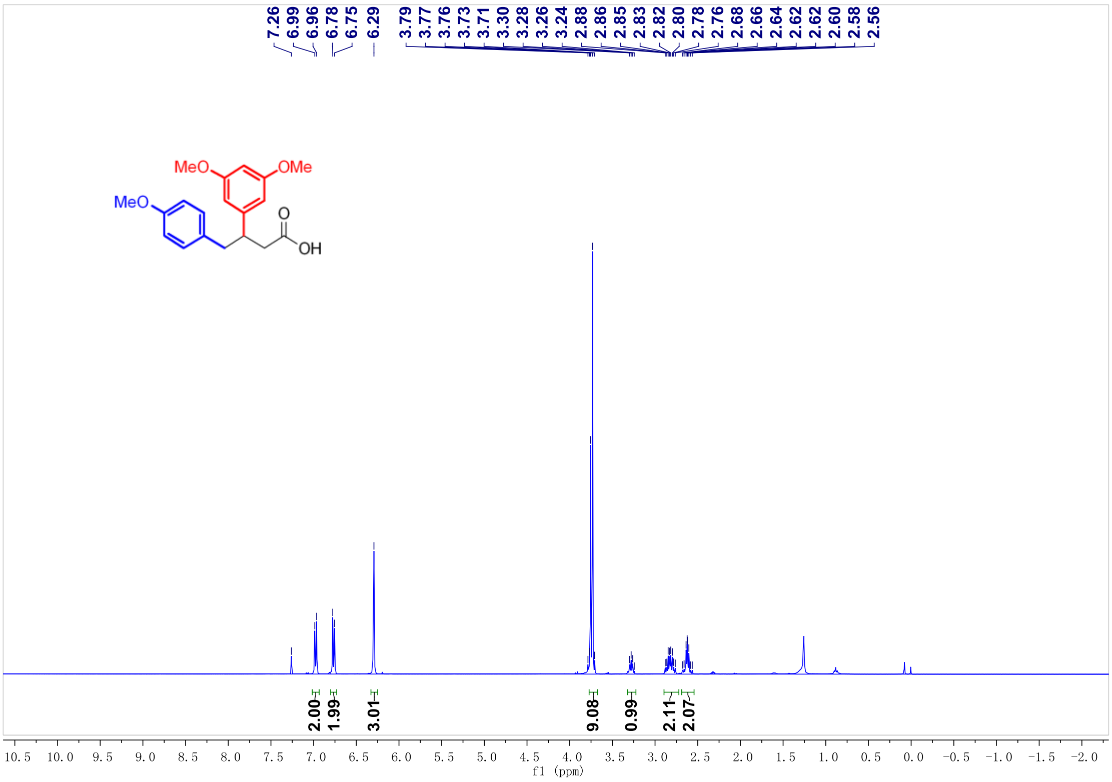

6. Control Experiments & Mechanistic Experiments

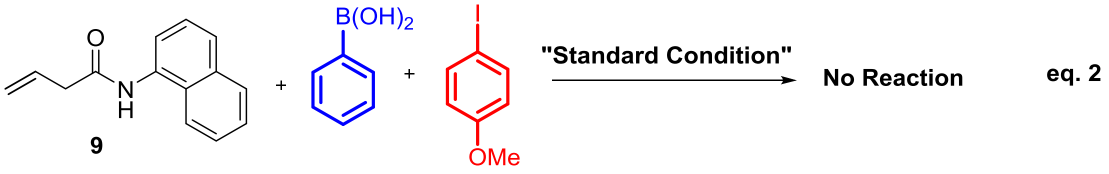

Following the general procedure, the reaction was carried out with **9** (0.3 mmol, 1.0 equiv), phenylboronic acid **2a** (0.6 mmol, 2.0 equiv), 4-methoxyphenyl iodide **3a**, Pd[(-)-sparteine]Cl_2 (0.03 mmol, 10 mol%), (-)-sparteine (0.06 mmol, 20 mol%), Na_2CO_3 (31.8 mg, 0.3 mmol) in the mixture of DCM/H_2O/CH_3CN (2 mL:0.4 mL:0.2 mL) for 24 h at 100 ℃ under a N_2 atmosphere. The reaction was monitored by TLC plate and detected by ^{1}H NMR, while no any product has been found in the reaction mixture.

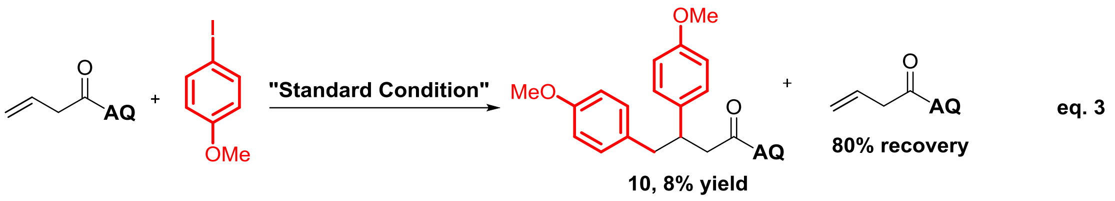

Following the general procedure, the reaction was carried out with **1a** (0.2 mmol, 1.0 equiv), 4-methoxyphenyl iodide **3a** (0.8 mmol, 4.0 equiv), Pd[(-)-sparteine]Cl_2 (0.02 mmol, 10 mol%), (-)-sparteine (0.04 mmol, 20 mol%), Na_2CO_3 (21.6 mg, 0.2 mmol) in the mixture of DCM/H_2O/CH_3CN (2 mL:0.4 mL:0.2 mL) for 24 h at 100 ℃ under a N_2 atmosphere. 3,4-Bis(4-methoxyphenyl)-N-(quinolin-8-yl)butanamide (**10**) was isolated by column chromatography (PE/acetone = 9/1) as a white solid in 8 % yield. 80% of **1a** was recovered. **^{1}H NMR (400 MHz, $CDCl_3$)**: δ 9.68 (s, 1H), 8.75– 8.71 (m, 2H), 8.09 (dd, *J* = 8.3, 1.6 Hz, 1H), 7.50 – 7.42 (m, 2H), 7.39 (dd, *J* = 8.3, 4.2 Hz, 1H), 7.16 (t, *J* = 5.7 Hz, 2H), 7.01 (d, *J* = 8.6 Hz, 2H), 6.82 – 6.73 (m, 4H), 3.72 (s, 3H), 3.71 (s, 3H), 3.63 – 3.52 (m, 1H), 3.04 – 2.79 (m, 4H). **^{13}C NMR (101 MHz, $CDCl_3$)**: δ 170.34, 158.12, 157.91, 147.99, 138.21, 136.31, 135.68, 134.42, 131.80, 130.30, 128.62, 127.86, 127.35, 121.54, 121.38, 116.44, 113.85, 113.58, 55.15, 55.13, 44.24, 43.61, 42.25. **HRMS (ESI)** m/z Calcd. for $C_{27}H_{26}N_2O_3$ $[M+H]^+$ 427.2016, Found 427.2020.

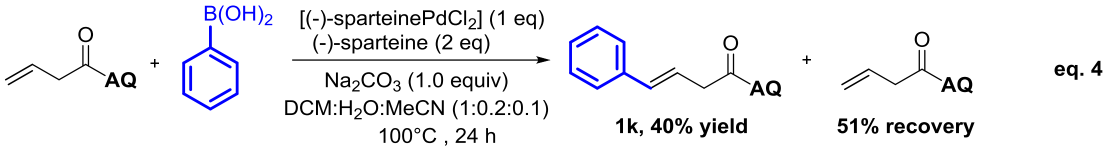

Following the general procedure, the reaction was carried out with **1a** (0.2 mmol, 1.0 equiv), phenylboronic acid **2a** (0.4 mmol, 2.0 equiv), Pd[(-)-sparteine]Cl_2 (0.2 mmol, 1.0 equiv), (-)-sparteine (0.4 mmol, 2.0 equiv), Na_2CO_3 (21.2 mg, 0.2 mmol) in the mixture of DCM/H_2O/CH_3CN (2 mL:0.4 mL:0.2 mL) for 24 h at 100 ℃ under a N_2 atmosphere. The Heck product (*E*)-4-phenyl-N-(quinolin-8-yl)but-3-enamide (**1k**) was isolated by column chromatography (PE/acetone = 9/1) as a white solid in 40 % yield. 51% of **1a** was recovered.

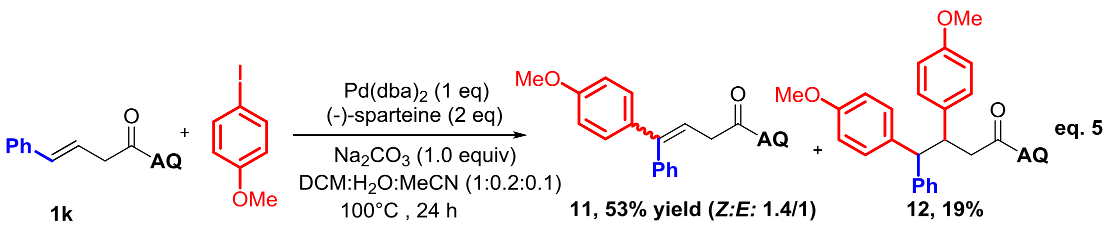

Following the general procedure, the reaction was carried out with **1k** (0.2 mmol, 1.0 equiv), 4-methoxyphenyl iodide **3a** (0.8 mmol, 4.0 equiv), Pd(dba)$_2$ (0.2 mmol, 1.0 equiv), (-)-sparteine (0.4 mmol, 2.0 equiv), Na_2CO_3 (21.2 mg, 0.2 mmol) in the mixture of DCM/H_2O/CH_3CN (2 mL:0.4 mL:0.2 mL) for 24 h at 100 ℃ under a N_2 atmosphere. 4-(4-Methoxyphenyl)-4-phenyl-N-(quinolin-8-yl)but-3-enamide (**11**) was isolated by column chromatography (PE/acetone = 9/1) as a mixture of *Z/E*-products in 53% yield (*Z/E* 1.4:1). The spectroscopic analyses were in agreement with the literature.[14] **^{1}H NMR (400 MHz, CDCl$_3$):** δ 10.15 (s, 1H), 8.86 – 8.65 (m, 2.23H), 8.20 (d, *J* = 8.2 Hz, 1H), 7.60 – 7.51 (m, 2.24H), 7.47 (dd, *J* = 8.1, 4.1 Hz, 1.16H), 7.43 – 7.36 (m, 2.11H), 7.36 – 7.28 (m, 4.11H), 7.25 – 7.23 (m, 1.1H), 6.96 – 6.90 (m, 0.86H), 6.88 – 6.82 (m, 1.25H), 6.41 – 6.34 (m, 1H), 3.83 (s, 1.19H), 3.81 (s, 1.67H), 3.47 (d, *J* = 7.7 Hz, 0.81H), 3.41 (d, *J* = 7.7 Hz, 1.13H). **HRMS (ESI)** m/z Calcd. for $C_{26}H_{22}N_2O_2$ $[M+H]^+$ 395.1754, Found 395.1758.

3,4-Bis(4-methoxyphenyl)-4-phenyl-N-(quinolin-8-yl)butanamide (**12**) was isolated by column chromatography (PE/acetone = 9/1) as a white solid in 19% yield. **^{1}H NMR (400 MHz, CDCl$_3$):** δ 9.41 (s, 1H), 8.70 (dd, *J* = 4.2, 1.3 Hz, 1H), 8.62 (dd, *J* = 7.0, 1.7 Hz, 1H), 8.08 (dd, *J* = 8.2, 1.2 Hz, 1H), 7.47 (d, *J* = 7.5 Hz, 2H), 7.43 (t, *J* = 4.8 Hz, 2H), 7.38 (dd, *J* = 8.4, 4.3 Hz, 1H), 7.31 (t, *J* = 7.6 Hz, 2H), 7.17 (t, *J* = 7.8 Hz, 3H), 7.09 (d, *J* = 8.6 Hz, 2H), 6.69 – 6.57 (m, 4H), 4.18 (d, *J* = 2.9 Hz, 2H), 3.66 (s, 3H), 3.61 (s, 3H), 2.95 – 2.87 (m, 1H), 2.75 – 2.67 (m, 1H). **^{13}C NMR (101 MHz, CDCl$_3$):** δ 170.42, 157.86, 157.62, 147.91, 143.90, 138.16, 136.41, 135.49, 134.57, 134.45, 129.31, 129.26, 128.99, 128.54, 128.31, 127.91, 127.45, 126.61, 121.54, 121.34, 113.74, 113.63, 57.29, 55.15, 55.07, 45.96, 44.39. **HRMS (ESI)** m/z Calcd. for $C_{33}H_{30}N_2O_3$ $[M+H]^+$ 503.2329, Found 503.2333.

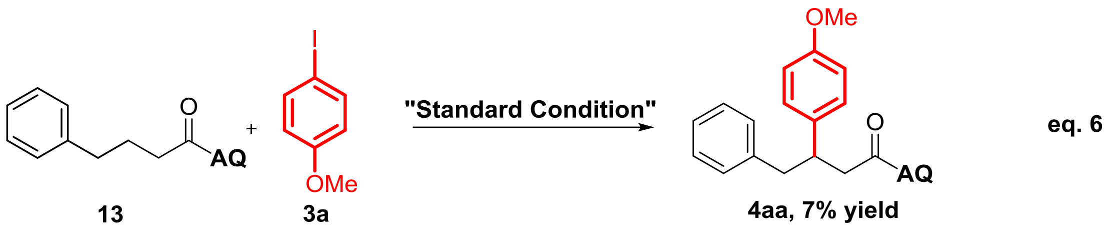

Following the general procedure, the reaction was carried out with **13** (0.3 mmol, 1.0 equiv), 4-methoxyphenyl iodide **3a** (0.9 mmol, 3.0 equiv), Pd[(-)-sparteine]Cl_2 (0.03 mmol, 10 mol%), (-)-sparteine (0.06 mmol, 20 mol%), Na_2CO_3 (31.8 mg, 0.3 mmol) in the mixture of DCM/H_2O/CH_3CN (2 mL:0.4 mL:0.2 mL) for 24 h at 100 ℃ under a N_2 atmosphere. 3-(4-Methoxyphenyl)-4-phenyl-*N*-(quinolin-8-yl)butanamide (**4aa**) was determined by ^{1}H NMR using CH_2Br_2 as internal standard in 7 % yield.

7. X-Ray Crystallography Data

X-ray Crystal Structure Data for Compound 5f

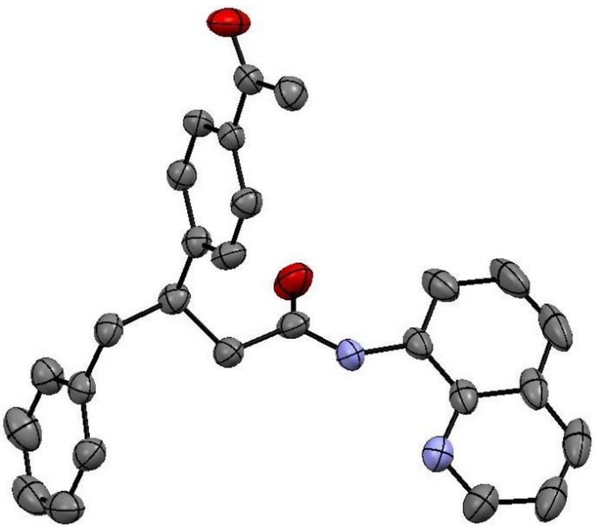

CCDC: 1852875

Figure S4. ORTEP plot of compound **4ah**. All H atoms have been omitted for clarity.

Identification code: **4ah**; Empirical formula: $C_{27}H_{24}N_2O_2$; Formula weight 408.48; Temperature: 113(2) K; Wavelength: 0.71073 A; Crystal system, space group: Monoclinic, P2(1)/c; Unit cell dimensions: a = 10.452(2) A alpha = 90 deg. b = 5.5211(11) A beta = 96.31(3) deg. c = 37.598(8) A gamma = 90 deg. Volume: 2156.5(8) A^3 Z, Calculated density 4, 1.258 Mg/m^3: Absorption coefficient

0.080 mm^-1: F(000): 864; Crystal size: 0.200 x 0.180 x 0.120 mm; Theta range for data collection: 1.090 to 25.020 deg.; Limiting indices: -12<=h<=12, -6<=k<=6, -44<=l<=44; Reflections collected / unique: 15043 / 3793 [R(int) = 0.0470] ; Completeness to theta = 25.020 99.5 %; Absorption correction Semi-empirical from equivalents; Max. and min. transmission: 1.0000 and 0.9079; Refinement method: Full-matrix least-squares on F^2; Data / restraints / parameters 3793 / 1 / 285; Goodness-of-fit on F^2 1.076; Final R indices: [I>2sigma(I)] R1 = 0.0612, wR2 = 0.1501; R indices (all data): R1 = 0.0736, wR2 = 0.1627; Extinction coefficient: 0.016(2); Largest diff. peak and hole: 0.330 and -0.191 e.A^-3

X-ray Crystal Structure Data for Compound 5m

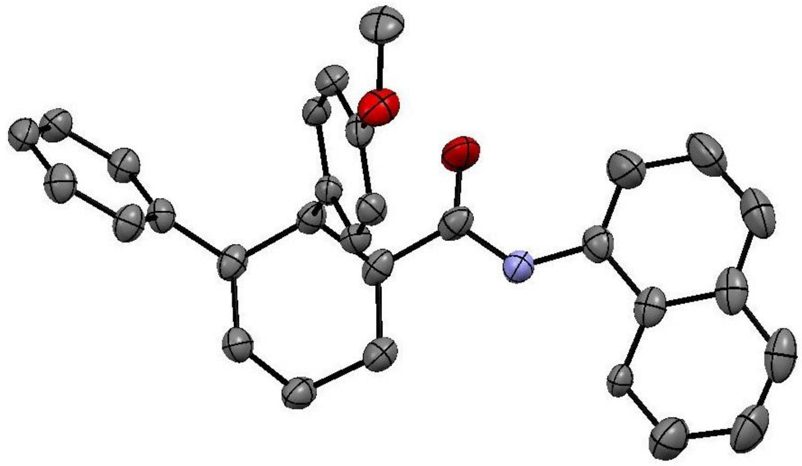

CCDC: 1852877

Figure S5. ORTEP plot of compound **5m**. All H atoms have been omitted for clarity.

Identification code: **5m**; Empirical formula: C30H29NO2; Formula weight: 435.54; Temperature: 113(2) K; Wavelength: 0.71073 A; Crystal system, space group Orthorhombic, Fdd2; Unit cell dimensions: a = 20.542(4) A alpha = 90 deg. b = 74.483(15) A beta = 90 deg. c = 5.9207(12) A gamma = 90 deg. Volume: 9059(3) A^3; Z, Calculated density: 16, 1.277 Mg/m^3; Absorption coefficient: 0.079 mm^-1; F(000): 3712; Crystal size: 0.200 x 0.180 x 0.120 mm; Theta range for data collection: 1.094 to 27.861 deg. Limiting indices: -26<=h<=26, -96<=k<=97, -7<=l<=7; Reflections collected / unique: 19378 / 5360 [R(int) = 0.0516];

Completeness to theta = 25.242 99.7 %; Absorption correction: Semi-empirical from equivalents; Max. and min. transmission: 1.0000 and 0.7278; Refinement method: Full-matrix least-squares on F^2; Data / restraints / parameters: 5360 / 2 / 305; Goodness-of-fit on F^2: 1.047; Final R indices [I>2sigma(I)]: R1 = 0.0487, wR2 = 0.1187; R indices (all data): R1 = 0.0580, wR2 = 0.1307; Absolute structure parameter: -1(2); Extinction coefficient: 0.0032(3); Largest diff. peak and hole: 0.358 and -0.421 e.A^-3

8. References

1. K. S. Yang, J. A. Jr. Gurak, Z. Liu and K. M. Engle, *J. Am. Chem. Soc.,* 2016, **138**, 14705.
2. Y. Kawamata, T. Hashimoto and K. Maruoka, *J. Am. Chem. Soc.*, 2016, **138**, 5206.
3. Y. Kawamata, T. Hashimoto, K. Maruoka, *J. Am. Chem. Soc.,* 2016, **138**, 165206.
4. J. A. Jr. Gurak, K. S. Yang, Z. Liu and K. M. Engle, *J. Am. Chem. Soc.*, 2016, **138**, 5805.
5. E. P. A. Talbot, T. de A. Fernandes, J. M. McKenna and F. D. Toste, *J. Am. Chem. Soc.*, 2014, **136**, 4101.
6. A. Dey, S. Pimparkar, A. De, S. Guin, D. Maiti, *Adv. Synth. Catal.*, 2017, **359**, 1301.
7. S.-Y. Zhang, Q. Li, G. He, W. A. Nack, G. Chen, *J.Am.Chem.Soc.*, 2013, **135**, 12135.
8. K. Kamata, I. Agata and A. I. Meyers, *J. Org. Chem.*, 1998, **63**, 3113.
9. A. M. Francisco, C. S. Davi, P. W. Edison, L. W. Celso, B. C. Quezia and B. Fabiana, *Lett. Org. Chem.*, 2007, **4**, 155.
10. T. Hvidt, W. A. Szarek and D. B. Maclean, *Can. J. Chem*., 1988, **66**, 779.
11. P. J. Murray, M. Kranz, M. Ladlow, S. Taylor, F. Berst, A. B. Holmes, K. N. Keavey, A. Jaxa-Chamiec, P. W. Seale, P. Stead, R. J. Upton, S. L. Croft, W. Clegg and M. R. Elsegood, *Bioorg. Med. Chem. Lett*., 2001, **11**, 773.

12. J. C. Antilla, A. Klapars and S. L. Buchwald, *J. Am. Chem. Soc.*, 2002, **124**, 11684.

13. F. Klotter and A. Studer, *Angew. Chem. Int. Ed.*, 2014, **53**, 2473.

14. C. Romano, C. Mazet, *J. Am. Chem. Soc.*, 2018, **140**, 4743.

9. HPLC Trace and NMR Spectra

Racemic Sample

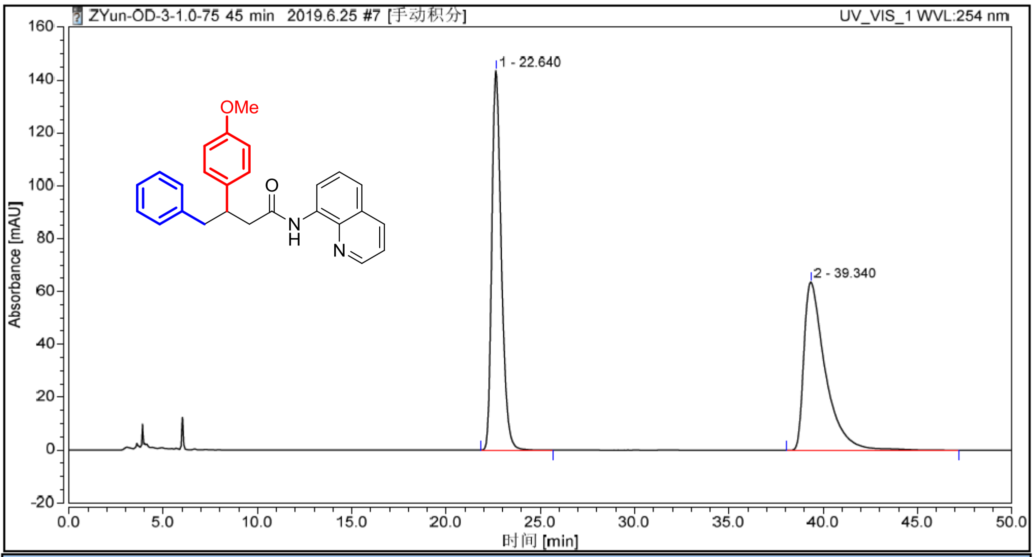

Result

Peak #	Run time [min][	Area [mAU*min]	Height [mAU]	Area %	Height %
1	22.640	83.921	143.655	49.81	69.25
2	39.340	84.552	63.795	50.19	30.75
Totals:		**168.473**	**207.450**	**100.00**	**100.00**

chiral 4a

Chromatogram

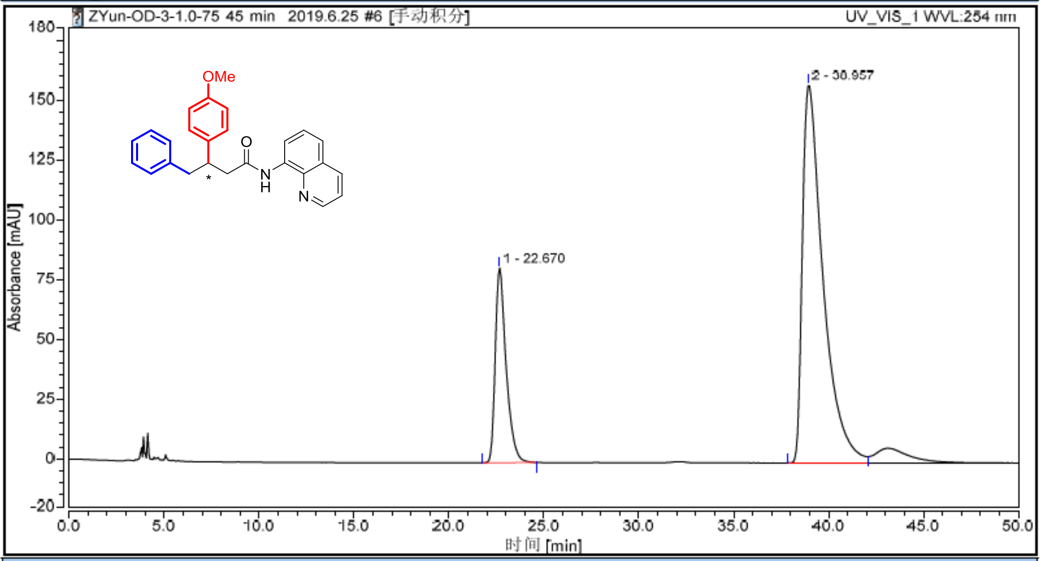

Result

Peak #	Run time [min][	Area [mAU*min]	Height [mAU]	Area %	Height %
1	22.670	53.989	81.571	21.32	34.02
2	38.957	199.275	158.215	78.68	65.98
Totals:		**253.264**	**239.786**	**100.00**	**100.00**

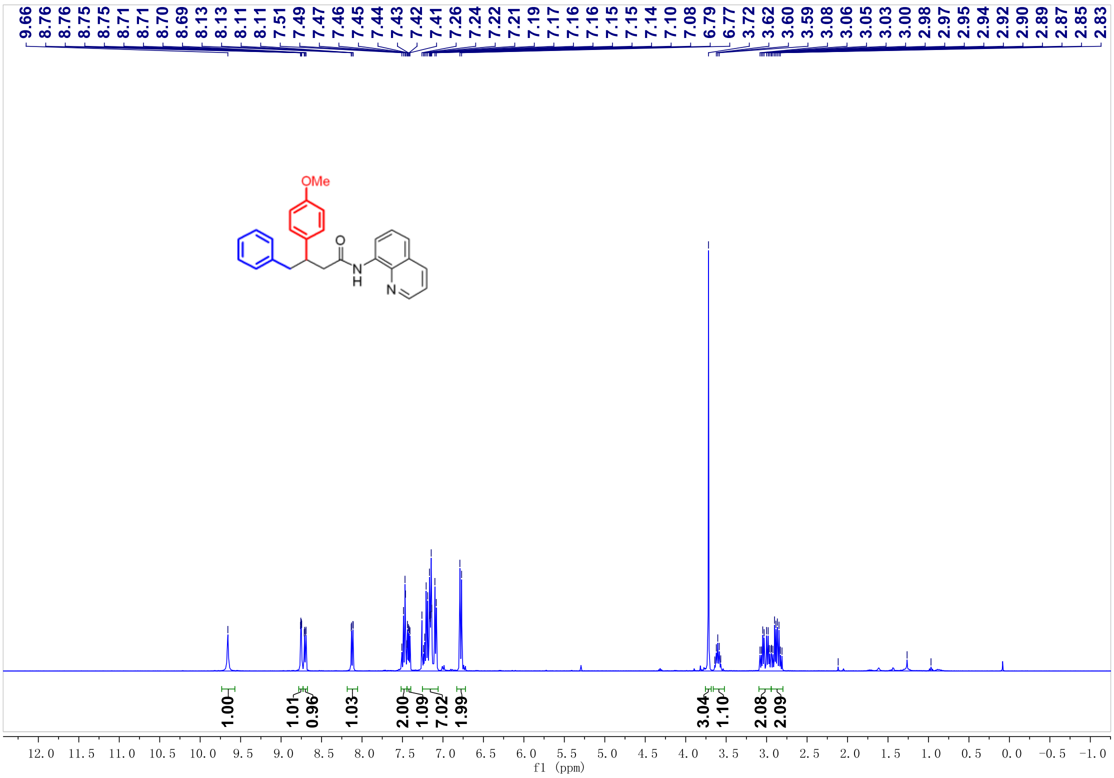

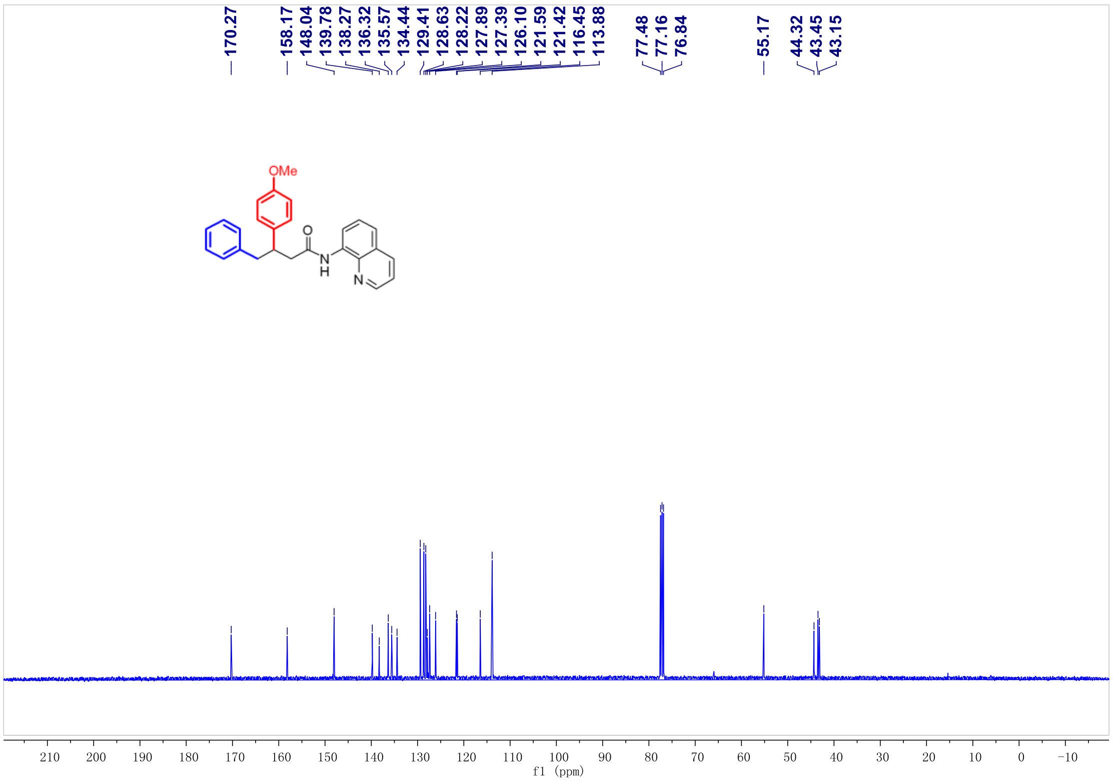

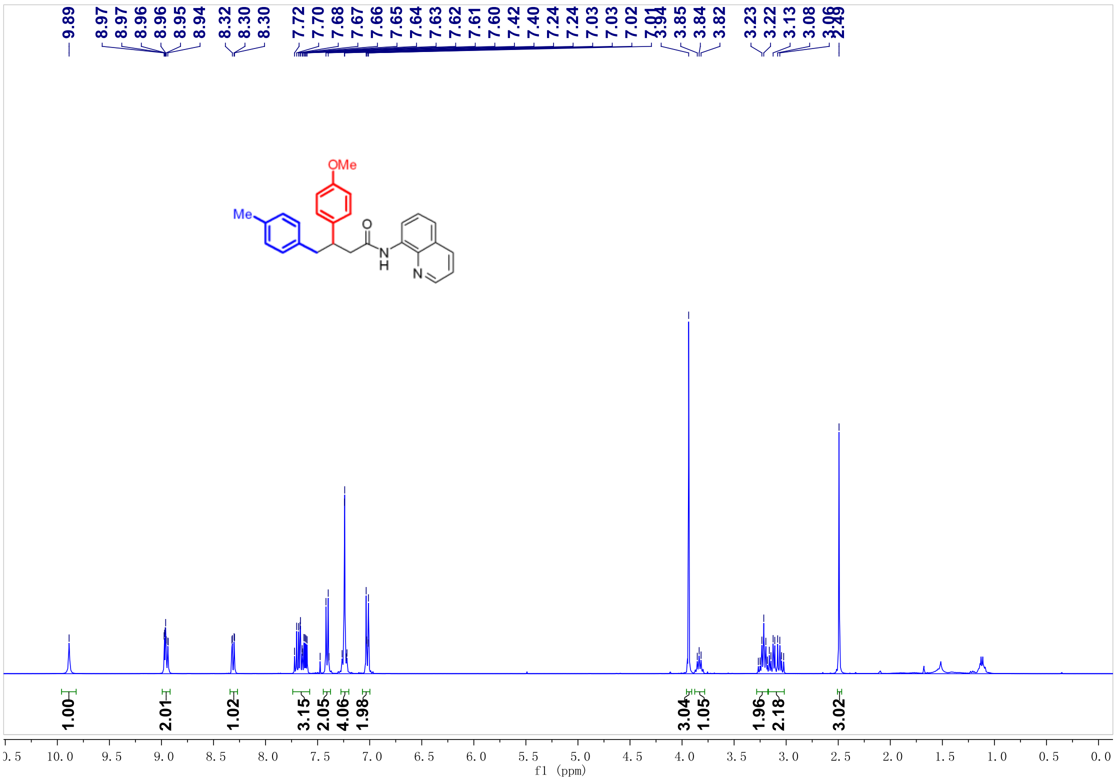
9.89
8.97
8.97
8.96
8.96
8.95
8.94
8.32
8.30
8.30
7.72
7.70
7.68
7.67
7.66
7.65
7.64
7.63
7.62
7.61
7.60
7.42
7.40
7.24
7.24
7.03
7.03
7.02
7.01
3.94
3.85
3.84
3.82
3.23
3.22
3.13
3.08
3.06
2.49
OMe
Me
O
N
H
N
1.00
2.01
1.02
3.15
2.05
4.06
1.98
3.04
1.05
1.96
2.18
3.02
f1 (ppm)

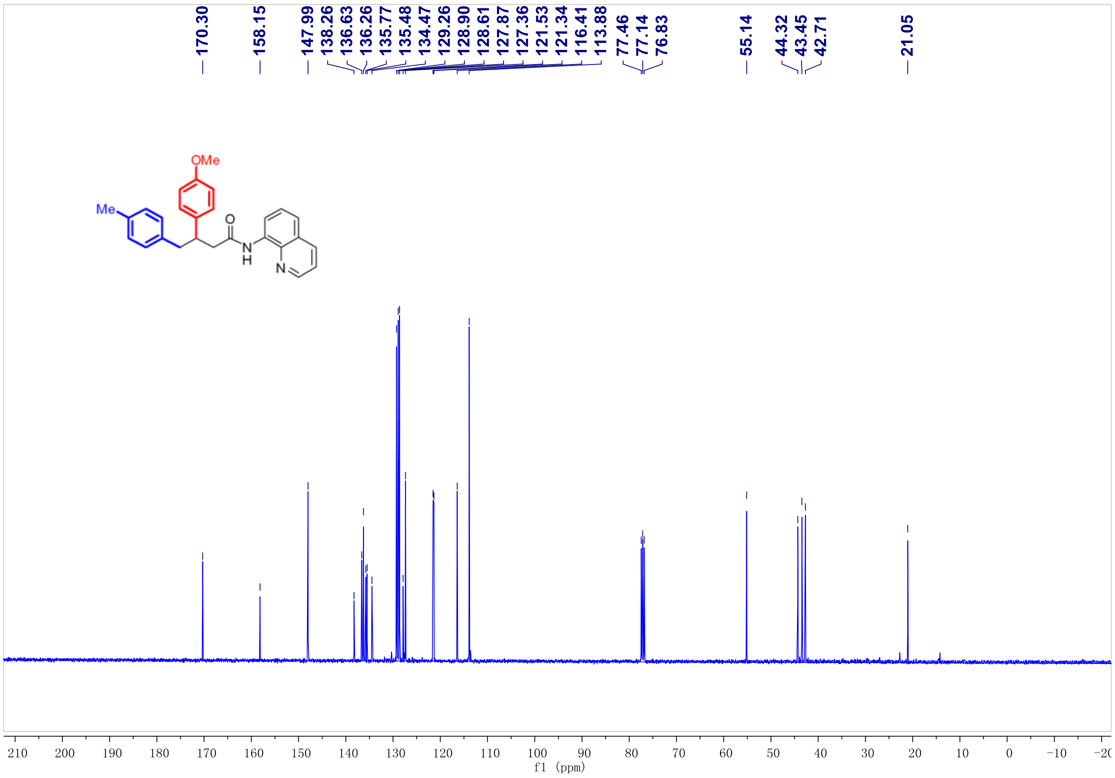
170.30
158.15
147.99
138.26
136.63
136.26
135.77
135.48
134.47
129.26
128.90
128.61
127.87
127.36
121.53
121.34
116.41
113.88
77.46
77.14
76.83
55.14
44.32
43.45
42.71
21.05
OMe
Me
O
N
H
N
f1 (ppm)

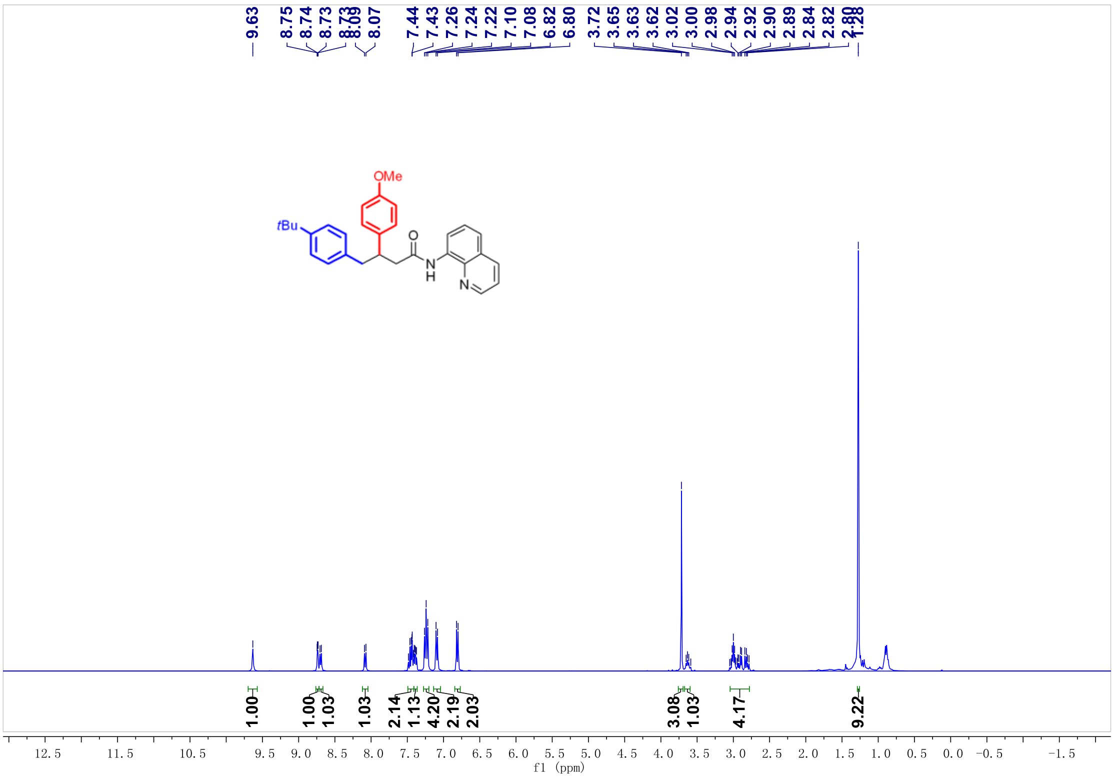
9.63
8.75
8.74
8.73
8.73
8.09
8.07
7.44
7.43
7.26
7.24
7.22
7.10
7.08
6.82
6.80
3.72
3.65
3.63
3.62
3.02
3.00
2.98
2.94
2.92
2.90
2.89
2.84
2.82
2.80
1.28
OMe
tBu
O
N
H
N
1.00
1.00
1.03
1.03
2.14
1.13
4.20
2.19
2.03
3.08
1.03
4.17
9.22
f1 (ppm)

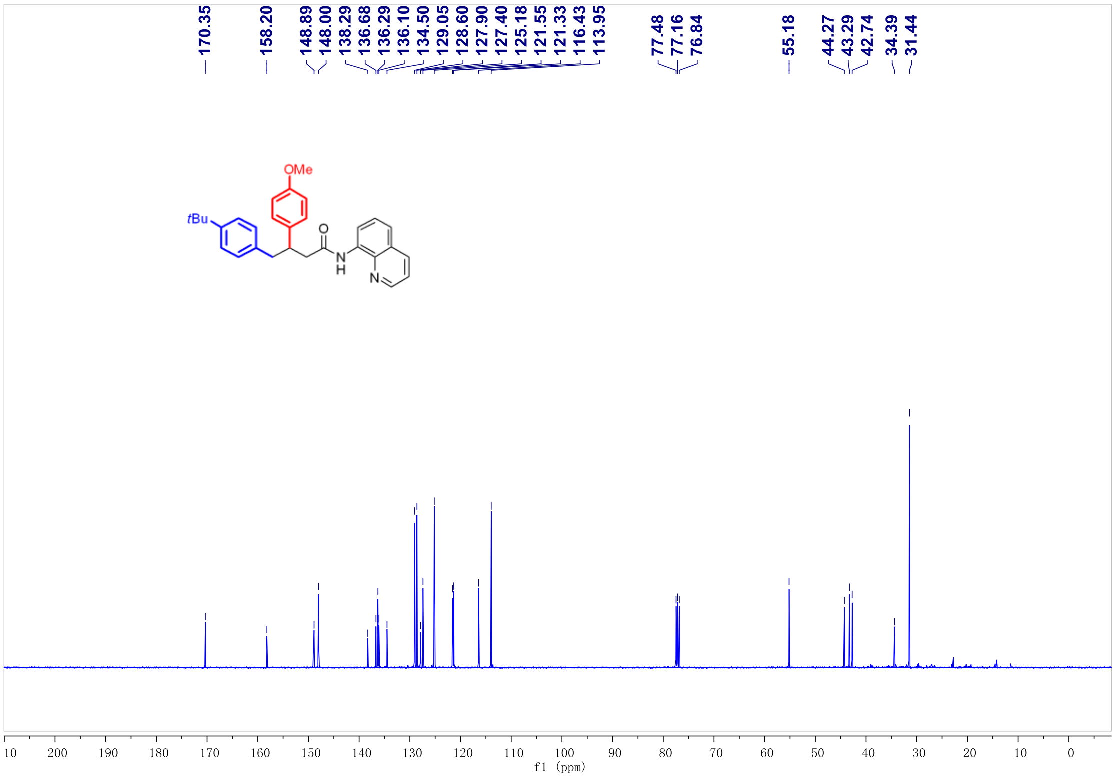
170.35
158.20
148.89
148.00
138.29
136.68
136.29
136.10
134.50
129.05
128.60
127.90
127.40
125.18
121.55
121.33
116.43
113.95
77.48
77.16
76.84
55.18
44.27
43.29
42.74
34.39
31.44
OMe
tBu
O
N
H
N
f1 (ppm)

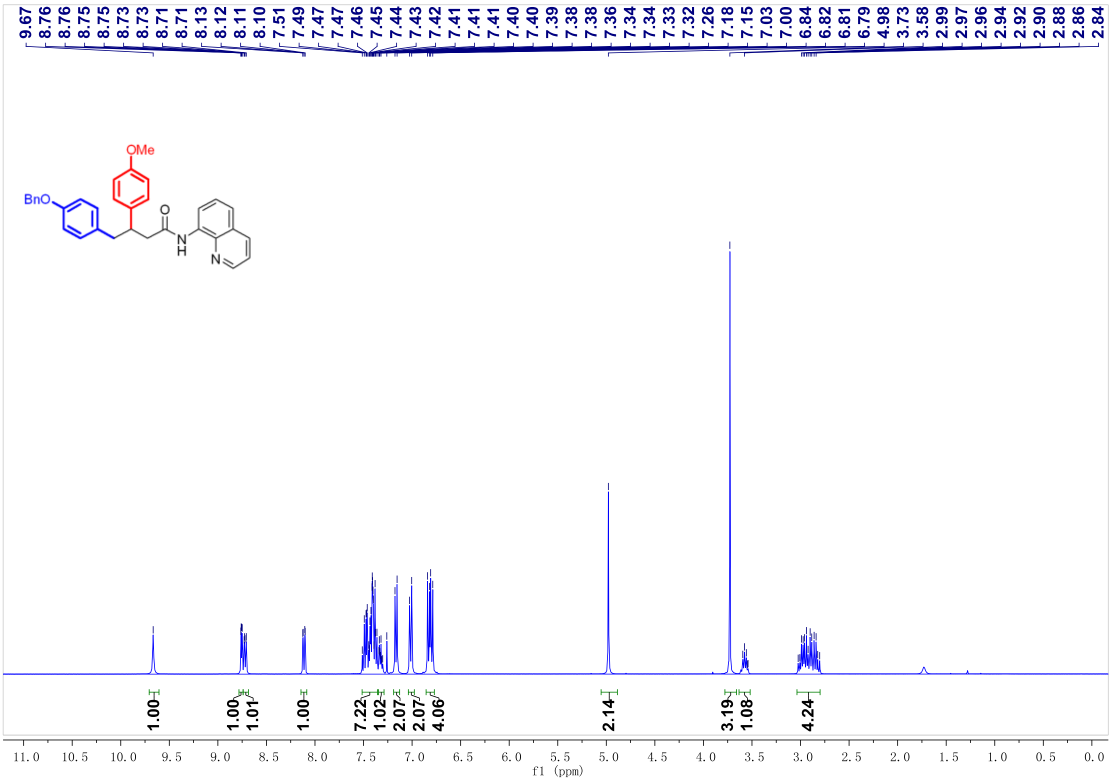
9.67
8.76
8.76
8.75
8.75
8.73
8.73
8.71
8.71
8.13
8.12
8.11
8.10
7.51
7.49
7.47
7.46
7.45
7.44
7.43
7.42
7.41
7.41
7.41
7.40
7.40
7.39
7.38
7.38
7.36
7.34
7.33
7.32
7.26
7.18
7.15
7.03
7.00
6.84
6.82
6.81
6.79
4.98
3.73
3.58
2.99
2.97
2.96
2.94
2.92
2.90
2.88
2.86
2.84
OMe
BnO
O
N
H
N
1.00
1.00
1.01
1.00
7.22
1.02
2.07
2.07
4.06
2.14
3.19
1.08
4.24
f1 (ppm)

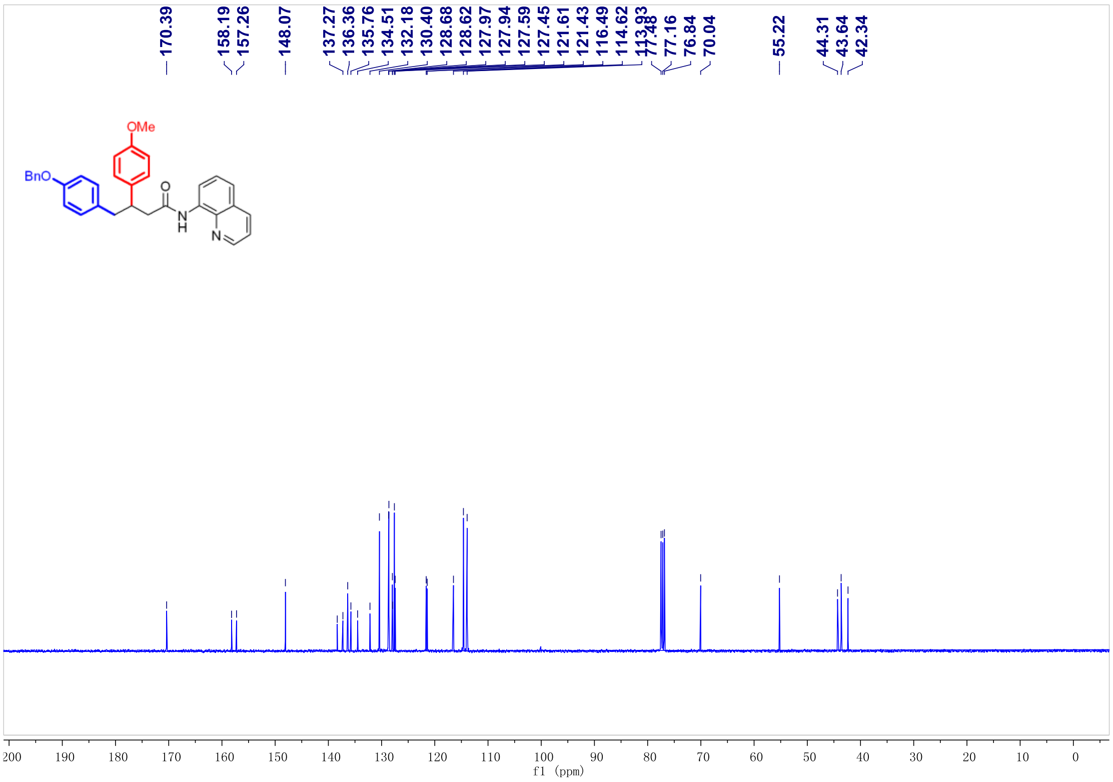
170.39
158.19
157.26
148.07
137.27
136.36
135.76
134.51
132.18
130.40
128.68
128.62
127.97
127.94
127.59
127.45
121.61
121.43
116.49
114.62
113.93
77.48
77.16
76.84
70.04
55.22
44.31
43.64
42.34
OMe
BnO
O
N
H
N
f1 (ppm)

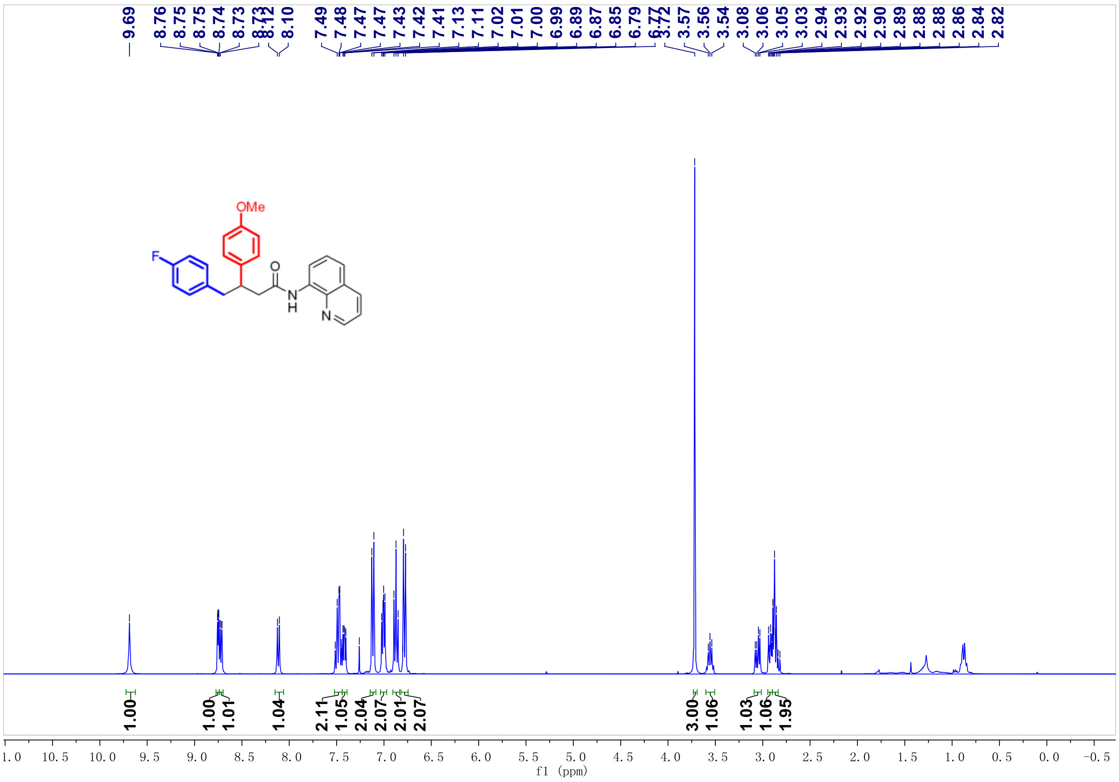

9.69
8.76
8.75
8.74
8.73
8.13
8.10
7.49
7.48
7.47
7.47
7.43
7.42
7.41
7.13
7.11
7.02
7.01
7.00
6.99
6.89
6.87
6.85
6.79
6.77
3.72
3.57
3.56
3.54
3.08
3.06
3.05
3.03
2.94
2.93
2.92
2.90
2.89
2.88
2.88
2.86
2.84
2.82
OMe
F
O
N
H
N
1.00
1.00
1.01
1.04
2.11
1.05
2.04
2.07
2.01
2.07
3.00
1.06
1.03
1.06
1.95
f1 (ppm)

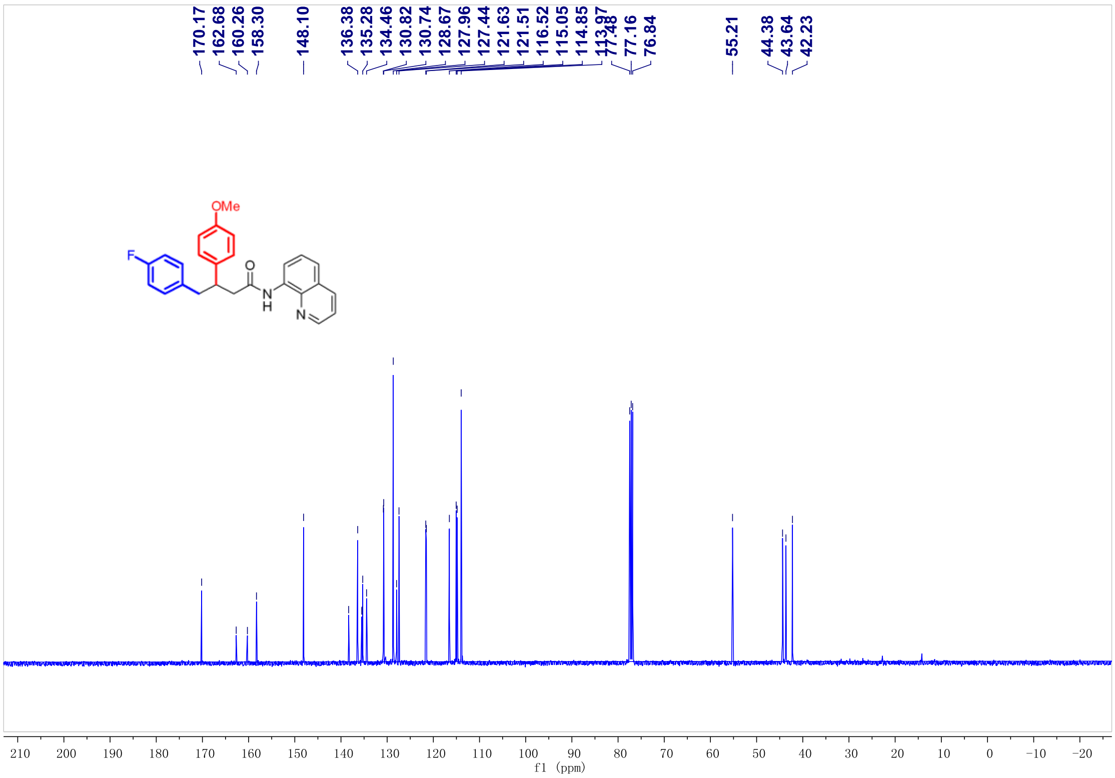

170.17
162.68
160.26
158.30
148.10
136.38
135.28
134.46
130.82
130.74
128.67
127.96
127.44
121.63
121.51
116.52
115.05
114.85
113.97
77.48
77.16
76.84
55.21
44.38
43.64
42.23
OMe
F
O
N
H
N
f1 (ppm)

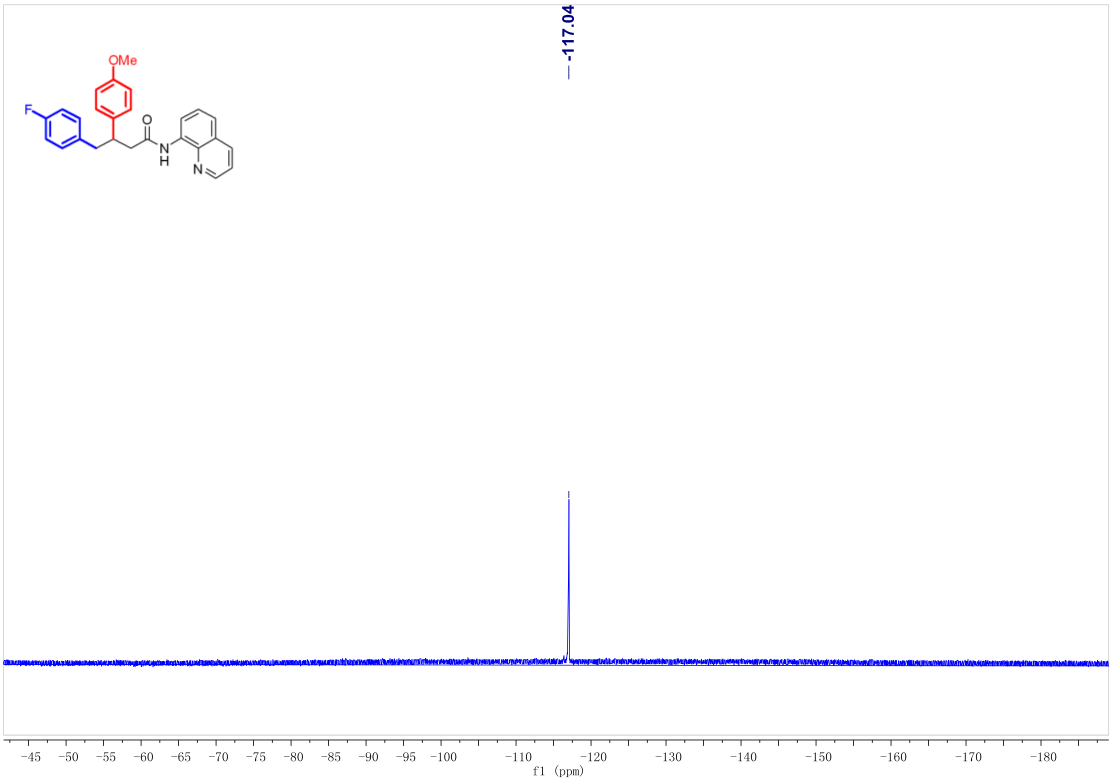

-117.04
OMe
F
O
N
H
N
-45 -50 -55 -60 -65 -70 -75 -80 -85 -90 -95 -100 -110 -120 -130 -140 -150 -160 -170 -180
f1 (ppm)

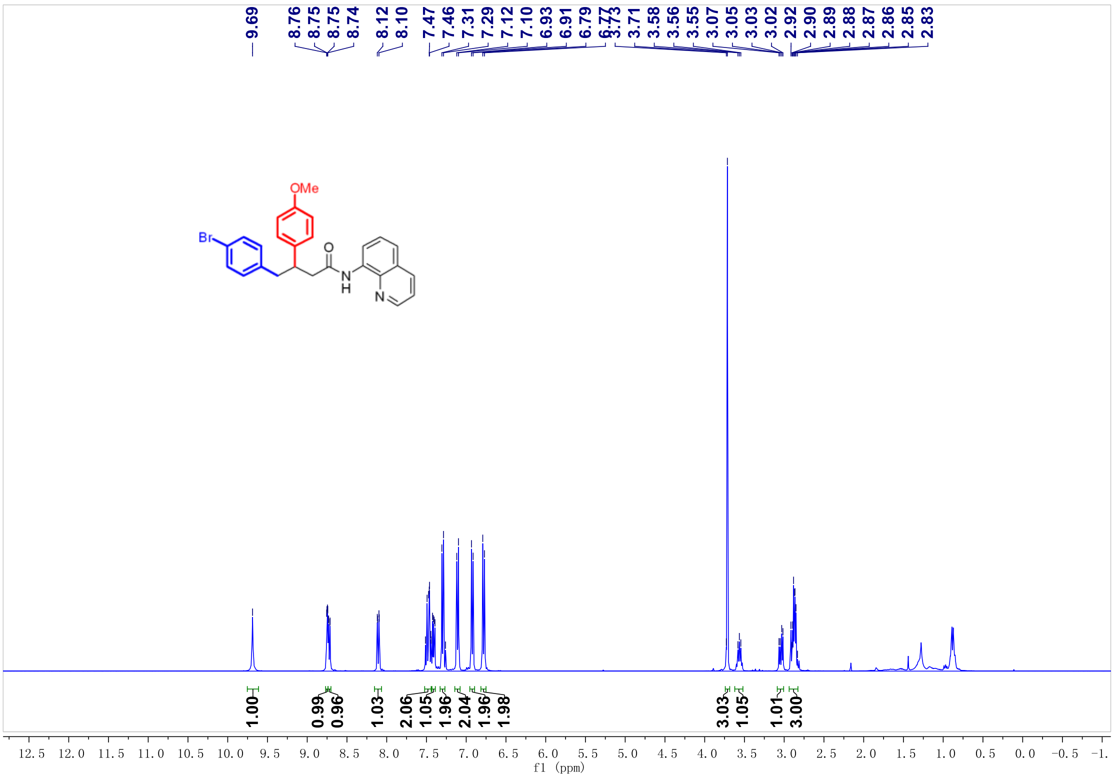

9.69
8.76 8.75 8.75 8.74
8.12 8.10
7.47 7.46 7.31 7.29 7.12 7.10 6.93 6.91 6.79 6.77
3.73 3.71 3.58 3.56 3.55 3.07 3.05 3.03 3.02 2.92 2.90 2.89 2.88 2.87 2.86 2.85 2.83
OMe
Br
O
N
H
N
1.00
0.99 0.96
1.03
2.06 1.05 1.96 2.04 1.96 1.98
3.03 1.05
1.01 3.00
12.5 12.0 11.5 11.0 10.5 10.0 9.5 9.0 8.5 8.0 7.5 7.0 6.5 6.0 5.5 5.0 4.5 4.0 3.5 3.0 2.5 2.0 1.5 1.0 0.5 0.0 -0.5 -1.
f1 (ppm)

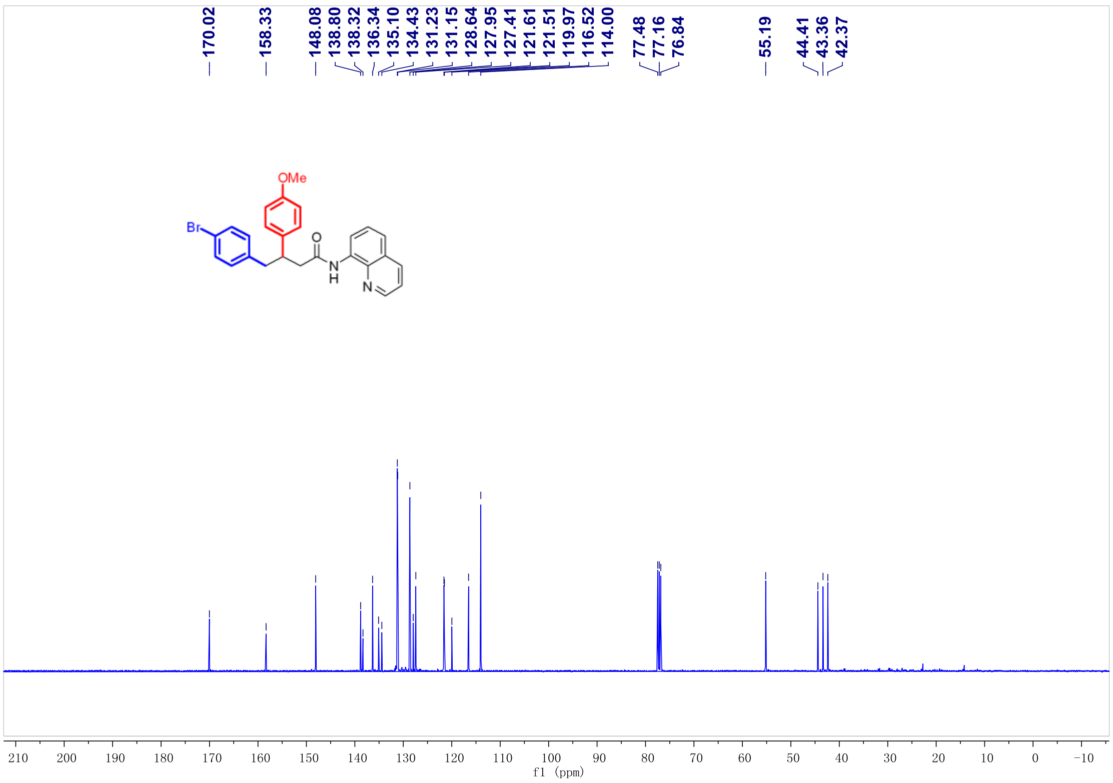
170.02
158.33
148.08
138.80
138.32
136.34
135.10
134.43
131.23
131.15
128.64
127.95
127.41
121.61
121.51
119.97
116.52
114.00
77.48
77.16
76.84
55.19
44.41
43.36
42.37
OMe
Br
O
N
H
N
f1 (ppm)

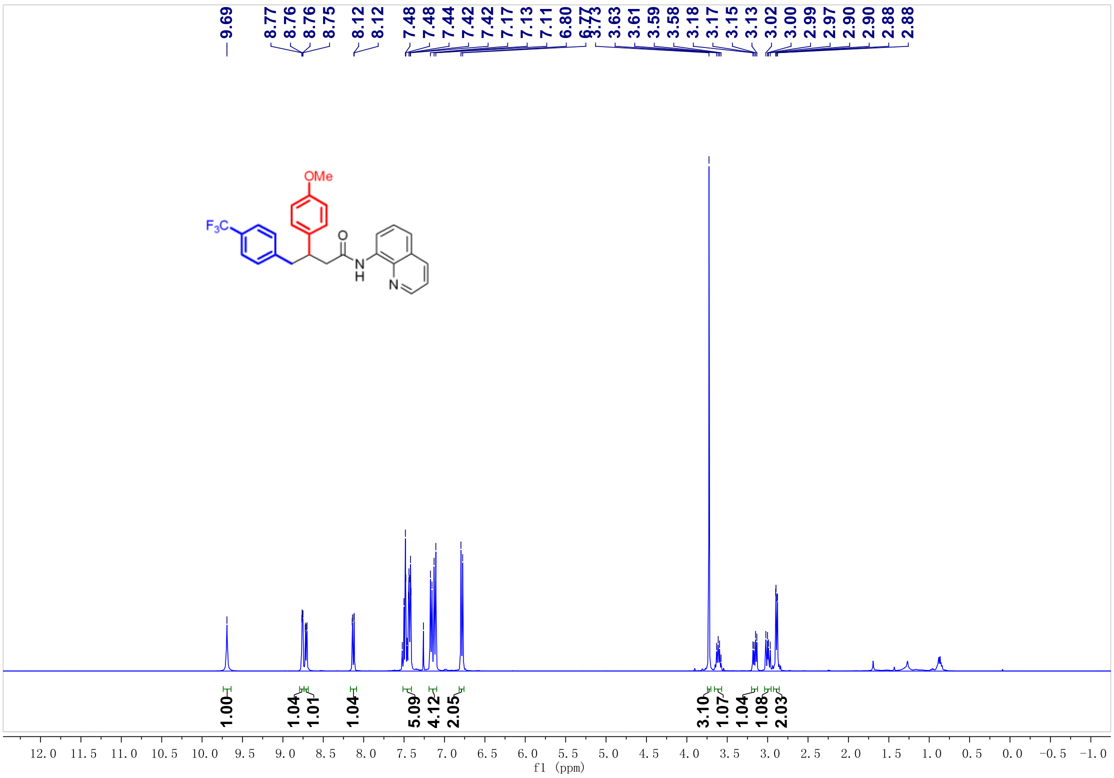
9.69
8.77
8.76
8.76
8.75
8.12
8.12
7.48
7.48
7.44
7.42
7.42
7.17
7.13
7.11
6.80
6.77
3.73
3.63
3.61
3.59
3.58
3.18
3.17
3.15
3.13
3.02
3.00
2.99
2.97
2.90
2.90
2.88
2.88
OMe
F3C
O
N
H
N
1.00
1.04
1.01
1.04
5.09
4.12
2.05
3.10
1.07
1.04
1.08
2.03
f1 (ppm)

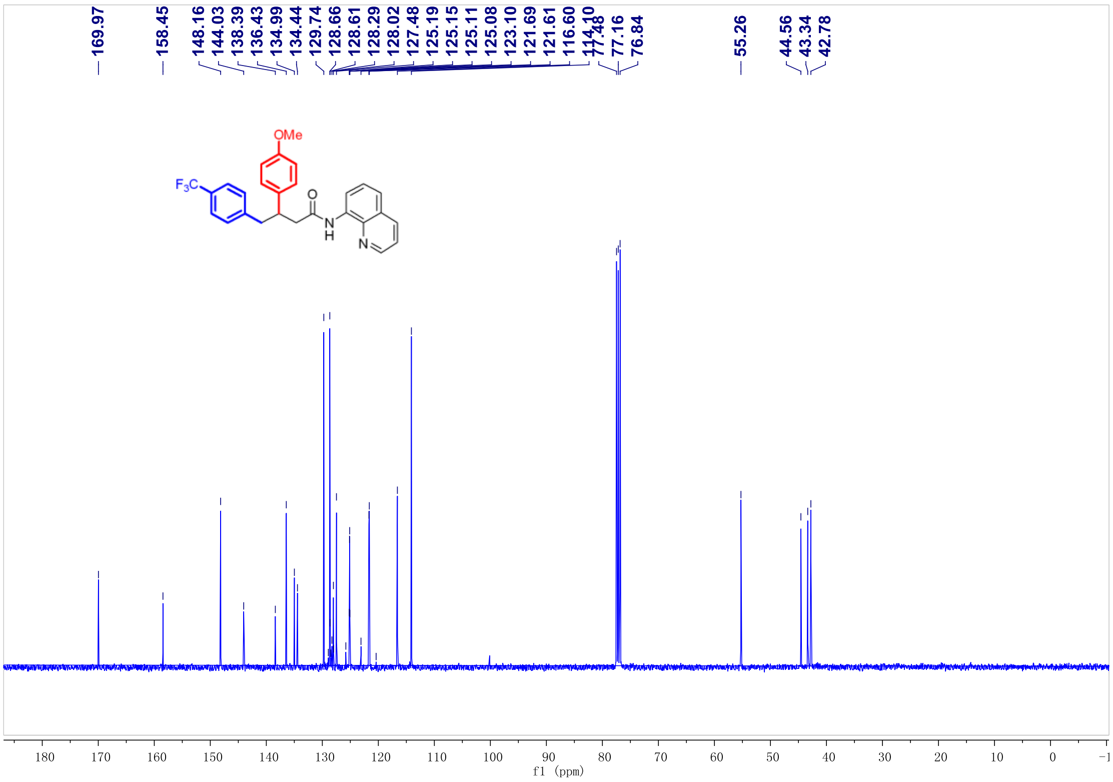
169.97
158.45
148.16
144.03
138.39
136.43
134.99
134.44
129.74
128.66
128.61
128.29
128.02
127.48
125.19
125.15
125.11
125.08
123.10
121.69
121.61
116.60
114.10
77.48
77.16
76.84
55.26
44.56
43.34
42.78
OMe
F3C
O
N
H
N
f1 (ppm)

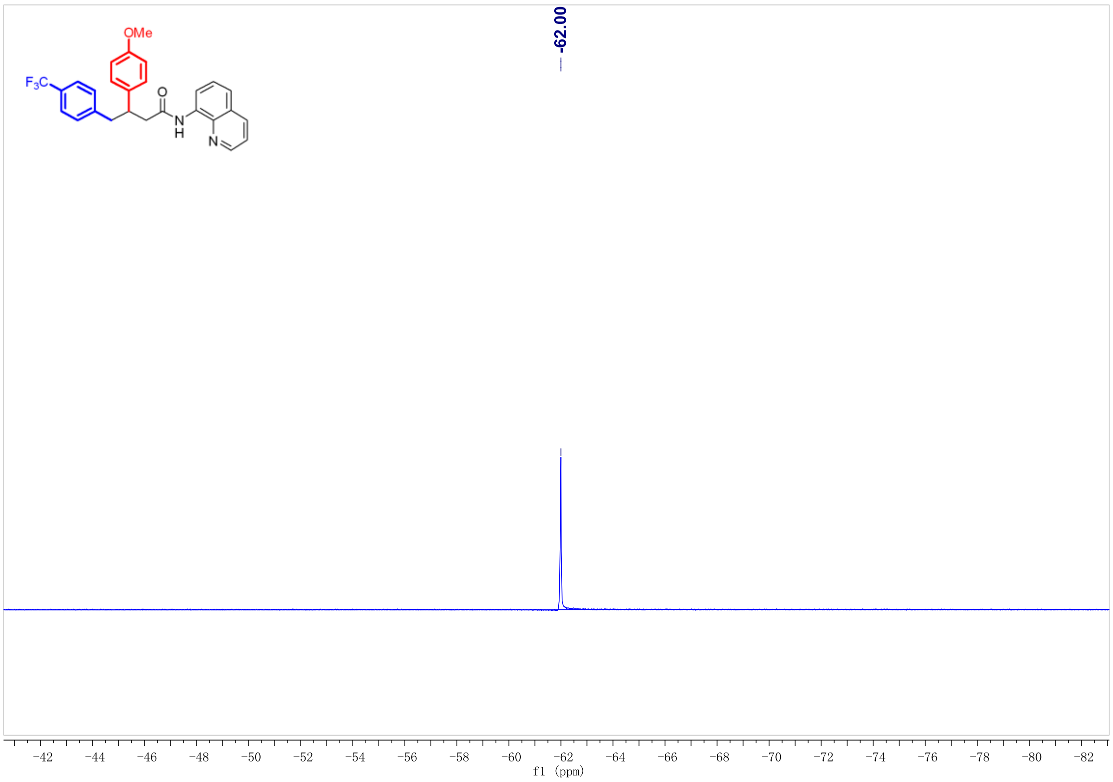
-62.00
OMe
F3C
O
N
H
N
f1 (ppm)

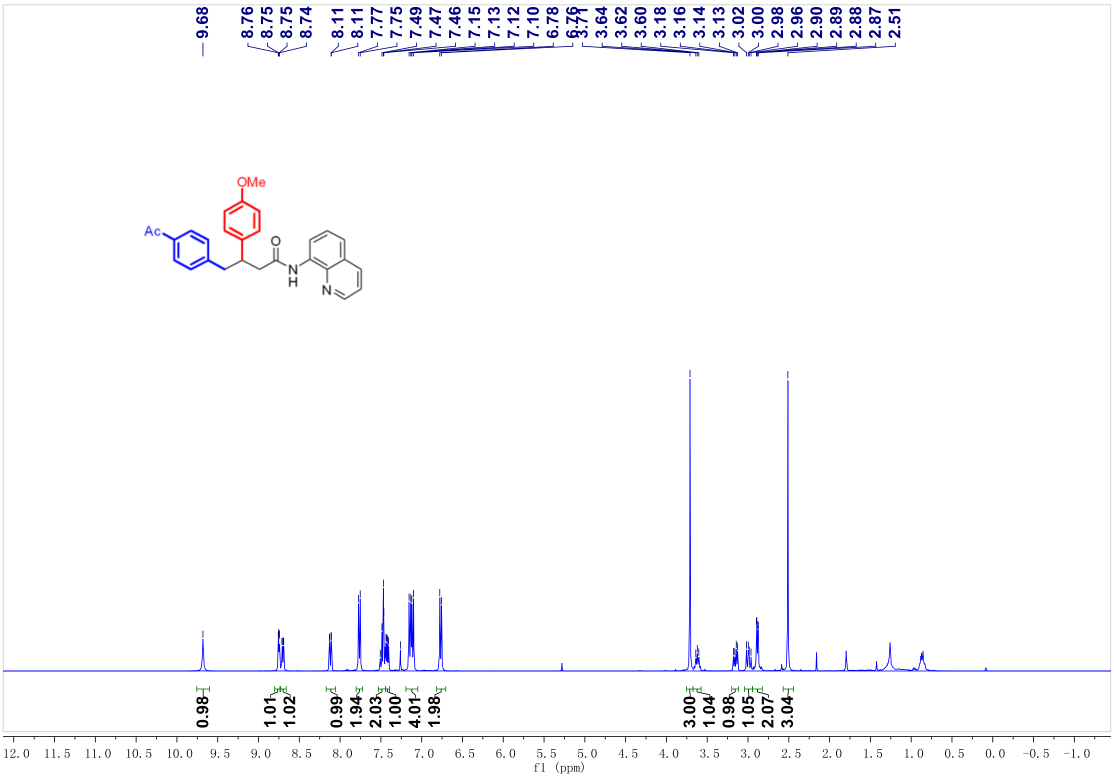
9.68
8.76
8.75
8.75
8.74
8.11
8.11
7.77
7.75
7.49
7.47
7.46
7.15
7.13
7.12
7.10
6.78
6.76
3.71
3.64
3.62
3.60
3.18
3.16
3.14
3.13
3.02
3.00
2.98
2.96
2.90
2.89
2.88
2.87
2.51
OMe
Ac
O
N
H
N
0.98
1.01
1.02
0.99
1.94
2.03
1.00
4.01
1.98
3.00
1.04
0.98
1.05
2.07
3.04
12.0 11.5 11.0 10.5 10.0 9.5 9.0 8.5 8.0 7.5 7.0 6.5 6.0 5.5 5.0 4.5 4.0 3.5 3.0 2.5 2.0 1.5 1.0 0.5 0.0 -0.5 -1.0
f1 (ppm)

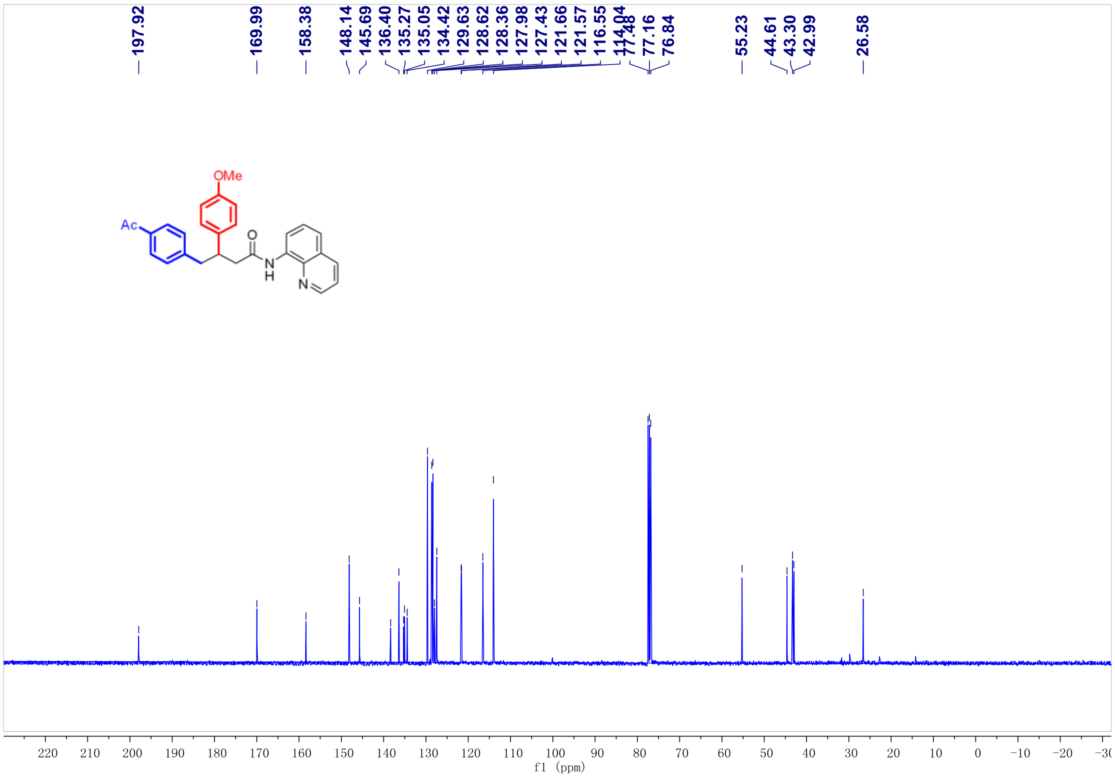
197.92
169.99
158.38
148.14
145.69
136.40
135.27
135.05
134.42
129.63
128.62
128.36
127.98
127.43
121.66
121.57
116.55
114.04
77.48
77.16
76.84
55.23
44.61
43.30
42.99
26.58
OMe
Ac
O
N
H
N
220 210 200 190 180 170 160 150 140 130 120 110 100 90 80 70 60 50 40 30 20 10 0 -10 -20 -30
f1 (ppm)

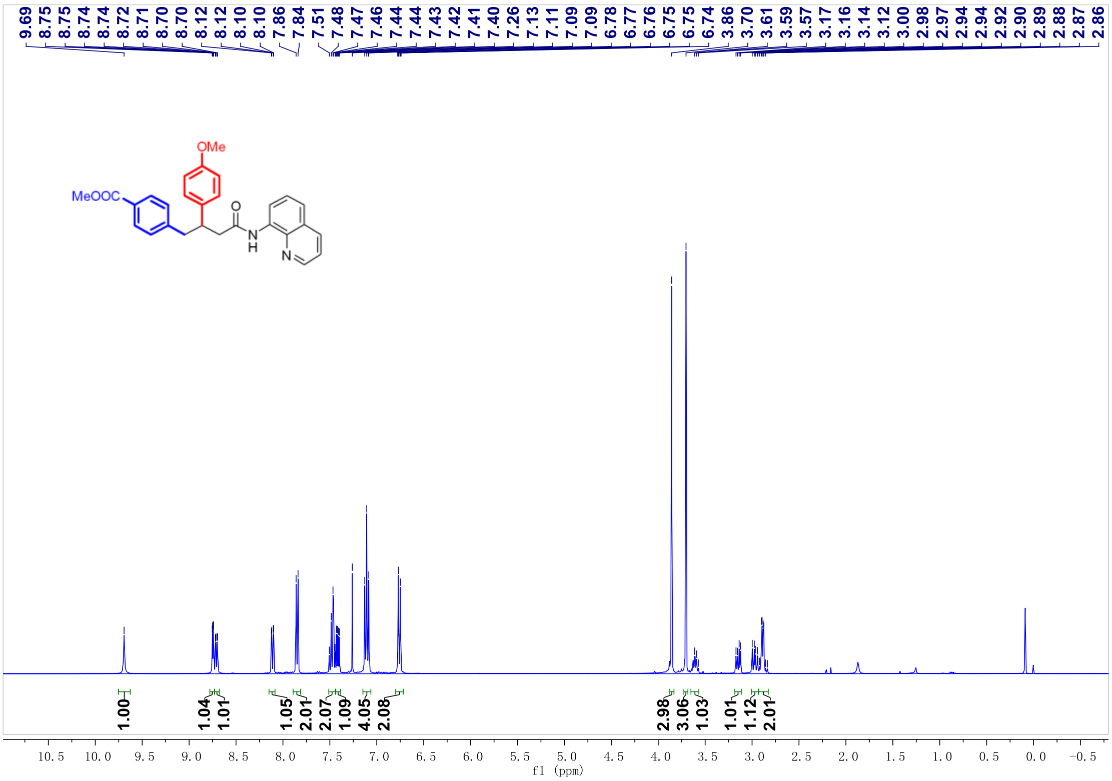
9.69 8.75 8.75 8.74 8.74 8.72 8.71 8.70 8.70 8.12 8.12 8.10 8.10 7.86 7.84 7.51 7.48 7.47 7.46 7.44 7.44 7.43 7.42 7.41 7.40 7.26 7.13 7.11 7.09 7.09 6.78 6.77 6.76 6.75 6.75 6.74 3.86 3.70 3.61 3.59 3.57 3.17 3.16 3.14 3.12 3.00 2.98 2.97 2.94 2.94 2.92 2.90 2.89 2.88 2.87 2.86
OMe
MeOOC
O
N
H
N
1.00
1.04
1.01
1.05
2.01
2.07
1.09
4.05
2.08
2.98
3.06
1.03
1.01
1.12
2.01
f1 (ppm)

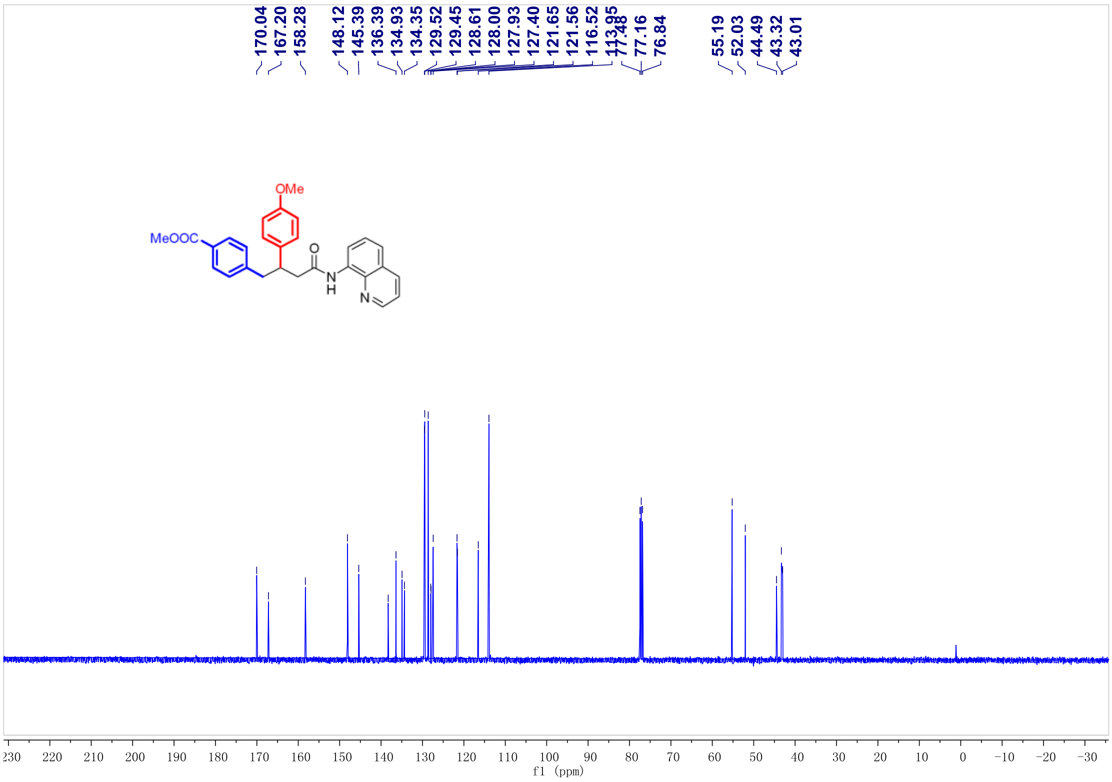
170.04 167.20 158.28 148.12 145.39 136.39 134.93 134.35 129.52 129.45 128.61 128.00 127.93 127.40 121.65 121.56 116.52 113.95 77.48 77.16 76.84 55.19 52.03 44.49 43.32 43.01
OMe
MeOOC
O
N
H
N
f1 (ppm)

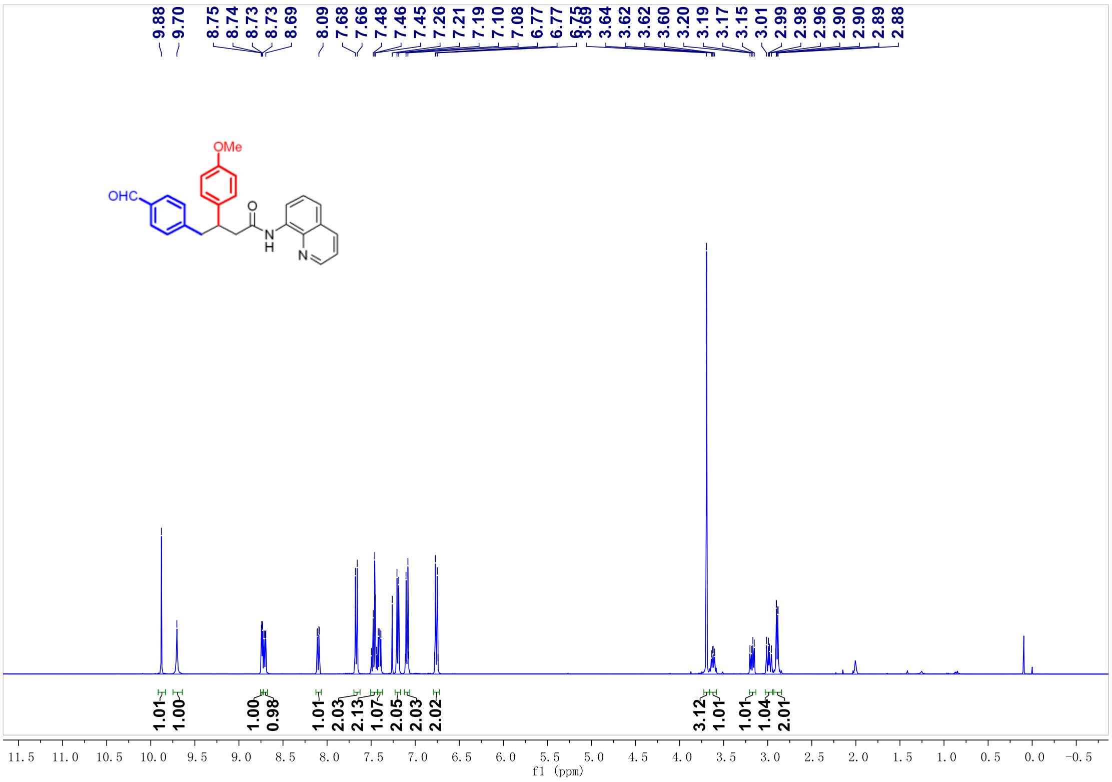
9.88
9.70
8.75
8.74
8.73
8.73
8.69
8.09
7.68
7.66
7.48
7.46
7.45
7.26
7.21
7.19
7.10
7.08
6.77
6.75
3.69
3.64
3.62
3.62
3.60
3.20
3.19
3.17
3.15
3.01
2.99
2.98
2.96
2.90
2.90
2.89
2.88
OHC
OMe
O
N
H
N
1.01
1.00
1.00
0.98
1.01
2.03
2.13
1.07
2.05
2.03
2.02
3.12
1.01
1.01
1.04
2.01
f1 (ppm)

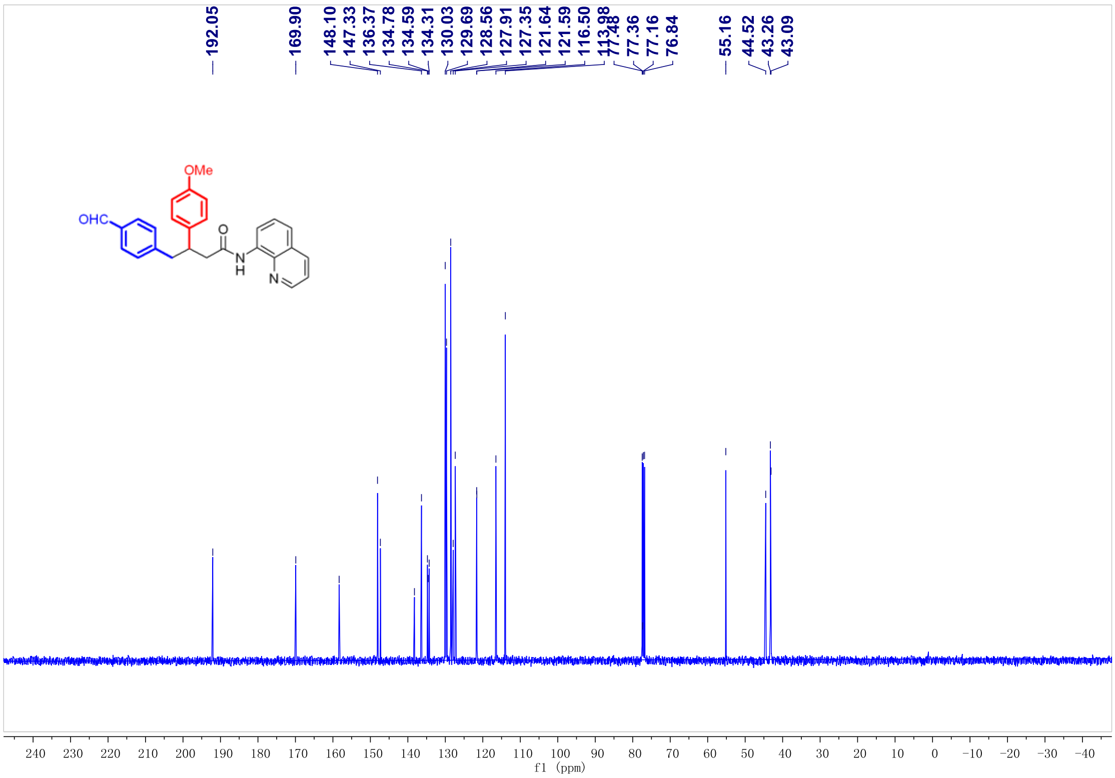
192.05
169.90
148.10
147.33
136.37
134.78
134.59
134.31
130.03
129.69
128.56
127.91
127.35
121.64
121.59
116.50
113.98
77.48
77.36
77.16
76.84
55.16
44.52
43.26
43.09
OHC
OMe
O
N
H
N
f1 (ppm)

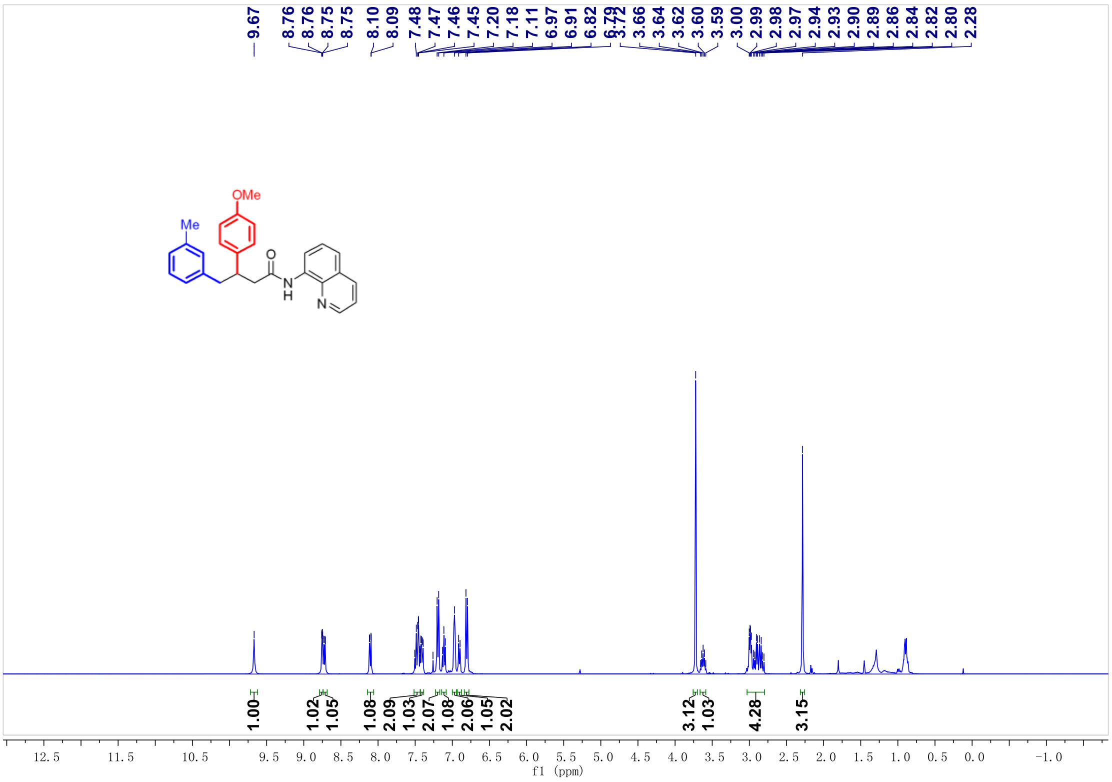

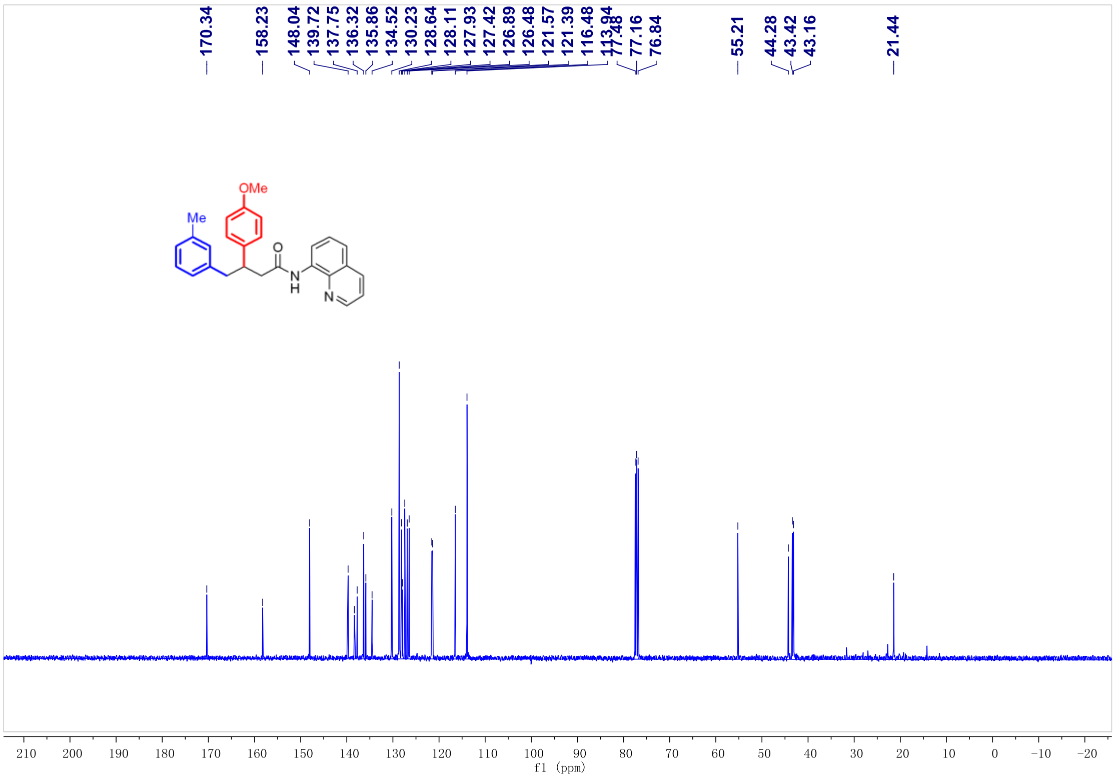

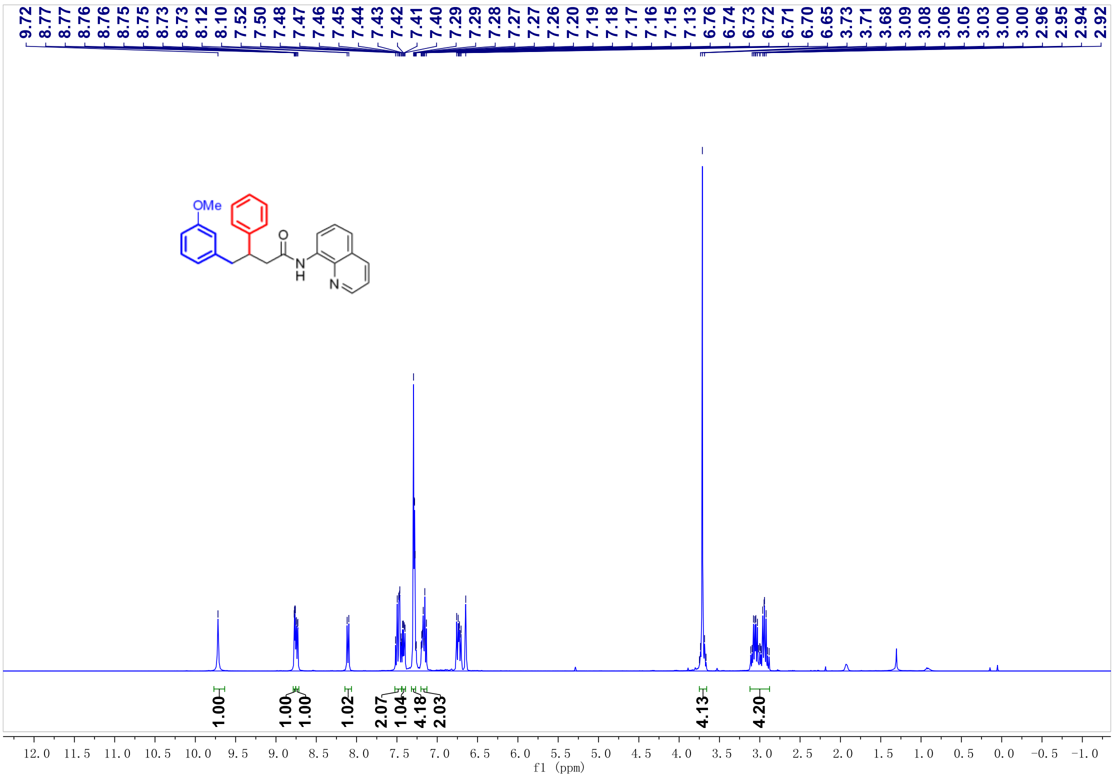

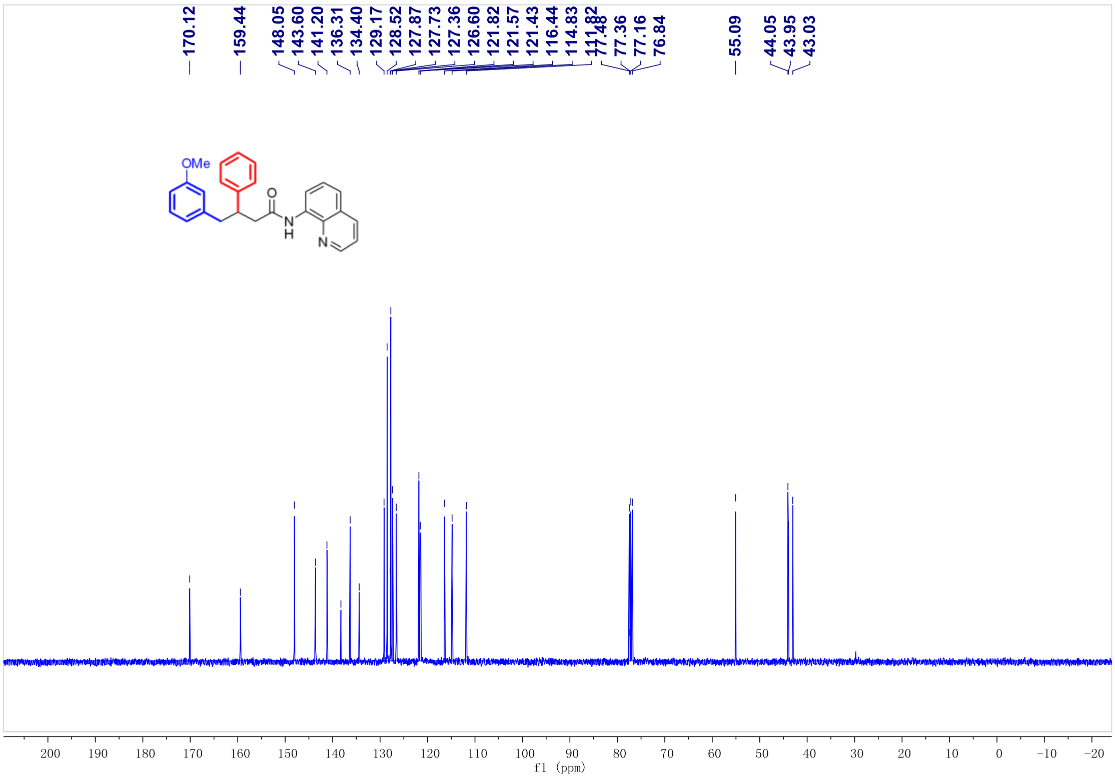

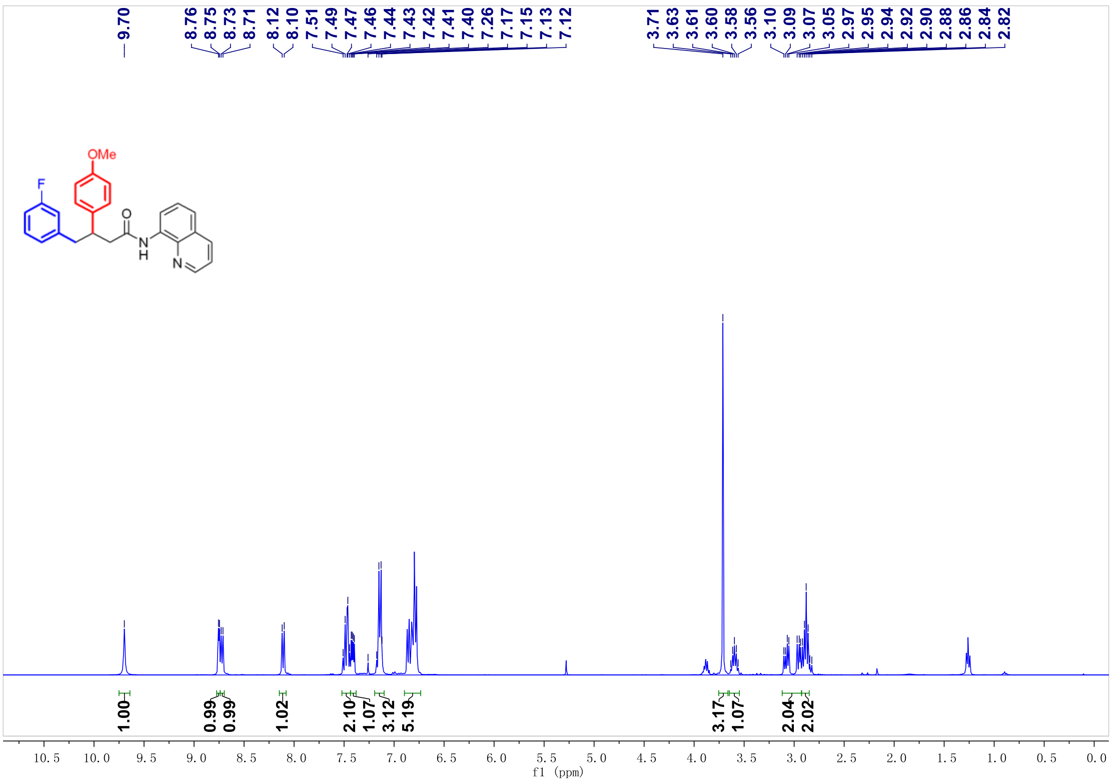

9.70
8.76
8.75
8.73
8.71
8.12
8.10
7.51
7.49
7.47
7.46
7.44
7.43
7.42
7.41
7.40
7.26
7.17
7.15
7.13
7.12
3.71
3.63
3.61
3.60
3.58
3.56
3.10
3.09
3.07
3.05
2.97
2.95
2.94
2.92
2.90
2.88
2.86
2.84
2.82
OMe
F
O
N
H
N
1.00
0.99
0.99
1.02
2.10
1.07
3.12
5.19
3.17
1.07
2.04
2.02
f1 (ppm)

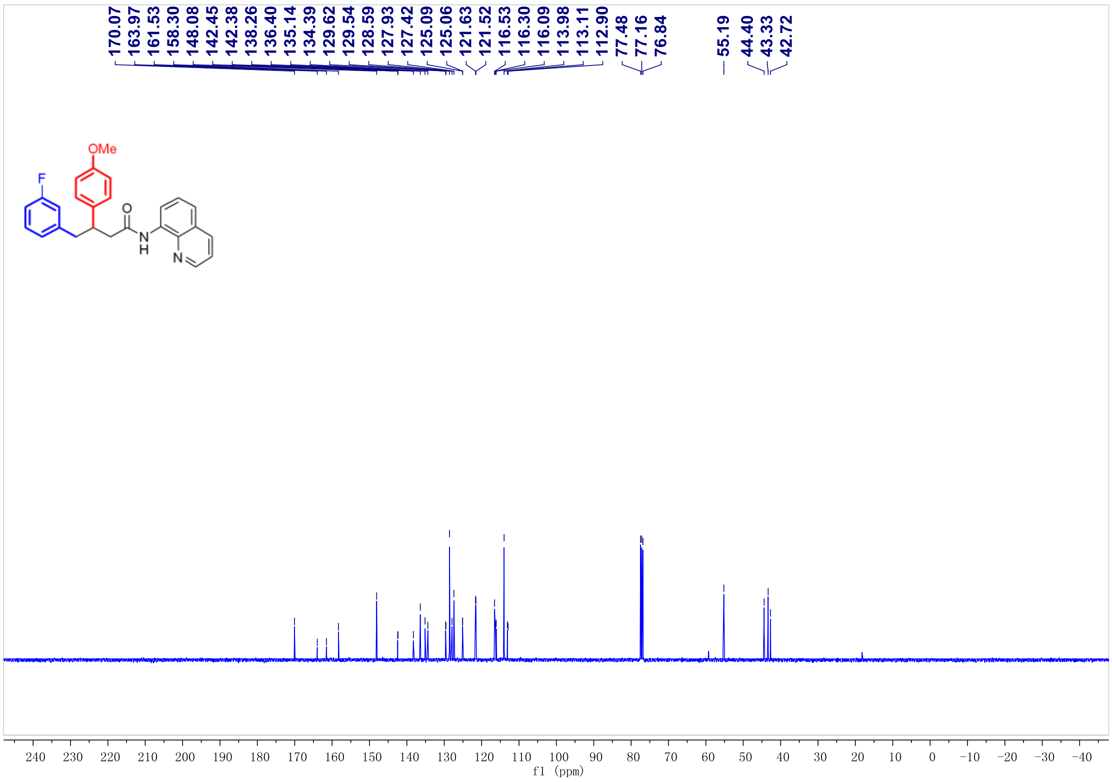

170.07
163.97
161.53
158.30
148.08
142.45
142.38
138.26
136.40
135.14
134.39
129.62
129.54
128.59
127.93
127.42
125.09
125.06
121.63
121.52
116.53
116.30
116.09
113.98
113.11
112.90
77.48
77.16
76.84
55.19
44.40
43.33
42.72
OMe
F
O
N
H
N
f1 (ppm)

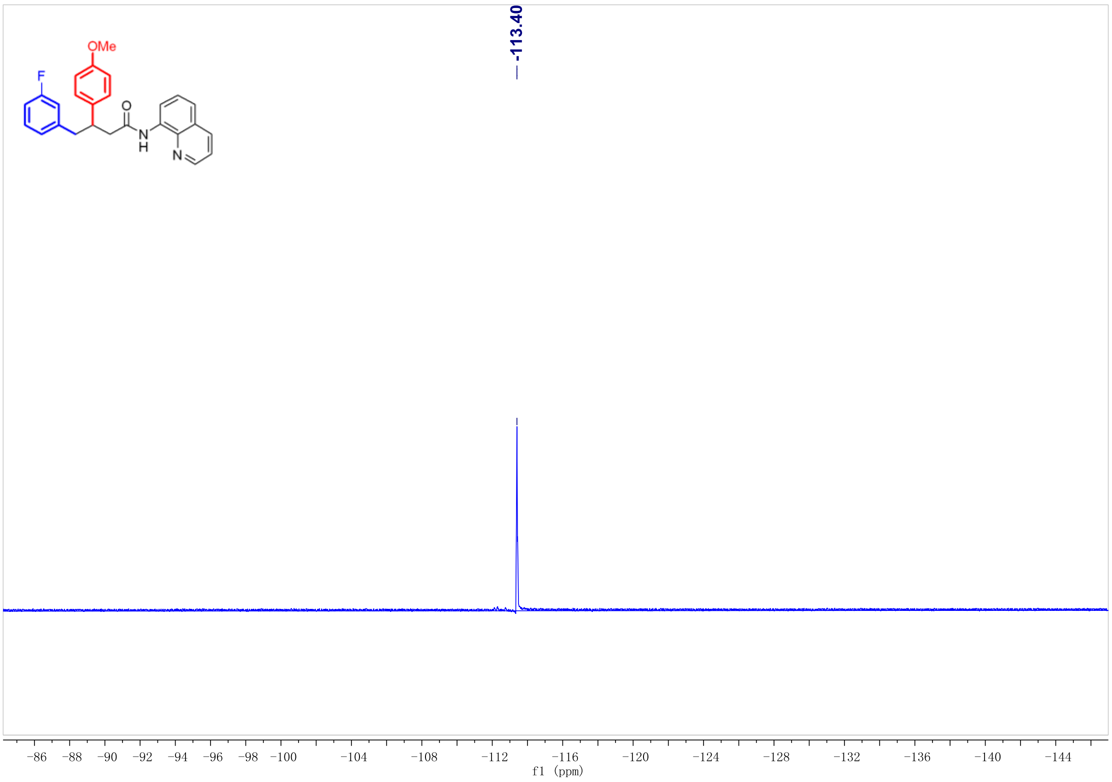

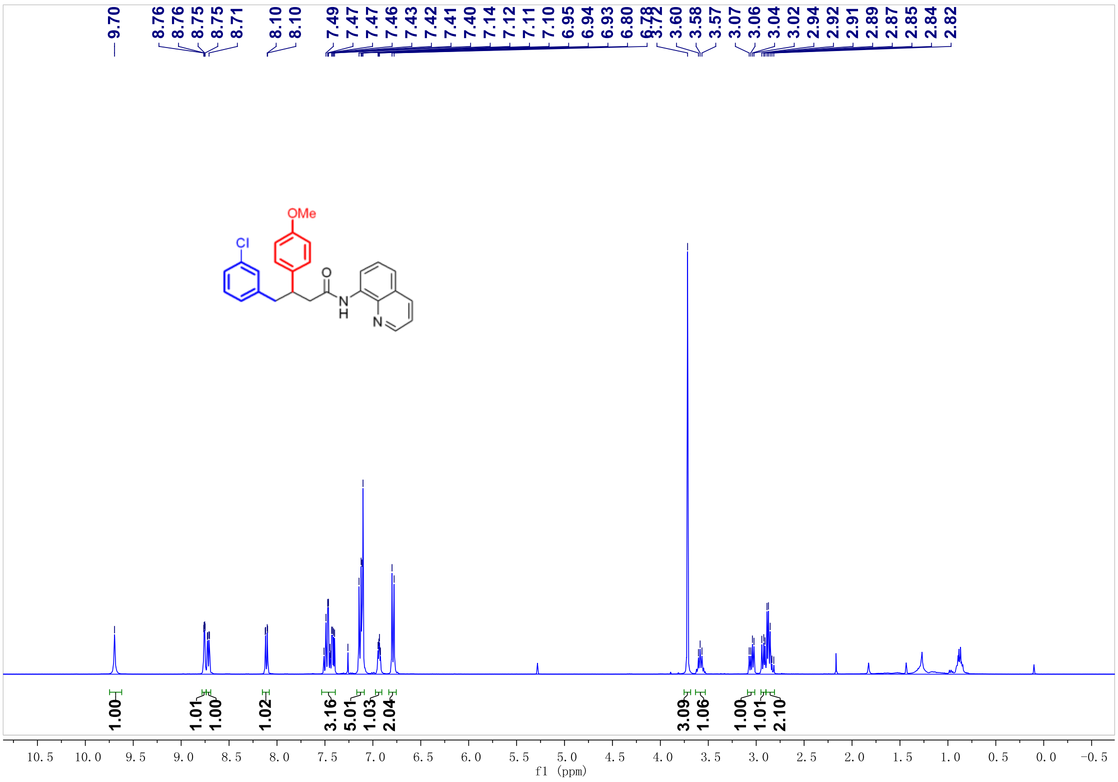

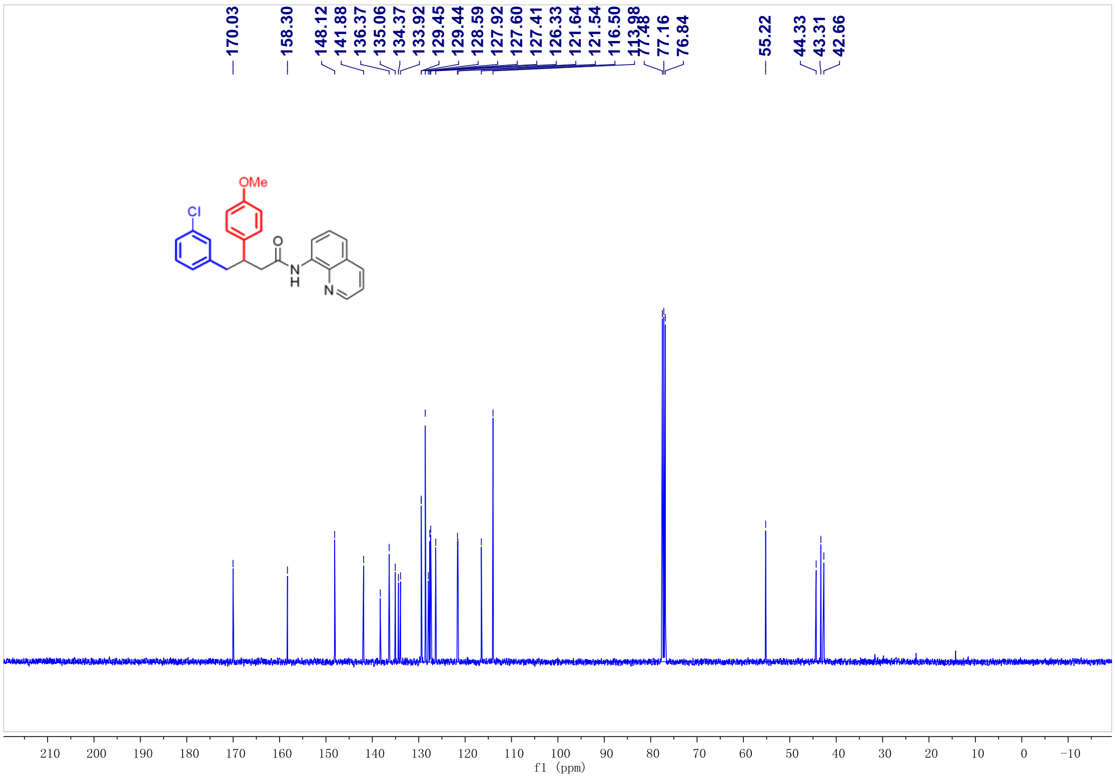
170.03
158.30
148.12
141.88
136.37
135.06
134.37
133.92
129.45
129.44
128.59
127.92
127.60
127.41
126.33
121.64
121.54
116.50
113.98
77.48
77.16
76.84
55.22
44.33
43.31
42.66
OMe
Cl
O
N
H
N
f1 (ppm)

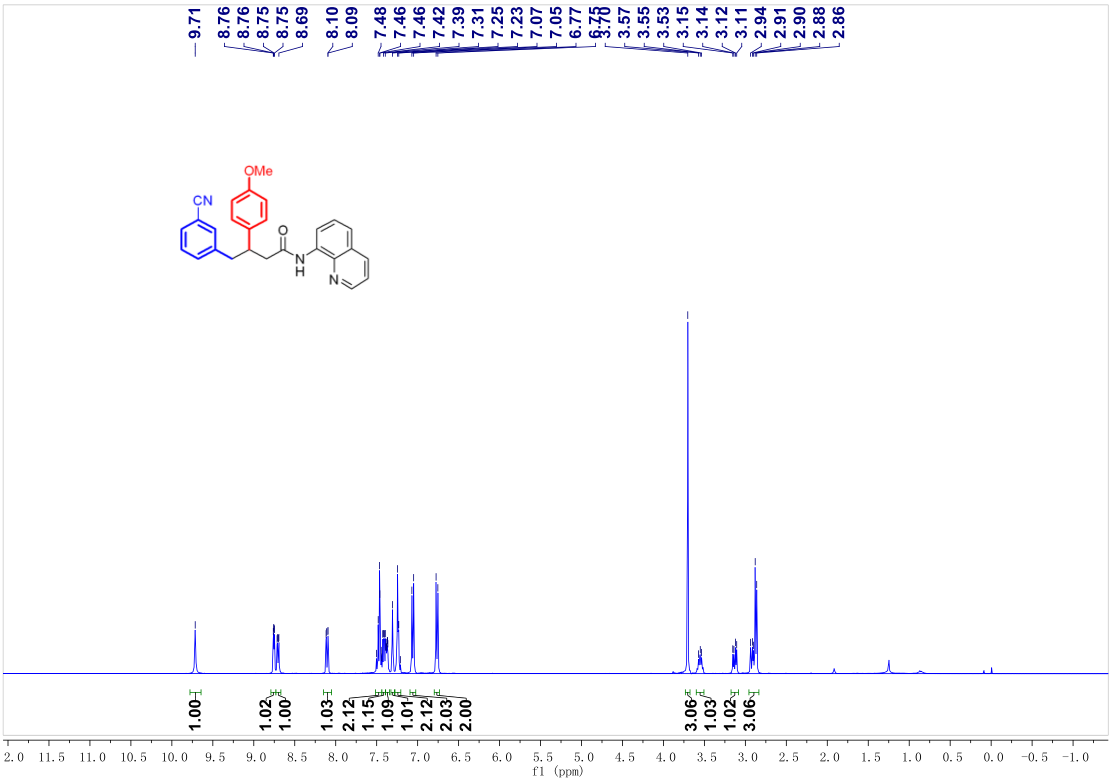
9.71
8.76
8.76
8.75
8.75
8.69
8.10
8.09
7.48
7.46
7.46
7.42
7.39
7.31
7.25
7.23
7.07
7.05
6.77
6.75
3.70
3.57
3.55
3.53
3.15
3.14
3.12
3.11
2.94
2.91
2.90
2.88
2.86
OMe
CN
O
N
H
N
1.00
1.02
1.00
1.03
2.12
1.15
1.09
1.01
2.12
2.03
2.00
3.06
1.03
1.02
3.06
f1 (ppm)

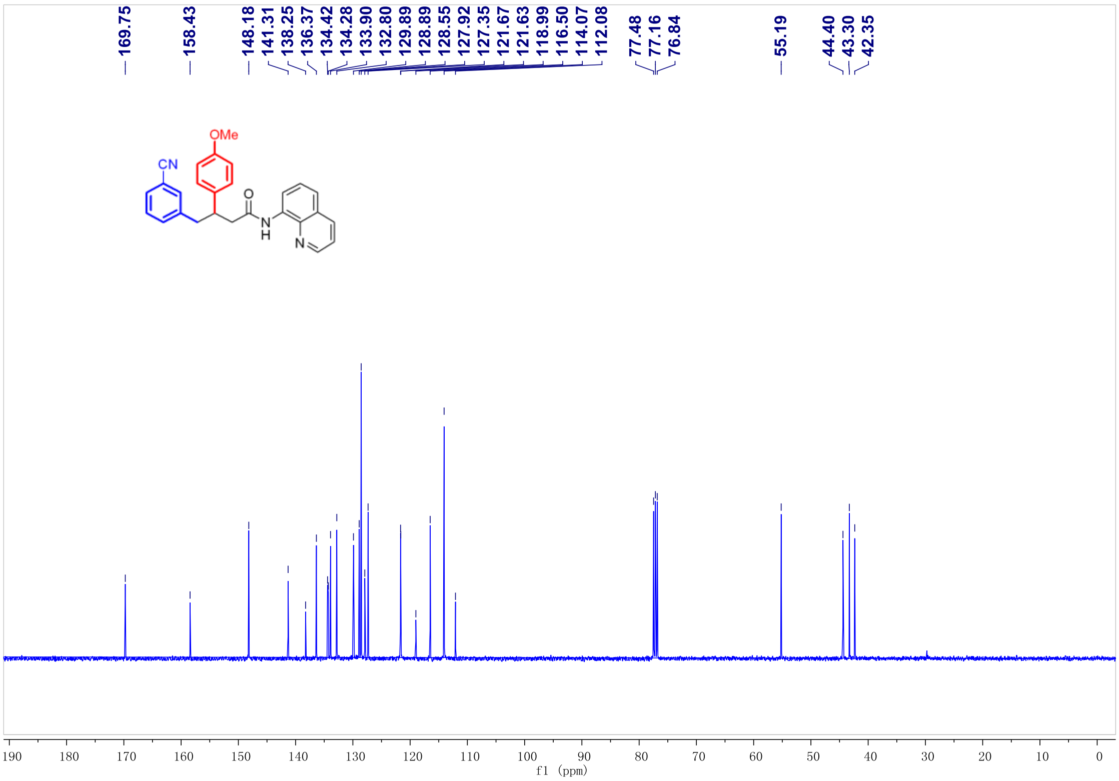
169.75
158.43
148.18
141.31
138.25
136.37
134.42
134.28
133.90
132.80
129.89
128.89
128.55
127.92
127.35
121.67
121.63
118.99
116.50
114.07
112.08
77.48
77.16
76.84
55.19
44.40
43.30
42.35
CN
OMe
O
N
H
N
190 180 170 160 150 140 130 120 110 100 90 80 70 60 50 40 30 20 10 0
f1 (ppm)

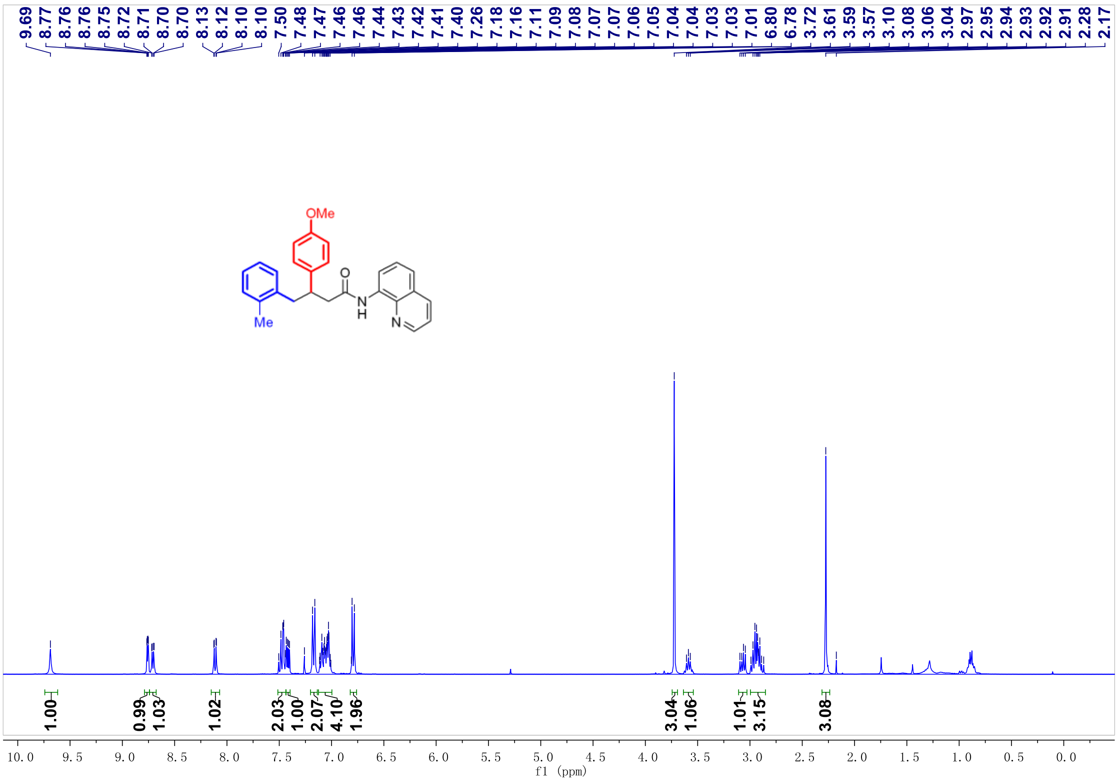
9.69
8.77
8.76
8.76
8.75
8.72
8.71
8.70
8.70
8.13
8.12
8.10
8.10
7.50
7.48
7.47
7.46
7.46
7.44
7.43
7.42
7.41
7.40
7.26
7.18
7.16
7.11
7.09
7.08
7.07
7.07
7.06
7.05
7.04
7.04
7.03
7.03
7.01
6.80
6.78
3.72
3.61
3.59
3.57
3.10
3.08
3.06
3.04
2.97
2.95
2.94
2.93
2.92
2.91
2.28
2.17
OMe
Me
O
N
H
N
1.00
0.99
1.03
1.02
2.03
1.00
2.07
4.10
1.96
3.04
1.06
1.01
3.15
3.08
10.0 9.5 9.0 8.5 8.0 7.5 7.0 6.5 6.0 5.5 5.0 4.5 4.0 3.5 3.0 2.5 2.0 1.5 1.0 0.5 0.0
f1 (ppm)

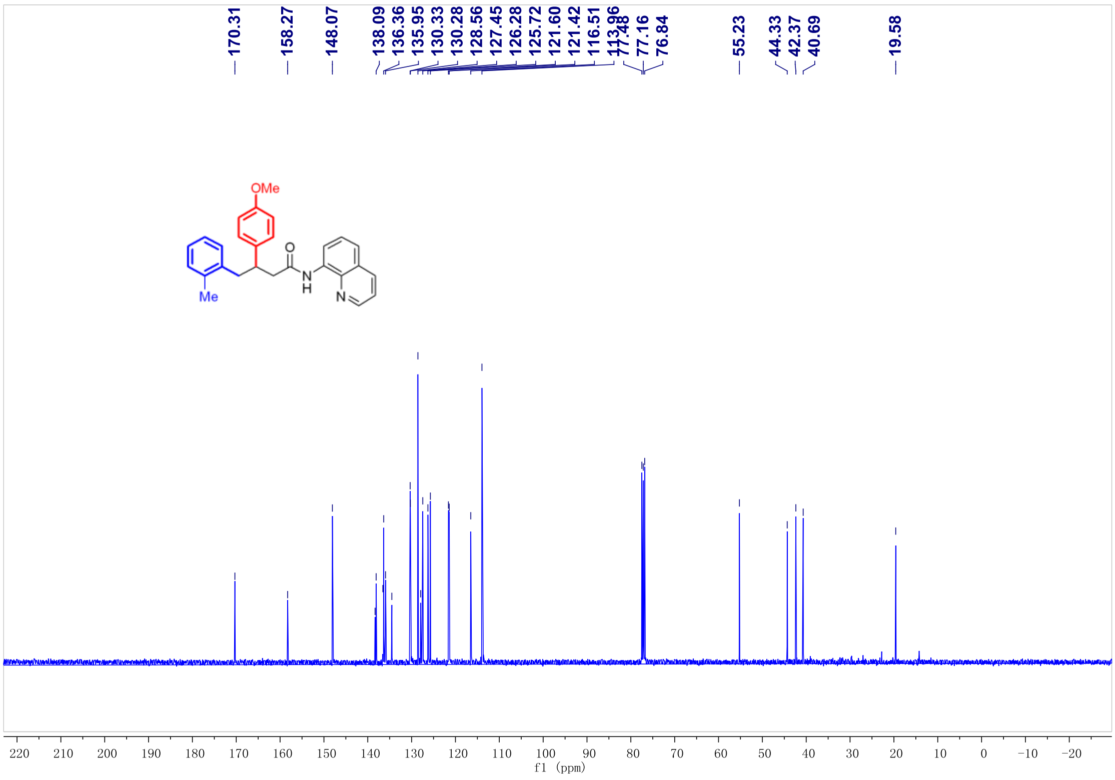
170.31
158.27
148.07
138.09
136.36
135.95
130.33
130.28
128.56
127.45
126.28
125.72
121.60
121.42
116.51
113.96
77.48
77.16
76.84
55.23
44.33
42.37
40.69
19.58
OMe
Me
O
N
H
f1 (ppm)

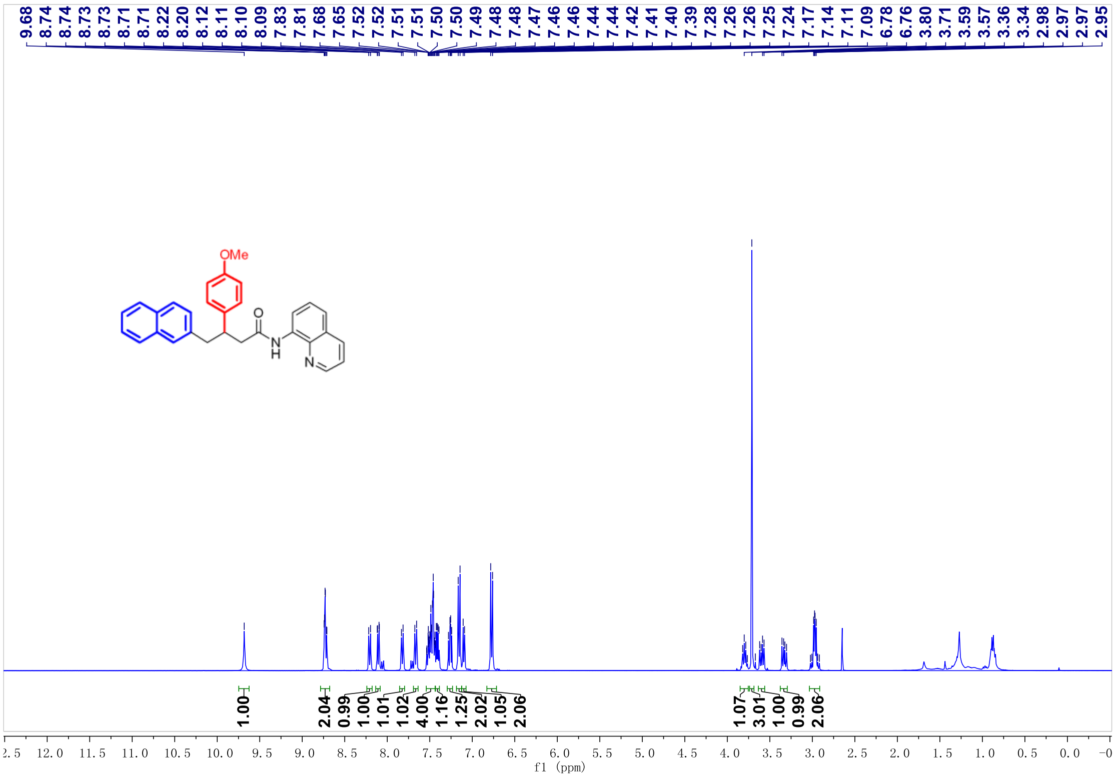
9.68
8.74
8.74
8.73
8.73
8.71
8.71
8.22
8.20
8.12
8.11
8.10
8.09
7.83
7.81
7.68
7.65
7.52
7.51
7.51
7.50
7.50
7.49
7.48
7.48
7.47
7.46
7.46
7.44
7.44
7.42
7.41
7.40
7.39
7.28
7.26
7.26
7.25
7.24
7.17
7.14
7.11
7.09
6.78
6.76
3.80
3.71
3.59
3.57
3.36
3.34
2.98
2.97
2.97
2.95
OMe
O
N
H
1.00
2.04
0.99
1.00
1.01
1.02
4.00
1.16
1.25
2.02
1.05
2.06
1.07
3.01
1.00
0.99
2.06
f1 (ppm)

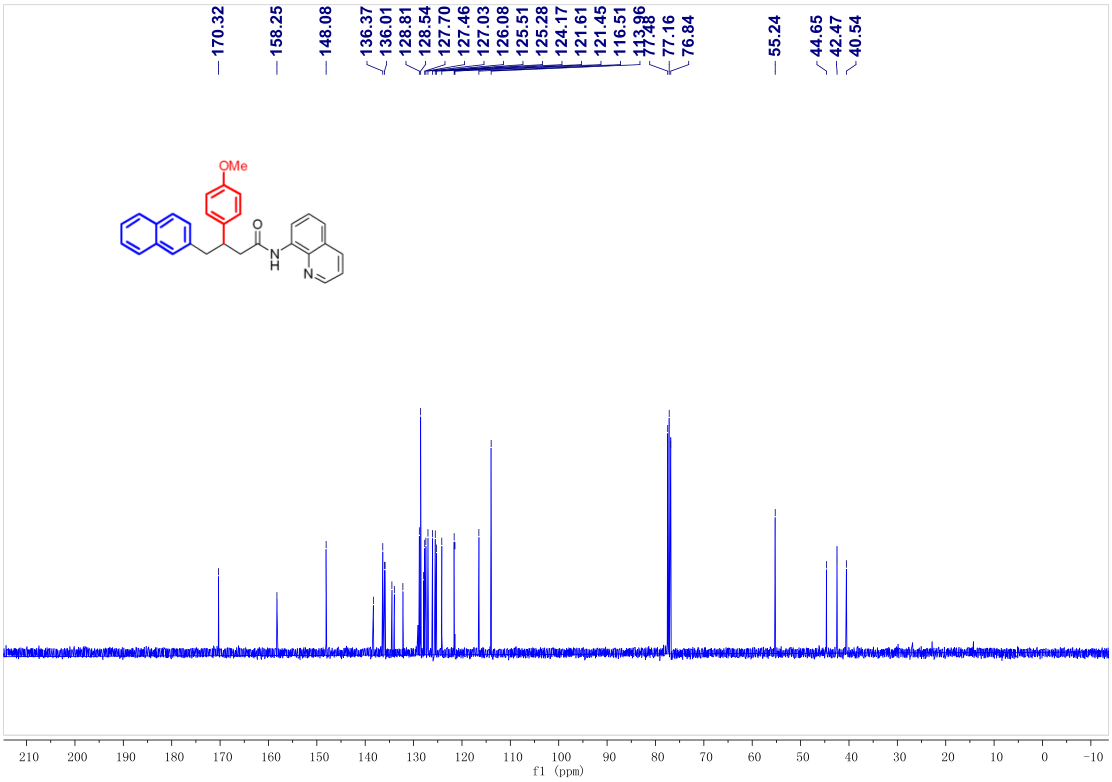
170.32
158.25
148.08
136.37
136.01
128.81
128.54
127.70
127.46
127.03
126.08
125.51
125.28
124.17
121.61
121.45
116.51
113.96
77.48
77.16
76.84
55.24
44.65
42.47
40.54
OMe
O
N
H
N
f1 (ppm)

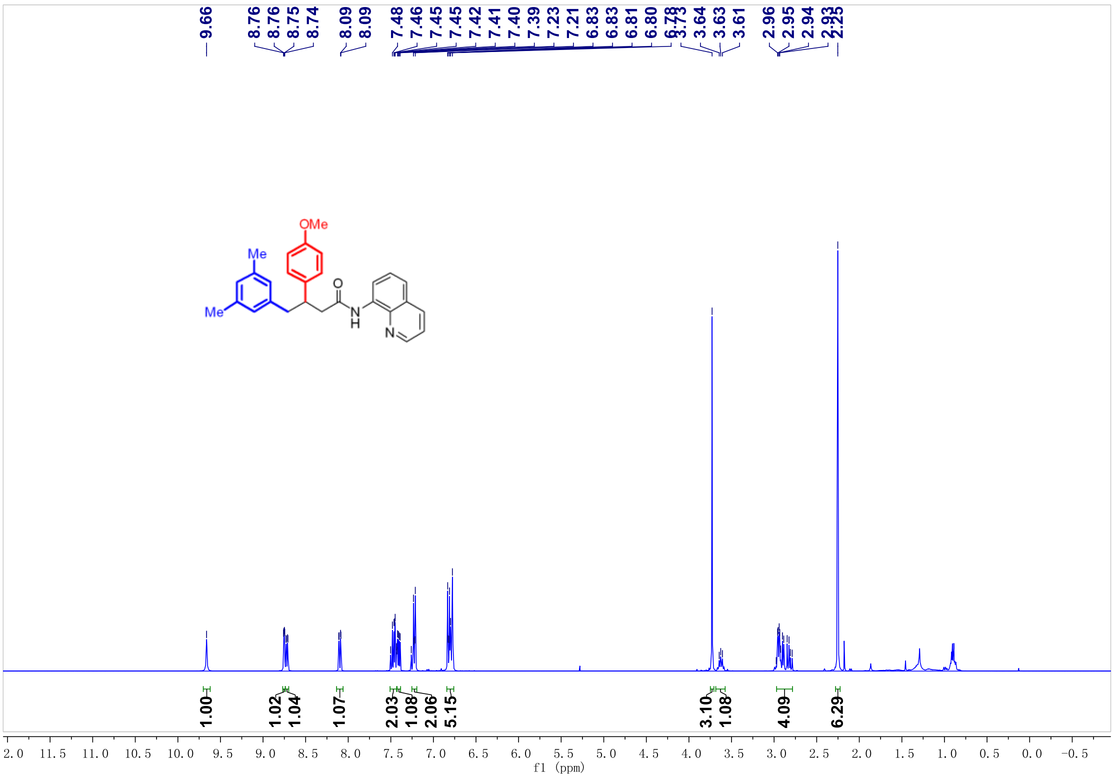
9.66
8.76
8.76
8.75
8.74
8.09
8.09
7.48
7.46
7.45
7.45
7.42
7.41
7.40
7.39
7.23
7.21
6.83
6.83
6.81
6.80
6.78
3.73
3.64
3.63
3.61
2.96
2.95
2.94
2.93
2.25
OMe
Me
Me
O
N
H
N
1.00
1.02
1.04
1.07
2.03
1.08
2.06
5.15
3.10
1.08
4.09
6.29
f1 (ppm)

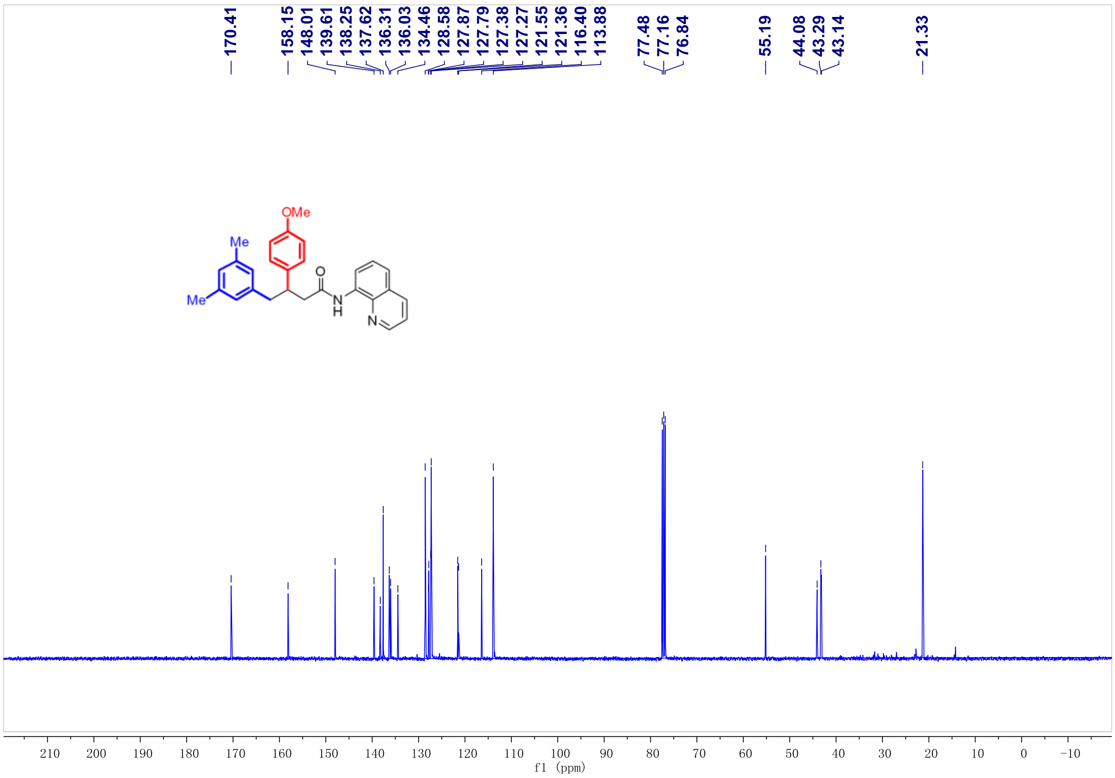

170.41
158.15
148.01
139.61
138.25
137.62
136.31
136.03
134.46
128.58
127.87
127.79
127.38
127.27
121.55
121.36
116.40
113.88
77.48
77.16
76.84
55.19
44.08
43.29
43.14
21.33
OMe
Me
Me
O
N
H
N
f1 (ppm)

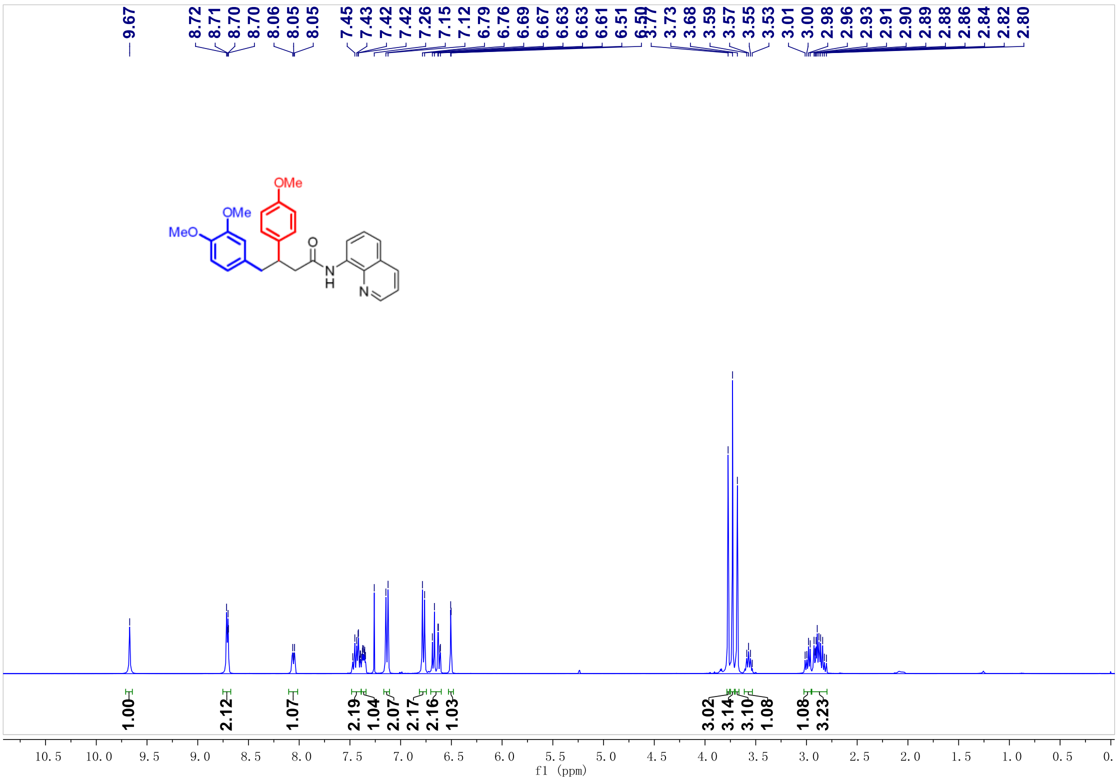

9.67
8.72
8.71
8.70
8.06
8.05
8.05
7.45
7.43
7.42
7.42
7.26
7.15
7.12
6.79
6.76
6.69
6.67
6.63
6.63
6.61
6.51
6.50
3.77
3.73
3.68
3.59
3.57
3.55
3.53
3.01
3.00
2.98
2.96
2.93
2.91
2.90
2.89
2.88
2.86
2.84
2.82
2.80
OMe
OMe
MeO
O
N
H
N
1.00
2.12
1.07
2.19
1.04
2.07
2.17
2.16
1.03
3.02
3.14
3.10
1.08
1.08
3.23
f1 (ppm)

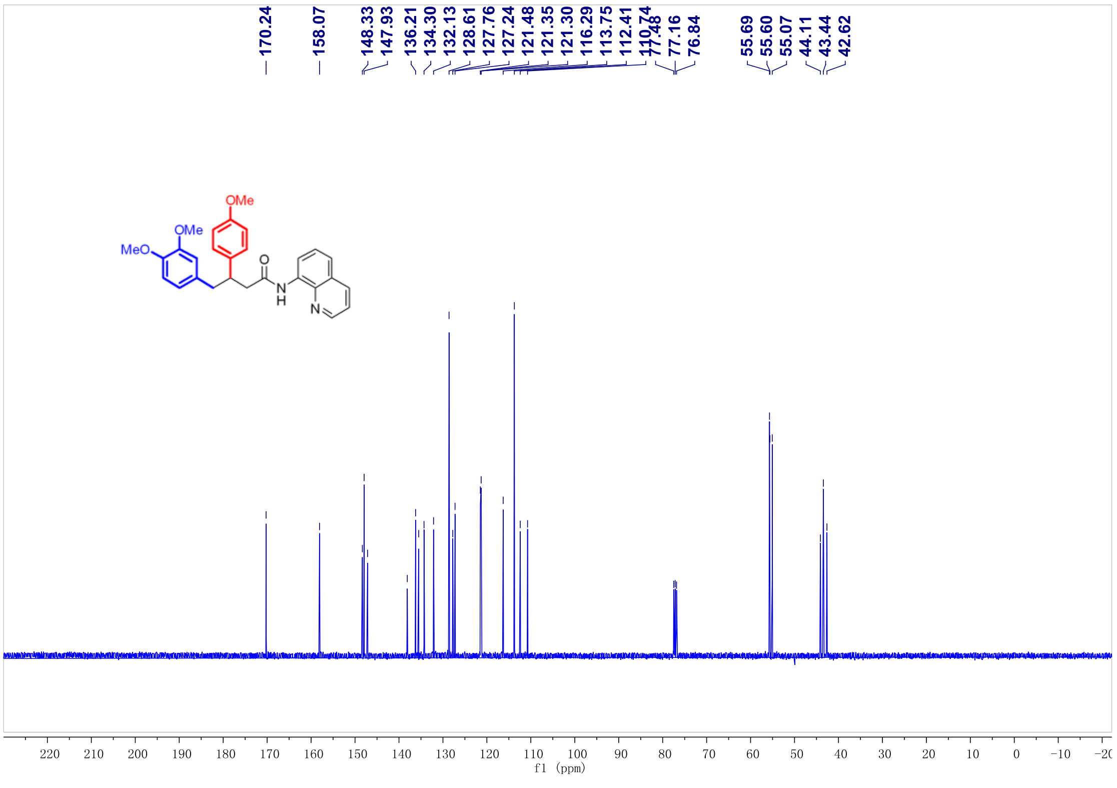

170.24
158.07
148.33
147.93
136.21
134.30
132.13
128.61
127.76
127.24
121.48
121.35
121.30
116.29
113.75
112.41
110.74
77.48
77.16
76.84
55.69
55.60
55.07
44.11
43.44
42.62
OMe
OMe
MeO
O
N
H
N
f1 (ppm)

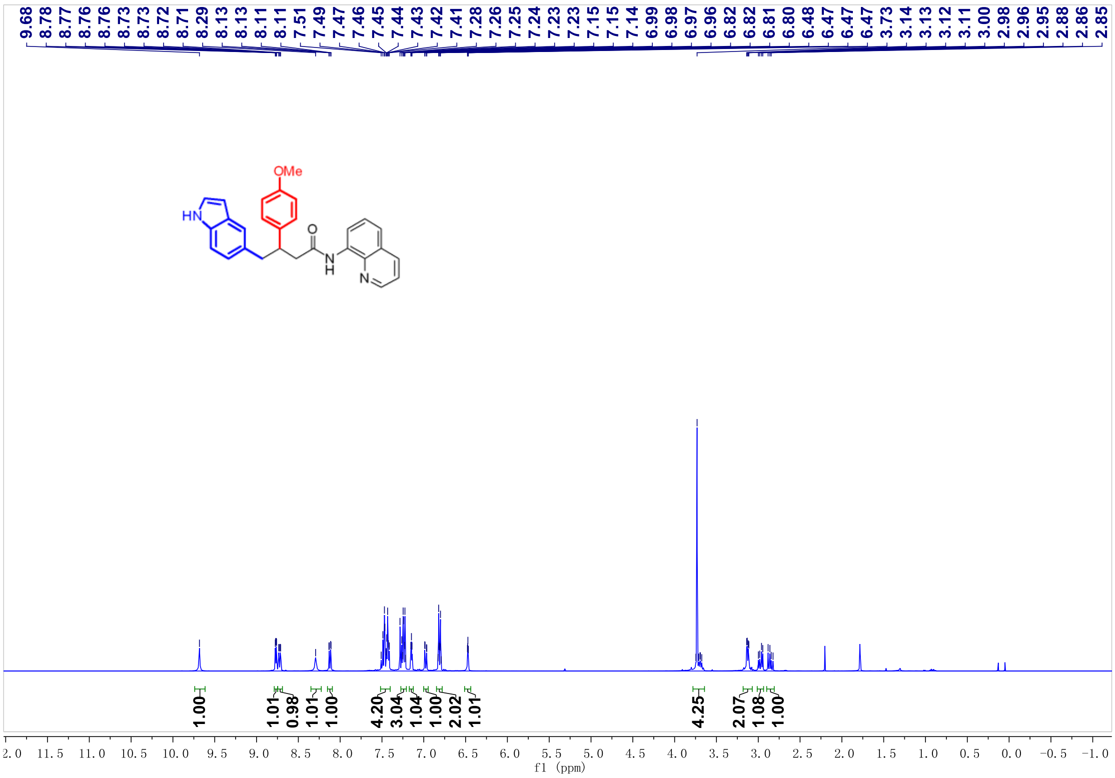
9.68
8.78
8.77
8.76
8.76
8.73
8.73
8.72
8.71
8.29
8.13
8.13
8.11
8.11
7.51
7.49
7.47
7.46
7.45
7.44
7.43
7.42
7.41
7.28
7.26
7.25
7.24
7.23
7.23
7.15
7.15
7.14
6.99
6.98
6.97
6.96
6.82
6.82
6.81
6.80
6.48
6.47
6.47
6.47
3.73
3.14
3.13
3.12
3.11
3.00
2.98
2.96
2.95
2.88
2.86
2.85
OMe
HN
O
N
H
N
1.00
1.01
0.98
1.01
1.00
4.20
3.04
1.04
1.00
2.02
1.01
4.25
2.07
1.08
1.00
f1 (ppm)

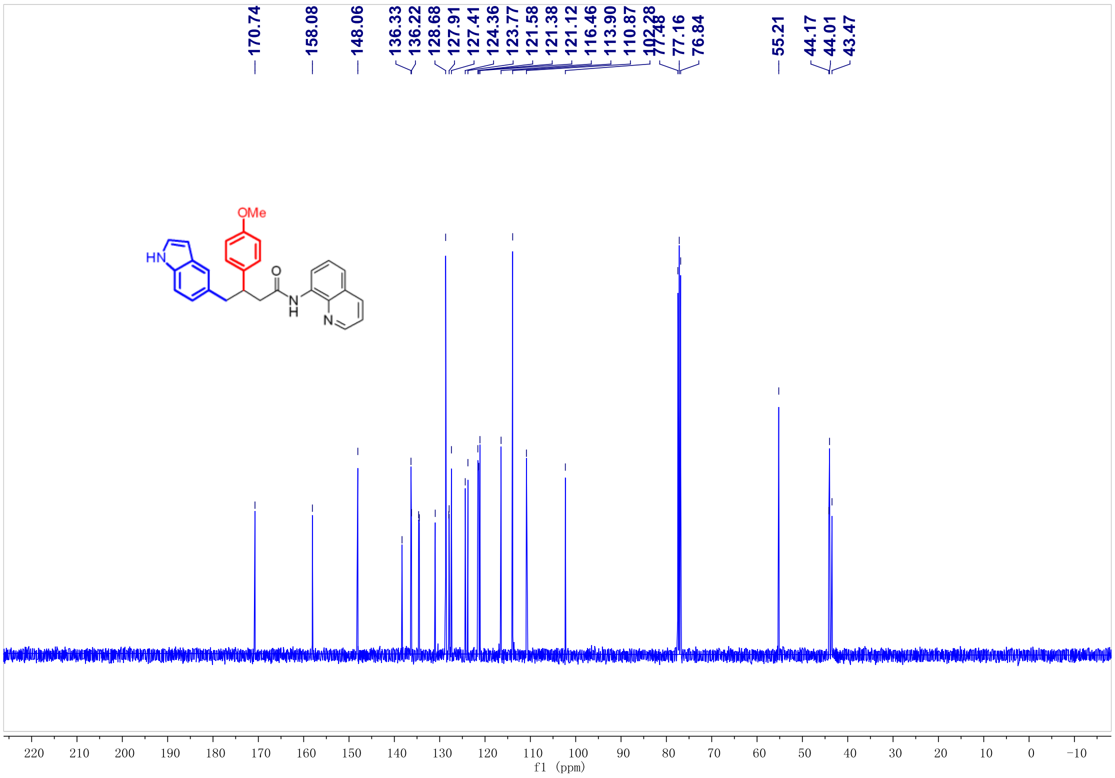
170.74
158.08
148.06
136.33
136.22
128.68
127.91
127.41
124.36
123.77
121.58
121.38
121.12
116.46
113.90
110.87
102.28
77.48
77.16
76.84
55.21
44.17
44.01
43.47
OMe
HN
O
N
H
N
f1 (ppm)

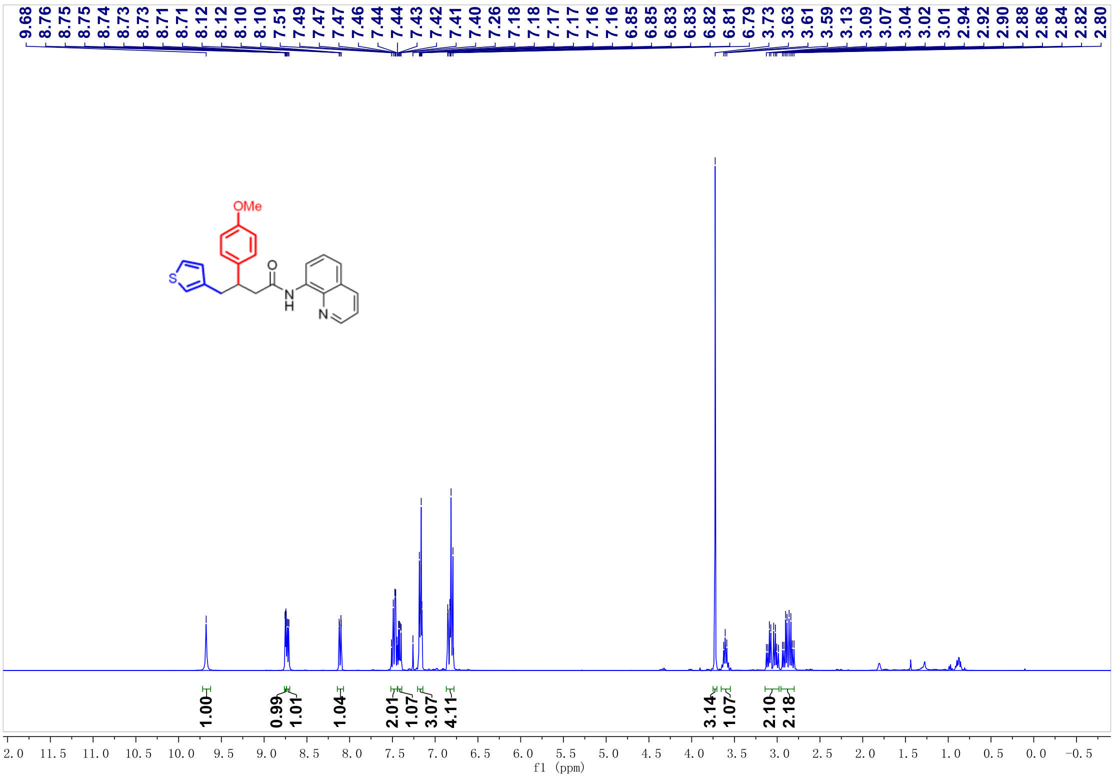
9.68
8.76
8.75
8.75
8.74
8.73
8.73
8.71
8.71
8.12
8.12
8.10
8.10
7.51
7.49
7.47
7.47
7.46
7.44
7.44
7.43
7.42
7.41
7.40
7.26
7.18
7.18
7.17
7.17
7.16
7.16
6.85
6.85
6.83
6.83
6.82
6.81
6.79
3.73
3.63
3.61
3.59
3.13
3.09
3.07
3.04
3.02
3.01
2.94
2.92
2.90
2.88
2.86
2.84
2.82
2.80
OMe
S
O
N
H
N
1.00
0.99
1.01
1.04
2.01
1.07
3.07
4.11
3.14
1.07
2.10
2.18
f1 (ppm)

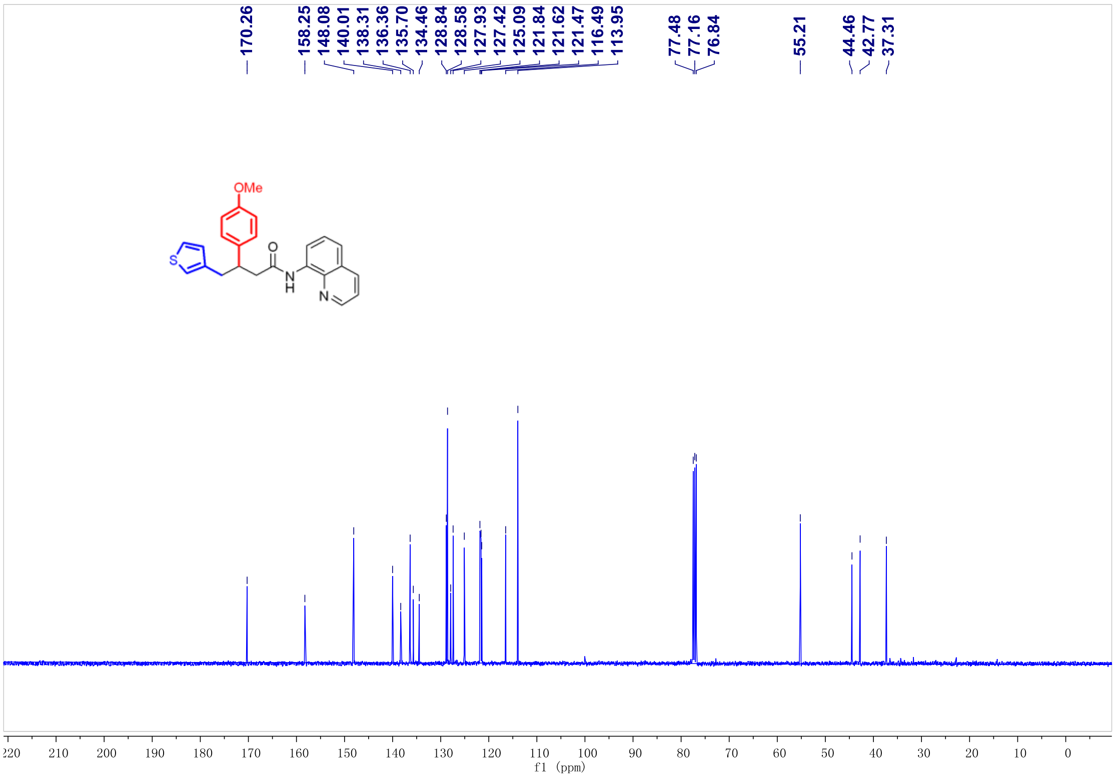
170.26
158.25
148.08
140.01
138.31
136.36
135.70
134.46
128.84
128.58
127.93
127.42
125.09
121.84
121.62
121.47
116.49
113.95
77.48
77.16
76.84
55.21
44.46
42.77
37.31
OMe
S
O
N
H
N
f1 (ppm)

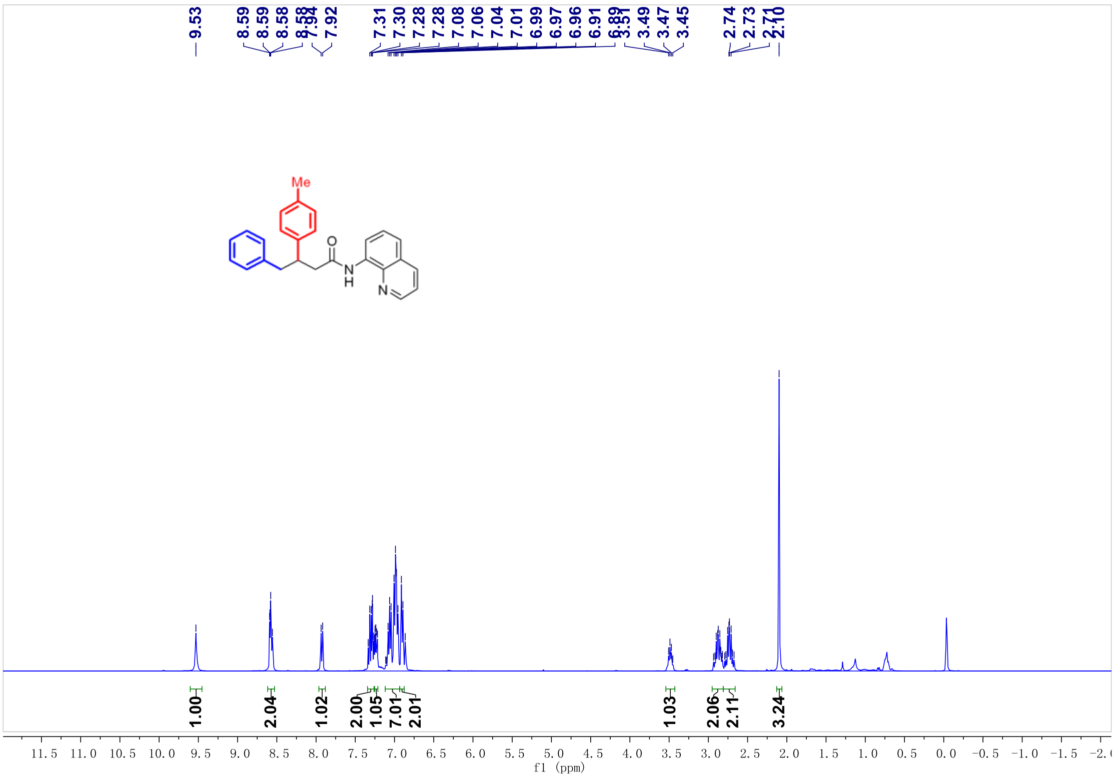
9.53
8.59
8.59
8.58
8.58
7.94
7.92
7.31
7.30
7.28
7.08
7.06
7.04
7.01
6.99
6.97
6.96
6.91
6.89
3.51
3.49
3.47
3.45
2.74
2.73
2.71
2.10
Me
O
N
H
N
1.00
2.04
1.02
2.00
1.05
7.01
2.01
1.03
2.06
2.11
3.24
f1 (ppm)

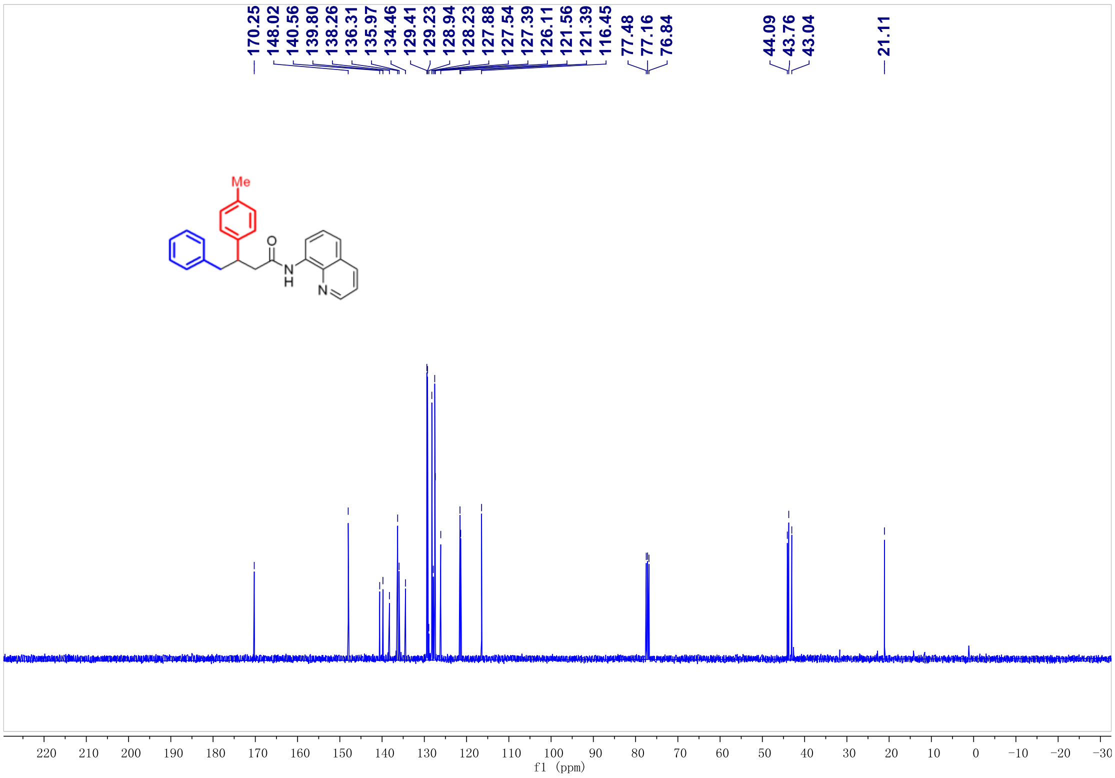
170.25
148.02
140.56
139.80
138.26
136.31
135.97
134.46
129.41
129.23
128.94
128.23
127.88
127.54
127.39
126.11
121.56
121.39
116.45
77.48
77.16
76.84
44.09
43.76
43.04
21.11
Me
O
N
H
N
f1 (ppm)

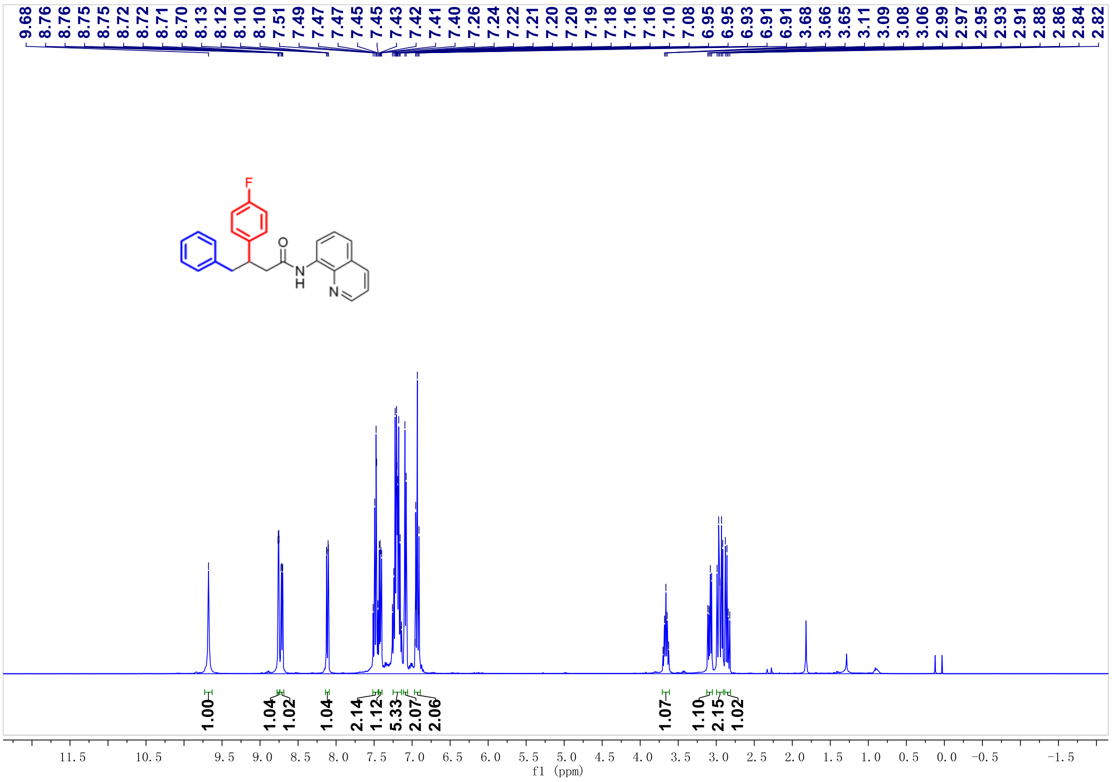

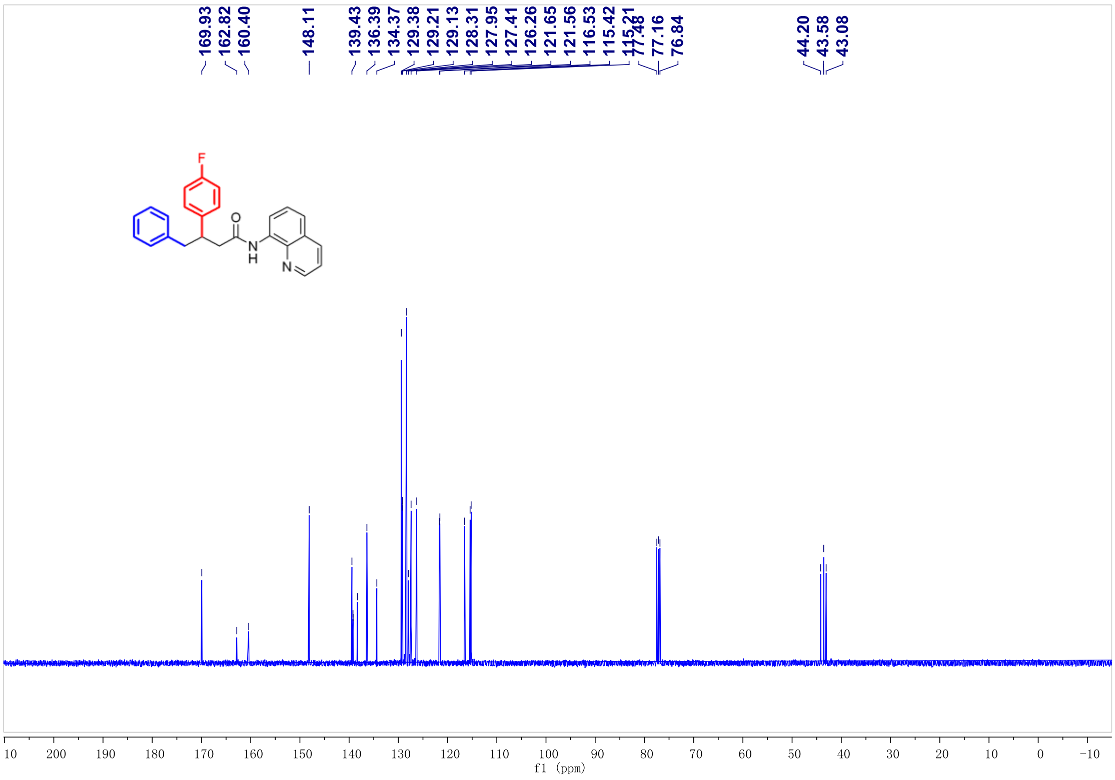

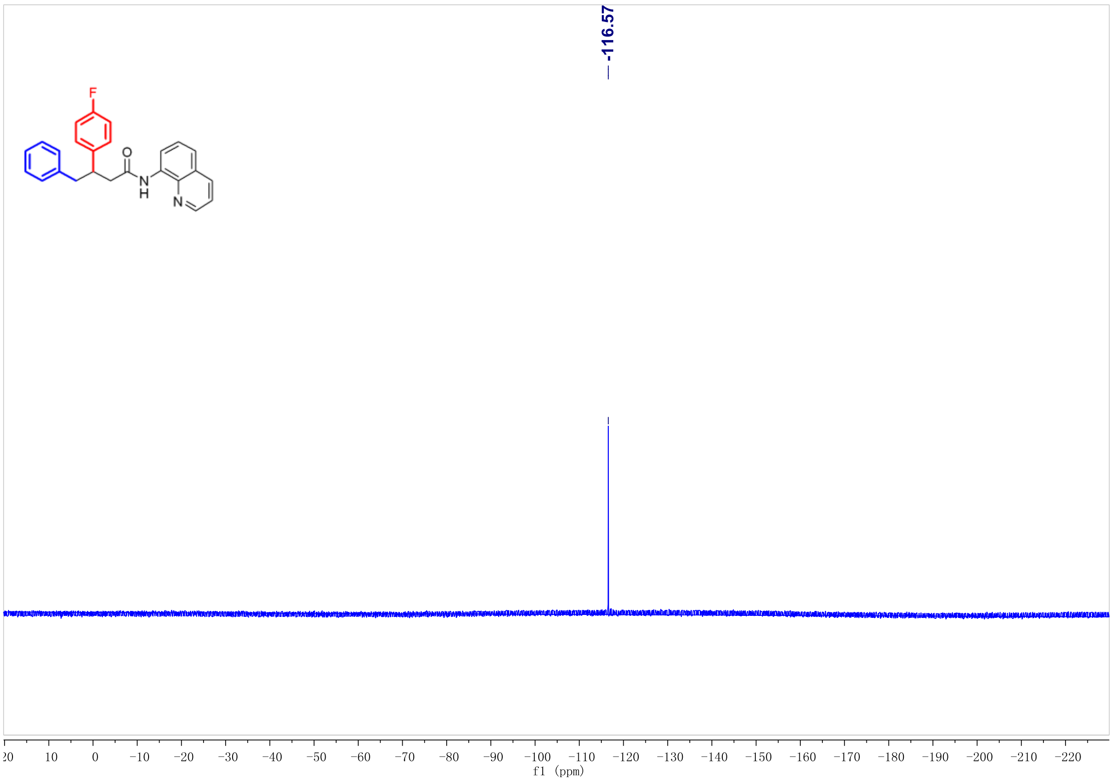

-116.57
F
O
N
H
N
f1 (ppm)

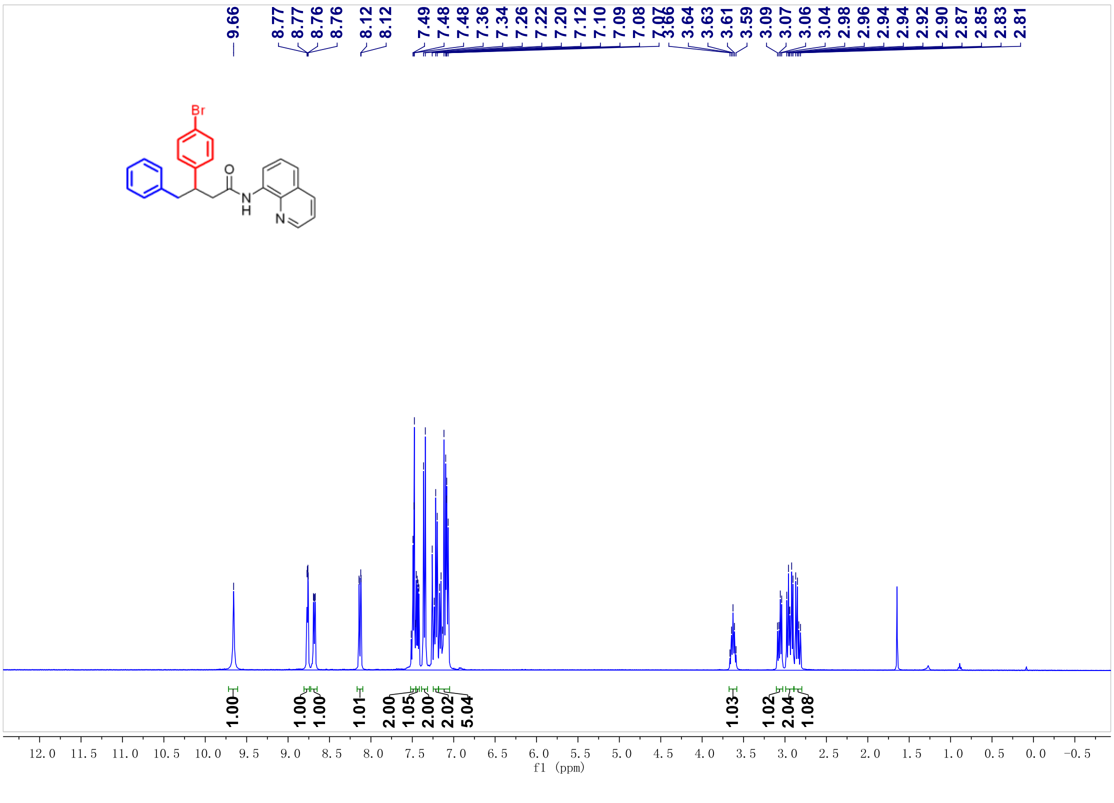

9.66
8.77
8.77
8.76
8.76
8.12
8.12
7.49
7.48
7.48
7.36
7.34
7.26
7.22
7.20
7.12
7.10
7.09
7.08
7.07
3.66
3.64
3.63
3.61
3.59
3.09
3.07
3.06
3.04
2.98
2.96
2.94
2.94
2.92
2.90
2.87
2.85
2.83
2.81
Br
O
N
H
N
1.00
1.00
1.00
1.01
2.00
1.05
2.00
2.02
5.04
1.03
1.02
2.04
1.08
f1 (ppm)

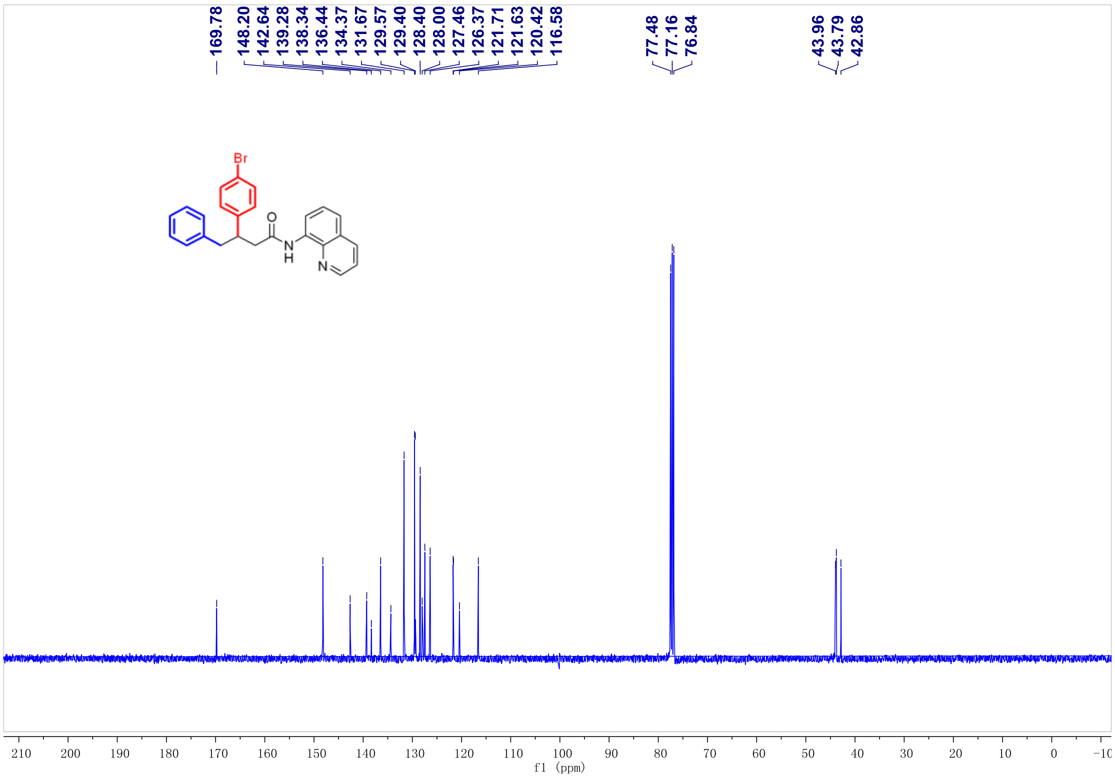
169.78
148.20
142.64
139.28
138.34
136.44
134.37
131.67
129.57
129.40
128.40
128.00
127.46
126.37
121.71
121.63
120.42
116.58
77.48
77.16
76.84
43.96
43.79
42.86
Br
O
N
H
N
f1 (ppm)

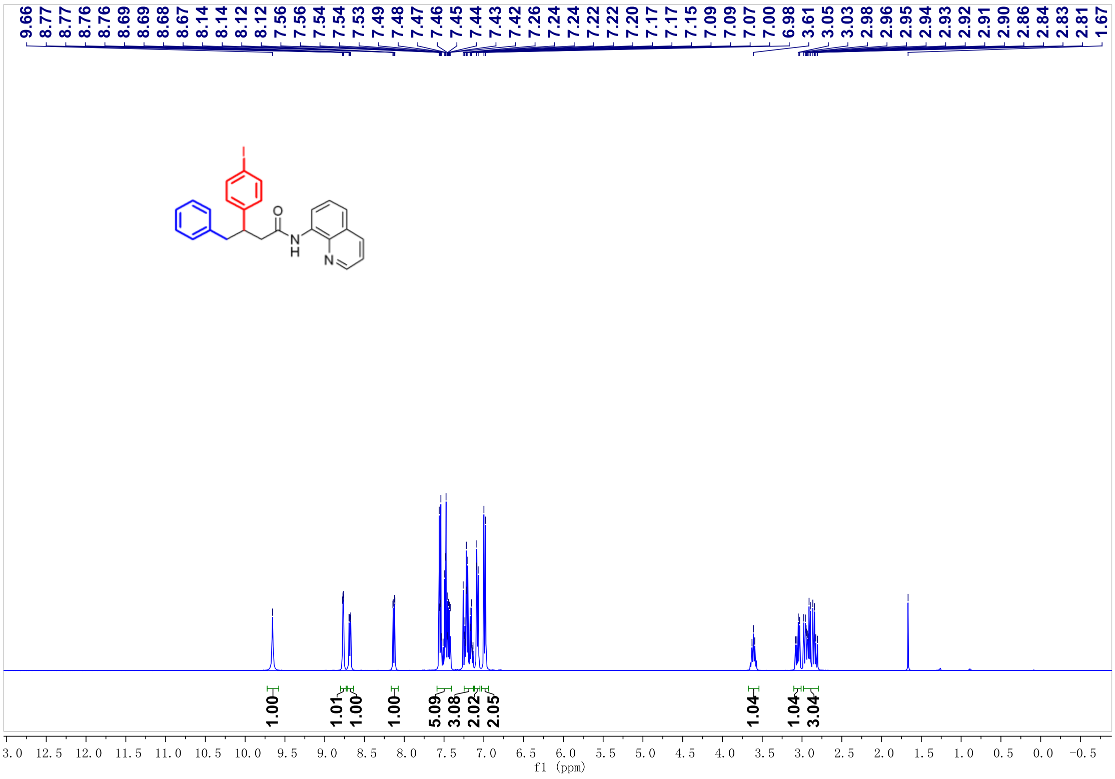
9.66
8.77
8.77
8.76
8.76
8.69
8.69
8.68
8.67
8.14
8.14
8.12
8.12
7.56
7.56
7.54
7.54
7.53
7.49
7.48
7.47
7.46
7.45
7.45
7.44
7.43
7.42
7.26
7.24
7.24
7.22
7.22
7.20
7.17
7.17
7.15
7.09
7.07
7.00
6.98
3.61
3.05
3.03
2.98
2.96
2.95
2.94
2.93
2.92
2.91
2.90
2.86
2.84
2.83
2.81
1.67
I
O
N
H
N
1.00
1.01
1.00
1.00
5.09
3.08
2.02
2.05
1.04
1.04
3.04
f1 (ppm)

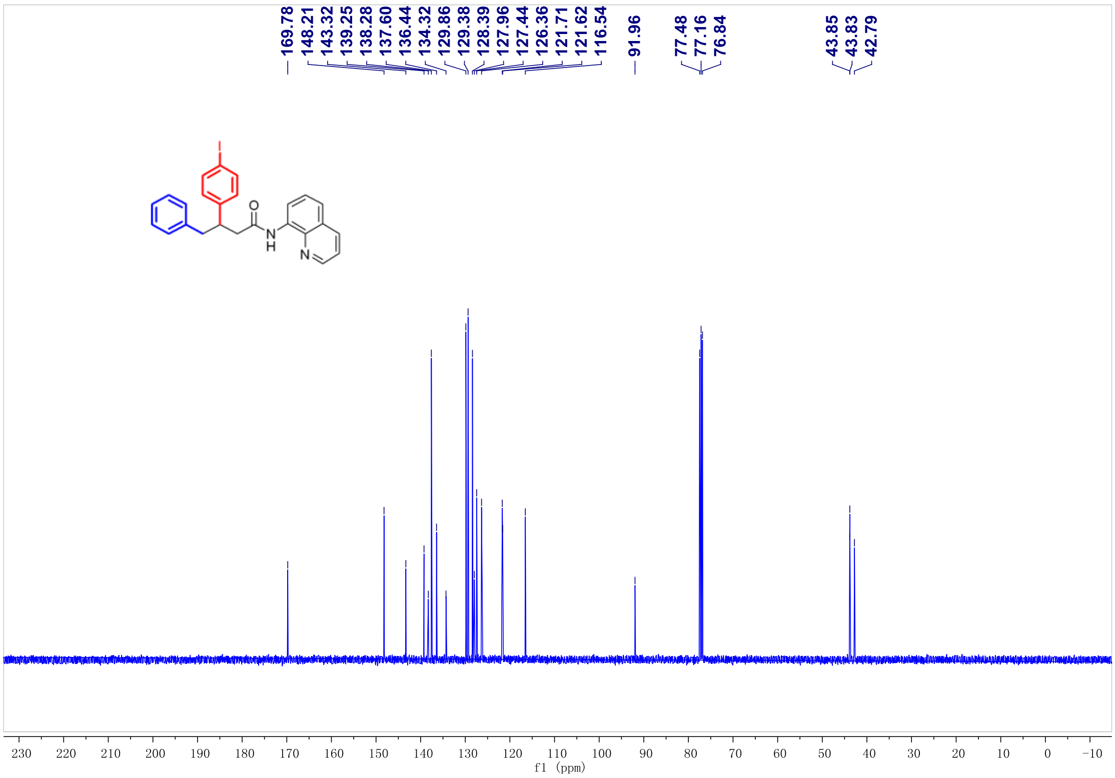
169.78
148.21
143.32
139.25
138.28
137.60
136.44
134.32
129.86
129.38
128.39
127.96
127.44
126.36
121.71
121.62
116.54
91.96
77.48
77.16
76.84
43.85
43.83
42.79
f1 (ppm)

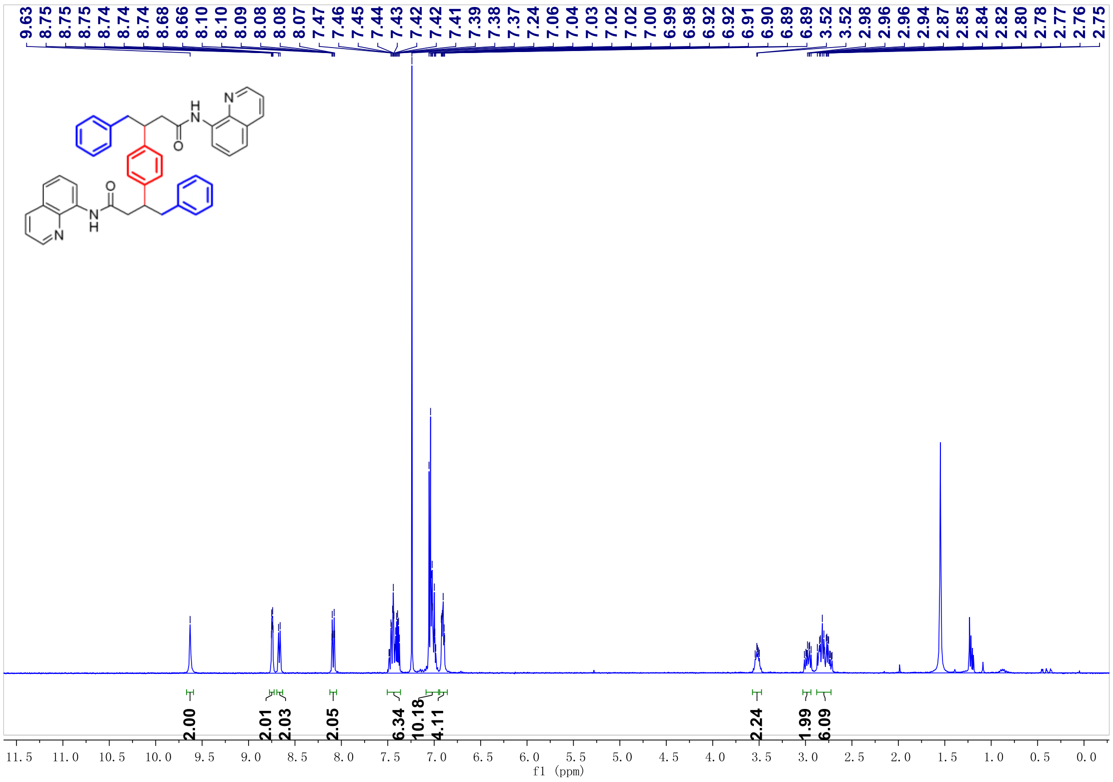
9.63
8.75
8.75
8.74
8.74
8.68
8.66
8.10
8.09
8.08
8.07
7.47
7.46
7.45
7.44
7.43
7.42
7.42
7.41
7.39
7.38
7.37
7.24
7.06
7.04
7.03
7.02
7.00
6.99
6.98
6.92
6.92
6.91
6.90
6.89
6.89
3.52
3.52
2.98
2.96
2.96
2.94
2.87
2.85
2.84
2.82
2.80
2.78
2.77
2.76
2.75
2.00
2.01
2.03
2.05
6.34
10.18
4.11
2.24
1.99
6.09
f1 (ppm)

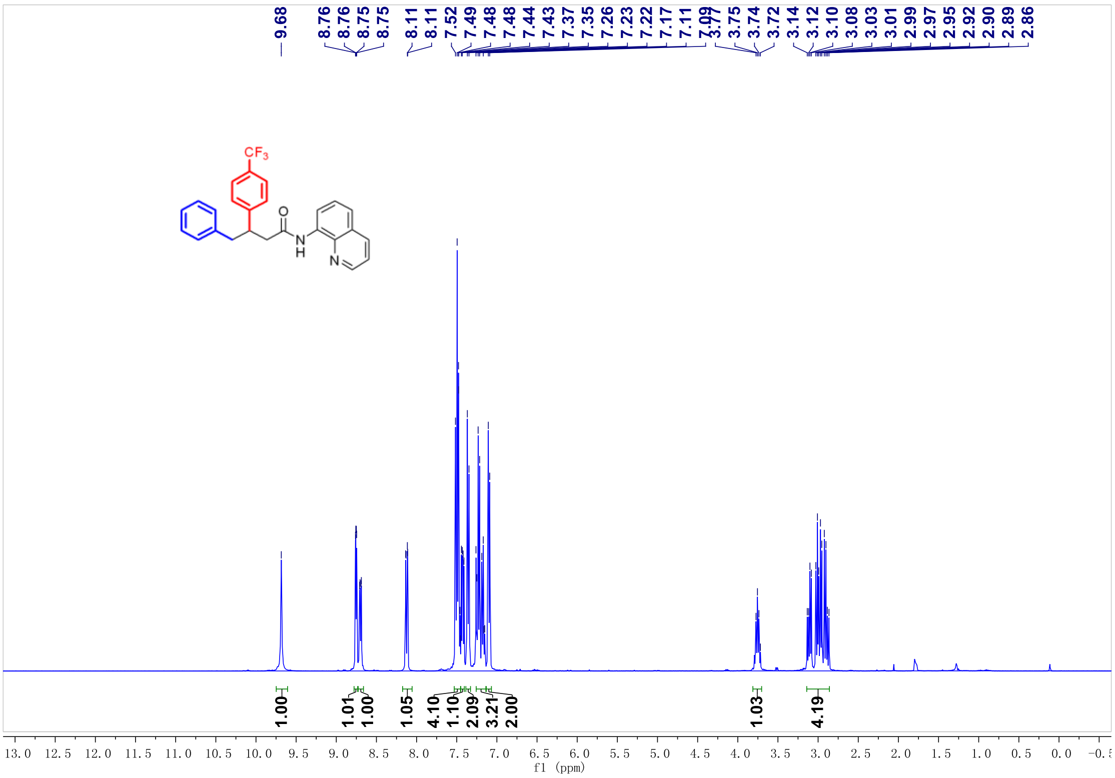
9.68
8.76
8.76
8.75
8.75
8.11
8.11
7.52
7.49
7.48
7.48
7.44
7.43
7.37
7.35
7.26
7.23
7.22
7.17
7.11
7.09
3.77
3.75
3.74
3.72
3.14
3.12
3.10
3.08
3.03
3.01
2.99
2.97
2.95
2.92
2.90
2.89
2.86
CF3
O
N
H
N
1.00
1.01
1.00
1.05
4.10
1.10
2.09
3.21
2.00
1.03
4.19
f1 (ppm)

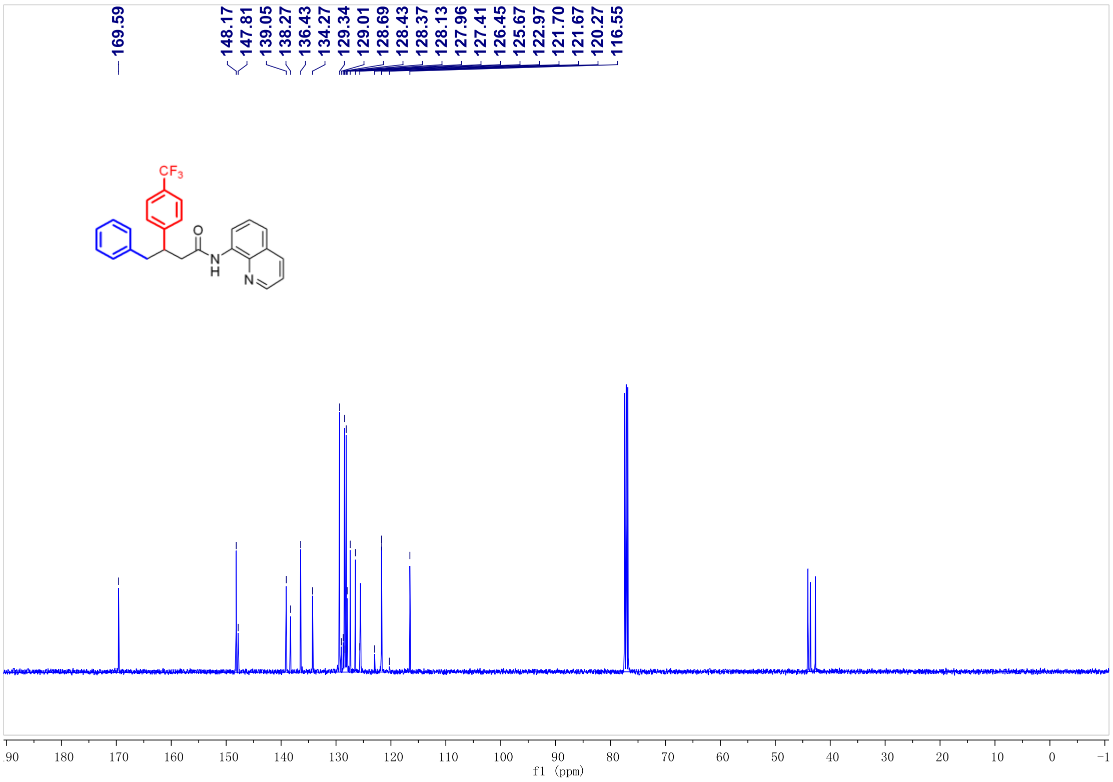
169.59
148.17
147.81
139.05
138.27
136.43
134.27
129.34
129.01
128.69
128.43
128.37
128.13
127.96
127.41
126.45
125.67
122.97
121.70
121.67
120.27
116.55
CF3
O
N
H
N
f1 (ppm)

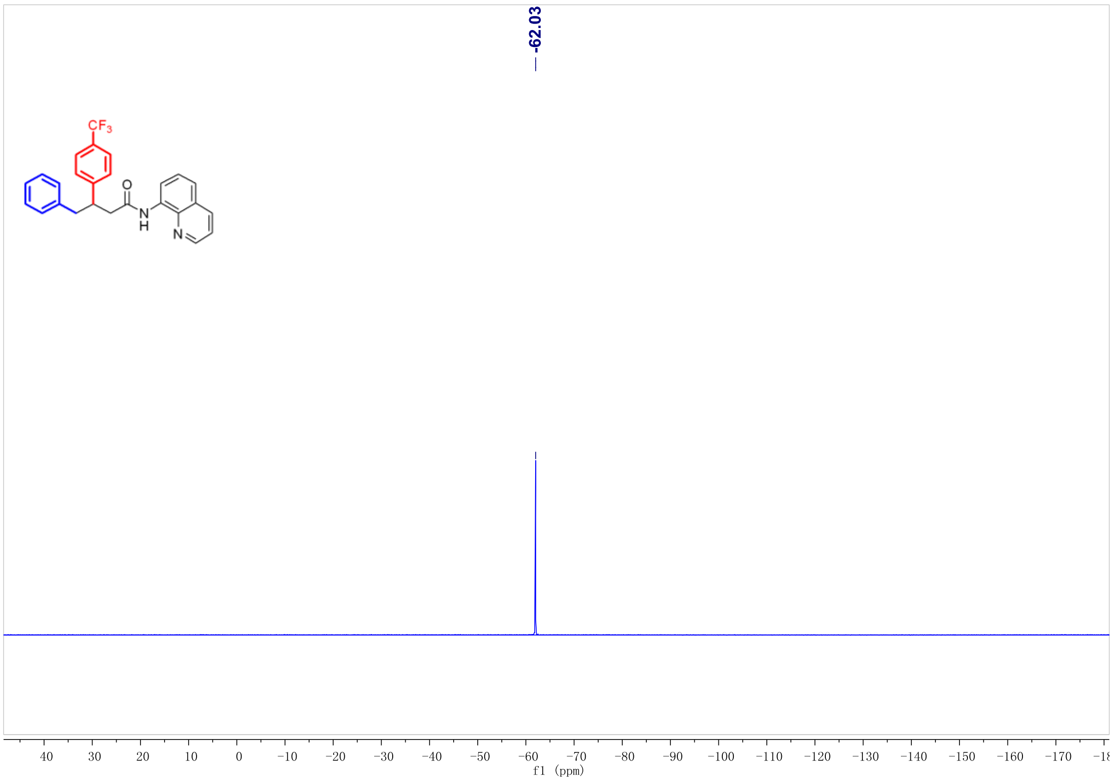

-62.03
CF3
O
N
H
N
f1 (ppm)

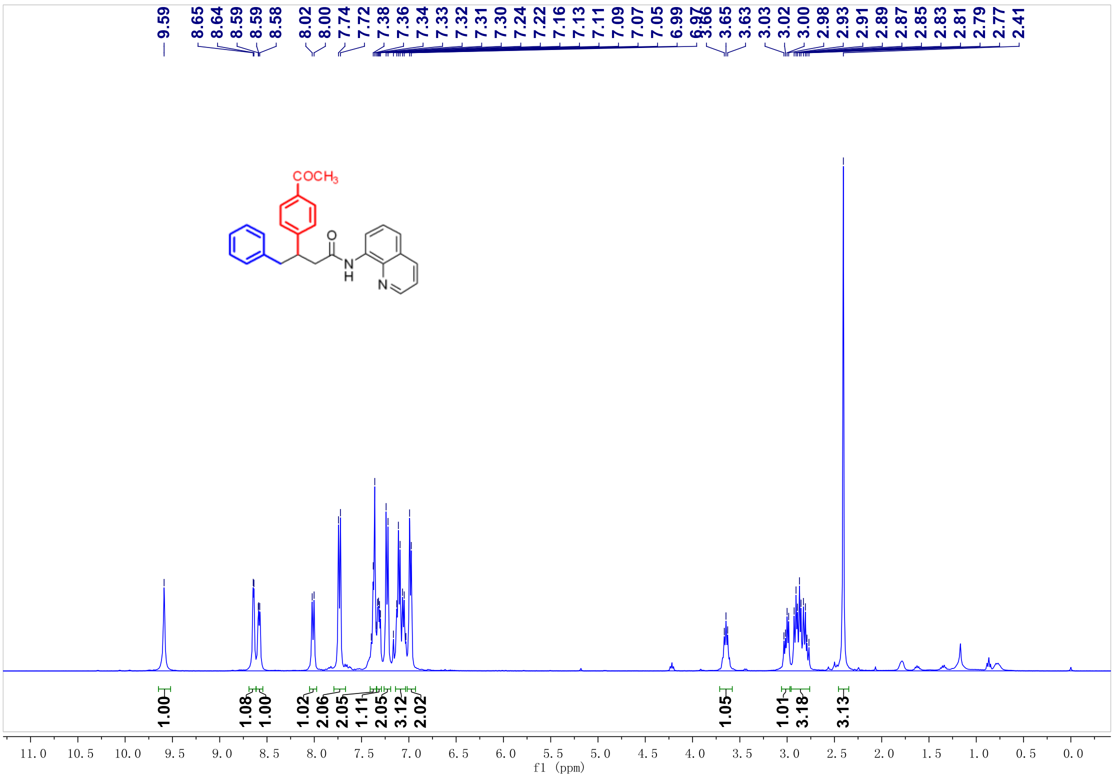

9.59
8.65
8.64
8.59
8.59
8.58
8.02
8.00
7.74
7.72
7.38
7.36
7.34
7.33
7.32
7.31
7.30
7.24
7.22
7.16
7.13
7.11
7.09
7.07
7.05
6.99
6.97
3.66
3.65
3.63
3.03
3.02
3.00
2.98
2.93
2.91
2.89
2.87
2.85
2.83
2.81
2.79
2.77
2.41
COCH3
O
N
H
N
1.00
1.08
1.00
1.02
2.06
2.05
1.11
2.05
3.12
2.02
1.05
1.01
3.18
3.13
f1 (ppm)

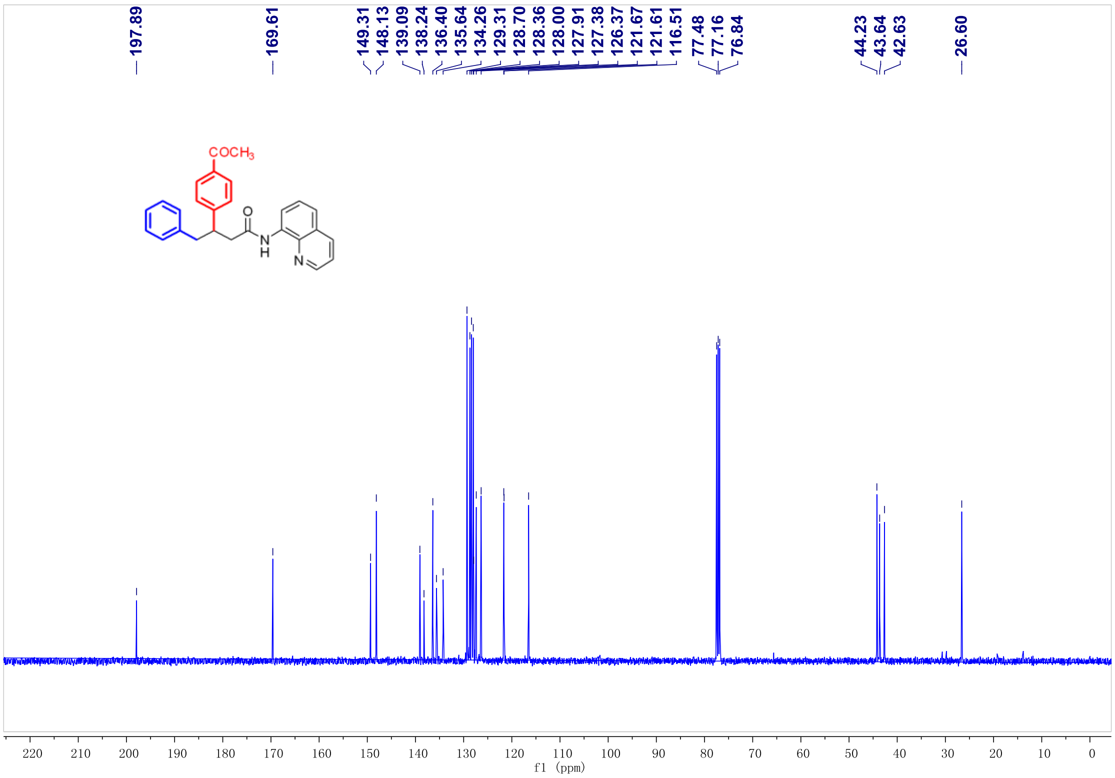
197.89
169.61
149.31
148.13
139.09
138.24
136.40
135.64
134.26
129.31
128.70
128.36
128.00
127.91
127.38
126.37
121.67
121.61
116.51
77.48
77.16
76.84
44.23
43.64
42.63
26.60
COCH3
O
N
H
N
f1 (ppm)

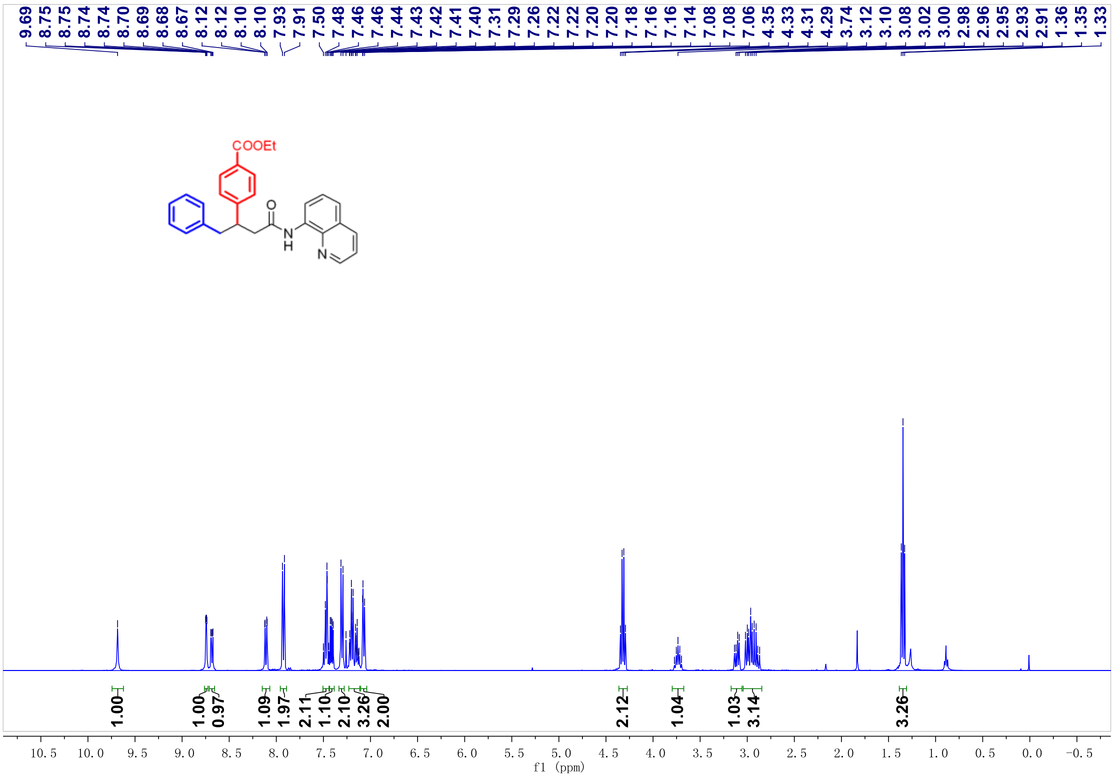
9.69
8.75
8.75
8.74
8.74
8.70
8.69
8.68
8.67
8.12
8.12
8.10
8.10
7.93
7.91
7.50
7.48
7.46
7.46
7.44
7.43
7.42
7.42
7.41
7.40
7.31
7.29
7.26
7.22
7.22
7.20
7.20
7.18
7.16
7.16
7.14
7.08
7.08
7.06
4.35
4.33
4.31
4.29
3.74
3.12
3.10
3.08
3.02
3.00
2.98
2.96
2.95
2.93
2.91
1.36
1.35
1.33
COOEt
O
N
H
N
1.00
1.00
0.97
1.09
1.97
2.11
1.10
2.10
3.26
2.00
2.12
1.04
1.03
3.14
3.26
f1 (ppm)

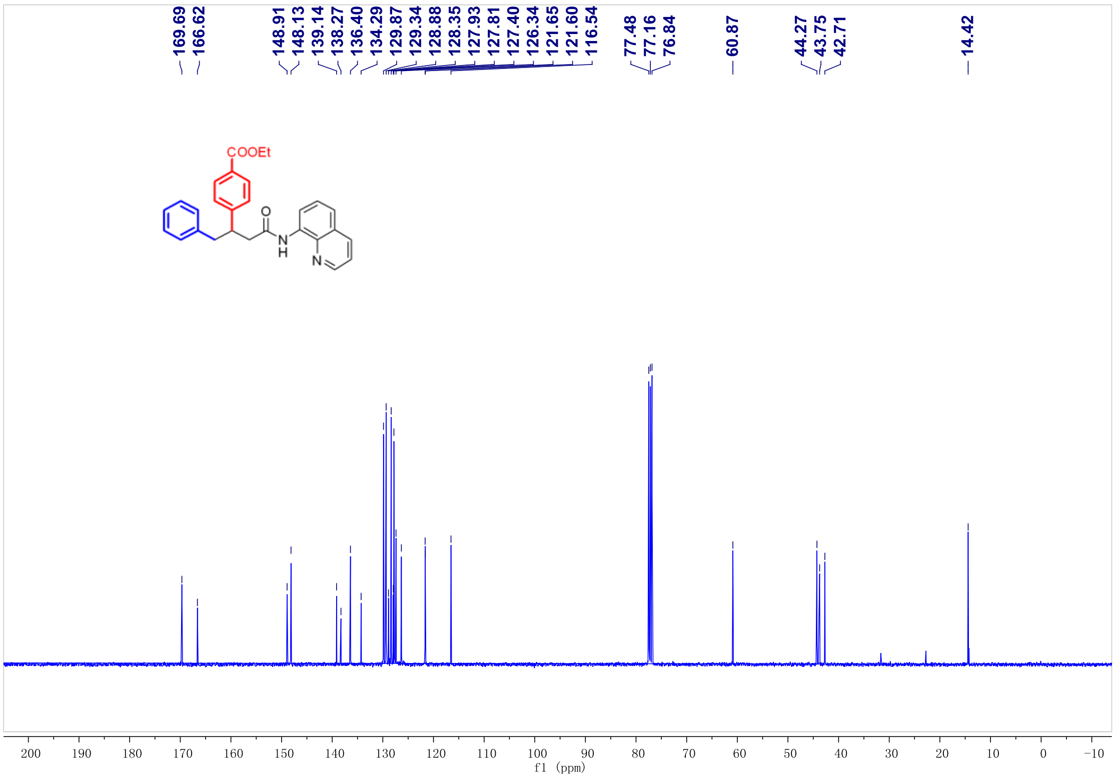
169.69
166.62
148.91
148.13
139.14
138.27
136.40
134.29
129.87
129.34
128.88
128.35
127.93
127.81
127.40
126.34
121.65
121.60
116.54
77.48
77.16
76.84
60.87
44.27
43.75
42.71
14.42
COOEt
O
N
H
N
f1 (ppm)

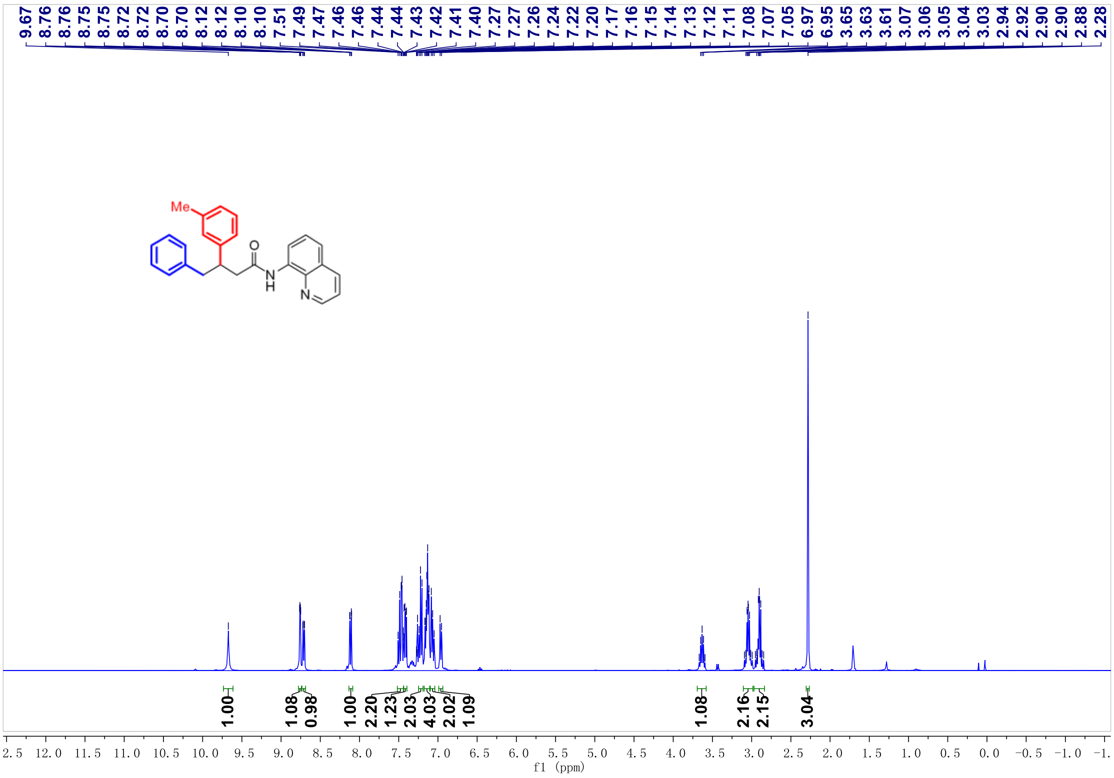
9.67
8.76
8.76
8.75
8.72
8.72
8.70
8.70
8.12
8.10
8.10
7.51
7.49
7.47
7.46
7.46
7.44
7.44
7.43
7.42
7.41
7.40
7.27
7.27
7.26
7.24
7.22
7.20
7.17
7.16
7.15
7.14
7.13
7.12
7.11
7.08
7.07
7.05
6.97
6.95
3.65
3.63
3.61
3.07
3.06
3.05
3.04
3.03
2.94
2.92
2.90
2.90
2.88
2.28
Me
O
N
H
N
1.00
1.08
0.98
1.00
2.20
1.23
2.03
4.03
2.02
1.09
1.08
2.16
2.15
3.04
f1 (ppm)

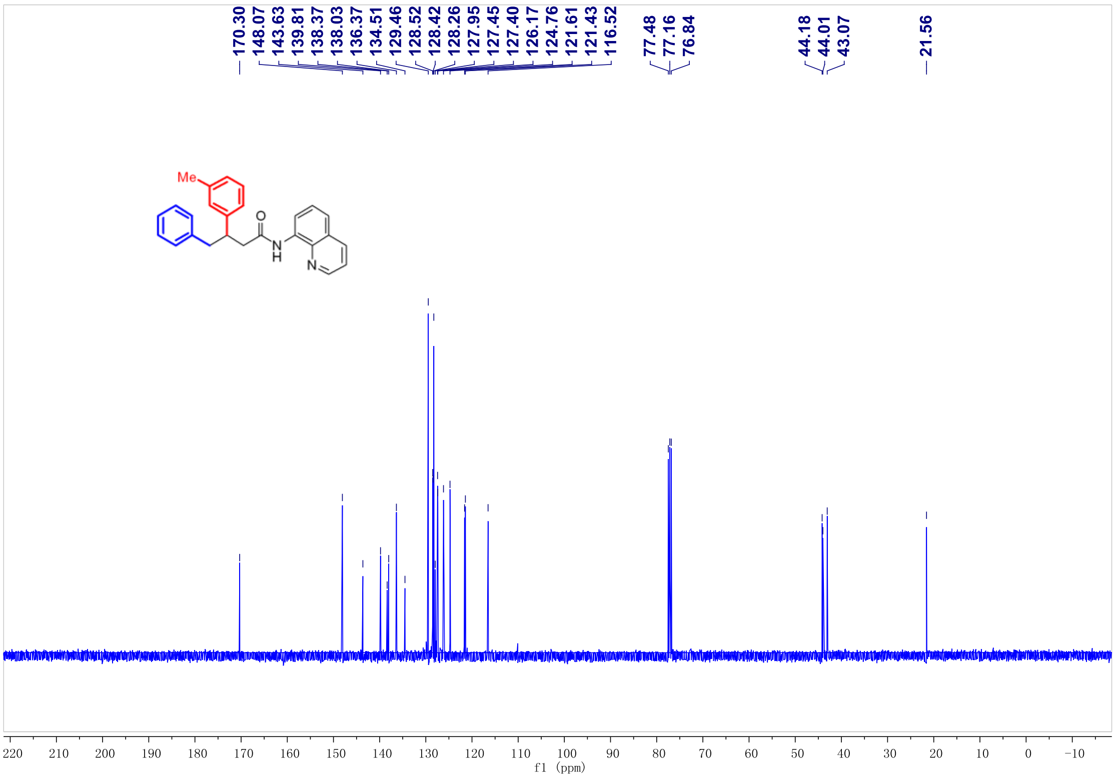
170.30
148.07
143.63
139.81
138.37
138.03
136.37
134.51
129.46
128.52
128.42
128.26
127.95
127.45
127.40
126.17
124.76
121.61
121.43
116.52
77.48
77.16
76.84
44.18
44.01
43.07
21.56
Me
O
N
H
N
f1 (ppm)

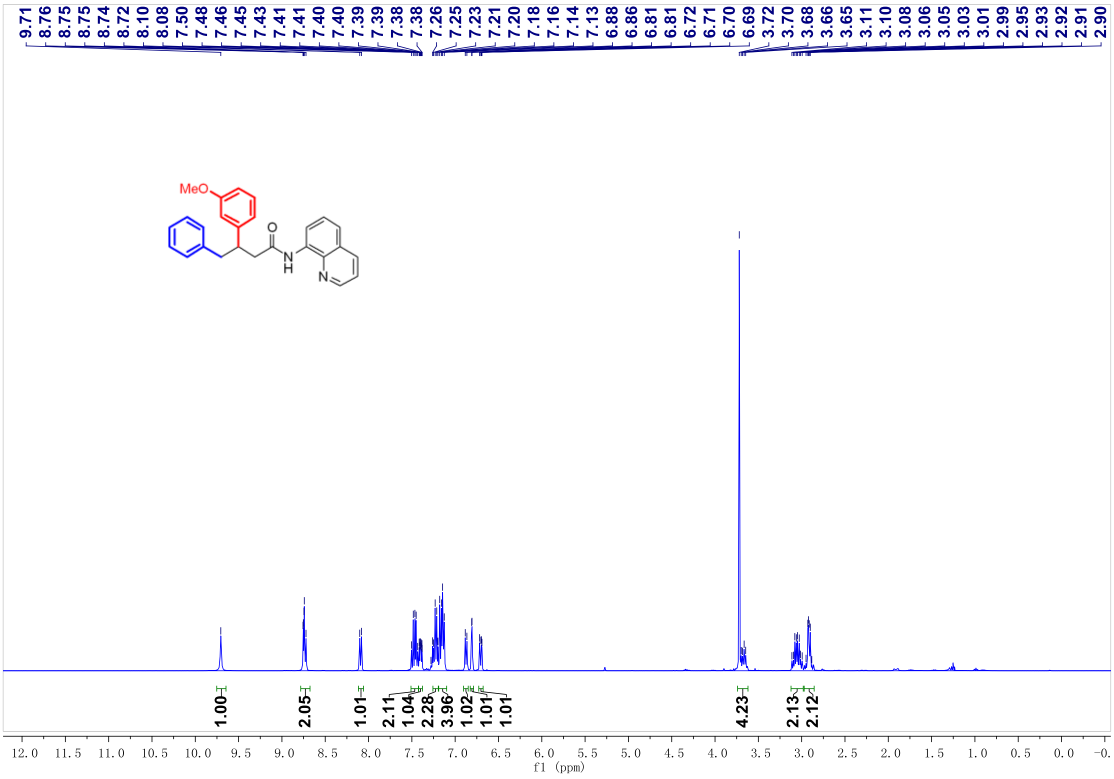
9.71
8.76
8.75
8.75
8.74
8.72
8.10
8.08
7.50
7.48
7.46
7.45
7.43
7.41
7.41
7.40
7.40
7.39
7.39
7.38
7.38
7.26
7.25
7.23
7.21
7.20
7.18
7.16
7.14
7.13
6.88
6.86
6.81
6.81
6.72
6.71
6.70
6.69
3.72
3.70
3.68
3.66
3.65
3.11
3.10
3.08
3.06
3.05
3.03
3.01
2.99
2.95
2.93
2.92
2.91
2.90
MeO
O
N
H
N
1.00
2.05
1.01
2.11
1.04
2.28
3.96
1.02
1.01
1.01
4.23
2.13
2.12
f1 (ppm)

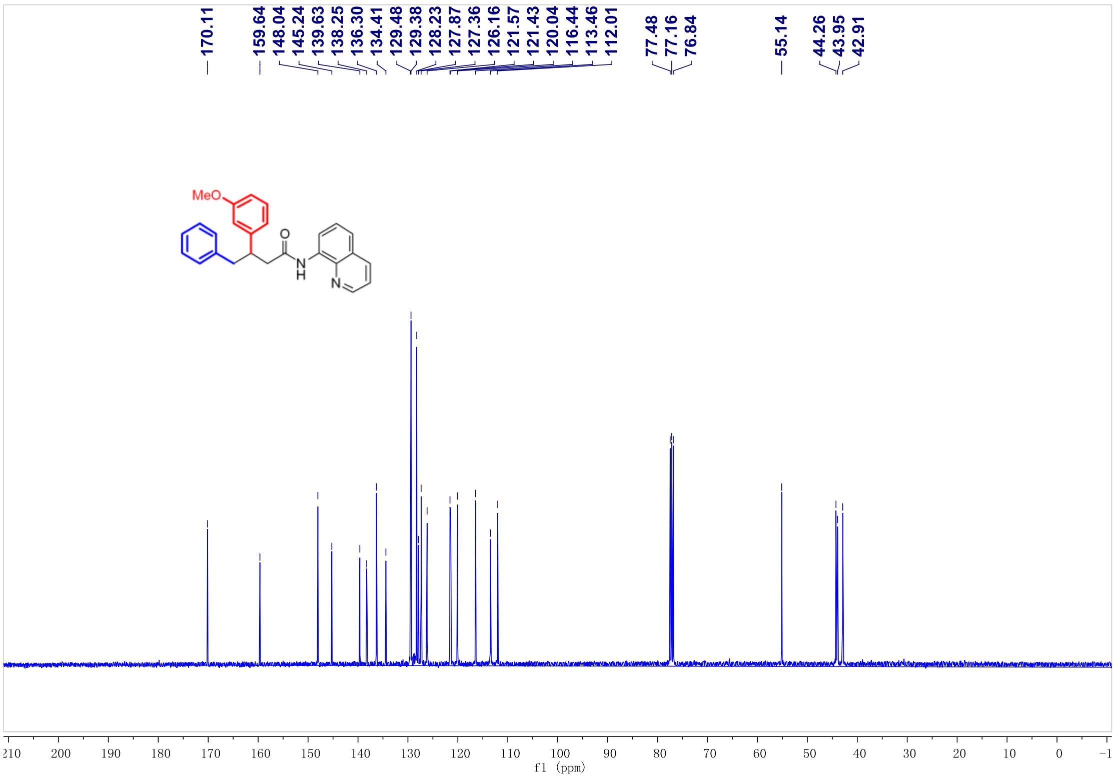
170.11
159.64
148.04
145.24
139.63
138.25
136.30
134.41
129.48
129.38
128.23
127.87
127.36
126.16
121.57
121.43
120.04
116.44
113.46
112.01
77.48
77.16
76.84
55.14
44.26
43.95
42.91
MeO
O
N
H
N
f1 (ppm)

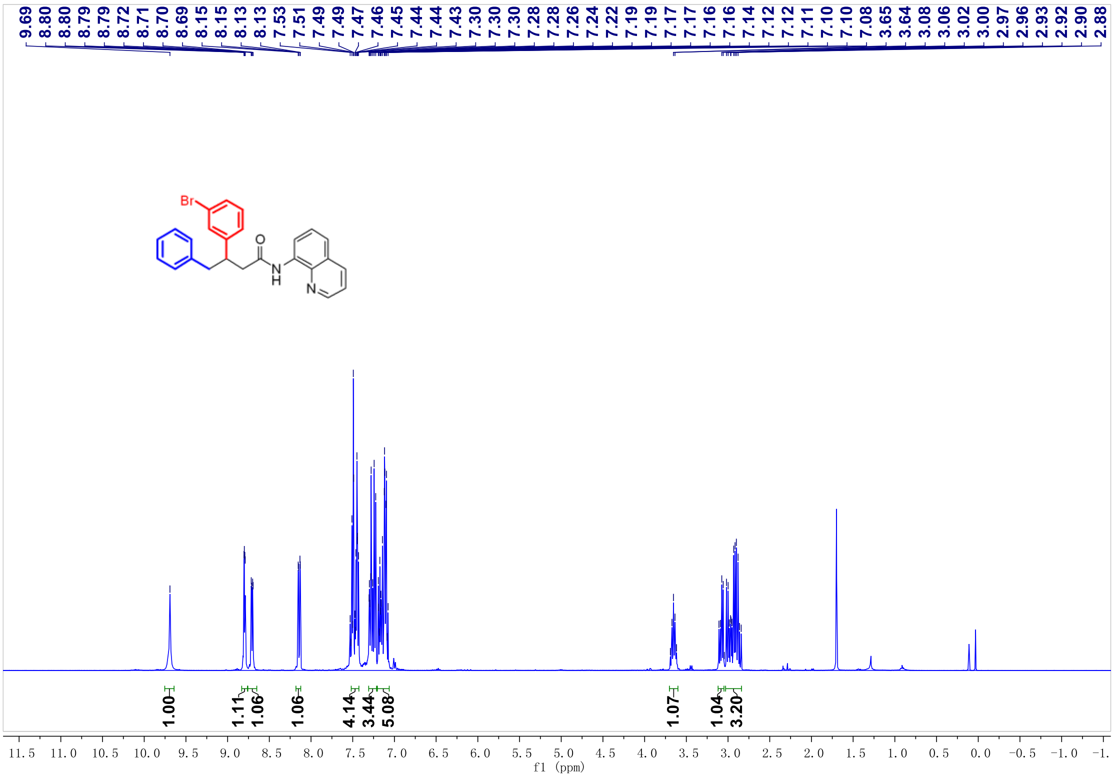
9.69
8.80
8.80
8.79
8.72
8.71
8.70
8.69
8.15
8.13
8.13
7.53
7.51
7.49
7.49
7.47
7.46
7.45
7.44
7.44
7.43
7.30
7.30
7.28
7.26
7.24
7.22
7.19
7.19
7.17
7.17
7.16
7.16
7.14
7.12
7.12
7.11
7.10
7.10
7.08
3.65
3.64
3.08
3.06
3.02
3.00
2.97
2.96
2.93
2.92
2.90
2.88
Br
O
N
H
N
1.00
1.11
1.06
1.06
4.14
3.44
5.08
1.07
1.04
3.20
f1 (ppm)

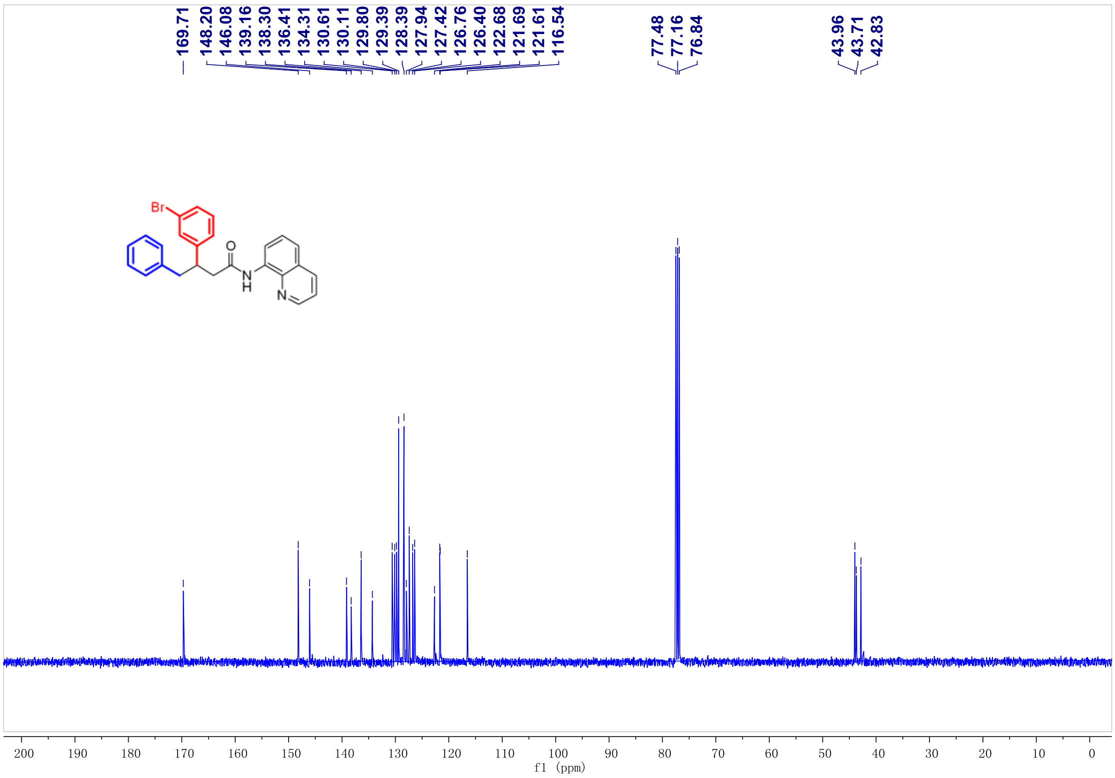
169.71
148.20
146.08
139.16
138.30
136.41
134.31
130.61
130.11
129.80
129.39
128.39
127.94
127.42
126.76
126.40
122.68
121.69
121.61
116.54
77.48
77.16
76.84
43.96
43.71
42.83
Br
O
N
H
N
f1 (ppm)

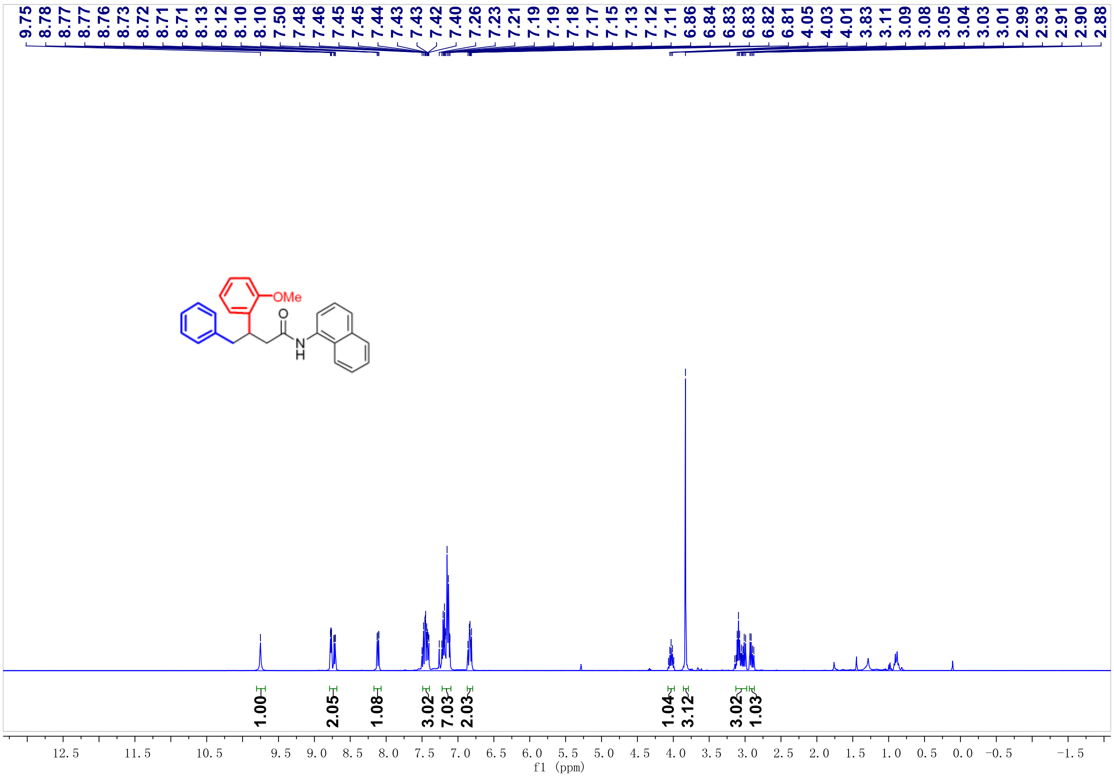
9.75
8.78
8.77
8.76
8.73
8.72
8.71
8.71
8.13
8.12
8.10
8.10
7.50
7.48
7.46
7.45
7.44
7.43
7.43
7.42
7.40
7.26
7.23
7.21
7.19
7.18
7.17
7.15
7.13
7.12
7.11
6.86
6.84
6.83
6.83
6.82
6.81
4.05
4.03
4.01
3.83
3.11
3.09
3.08
3.05
3.04
3.03
3.01
2.99
2.93
2.91
2.90
2.88
OMe
O
N
H
1.00
2.05
1.08
3.02
7.03
2.03
1.04
3.12
3.02
1.03
f1 (ppm)

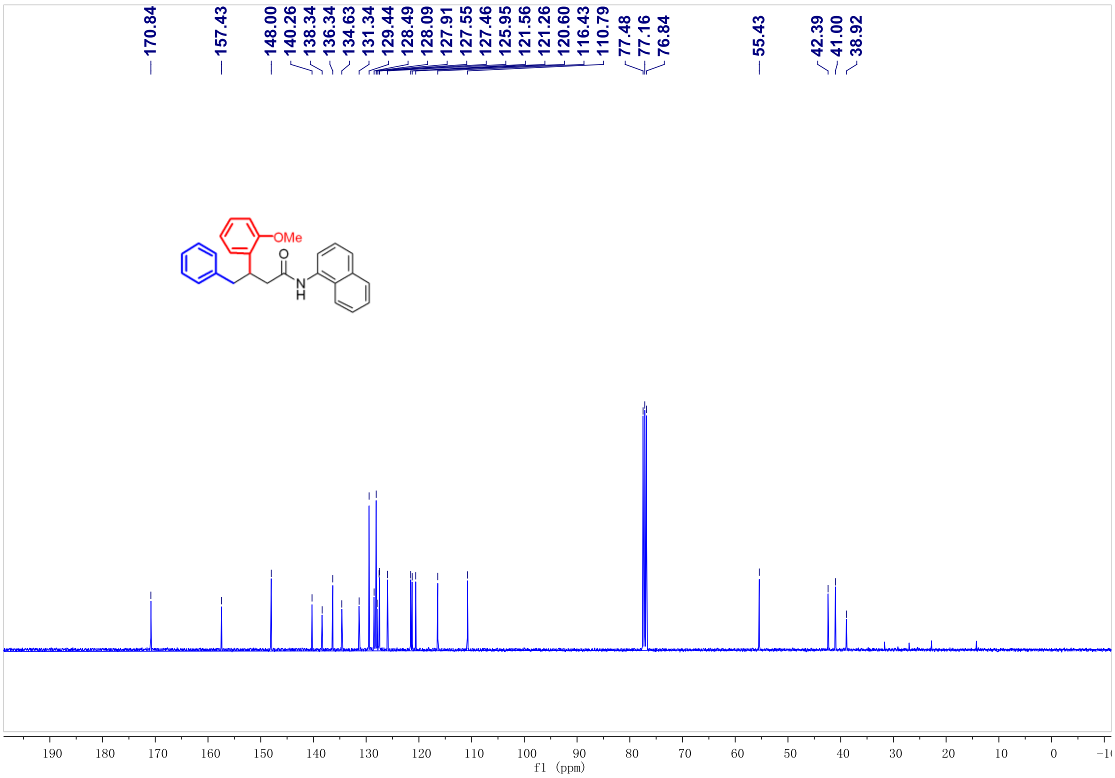
170.84
157.43
148.00
140.26
138.34
136.34
134.63
131.34
129.44
128.49
128.09
127.91
127.55
127.46
125.95
121.56
121.26
120.60
116.43
110.79
77.48
77.16
76.84
55.43
42.39
41.00
38.92
OMe
O
N
H
f1 (ppm)

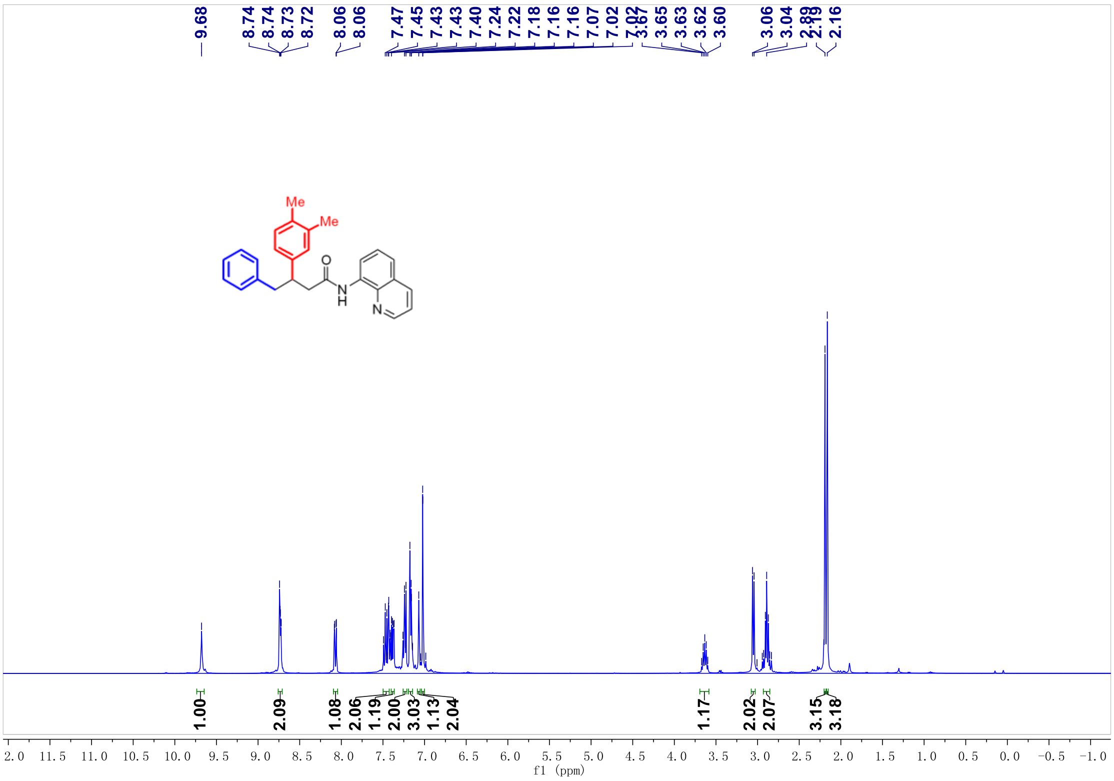
9.68
8.74
8.74
8.73
8.72
8.06
8.06
7.47
7.45
7.43
7.43
7.40
7.24
7.22
7.18
7.16
7.16
7.07
7.02
7.02
3.67
3.65
3.63
3.62
3.60
3.06
3.04
2.89
2.19
2.16
Me
Me
O
N
H
N
1.00
2.09
1.08
2.06
1.19
2.00
3.03
1.13
2.04
1.17
2.02
2.07
3.15
3.18
f1 (ppm)

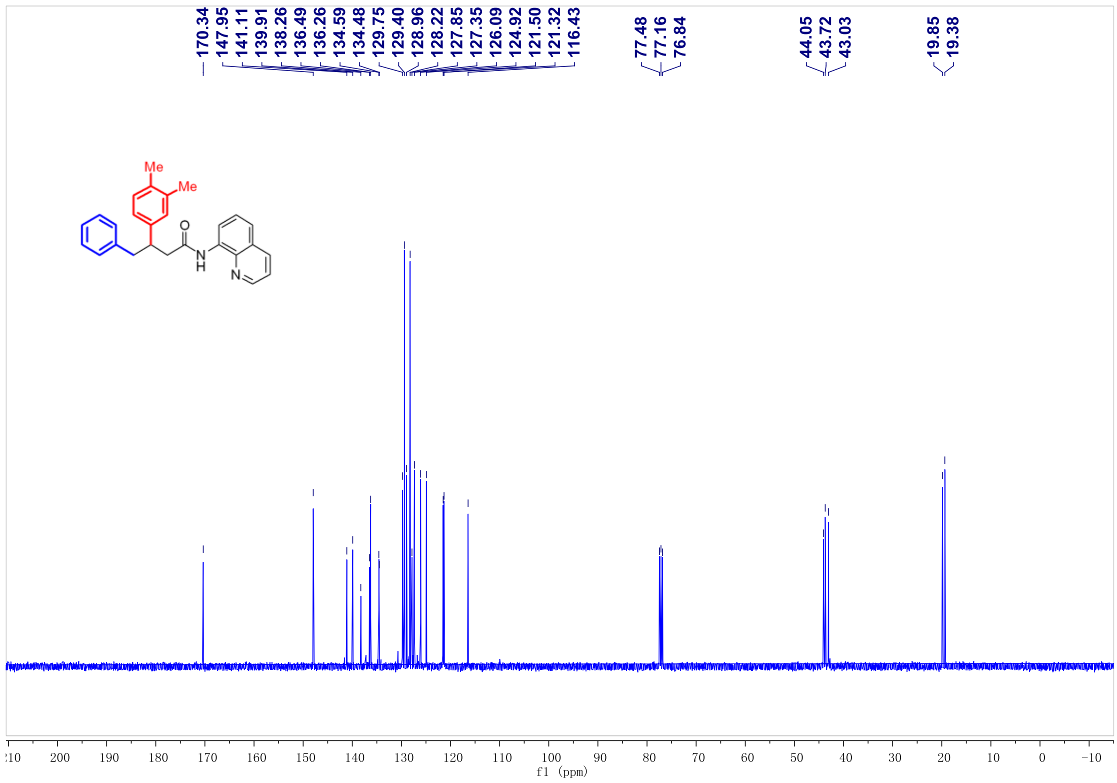
170.34
147.95
141.11
139.91
138.26
136.49
136.26
134.59
134.48
129.75
129.40
128.96
128.22
127.85
127.35
126.09
124.92
121.50
121.32
116.43
77.48
77.16
76.84
44.05
43.72
43.03
19.85
19.38
Me
Me
O
N
H
N
f1 (ppm)

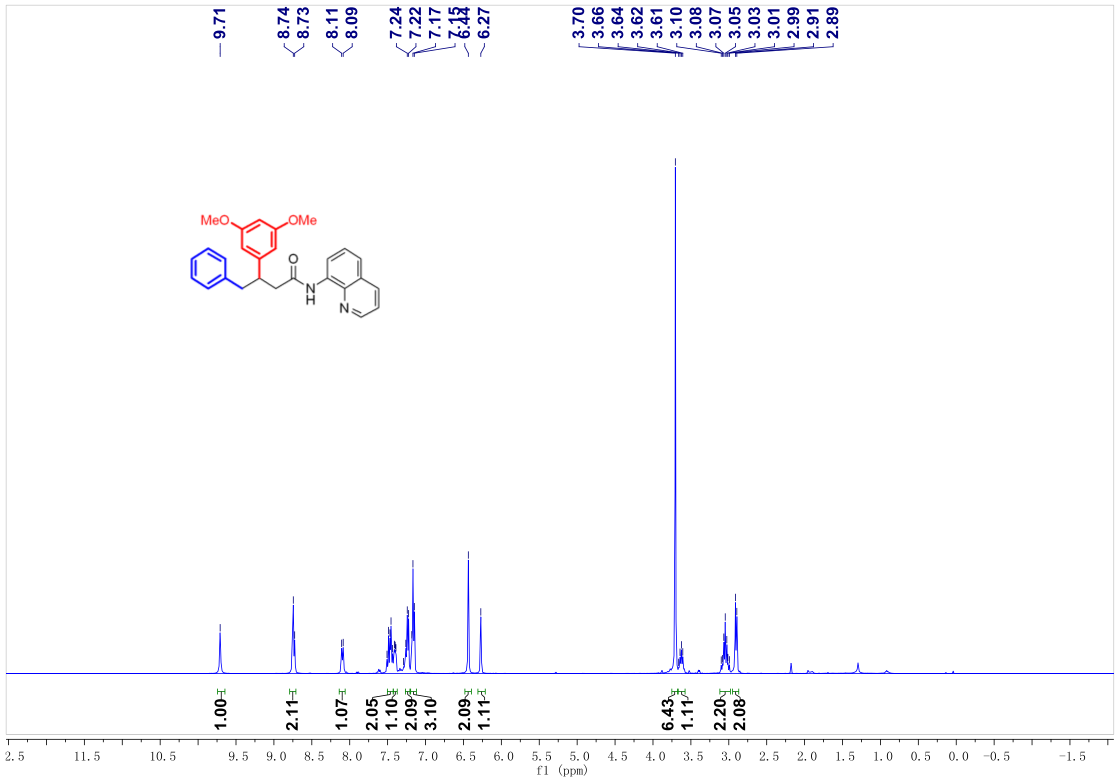
9.71
8.74
8.73
8.11
8.09
7.24
7.22
7.17
7.15
6.44
6.27
3.70
3.66
3.64
3.62
3.61
3.10
3.08
3.07
3.05
3.03
3.01
2.99
2.91
2.89
MeO
OMe
O
N
H
N
1.00
2.11
1.07
2.05
1.10
2.09
3.10
2.09
1.11
6.43
1.11
2.20
2.08
f1 (ppm)

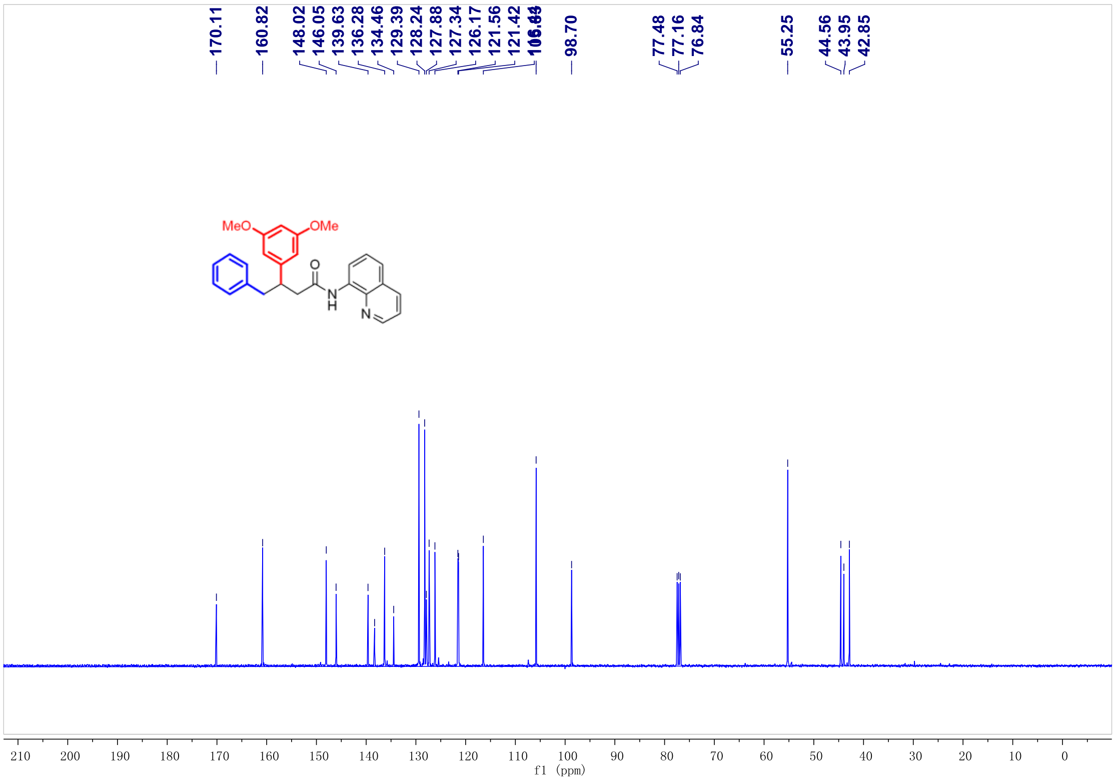

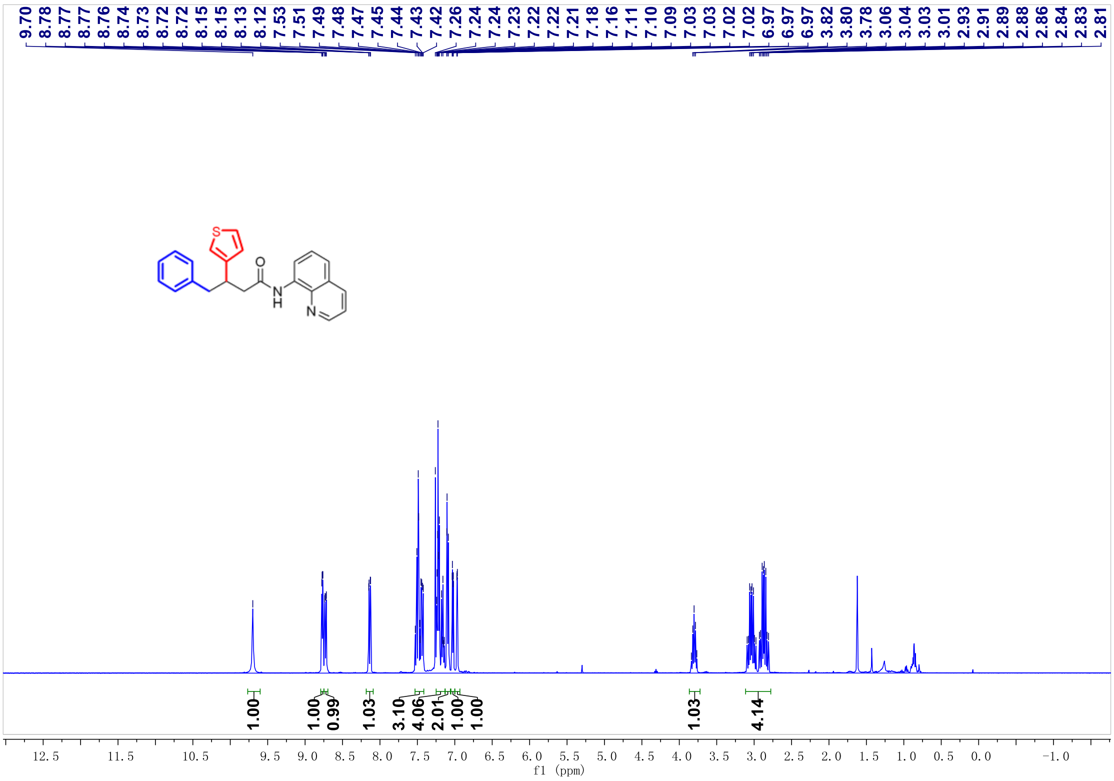

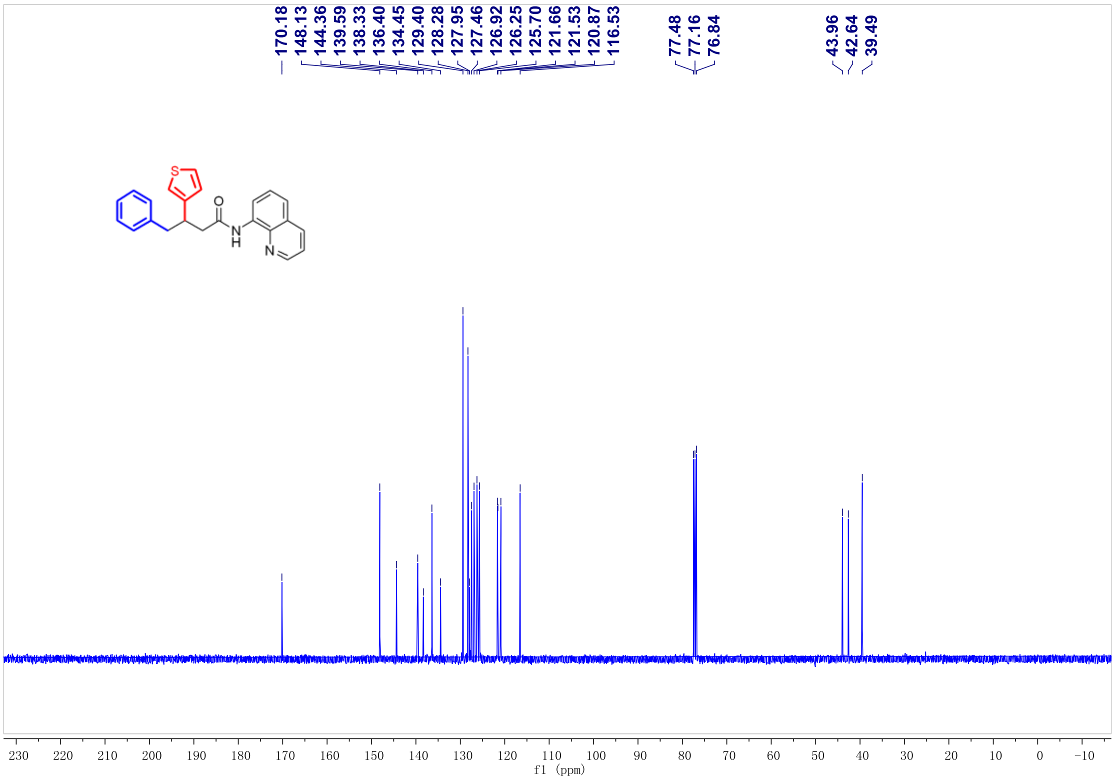

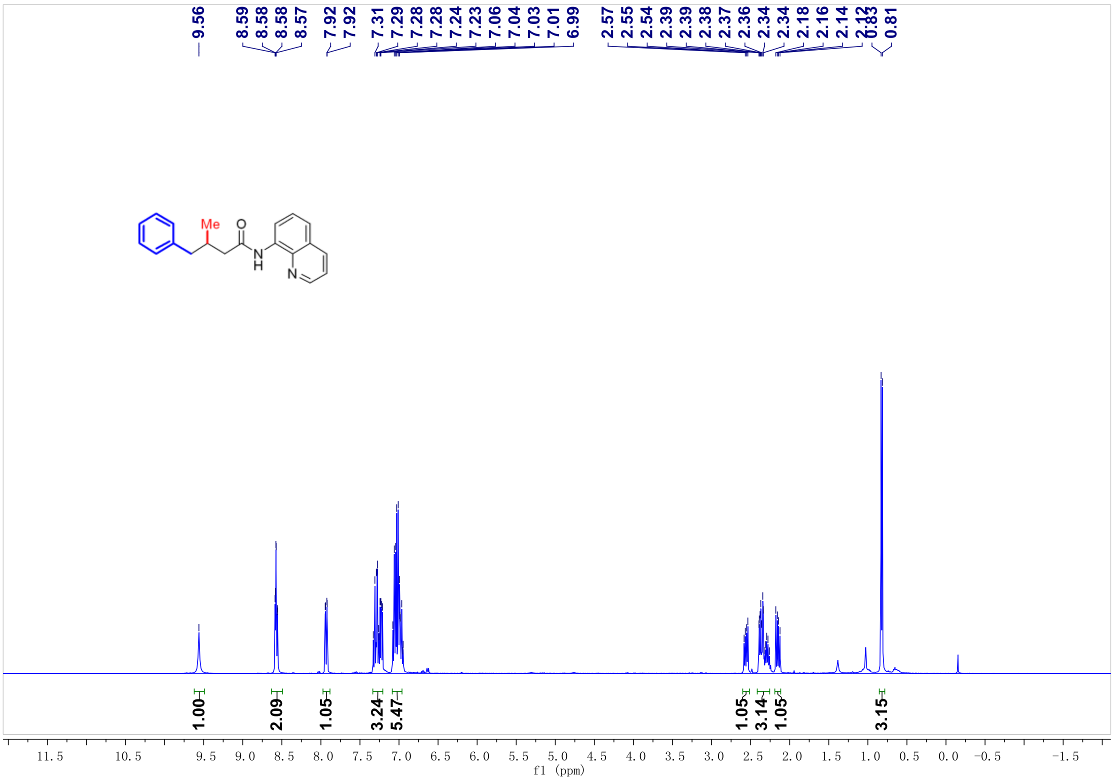

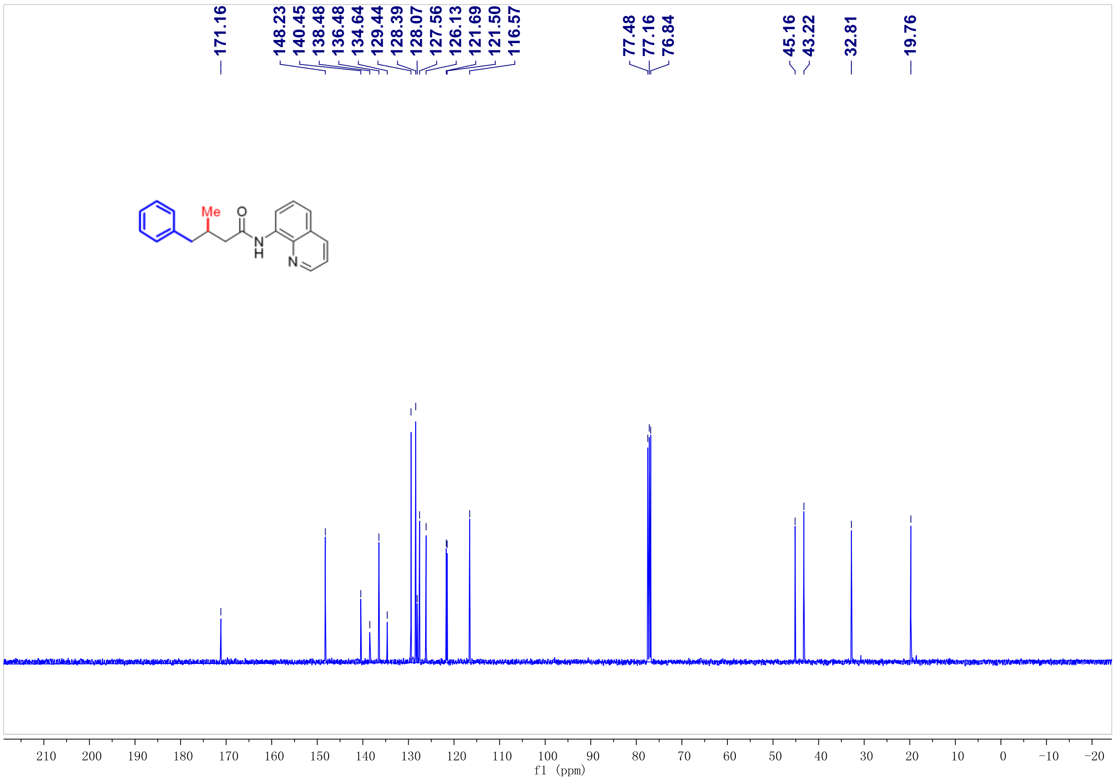
171.16
148.23
140.45
138.48
136.48
134.64
129.44
128.39
128.07
127.56
126.13
121.69
121.50
116.57
77.48
77.16
76.84
45.16
43.22
32.81
19.76
Me
O
N
H
N
f1 (ppm)

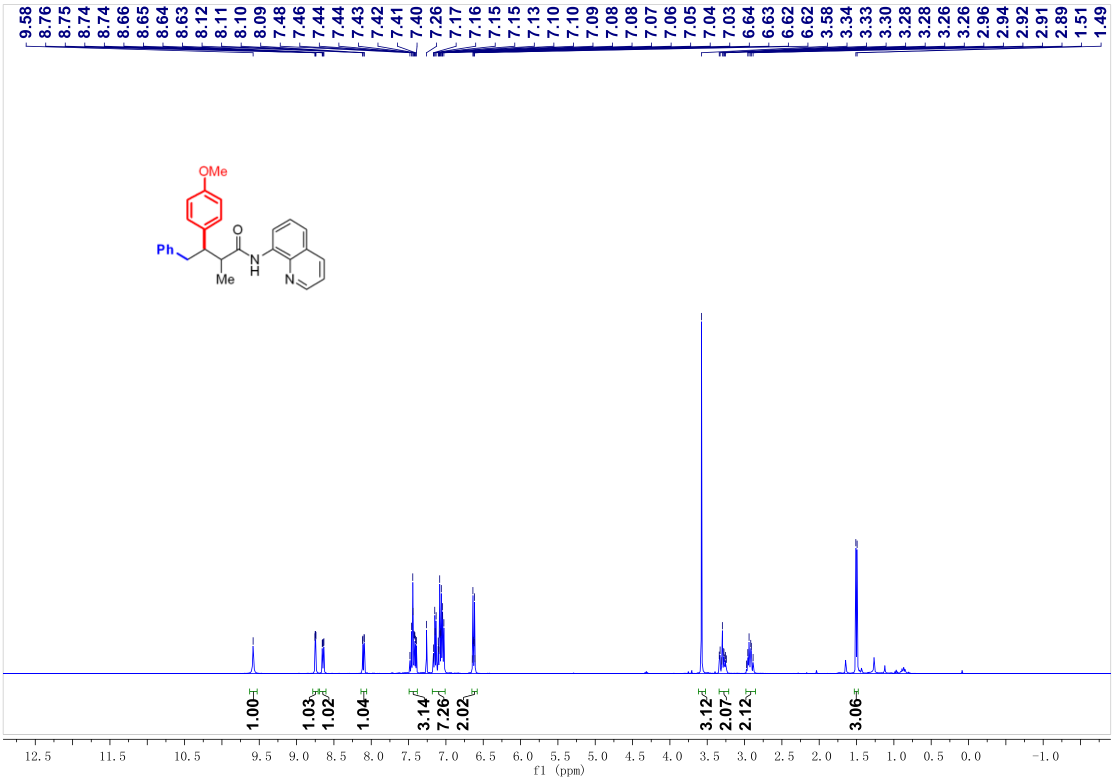
OMe
Ph
Me
O
N
H
N
1.00
1.03
1.02
1.04
3.14
7.26
2.02
3.12
2.07
2.12
3.06
f1 (ppm)

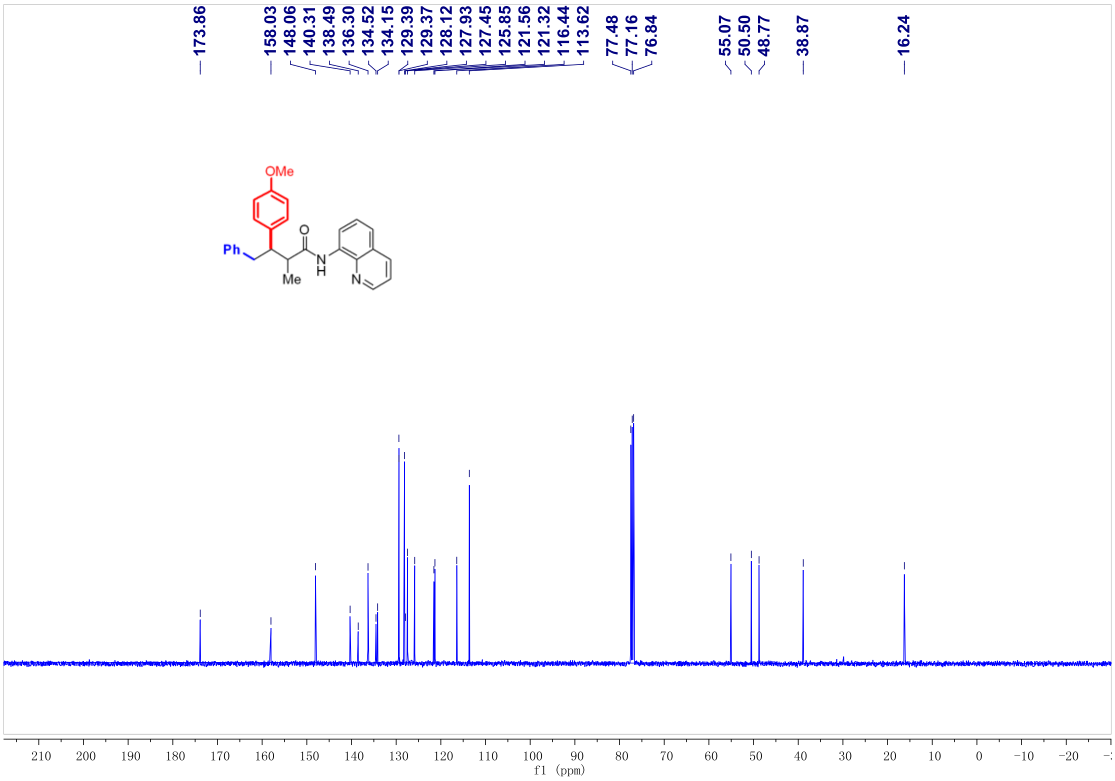
173.86
158.03
148.06
140.31
138.49
136.30
134.52
134.15
129.39
129.37
128.12
127.93
127.45
125.85
121.56
121.32
116.44
113.62
77.48
77.16
76.84
55.07
50.50
48.77
38.87
16.24
OMe
Ph
O
N
H
Me
N
f1 (ppm)

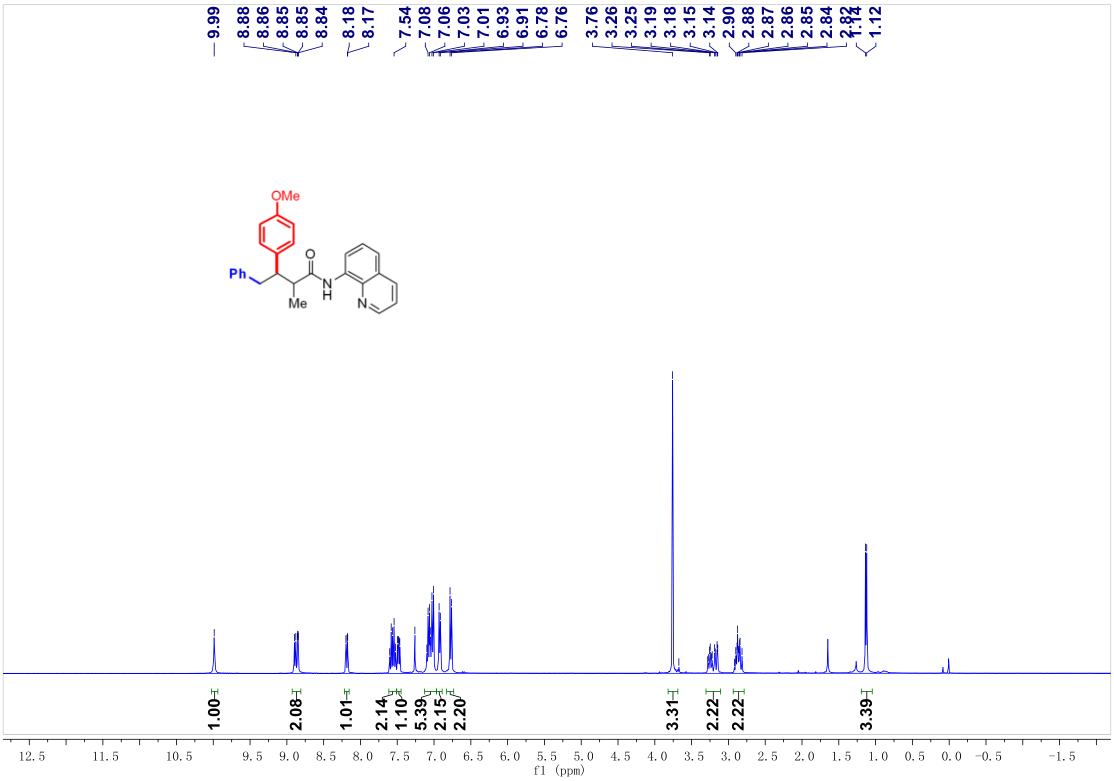
9.99
8.88
8.86
8.85
8.85
8.84
8.18
8.17
7.54
7.08
7.06
7.03
7.01
6.93
6.91
6.78
6.76
3.76
3.26
3.25
3.19
3.18
3.15
3.14
2.90
2.88
2.87
2.86
2.85
2.84
2.82
1.14
1.12
OMe
Ph
O
N
H
Me
N
1.00
2.08
1.01
2.14
1.10
5.39
2.15
2.20
3.31
2.22
2.22
3.39
f1 (ppm)

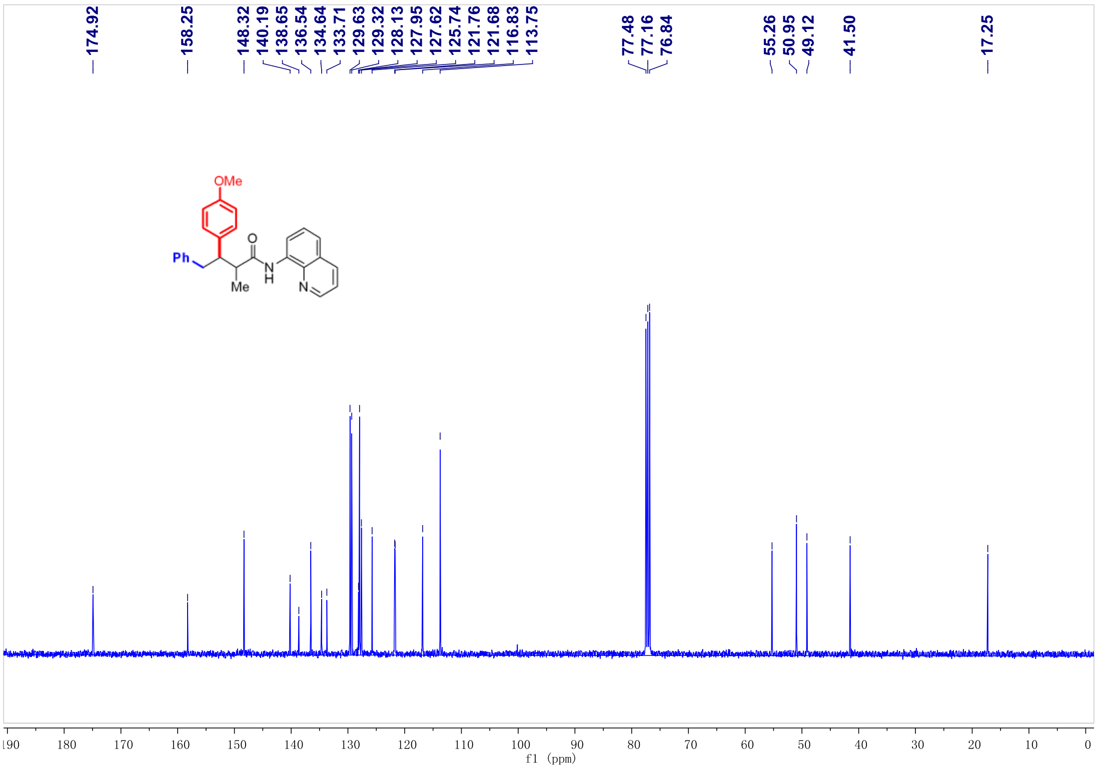
174.92
158.25
148.32
140.19
138.65
136.54
134.64
133.71
129.63
129.32
128.13
127.95
127.62
125.74
121.76
121.68
116.83
113.75
77.48
77.16
76.84
55.26
50.95
49.12
41.50
17.25
OMe
Ph
O
N
H
Me
N
f1 (ppm)

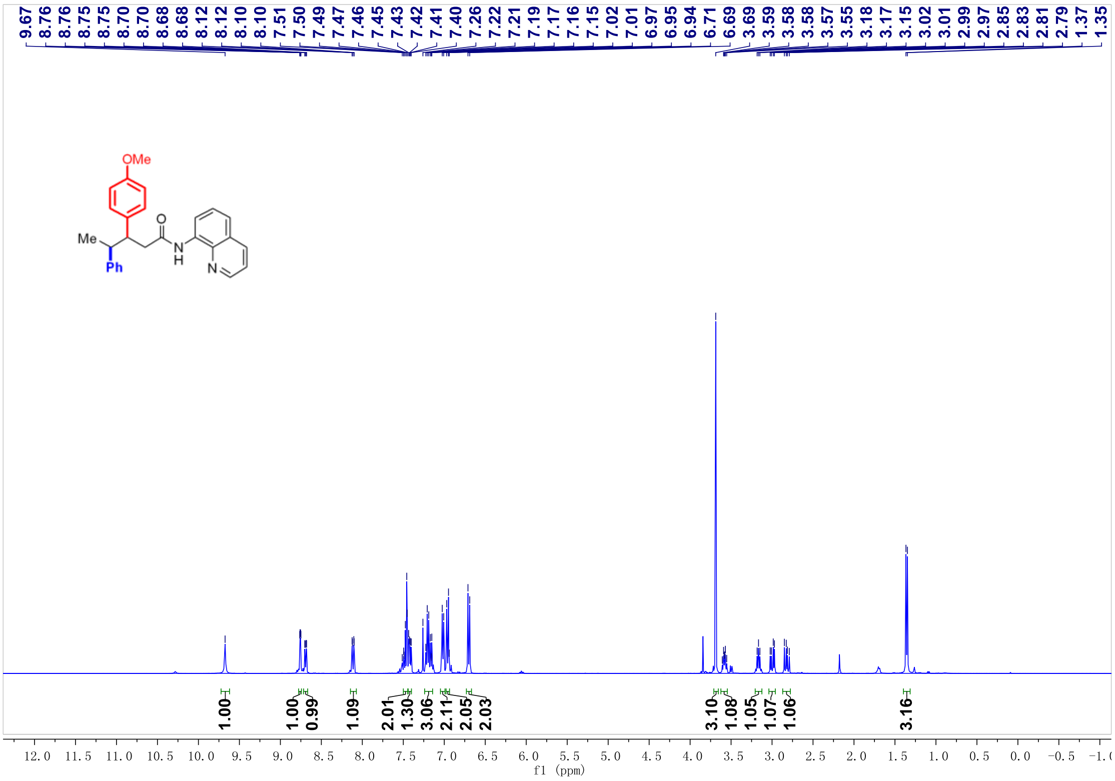
9.67
8.76
8.76
8.75
8.75
8.70
8.70
8.68
8.68
8.12
8.12
8.10
8.10
7.51
7.50
7.49
7.47
7.46
7.45
7.43
7.42
7.41
7.40
7.26
7.22
7.21
7.19
7.17
7.16
7.15
7.02
7.01
6.97
6.95
6.94
6.71
6.69
3.69
3.59
3.58
3.58
3.57
3.55
3.18
3.17
3.15
3.02
3.01
2.99
2.97
2.85
2.83
2.81
2.79
1.37
1.35
OMe
Me
Ph
O
N
H
N
1.00
1.00
0.99
1.09
2.01
1.30
3.06
2.11
2.05
2.03
3.10
1.08
1.05
1.07
1.06
3.16
f1 (ppm)

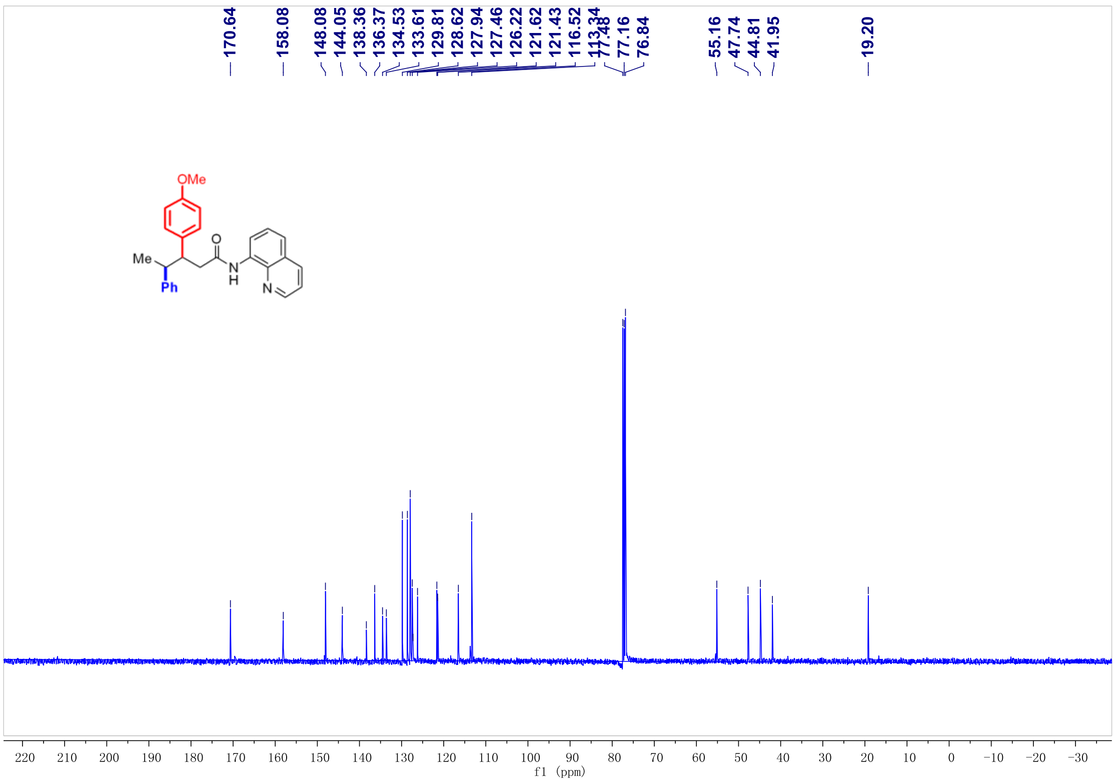
170.64
158.08
148.08
144.05
138.36
136.37
134.53
133.61
129.81
128.62
127.94
127.46
126.22
121.62
121.43
116.52
113.34
77.48
77.16
76.84
55.16
47.74
44.81
41.95
19.20
OMe
Me
Ph
O
N
H
N
f1 (ppm)

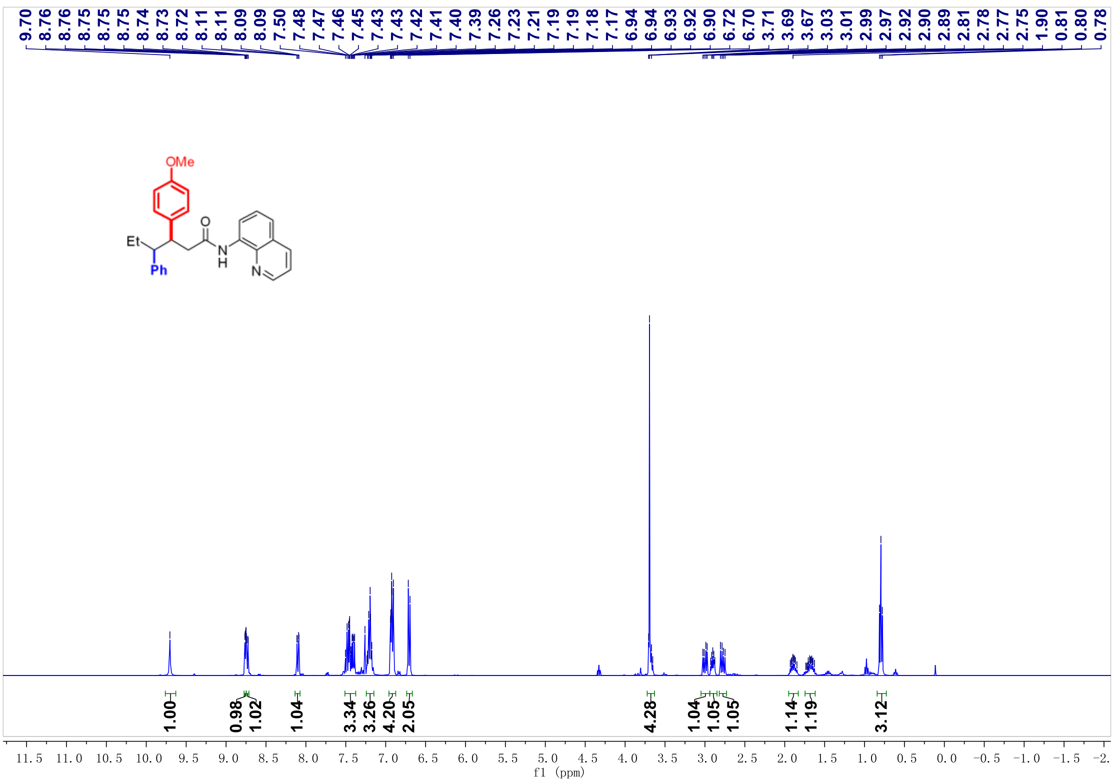
9.70
8.76
8.76
8.75
8.75
8.74
8.73
8.72
8.11
8.11
8.09
8.09
7.50
7.48
7.47
7.46
7.45
7.43
7.43
7.42
7.41
7.40
7.39
7.26
7.23
7.21
7.19
7.19
7.18
7.17
6.94
6.94
6.93
6.92
6.90
6.72
6.70
3.71
3.69
3.67
3.03
3.01
2.99
2.97
2.92
2.90
2.89
2.81
2.78
2.77
2.75
1.90
0.81
0.80
0.78
OMe
Et
Ph
O
N
H
N
1.00
0.98
1.02
1.04
3.34
3.26
4.20
2.05
4.28
1.04
1.05
1.05
1.14
1.19
3.12
f1 (ppm)

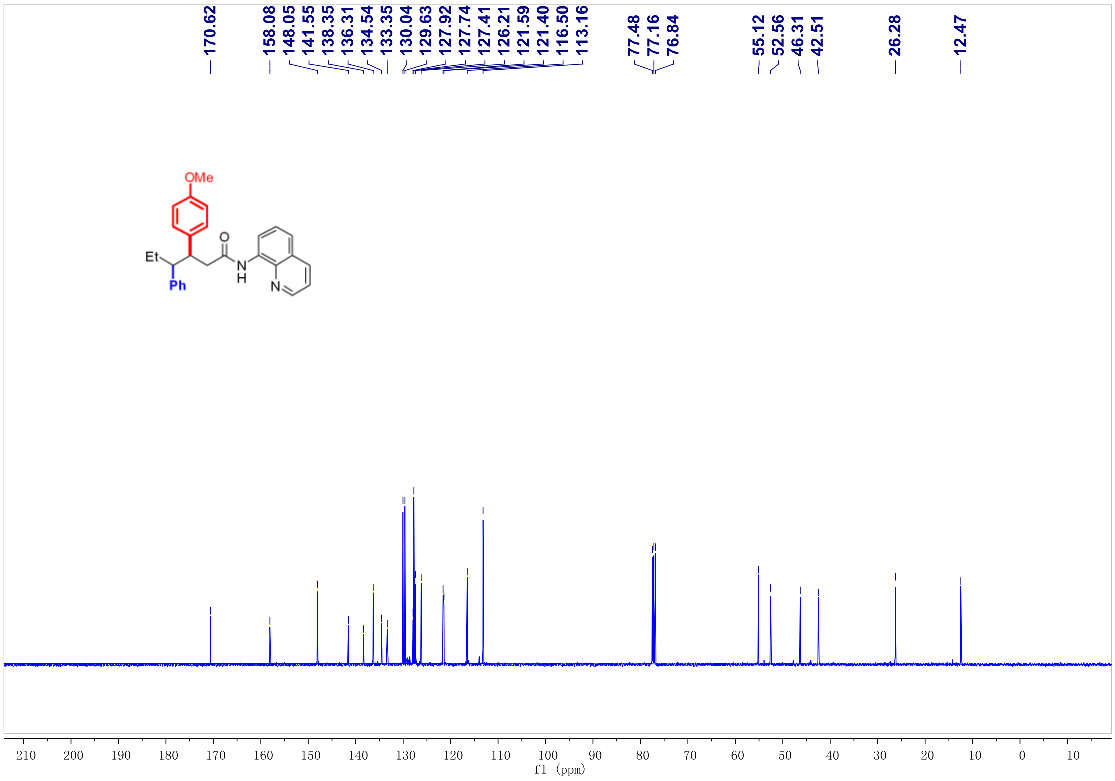
170.62
158.08
148.05
141.55
138.35
136.31
134.54
133.35
130.04
129.63
127.92
127.74
127.41
126.21
121.59
121.40
116.50
113.16
77.48
77.16
76.84
55.12
52.56
46.31
42.51
26.28
12.47
OMe
Et
Ph
O
N
H
N
f1 (ppm)

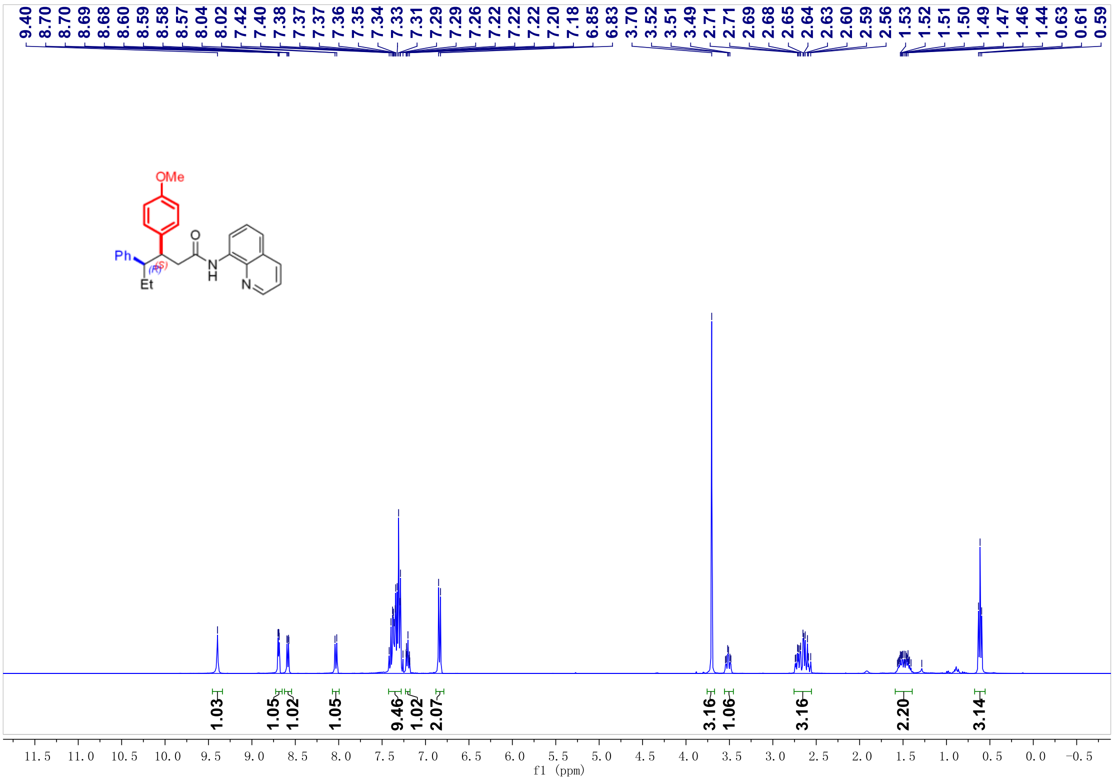
9.40
8.70
8.70
8.69
8.68
8.60
8.59
8.58
8.57
8.04
8.02
7.42
7.40
7.38
7.37
7.37
7.36
7.35
7.34
7.33
7.31
7.29
7.29
7.26
7.22
7.22
7.22
7.20
7.18
6.85
6.83
3.70
3.52
3.51
3.49
2.71
2.71
2.69
2.68
2.65
2.64
2.63
2.60
2.59
2.56
1.53
1.52
1.51
1.50
1.49
1.47
1.46
1.44
0.63
0.61
0.59
OMe
Ph
(R)
(S)
Et
O
N
H
N
1.03
1.05
1.02
1.05
9.46
1.02
2.07
3.16
1.06
3.16
2.20
3.14
f1 (ppm)

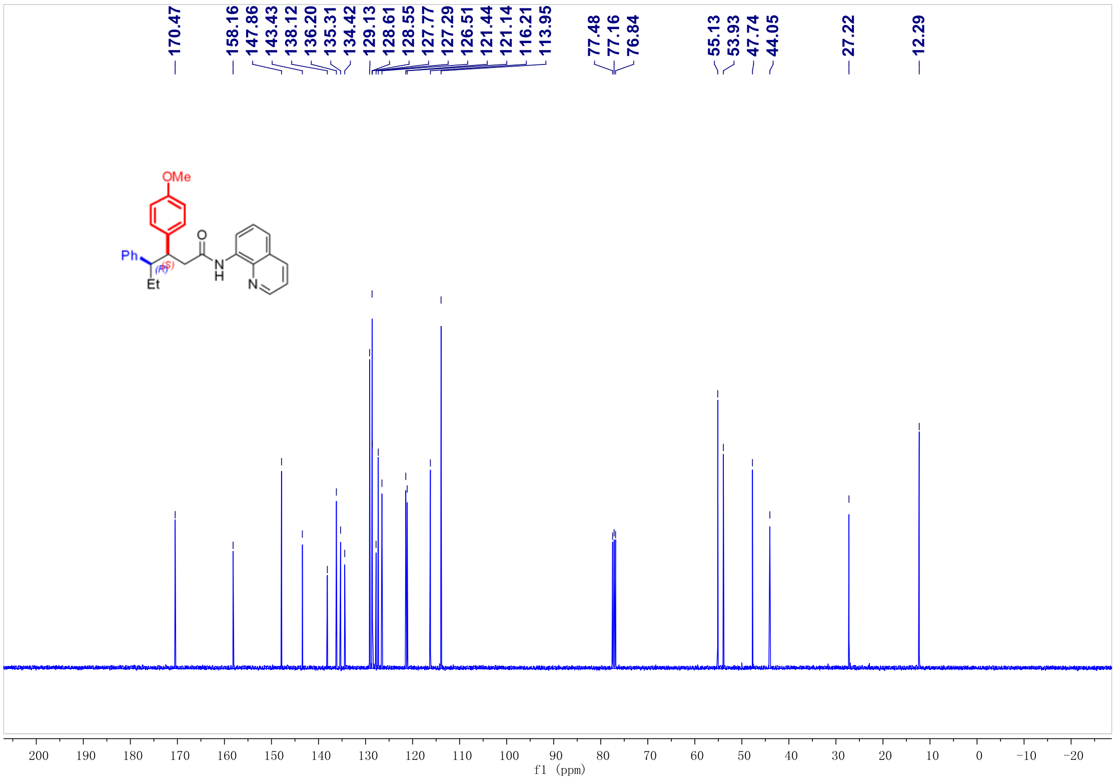
170.47
158.16
147.86
143.43
138.12
136.20
135.31
134.42
129.13
128.61
128.55
127.77
127.29
126.51
121.44
121.14
116.21
113.95
77.48
77.16
76.84
55.13
53.93
47.74
44.05
27.22
12.29
OMe
Ph
(S)
(R)
Et
O
N
H
N
f1 (ppm)

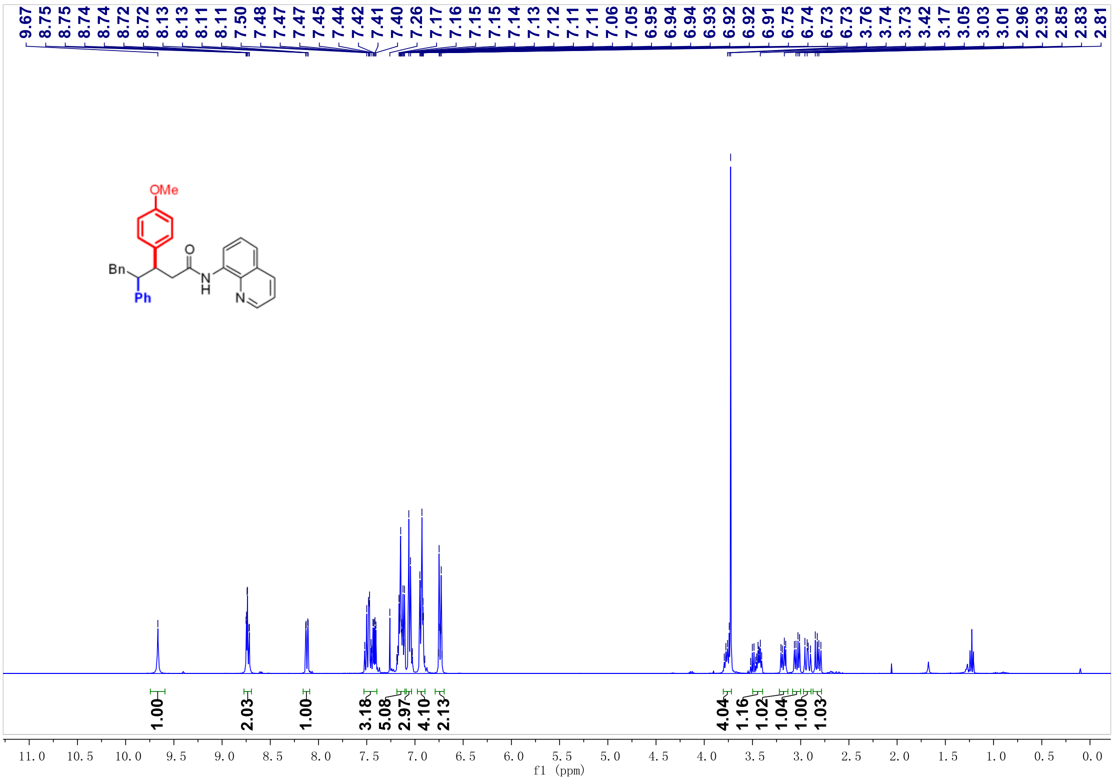
9.67
8.75
8.75
8.74
8.74
8.72
8.72
8.13
8.13
8.11
8.11
7.50
7.48
7.47
7.47
7.45
7.44
7.42
7.41
7.40
7.26
7.17
7.16
7.15
7.15
7.14
7.13
7.12
7.11
7.11
7.06
7.05
6.95
6.94
6.94
6.93
6.92
6.92
6.91
6.75
6.74
6.73
6.73
3.76
3.74
3.73
3.42
3.17
3.05
3.03
3.01
2.96
2.93
2.85
2.83
2.81
OMe
Bn
Ph
O
N
H
N
1.00
2.03
1.00
3.18
5.08
2.97
4.10
2.13
4.04
1.16
1.02
1.04
1.00
1.03
f1 (ppm)

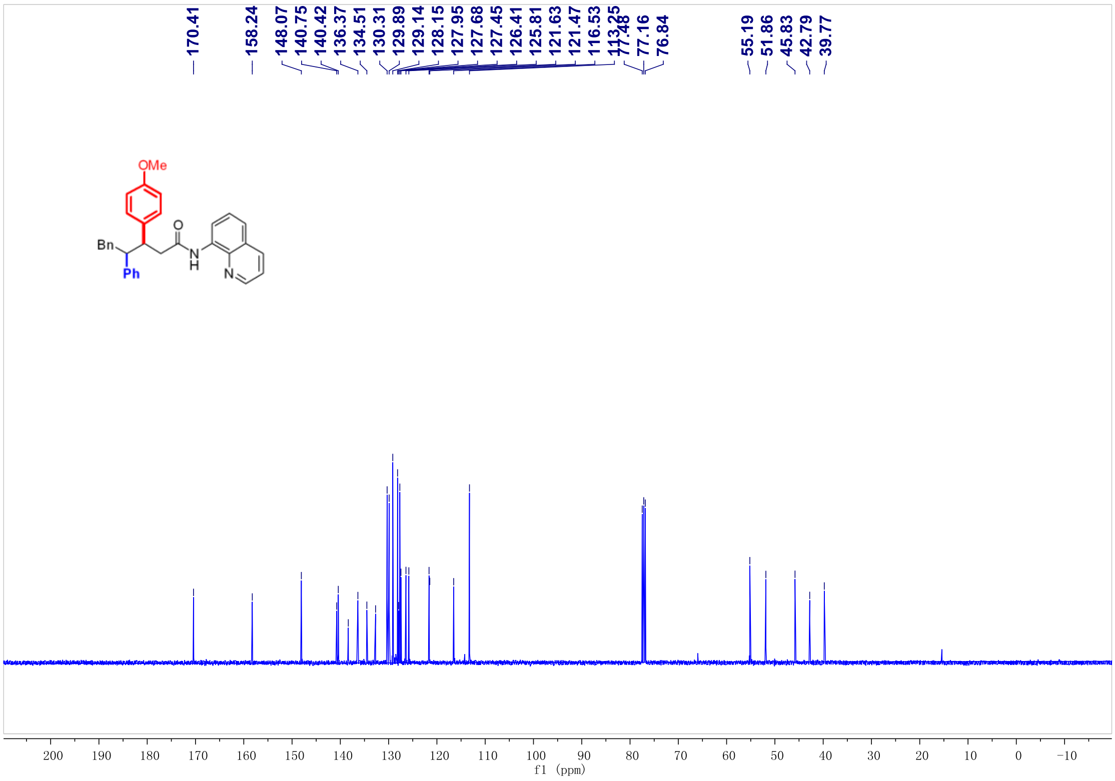
170.41
158.24
148.07
140.75
140.42
136.37
134.51
130.31
129.89
129.14
128.15
127.95
127.68
127.45
126.41
125.81
121.63
121.47
116.53
113.25
77.48
77.16
76.84
55.19
51.86
45.83
42.79
39.77
OMe
Bn
Ph
O
N
H
N
f1 (ppm)

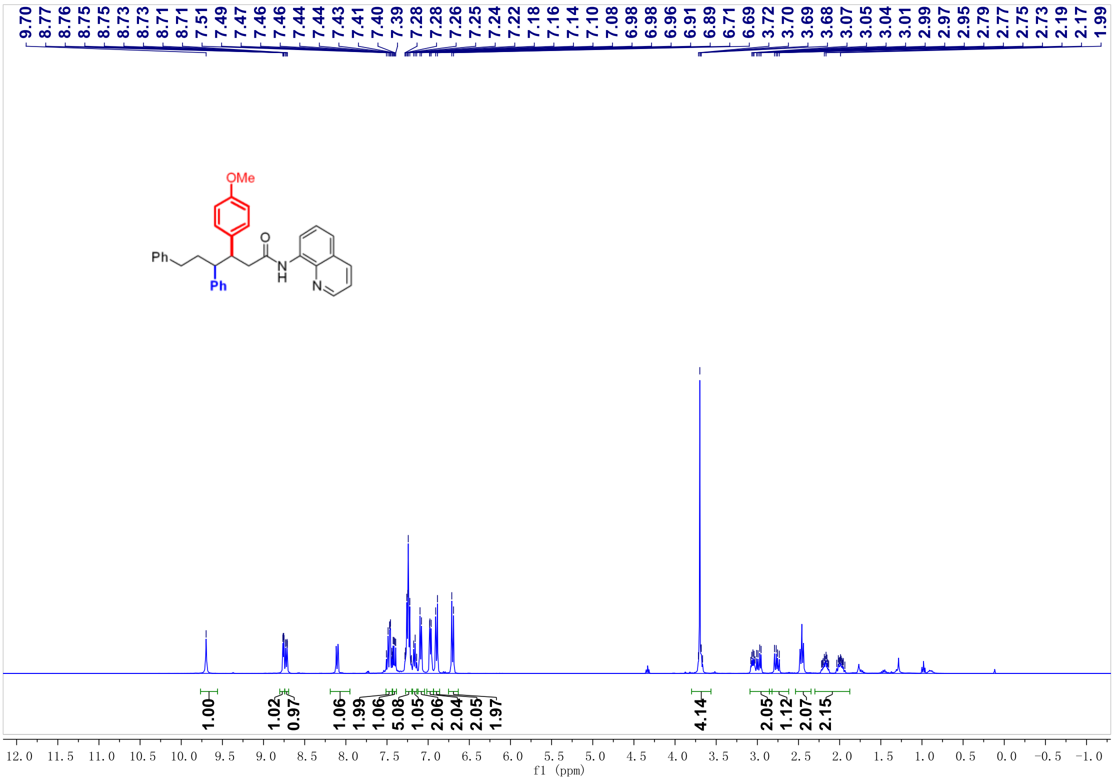
9.70
8.77
8.76
8.75
8.73
8.73
8.71
8.71
7.51
7.49
7.47
7.46
7.46
7.44
7.44
7.43
7.41
7.40
7.39
7.28
7.28
7.26
7.25
7.24
7.22
7.18
7.16
7.14
7.10
7.08
6.98
6.96
6.91
6.89
6.71
6.69
3.72
3.70
3.69
3.68
3.07
3.05
3.04
3.01
2.99
2.97
2.95
2.79
2.77
2.75
2.73
2.19
2.17
1.99
OMe
Ph
Ph
O
N
H
N
1.00
1.02
0.97
1.06
1.99
1.06
5.08
1.05
2.06
2.04
2.05
1.97
4.14
2.05
1.12
2.07
2.15
f1 (ppm)

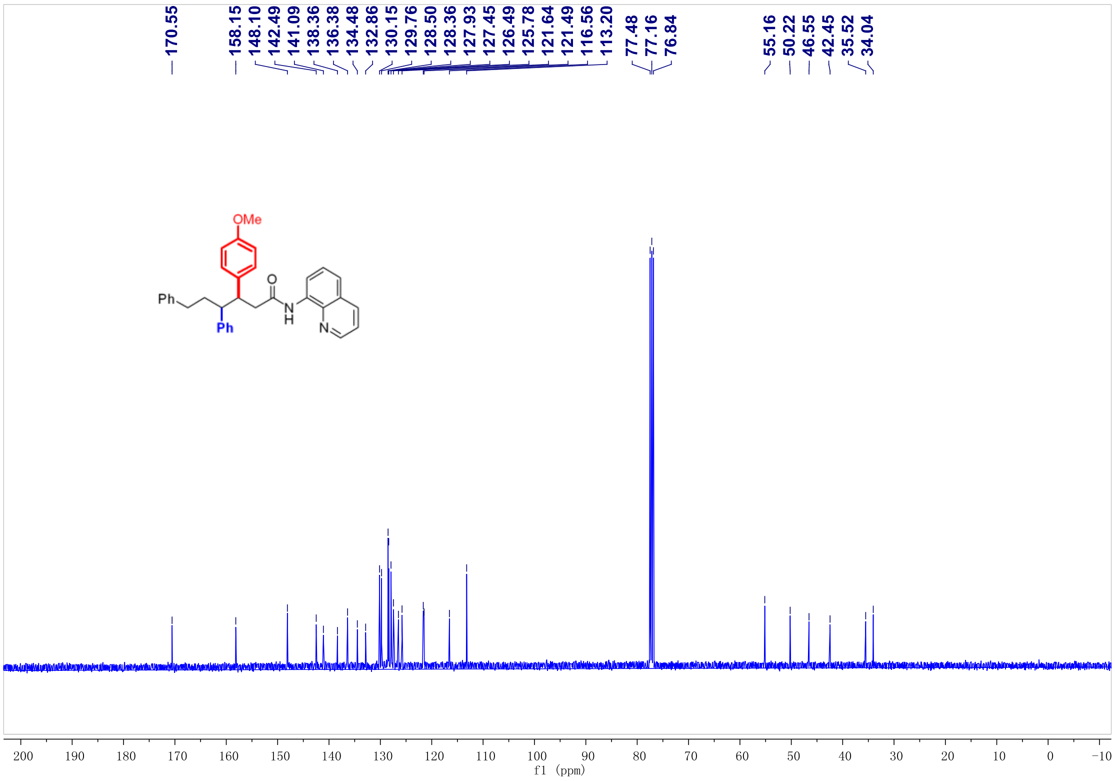
170.55
158.15
148.10
142.49
141.09
138.36
136.38
134.48
132.86
130.15
129.76
128.50
128.36
127.93
127.45
126.49
125.78
121.64
121.49
116.56
113.20
77.48
77.16
76.84
55.16
50.22
46.55
42.45
35.52
34.04
OMe
Ph
Ph
O
N
H
N
f1 (ppm)

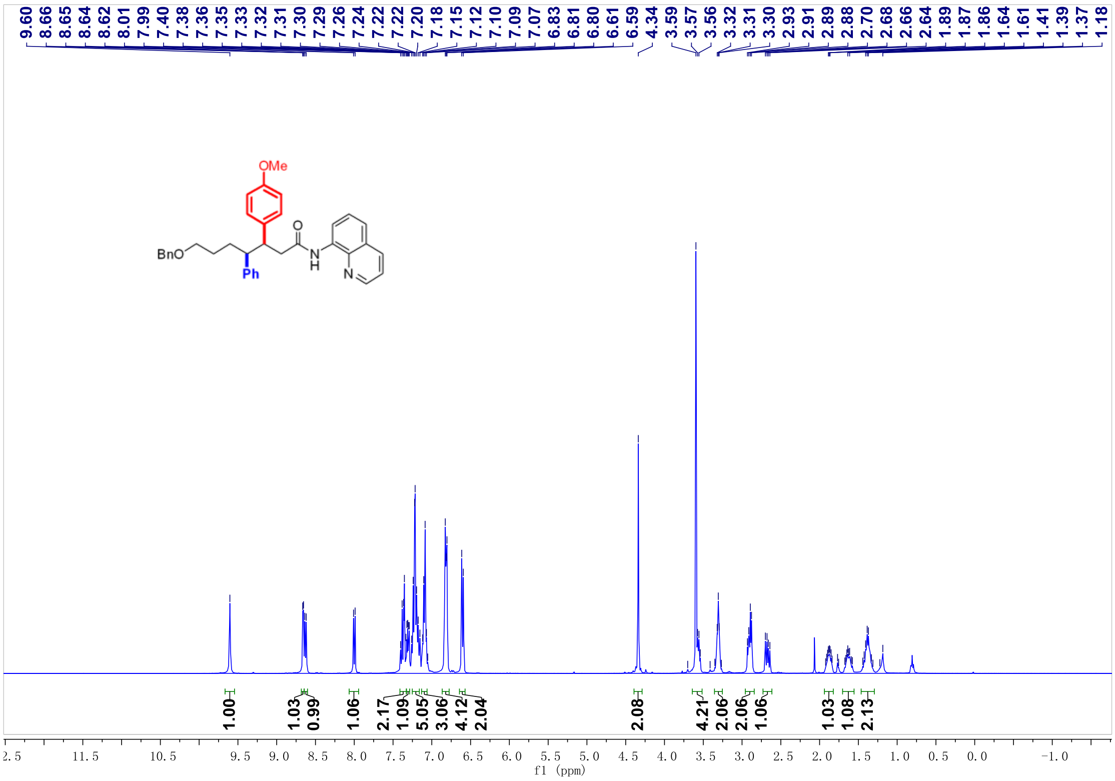
9.60
8.66
8.65
8.64
8.62
8.01
7.99
7.40
7.38
7.36
7.35
7.33
7.32
7.31
7.30
7.29
7.26
7.24
7.22
7.22
7.20
7.18
7.15
7.12
7.10
7.09
7.07
6.83
6.81
6.80
6.61
6.59
4.34
3.59
3.57
3.56
3.32
3.31
3.30
2.93
2.91
2.89
2.88
2.70
2.68
2.66
2.64
1.89
1.87
1.86
1.64
1.61
1.41
1.39
1.37
1.18
OMe
BnO
Ph
O
N
H
N
1.00
1.03
0.99
1.06
2.17
1.09
5.05
3.06
4.12
2.04
2.08
4.21
2.06
2.06
1.06
1.03
1.08
2.13
f1 (ppm)

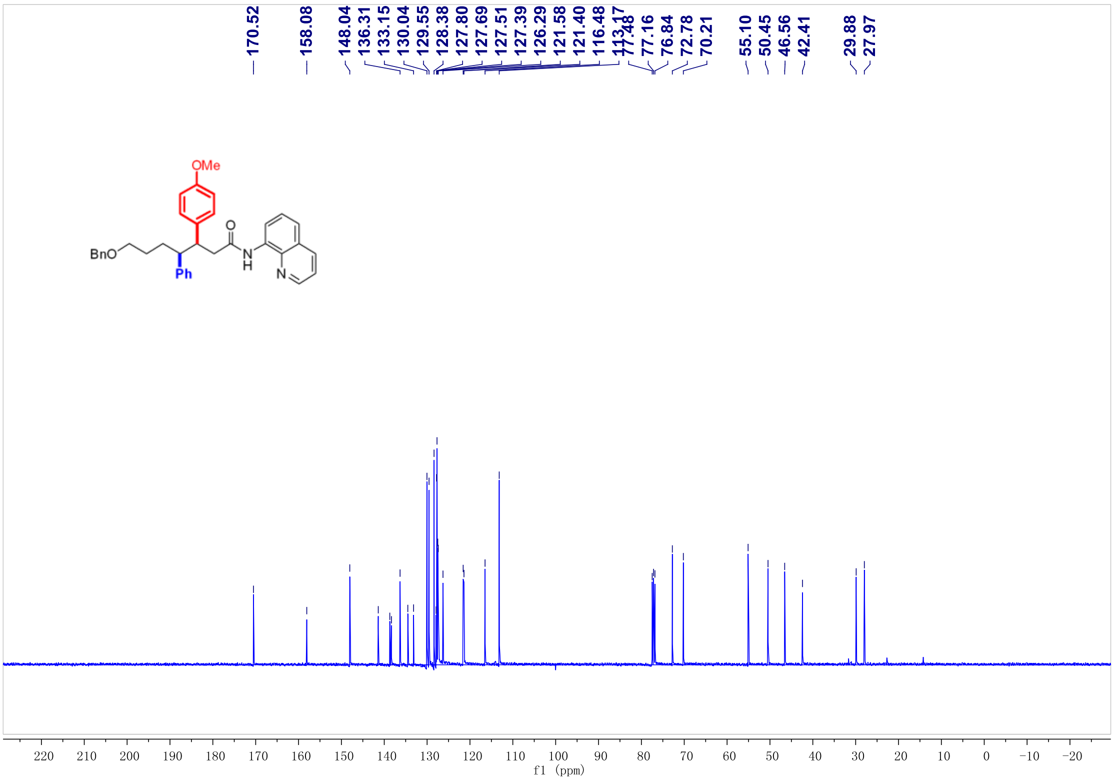
170.52
158.08
148.04
136.31
133.15
130.04
129.55
128.38
127.80
127.69
127.51
127.39
126.29
121.58
121.40
116.48
113.17
77.48
77.16
76.84
72.78
70.21
55.10
50.45
46.56
42.41
29.88
27.97
OMe
BnO
Ph
O
N
H
N
f1 (ppm)

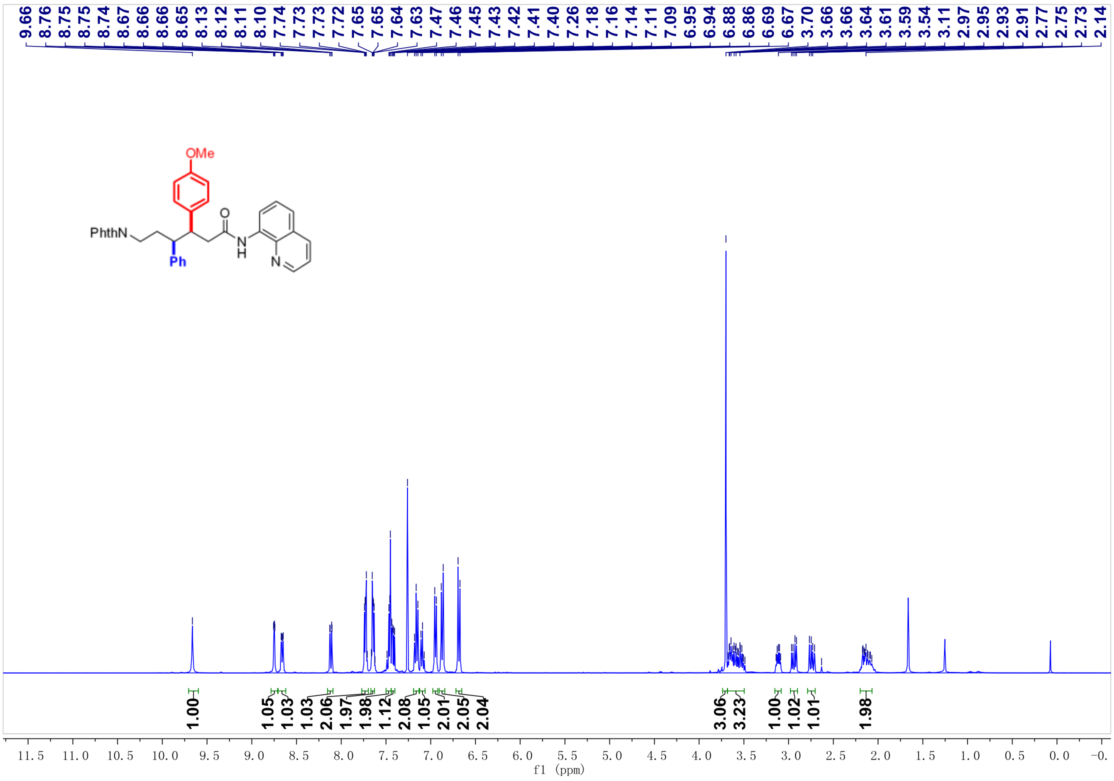
9.66
8.76
8.75
8.75
8.74
8.67
8.66
8.66
8.65
8.13
8.12
8.11
8.10
7.74
7.73
7.73
7.72
7.65
7.65
7.64
7.63
7.47
7.46
7.45
7.43
7.42
7.41
7.40
7.26
7.18
7.16
7.14
7.11
7.09
6.95
6.94
6.88
6.86
6.69
6.67
3.70
3.66
3.66
3.64
3.61
3.59
3.54
3.11
2.97
2.95
2.93
2.91
2.77
2.75
2.73
2.14
OMe
PhthN
O
N
H
N
Ph
1.00
1.05
1.03
1.03
2.06
1.97
1.98
1.12
2.08
1.05
2.01
2.05
2.04
3.06
3.23
1.00
1.02
1.01
1.98
f1 (ppm)

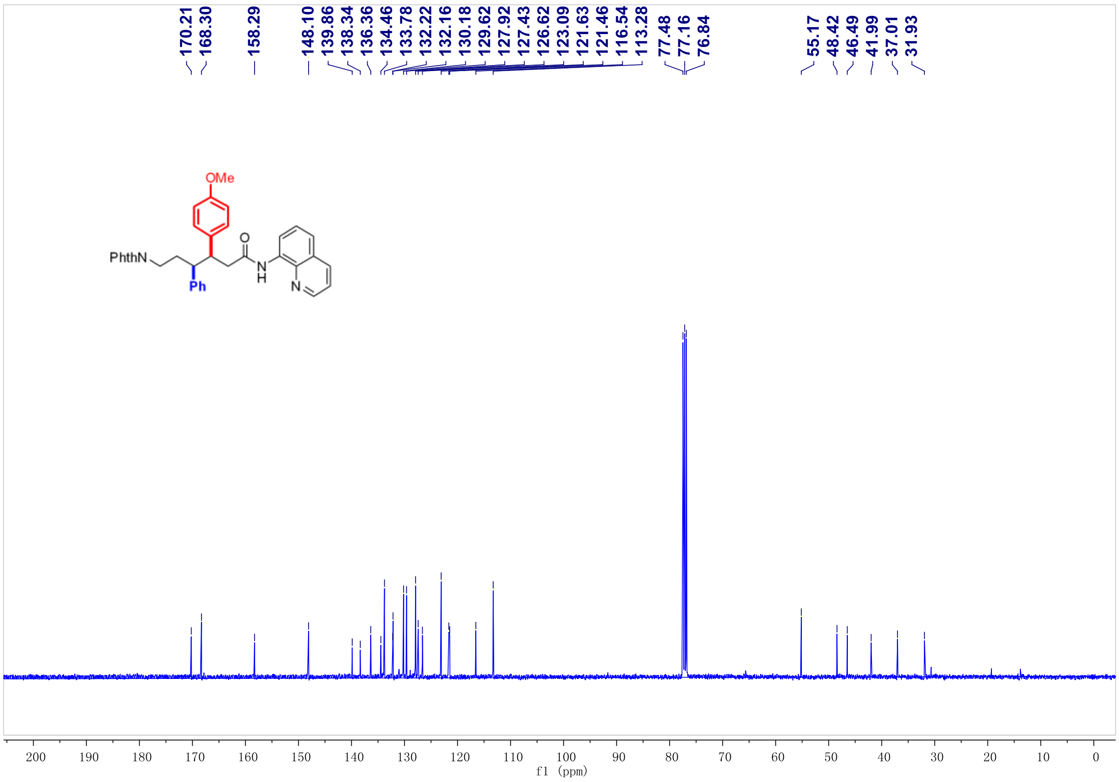
170.21
168.30
158.29
148.10
139.86
138.34
136.36
134.46
133.78
132.22
132.16
130.18
129.62
127.92
127.43
126.62
123.09
121.63
121.46
116.54
113.28
77.48
77.16
76.84
55.17
48.42
46.49
41.99
37.01
31.93
OMe
PhthN
O
N
H
N
Ph
f1 (ppm)

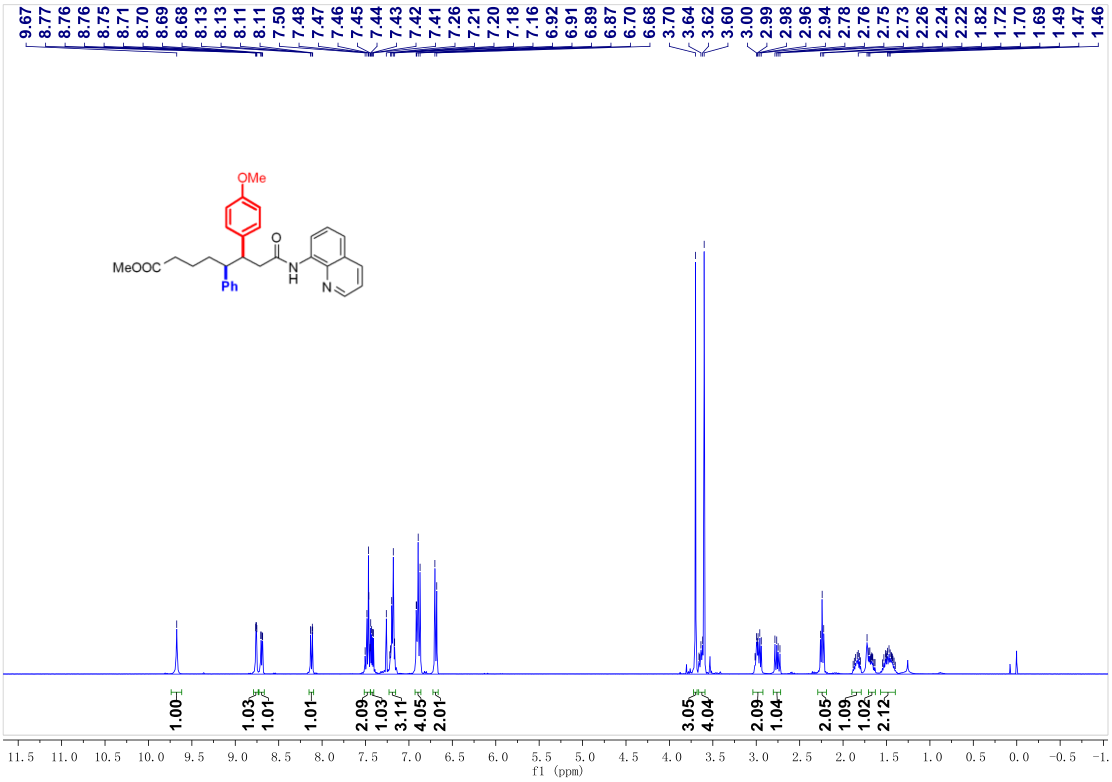

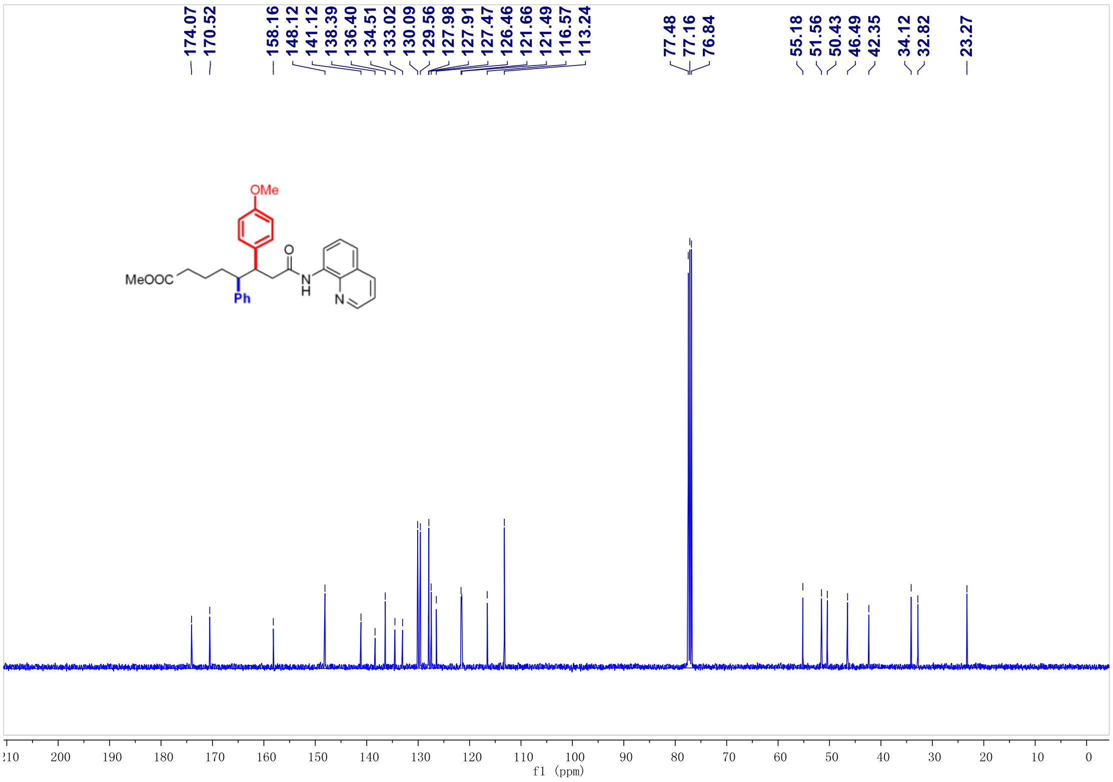

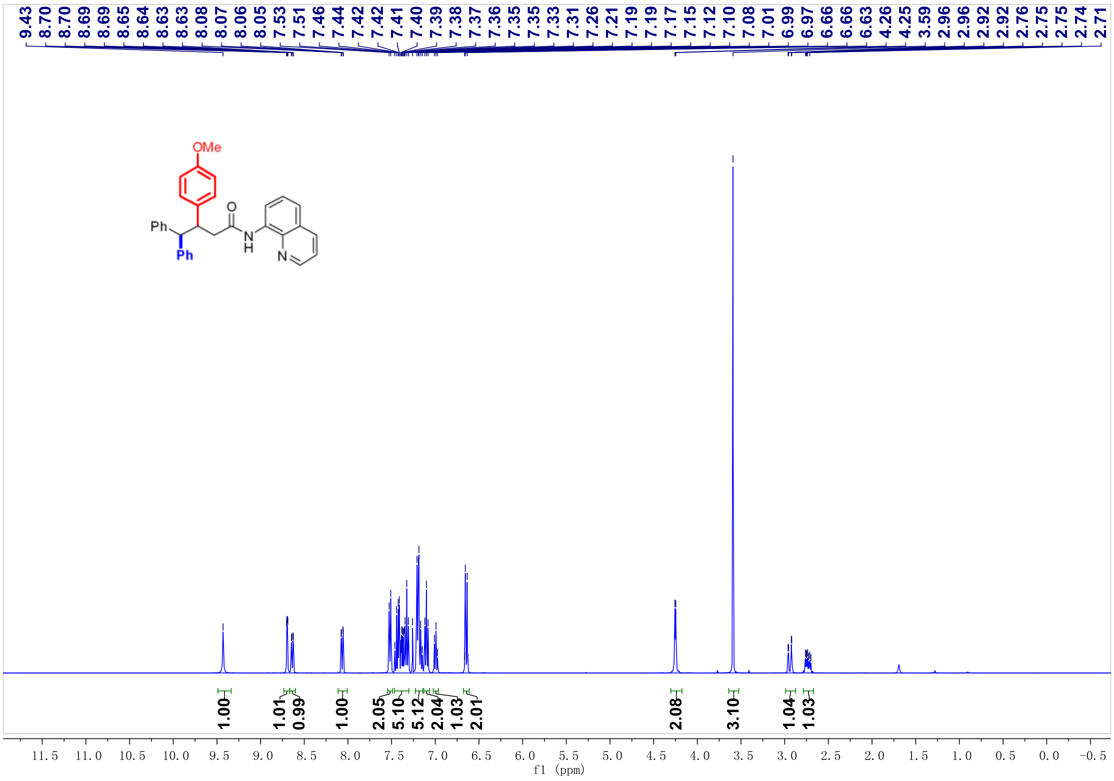
9.43
8.70
8.70
8.69
8.65
8.64
8.63
8.63
8.08
8.07
8.06
8.05
7.53
7.51
7.46
7.44
7.42
7.42
7.41
7.40
7.39
7.38
7.37
7.36
7.35
7.35
7.33
7.31
7.26
7.21
7.19
7.17
7.15
7.12
7.10
7.08
7.01
6.99
6.97
6.66
6.63
4.26
4.25
3.59
2.96
2.96
2.92
2.92
2.76
2.75
2.75
2.74
2.71
OMe
Ph
Ph
O
N
H
N
1.00
1.01
0.99
1.00
2.05
5.10
5.12
2.04
1.03
2.01
2.08
3.10
1.04
1.03
f1 (ppm)

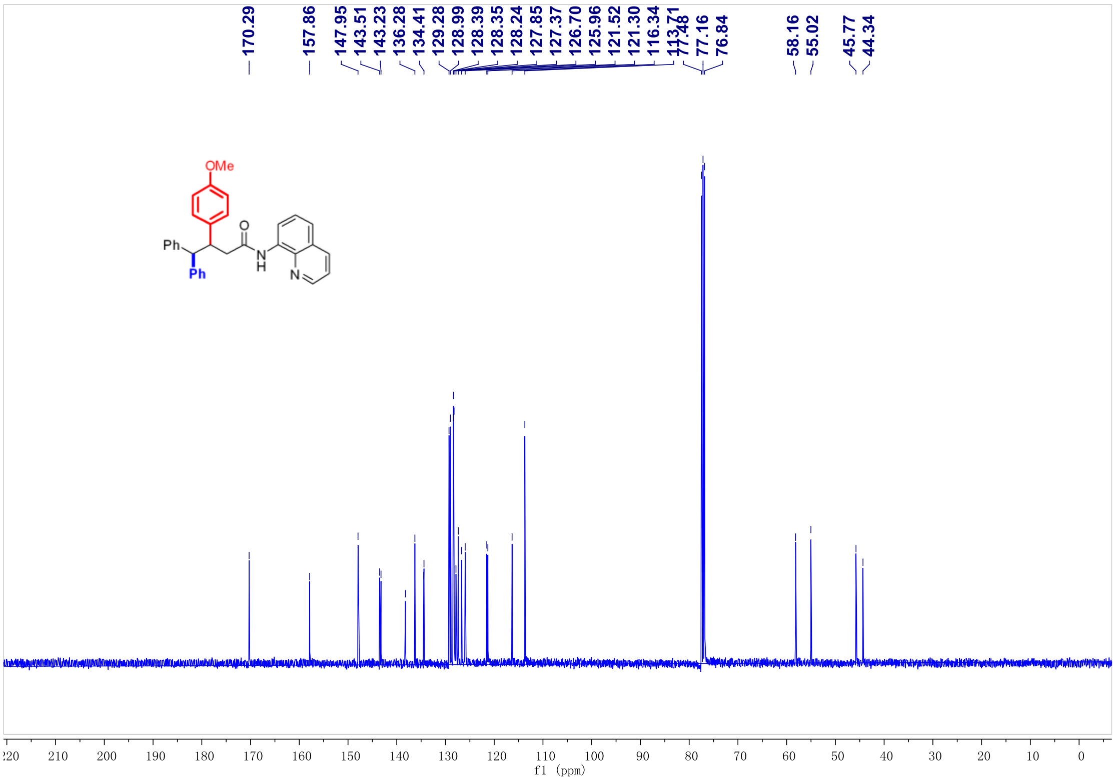
170.29
157.86
147.95
143.51
143.23
136.28
134.41
129.28
128.99
128.39
128.35
128.24
127.85
127.37
126.70
125.96
121.52
121.30
116.34
113.71
77.48
77.16
76.84
58.16
55.02
45.77
44.34
OMe
Ph
Ph
O
N
H
N
f1 (ppm)

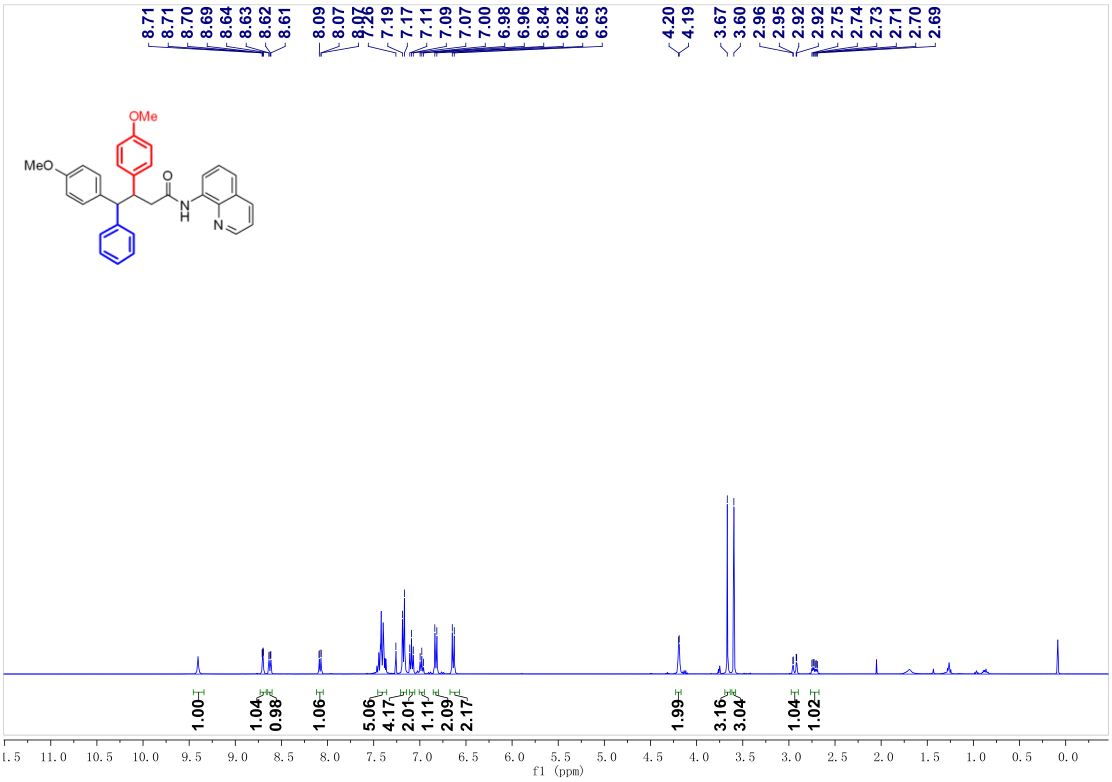

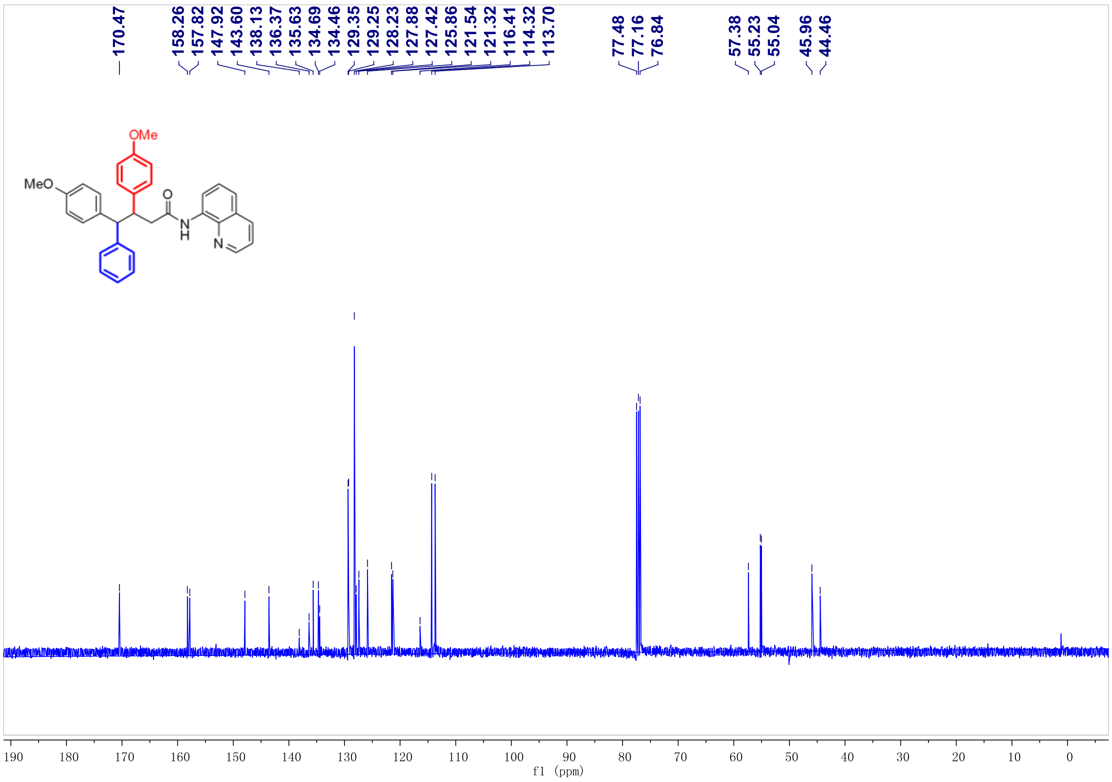

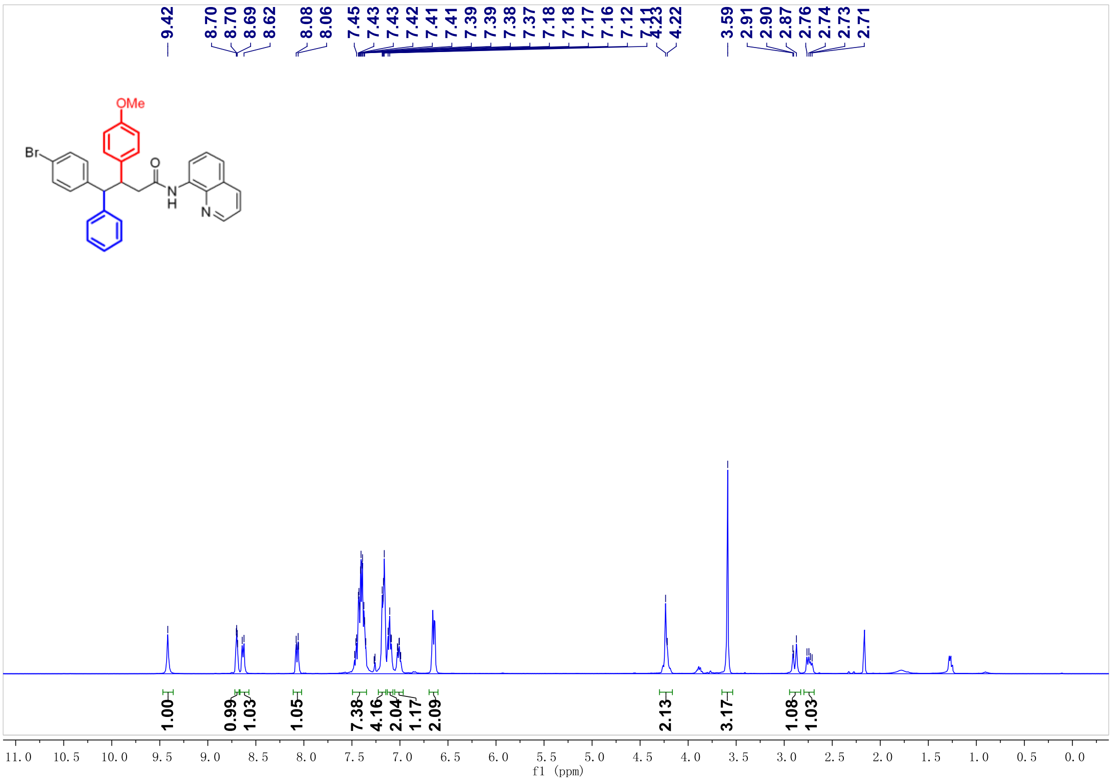
9.42
8.70
8.70
8.69
8.62
8.08
8.06
7.45
7.43
7.43
7.42
7.41
7.41
7.39
7.39
7.38
7.37
7.18
7.17
7.16
7.12
7.11
4.23
4.22
3.59
2.91
2.90
2.87
2.76
2.74
2.73
2.71
OMe
Br
O
N
H
N
1.00
0.99
1.03
1.05
7.38
4.16
2.04
1.17
2.09
2.13
3.17
1.08
1.03
11.0 10.5 10.0 9.5 9.0 8.5 8.0 7.5 7.0 6.5 6.0 5.5 5.0 4.5 4.0 3.5 3.0 2.5 2.0 1.5 1.0 0.5 0.0
f1 (ppm)

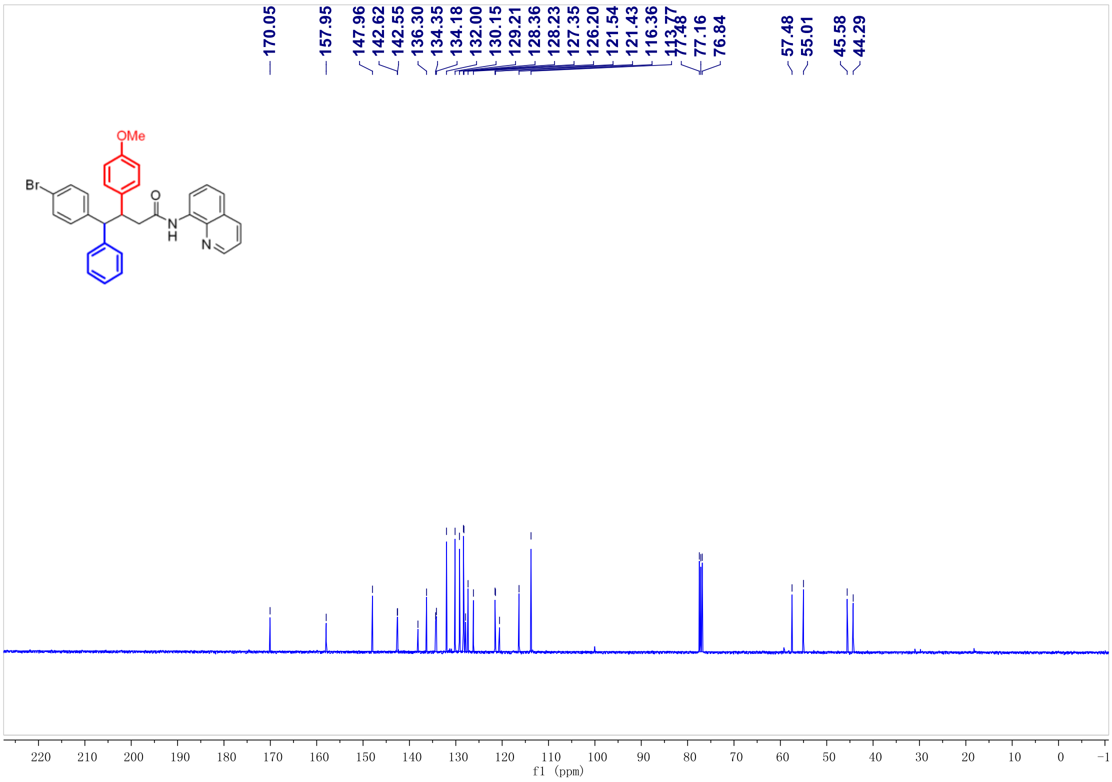
170.05
157.95
147.96
142.62
142.55
136.30
134.35
134.18
132.00
130.15
129.21
128.36
128.23
127.35
126.20
121.54
121.43
116.36
113.77
77.48
77.16
76.84
57.48
55.01
45.58
44.29
OMe
Br
O
N
H
N
220 210 200 190 180 170 160 150 140 130 120 110 100 90 80 70 60 50 40 30 20 10 0 -1
f1 (ppm)

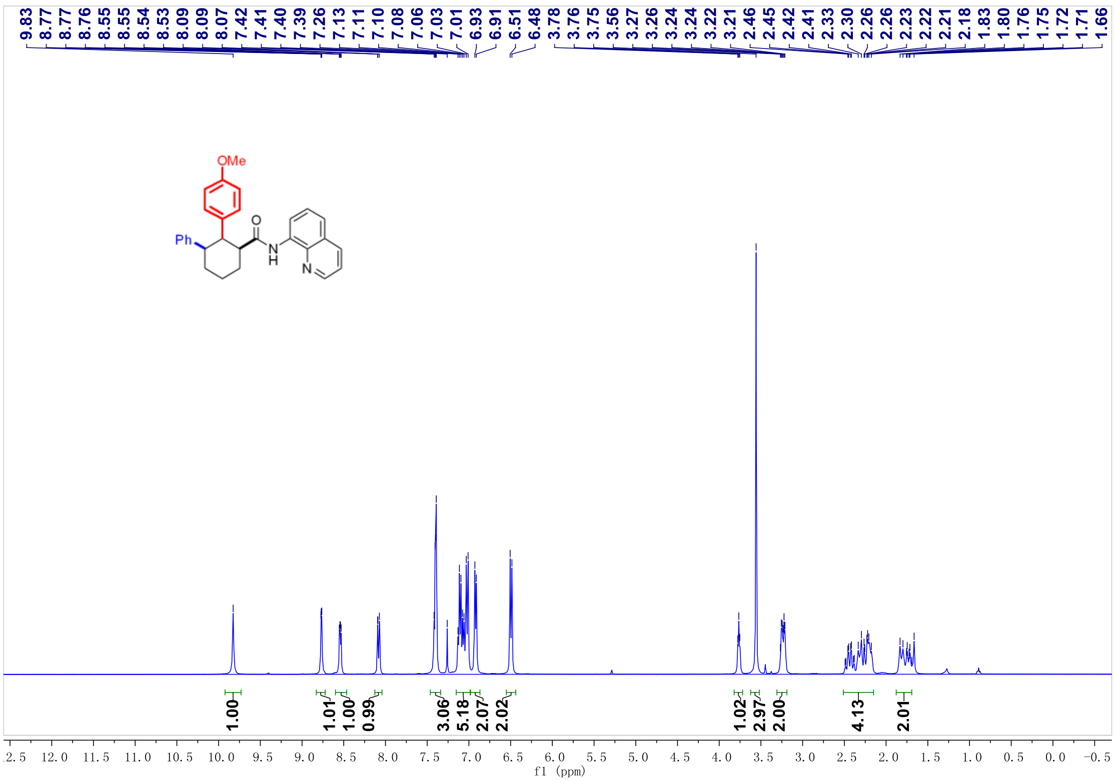
9.83
8.77
8.76
8.55
8.54
8.53
8.09
8.07
7.42
7.41
7.40
7.39
7.26
7.13
7.11
7.10
7.08
7.06
7.03
7.01
6.93
6.91
6.51
6.48
3.78
3.76
3.75
3.56
3.27
3.26
3.24
3.22
3.21
2.46
2.45
2.42
2.41
2.33
2.30
2.26
2.23
2.22
2.21
2.18
1.83
1.80
1.76
1.75
1.72
1.71
1.66
OMe
Ph
O
N
H
N
1.00
1.01
1.00
0.99
3.06
5.18
2.07
2.02
1.02
2.97
2.00
4.13
2.01
f1 (ppm)

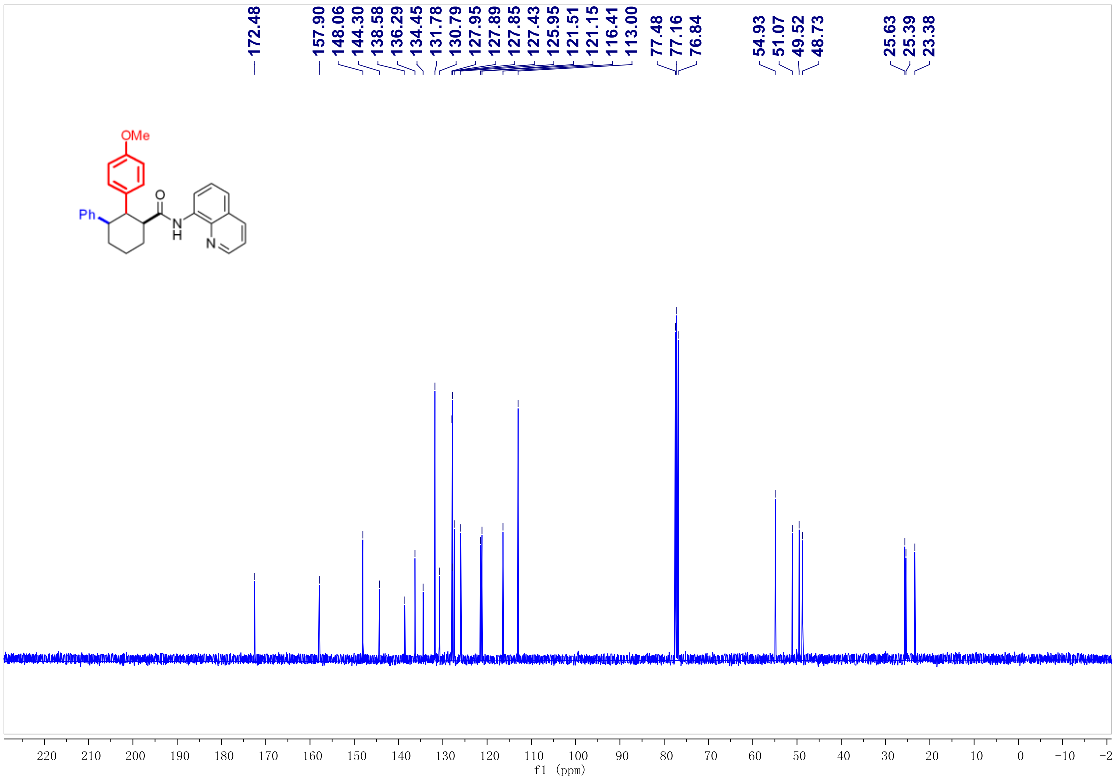
172.48
157.90
148.06
144.30
138.58
136.29
134.45
131.78
130.79
127.95
127.89
127.85
127.43
125.95
121.51
121.15
116.41
113.00
77.48
77.16
76.84
54.93
51.07
49.52
48.73
25.63
25.39
23.38
OMe
Ph
O
N
H
N
f1 (ppm)

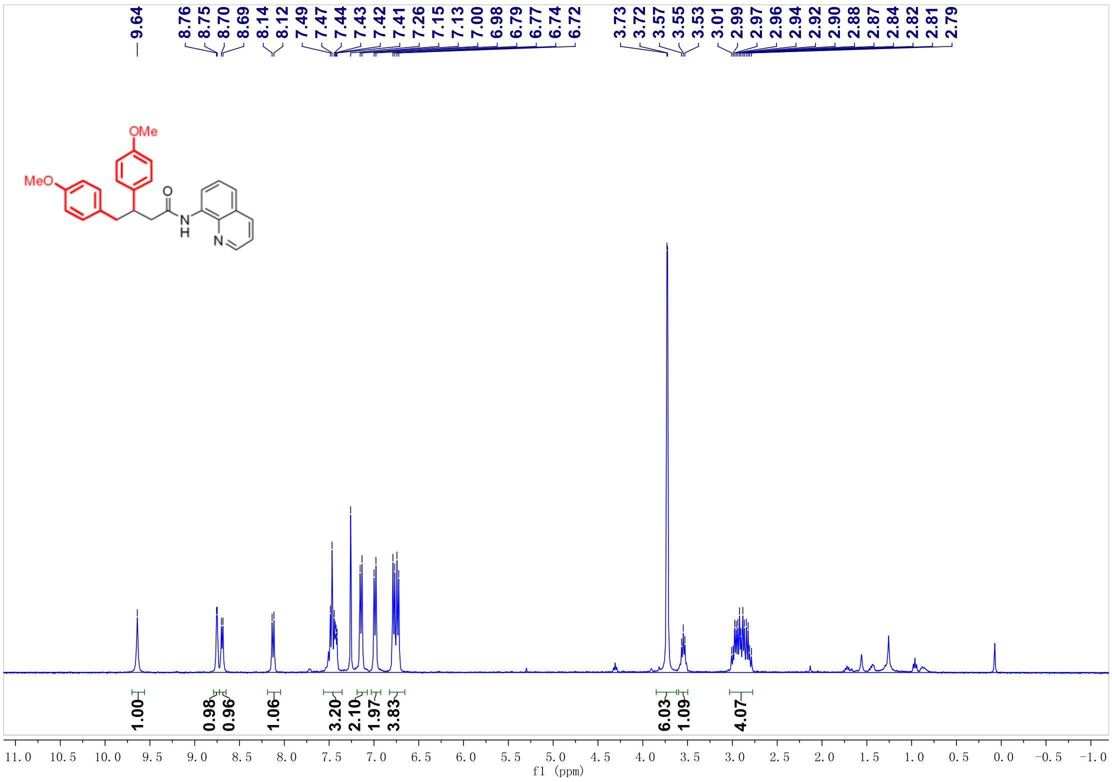
9.64
8.76
8.75
8.70
8.69
8.14
8.12
7.49
7.47
7.44
7.43
7.42
7.41
7.26
7.15
7.13
7.00
6.98
6.79
6.77
6.74
6.72
3.73
3.72
3.57
3.55
3.53
3.01
2.99
2.97
2.96
2.94
2.92
2.90
2.88
2.87
2.84
2.82
2.81
2.79
OMe
MeO
O
N
H
N
1.00
0.98
0.96
1.06
3.20
2.10
1.97
3.83
6.03
1.09
4.07
f1 (ppm)

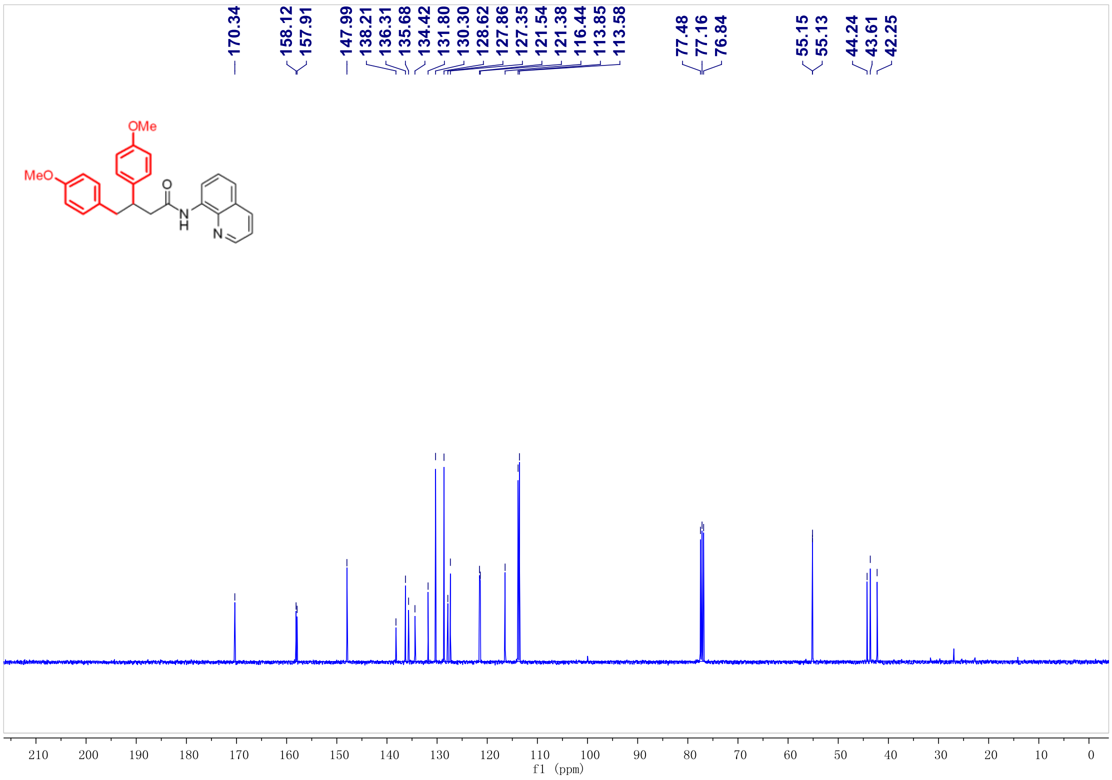
170.34
158.12
157.91
147.99
138.21
136.31
135.68
134.42
131.80
130.30
128.62
127.86
127.35
121.54
121.38
116.44
113.85
113.58
77.48
77.16
76.84
55.15
55.13
44.24
43.61
42.25
OMe
MeO
O
N
H
N
f1 (ppm)

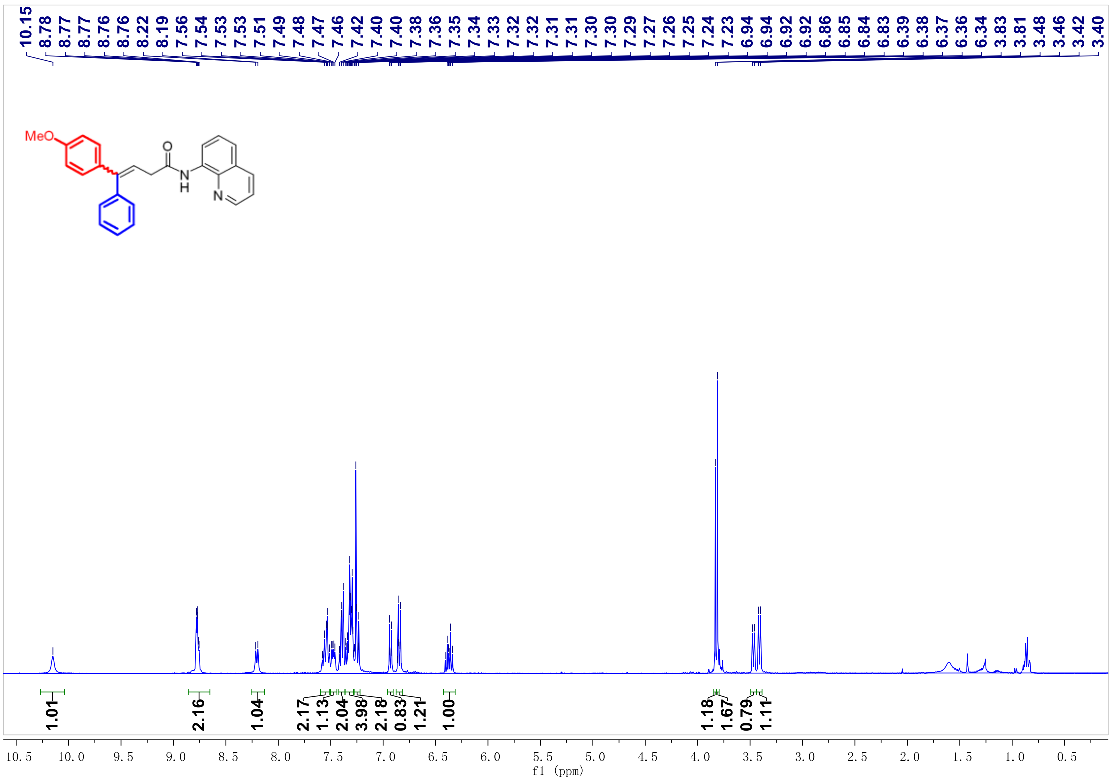

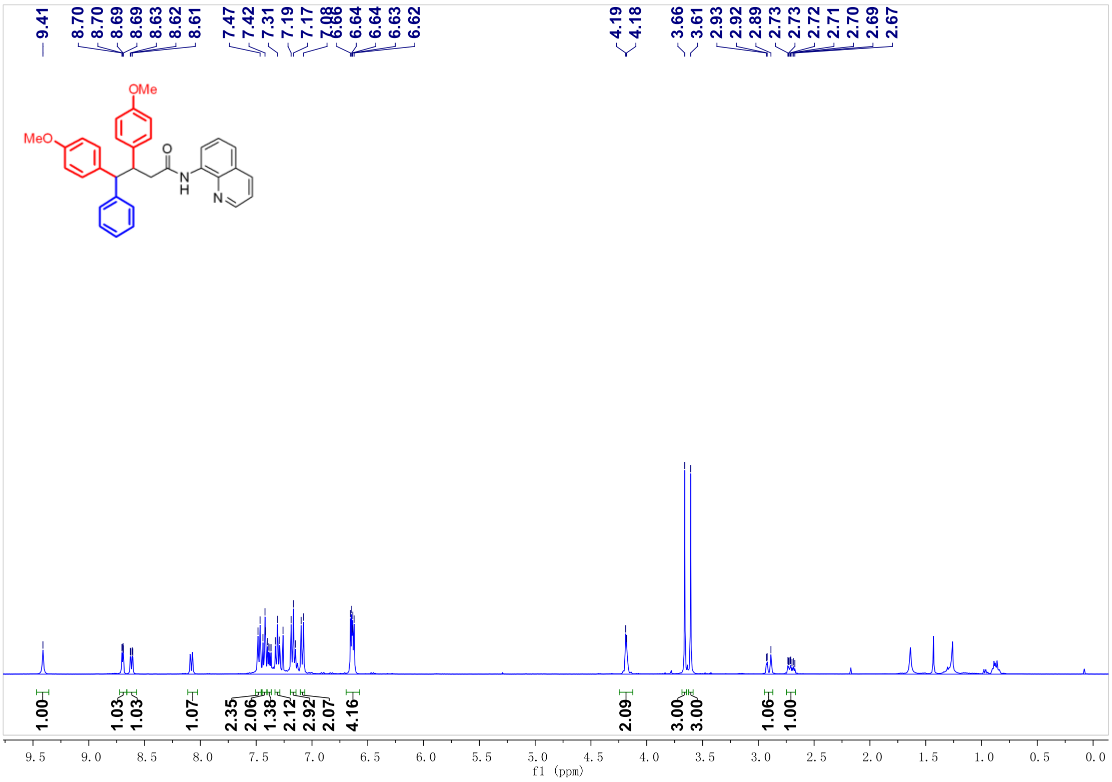

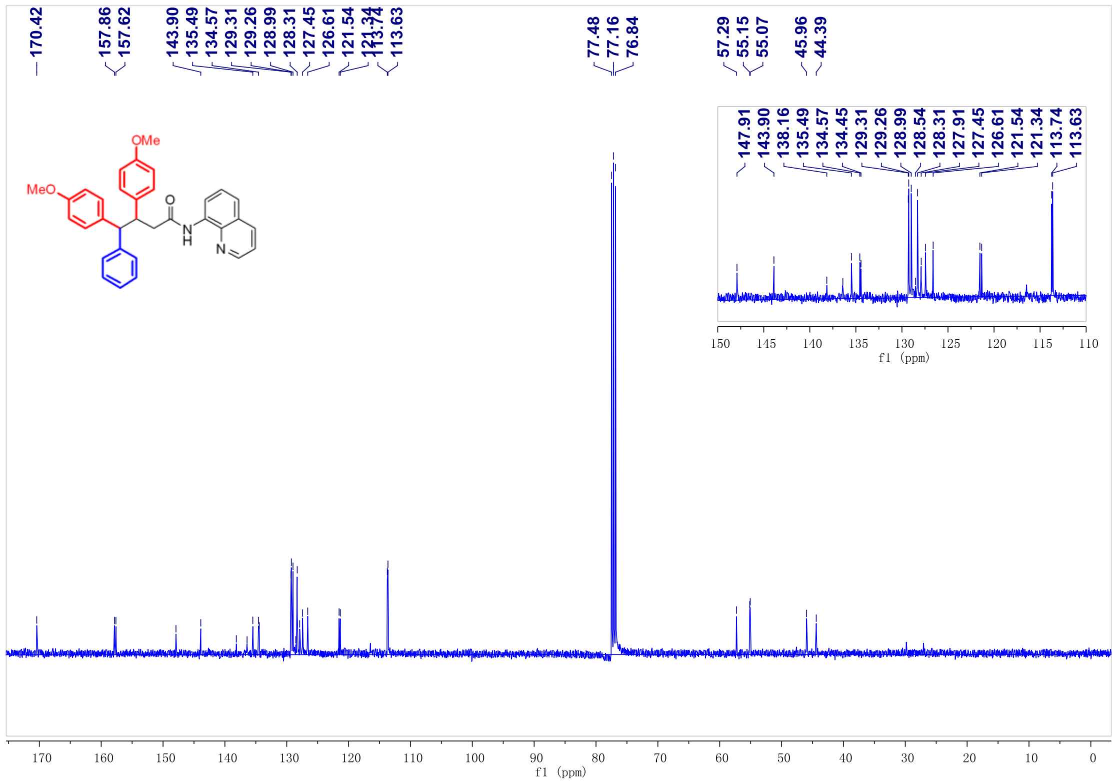
170.42
157.86
157.62
143.90
135.49
134.57
129.31
129.26
128.99
128.31
127.45
126.61
121.54
121.34
113.74
113.63
77.48
77.16
76.84
57.29
55.15
55.07
45.96
44.39
OMe
MeO
O
N
H
N
147.91
143.90
138.16
135.49
134.57
134.45
129.31
129.26
128.99
128.54
128.31
127.91
127.45
126.61
121.54
121.34
113.74
113.63
f1 (ppm)
f1 (ppm)
